# Iron(ii)-catalyzed asymmetric intramolecular olefin aminochlorination using chloride ion†
†Electronic supplementary information (ESI) available: Experimental procedure, characterization data for all new compounds, selected NMR spectra and HPLC traces. CCDC 1041826. For ESI and crystallographic data in CIF or other electronic format see DOI: 10.1039/c5sc00221d



**DOI:** 10.1039/c5sc00221d

**Published:** 2015-03-13

**Authors:** Cheng-Liang Zhu, Jun-Shan Tian, Zhen-Yuan Gu, Guo-Wen Xing, Hao Xu

**Affiliations:** a Department of Chemistry , Georgia State University , 100 Piedmont Avenue SE , Atlanta , Georgia 30303 , USA . Email: hxu@gsu.edu ; Fax: +1-404-413-5505 ; Tel: +1-404-413-5553; b Department of Chemistry , Beijing Normal University , Beijing , 100875 , China

## Abstract

An iron-catalyzed enantioselective and diastereoselective intramolecular olefin aminochlorination reaction is reported (ee up to 92%, dr up to 15 : 1). In this reaction, a functionalized hydroxylamine and chloride ion are utilized as nitrogen and chlorine sources, respectively. This new method tolerates a range of synthetically valuable internal olefins that are all incompatible with existing asymmetric olefin aminochlorination methods.

## Abstract 2

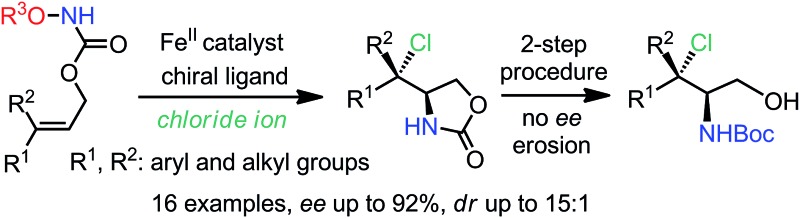
We report an iron-catalyzed asymmetric aminochlorination method for internal olefins; it tolerates valuable olefins that are incompatible with existing methods.

## Introduction

**Scheme 1:**
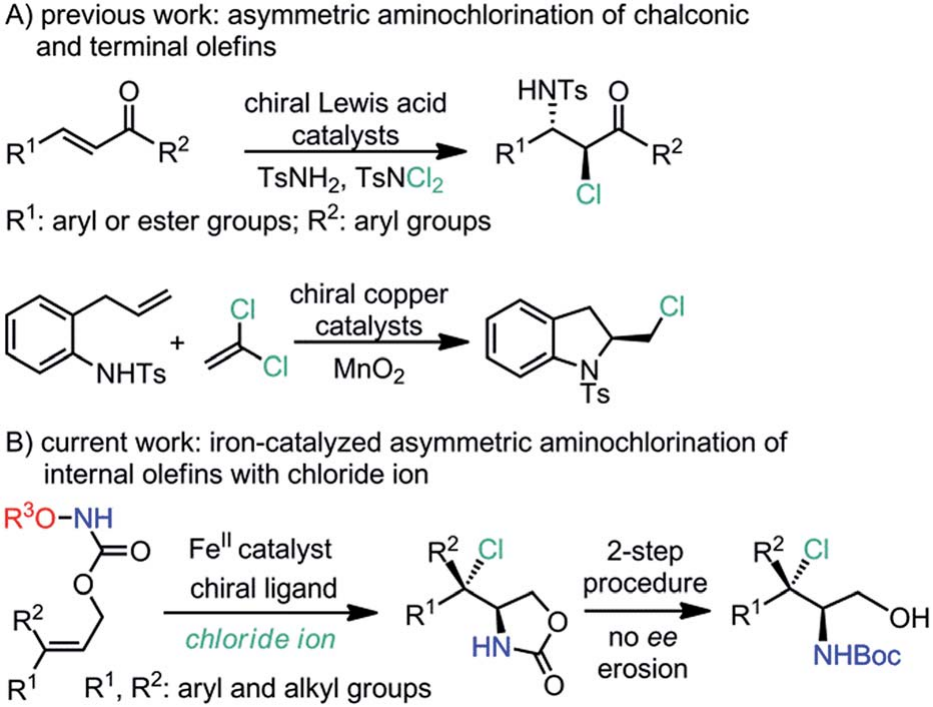
Catalytic asymmetric olefin aminochlorination: summary of this work and other existing asymmetric methods.

Enantioselective olefin halo-functionalization reactions constitute a range of synthetically valuable yet challenging transformations.^1^ Although a variety of excellent asymmetric olefin halo-oxygenation reactions have been discovered,^2^ there are much fewer asymmetric olefin aminohalogenation methods available.^3^ In particular, there have been just a few reported catalytic asymmetric olefin aminochlorination reactions.^4^ In one instance, Feng discovered the chiral Lewis acid-catalyzed aminochlorination of chalconic and other α,β-unsaturated olefins.^4a,c^ Also, Chemler reported copper-catalyzed aminochlorination of terminal olefins with chlorine radical donors in the presence of MnO_2_ (Scheme 1A).^4*b*^ Despite these and other important discoveries, catalytic asymmetric aminochlorination methods for internal, non-chalconic olefins have yet to be developed. These methods would be synthetically valuable because they would readily provide vicinal amino chlorides, a class of important chiral building blocks. Moreover, asymmetric olefin aminochlorination that proceeds through an iron-nitrenoid intermediate has not yet been reported.^5^

We previously discovered Fe(BF_4_)_2_-based catalysts for both diastereoselective and enantioselective intramolecular olefin aminofluorination reactions.^6^ Our initial attempts to apply these catalysts to olefin aminochlorination reactions led to either low diastereoselectivity or low yield, presumably due to the reason that chlorine and fluorine atom-transfer may proceed through distinct mechanisms. Therefore, we explored a range of activating group–ligand combinations and discovered entirely new catalytic conditions for asymmetric olefin aminochlorination. Herein, we describe iron-catalyzed enantioselective and diastereoselective intramolecular aminochlorination for a range of internal, non-chalconic olefins (ee up to 92%, dr up to 15 : 1). In these reactions, a functionalized hydroxylamine and chloride ion were utilized as nitrogen and chlorine sources, respectively. This method tolerates a range of synthetically valuable internal olefins that are all incompatible with existing asymmetric olefin aminochlorination approaches; it also provides a new approach that is complementary to known methods for the asymmetric synthesis of amino chlorides with contiguous stereogenic centers.

Prior to this research, Bach reported an FeCl_2_-catalyzed racemic intramolecular olefin aminochlorination method using acyl azides, TMSCl, and EtOH under ligand-free conditions.^7^ Excellent syn-selectivity was observed with styrenyl olefins (dr up to > 20 : 1). However, poor diastereoselectivity was recorded with non-styrenyl acyclic olefins (dr: 1 : 1). The new method presented here has a few unique features which complement the existing iron-catalyzed olefin aminochlorination method. First, excellent anti-selectivity has been observed across a wide range of styrenyl and non-styrenyl olefins. Second, good to excellent enantioselectivity has been achieved with a variety of internal, non-chalconic olefins (ee up to 92%). Finally, acyl azides are non-reactive under the described reaction conditions (*vide infra*), which suggests that iron-nitrenoid generation may proceed *via* different pathways compared with the known azide activation pathway.

## Results and discussion

A cinnamyl alcohol-derived acyloxyl carbamate **1** was selected as the model substrate for catalyst discovery (Table 1).^8^ In the presence of tetra-*n*-butylammonium chloride (TBAC), we observed that FeCl_2_ alone catalyzed a sluggish reaction under ligand-free conditions (entry 1, 45% yield, dr: 2 : 1).^9^ However, the FeCl_2_–phenanthroline (**L1**) complex catalyzed the anti-aminochlorination with significantly improved yield and dr (entry 2, 80% yield, dr > 20 : 1). We also noted that the Fe(NTf_2_)_2_–**L1** complex provided essentially the same reactivity and diastereoselectivity (entry 3, 86% yield, dr > 20 : 1). Interestingly, the Fe(NTf_2_)_2_–bisoxazoline (**L2**) complex resulted in a loss of diastereoselectivity (entry 4, 82% yield, dr: 0.83 : 1). Furthermore, the Fe(NTf_2_)_2_–**L3** complex promoted the syn-aminochlorination with moderate yield and dr (entry 5, 34% yield, dr: 0.25 : 1). We also observed that the Fe(NTf_2_)_2_–**L4** complex catalyzed the anti-aminochlorination with a modest dr (entry 6, 75% yield, dr: 1.8 : 1). Notably, an iron–**L4** complex resulted in high dr and reaction rate in the previously reported olefin aminofluorination reaction.^6^ These observations suggest that ligands are involved in the diastereoselectivity-determining step and provide excellent opportunities for diastereo-control.

**Table 1:** Catalyst discovery for the iron-catalyzed diastereoselective olefin aminochlorination reaction

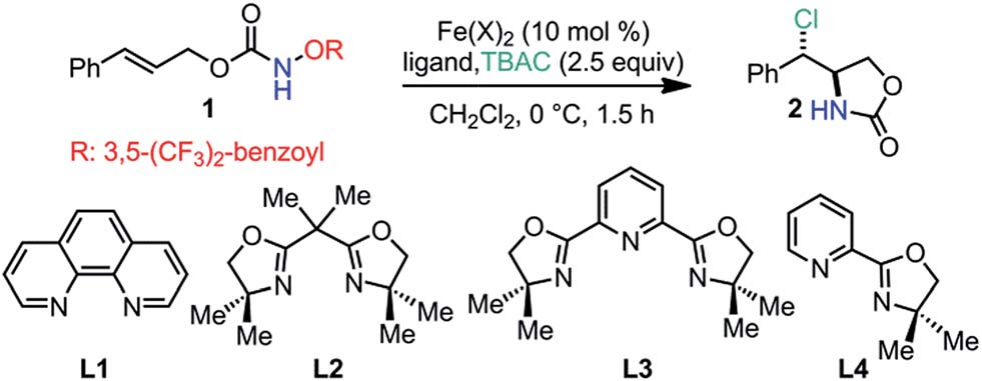					
Entry^*a*^	Fe(X)_2_	Ligand (mol%)	Conversion^*b*^	Yield^*c*^	dr^*b*^ (anti : syn)
1	FeCl_2_	None	62%	45%	2 : 1
2	FeCl_2_	**L1** (20)	>95%	80%	>20 : 1
3	Fe(NTf_2_)_2_	**L1** (20)	>95%	86%	>20 : 1
4	Fe(NTf_2_)_2_	**L2** (10)	>95%	82%	0.83 : 1
5	Fe(NTf_2_)_2_	**L3** (10)	61%	34%	0.25 : 1
6	Fe(NTf_2_)_2_	**L4** (20)	>95%	75%	1.8 : 1

^*a*^Unless stated otherwise, the reactions were carried out under a nitrogen atmosphere. TBAC: tetra-*n*-butylammonium chloride.

^*b*^Conversion and dr were determined by ^1^H NMR.

^*c*^Isolated yield.

**Scheme 2:**
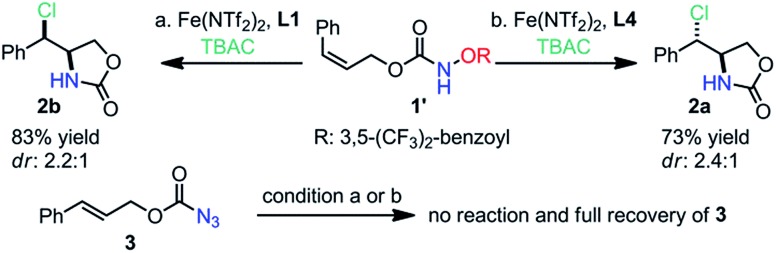
Iron-catalyzed aminochlorination with a *cis* olefin and an acyl azide. ^*a*^Reaction conditions: Fe(NTf_2_)_2_ (10 mol%), **L1** (20 mol%), TBAC (2.5 equiv.), CH_2_Cl_2_, 0 °C, 2 h. ^*b*^Reaction conditions: Fe(NTf_2_)_2_ (10 mol%), **L4** (20 mol%), TBAC (2.5 equiv.), CH_2_Cl_2_, 0 °C, 2 h.

The observed ligand-enabled diastereo-control with *trans*-olefin **1** prompted us to evaluate *cis*-olefin **1′** (Scheme 2). To our surprise, the Fe(NTf_2_)_2_–**L1** complex catalyzed syn-aminochlorination, while the Fe(NTf_2_)_2_–**L4** complex promoted anti-aminochlorination with essentially the same dr (Scheme 2). The different reaction profiles for isomeric olefins **1** and **1′** suggest that the aminochlorination reaction is neither stereospecific nor fully stereo-convergent, which is significantly different from the iron-catalyzed olefin aminofluorination reaction.^6^

Furthermore, an acyl azide **3** was evaluated under the reaction conditions as a control experiment. Interestingly, the acyl azide **3** was fully recovered and no aminochlorination product was detected. These results suggest that the activation of acyloxyl carbamates (**1** and **1′**) may proceed *via* different pathways compared with the known azide activation pathway.^7^

We subsequently explored a range of olefins under the optimized conditions to evaluate the scope and limitations of this anti-aminochlorination method (Table 2). We discovered that di-substituted styrenyl olefins are generally good substrates; both electron-donating and electron-withdrawing substituents are compatible with this method (entries 1–4). Importantly, *ortho*-substituents and pyridyl groups are both tolerated (entries 5–6). Furthermore, extended aromatics, including naphthyl olefins, are reasonable substrates (entries 7–8). Moreover, isomeric ene–ynes are both excellent substrates for the stereo-convergent and anti-selective method (entry 9). Additionally, we observed that both styrenyl and non-styrenyl tri-substituted olefins undergo aminochlorination smoothly with excellent dr (entries 10–11).^10^ We also discovered that a cyclohexyl-substituted olefin was an excellent substrate (entry 12, dr > 20 : 1). Further exploration revealed that both 1,1-disubstituted olefins and dienes are viable substrates with excellent regioselectivity (entries 13–14). Most notably, a cyclic olefin could also undergo highly diastereoselective anti-aminochlorination (entry 15, dr > 20 : 1), yielding a product which is difficult to obtain with known methods.^11^ Since the FeCl_2_–**L1** complex provides essentially the same dr and yield in these diastereoselective reactions, FeCl_2_ can be a convenient substitute for Fe(NTf_2_)_2_ in racemic reactions.

**Table 2:** Substrate scope of the iron-catalyzed diastereoselective olefin aminochlorination reaction

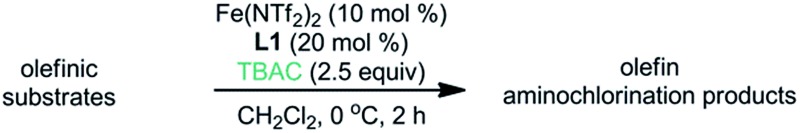
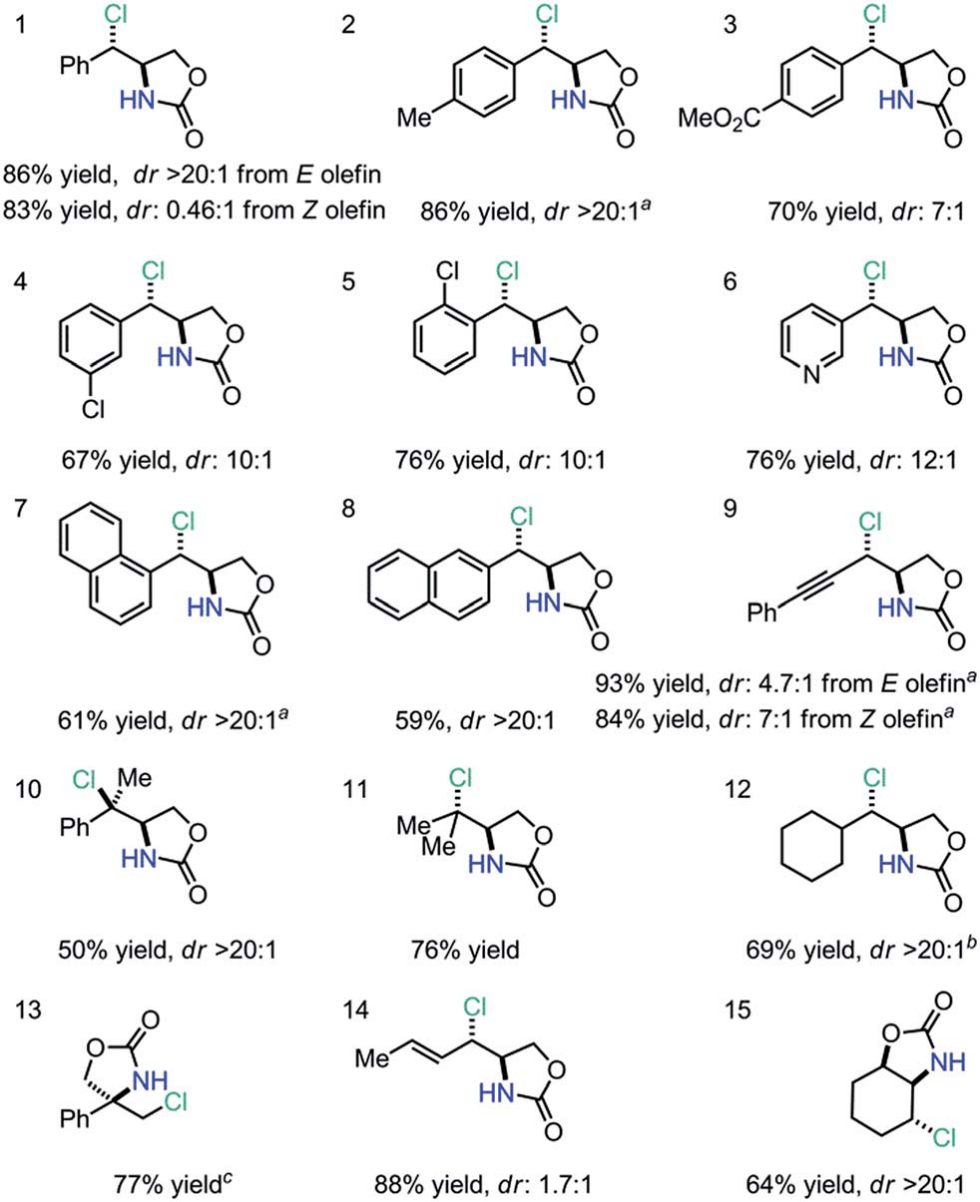

^*a*^Reaction conditions: –15 °C, 2 h.

^*b*^Reaction conditions: 0 °C, 5 h.

^*c*^Reaction conditions: 0 °C, 12 h.

In order to fulfil the need for catalytic asymmetric olefin aminochlorination, we further explored asymmetric induction for internal, non-chalconic olefins with a variety of iron–chiral ligand complexes (Table 3).^12^ First, we discovered that the iron–**L5** complex induced diastereoselective and enantioselective anti-aminochlorination, albeit with a low yield, mostly due to the competing aminohydroxylation reaction (entry 1, 53% yield, dr: 9.9 : 1). Interestingly, the anti-addition product **2a** was obtained with excellent ee (84% ee), while the syn-addition product **2b** was obtained essentially as a racemate (<5% ee).^13^ Additionally, a two-step procedure can convert **2a** to a chlorinated amino alcohol triad **4** without ee erosion.^14^ Next, we observed that the iron–**L6** complex induced moderately diastereoselective syn-aminochlorination (entry 2, 68% yield, dr: 0.48 : 1). To our surprise, the anti-addition product **2a** was obtained with moderate ee (24% ee), while the syn-addition product **2b** was isolated with significant ee (79% ee). Furthermore, we evaluated chiral ligands **L7** and **L8** and determined that they are less effective for asymmetric induction (entries 3–4). Additionally, chiral ligand **L9** induced fast yet non-selective aminochlorination with a high overall yield (entry 5).^15^ With the iron–**L5** complex in hand, we subsequently explored other reaction parameters. First, a decreased reaction temperature was found to benefit both dr and ee (entry 6, dr: 11 : 1 and 90% ee for **2a** at –60 °C). Next, replacing the 3,5-bis(trifluoromethyl)benzoyl activating group with a smaller acetyl group further enhanced the ee (entry 7, 97% ee for **2a**); however, much lower dr and yield were obtained (entry 7, dr: 1.1 : 1, 42% yield). Finally, a chloroacetyl activating group induced an effective balance between overall yield and stereoselectivity (entry 8, 67% yield, dr: 9.6 : 1 and 89% ee for **2a**). We also observed that the FeCl_2_–**L5** complex induced a slightly less selective reaction with a lower yield (entry 9, 58% yield, dr: 9.0 : 1 and 83% ee for **2a**).

**Table 3:** Catalyst discovery for the iron-catalyzed asymmetric olefin aminochlorination reaction

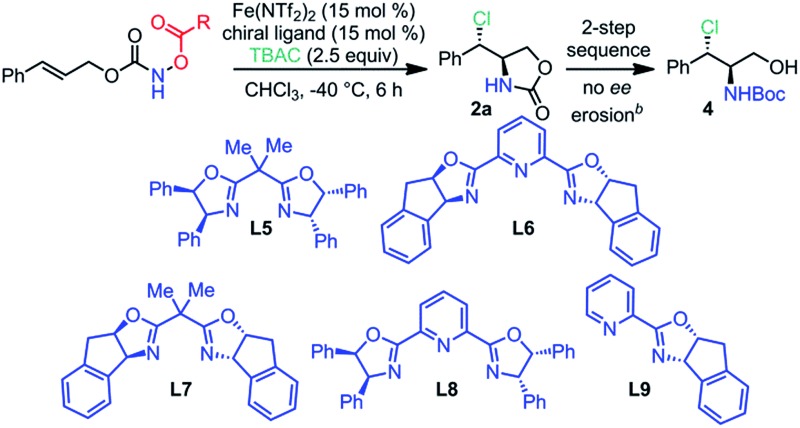							
Entry^*a*^	R	Ligand	Conversion^*c*^	Yield^*d*^	dr^*c*^ (anti : syn)	ee^*e*^ (anti)	ee^*e*^ (syn)
1	3,5-(CF_3_)_2_-Ph	**L5**	>95%	53%	9.9 : 1	84%	<5%
2	3,5-(CF_3_)_2_-Ph	**L6**	>95%	68%	0.5 : 1	24%	79%
3	3,5-(CF_3_)_2_-Ph	**L7**	88%	61%	1.7 : 1	<5%	<5%
4	3,5-(CF_3_)_2_-Ph	**L8**	>95%	32%	2.5 : 1	47%	30%
5	3,5-(CF_3_)_2_-Ph	**L9**	>95%	82%	0.5 : 1	8%	24%
6^*f*^	3,5-(CF_3_)_2_-Ph	**L5**	>95%	51%	11.0 : 1	90%	<5%
7^*f*^	CH_3_	**L5**	>95%	42%	1.1 : 1	97%	<5%
8^*f*^	CH_2_Cl	**L5**	>95%	67%	9.6 : 1	89%	<5%
9^*f*^ ^,^^*g*^	CH_2_Cl	**L5**	>95%	58%	9.0 : 1	83%	<5%

^*a*^Unless stated otherwise, the reactions were carried out under a nitrogen atmosphere with 4 Å molecular sieves.

^*b*^Reaction conditions: Boc_2_O, Et_3_N, DMAP; then Cs_2_CO_3_, MeOH, 85% over two steps; see ESI for details.

^*c*^Conversion and dr were determined by ^1^H NMR.

^*d*^Isolated yield.

^*e*^Enantiomeric excess (ee) was measured by HPLC with chiral columns; the absolute stereochemistry was determined by X-ray crystallographic analysis of an analog of **2a**.

^*f*^The reaction was carried out at –60 °C for 12 h.

^*g*^The FeCl_2_–**L5** complex was used.

In order to evaluate the scope of this asymmetric method, we explored the asymmetric induction with a range of internal olefins (Table 4). The chiral catalyst provides excellent asymmetric induction with styrenyl olefins. A range of *para*-substituted styrenyl olefins with different electronic properties were converted to the corresponding aminochlorination products with high dr and ee (entries 1–6, dr: 9.6–15 : 1, ee: 86–91%). Additionally, *meta*-substituted styrenyl olefins are also good substrates but with slightly decreased ee (entries 7–9, dr: 10–15 : 1, ee: 80–87%). However, we discovered that *ortho*-substitution of styrenes has a deleterious effect on ee (entries 10–11, dr: 4.5–12 : 1, ee: 77–79%). Interestingly, both α- and β-naphthyl olefins are excellent substrates (entries 12–13, dr: 4.5–10 : 1, ee: 89–92%). To our delight, a 3-pyridyl olefin with a basic nitrogen atom is a reasonable substrate for the asymmetric aminochlorination (entry 14, dr: 1.8 : 1, ee: 70% for the anti-diastereomer). Moreover, we observed that the iron–**L5** complex can induce significant ee in the aminochlorination with non-styrenyl olefins (entry 15, dr: 2 : 1, ee: 54% for the anti-diastereomer). To our surprise, the iron–**L6** complex proved to be uniquely effective for the asymmetric induction with tri-substituted olefins, while the iron–**L5** complex was less effective (entry 16, dr: 2.3 : 1, ee: 86% for the anti-diastereomer).^16^

**Table 4:** Substrate scope for the iron-catalyzed asymmetric olefin aminochlorination reaction

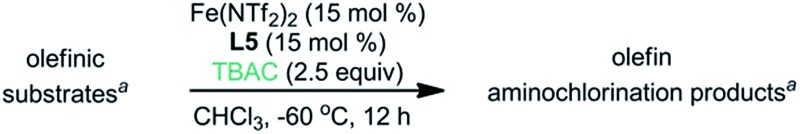
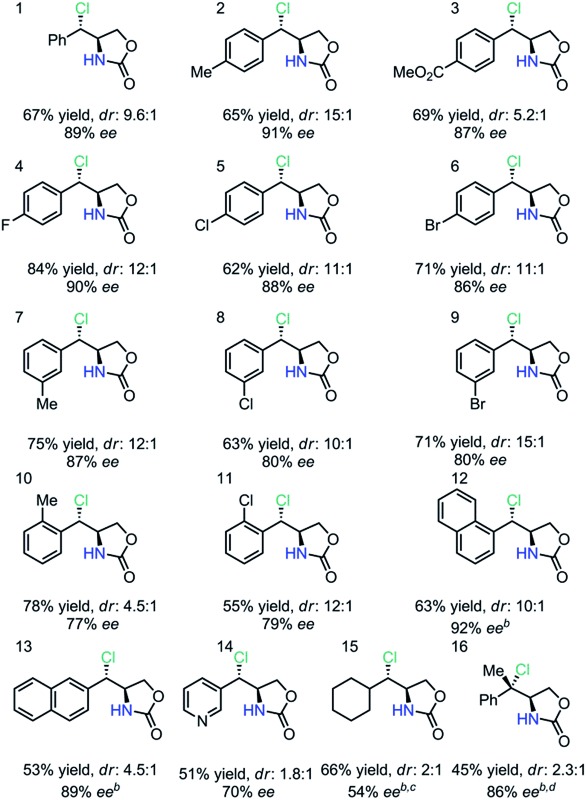

^*a*^Unless stated otherwise, mono-chloroacetyl was selected as the activating group for asymmetric catalysis; the ee for all syn-aminochlorination products was less than 5%.

^*b*^Bis(trifluoromethyl)-benzoyl was selected as the activating group.

^*c*^The ee for the syn-addition product was 12%.

^*d*^ **L6** was used as the ligand for asymmetric induction; the ee for the syn-addition product was 50%.

**Scheme 3:**
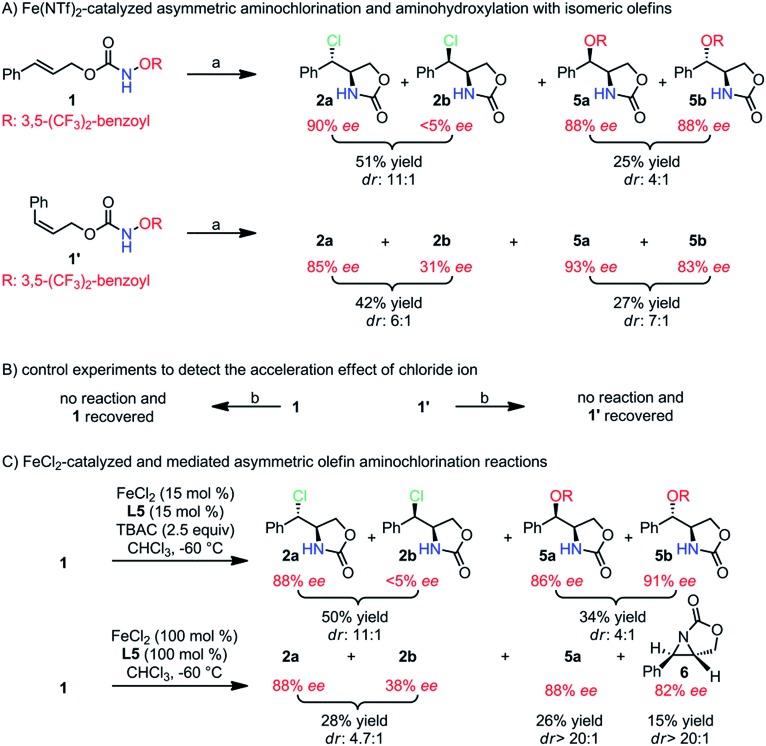
Control experiments to probe the mechanism. ^*a*^Reaction conditions: Fe(NTf_2_)_2_ (15 mol%), **L1** (15 mol%), TBAC (2.5 equiv.), CHCl_3_, –60 °C, 12 h. ^*b*^Reaction conditions: Fe(NTf_2_)_2_ (15 mol%), **L1** (15 mol%), CHCl_3_, –60 °C, 12 h.

During the exploration of substrate scope, it was surprising to observe completely different ee values for anti- and syn-diastereomers (*e.g.***2a** and **2b**). In contrast, exactly the same ee for both diastereomeric products was observed in the iron-catalyzed aminofluorination of **1**.^6^ In order to obtain greater mechanistic insights, we carried out ee analysis for all isolable products using several control experiments (Scheme 3). First, in an Fe(NTf_2_)_2_-catalyzed reaction with *trans*-olefin **1**, two aminochlorination products were obtained (Scheme 3A, 90% ee for **2a**, <5% ee for **2b**, dr: 11 : 1).^17^ Simultaneously, diastereomers **5a** and **5b** were also isolated with the same ee as two competing olefin aminohydroxylation products (Scheme 3A, 88% ee for **5a** and **5b**, dr: 4 : 1). However, completely different selectivity (both dr and ee) was observed in an Fe(NTf_2_)_2_-catalyzed reaction with *cis*-olefin **1′** (Scheme 3A, 85% ee for **2a** and 31% ee for **2b**, dr: 6 : 1; 93% ee for **5a** and 83% ee for **5b**, dr: 7 : 1). In both cases, **5a** and **5b** cannot be converted to **2a** under the reaction conditions.

These observations provide several important mechanistic insights. First, the non-stereospecificity observed in the iron-catalyzed olefin aminochlorination suggests that the formation of C–N and C–Cl bonds occurs in a stepwise fashion.^18^ Second, the lack of complete stereo-convergence between the reaction profiles of isomeric olefins (**1** and **1′**) suggests that C–N bond formation may be the rate- and ee-determining step.^18^ Furthermore, since essentially the same ee was observed for **2a**, **5a**, and **5b** from the reaction with *trans*-olefin **1**, it is likely that these products are derived from the same intermediate after the ee-determining step. Additionally, the fact that the syn-aminochlorination product **2b** was isolated as a racemate suggests that **2b** may be derived from non-stereoselective pathways which are distinct from the one leading to the formation of **2a**, **5a**, and **5b**.

The product divergence (**2a***vs.***5a**/**b**) after the ee-determining step is mechanistically interesting. Therefore, we studied the effect of external chloride ion. To our surprise, in the absence of TBAC, the Fe(NTf_2_)_2_–**L5** complex alone was ineffective for the nitrogen atom-transfer at –60 °C; **1** and **1′** were both fully recovered (Scheme 3B). However, aminochlorination occurred as soon as a stoichiometric amount of TBAC was introduced. This observation suggests that the Fe(NTf_2_)_2_–**L5** complex may serve as a pre-catalyst and it may be activated by chloride ion *in situ*.

In order to test this hypothesis, we further carried out the FeCl_2_-catalyzed reaction in the presence of TBAC (Scheme 3C). Notably, **2a** was isolated with essentially the same ee as that obtained under the standard conditions (88% ee for **2a** and <5% ee for **2b**). This result suggests that the catalytically relevant species may also be generated from the FeCl_2_–**L5** complex.

To probe for more mechanistic details, we subsequently carried out the FeCl_2_-promoted olefin aminochlorination in the absence of TBAC (100 mol% FeCl_2_, 100 mol% **L5**, Scheme 3C). Under these conditions, FeCl_2_ is the only available chlorine source. Surprisingly, we discovered that **2a** was obtained with essentially the same ee compared with the two previous control experiments (88% ee for **2a**). Furthermore, a syn-aminohydroxylation product **5a** was isolated with excellent dr and ee (dr > 20 : 1, 88% ee). These observations suggest that Fe–Cl bond cleavage may be relevant for the chlorine atom-transfer step during the enantioselective anti-aminochlorination.^19^ In addition, we also identified a small amount of aziridine **6** (15% yield, 82% ee), and further discovered that it could not be converted to either **2a** or **5a** under the reaction conditions.

**Scheme 4:**
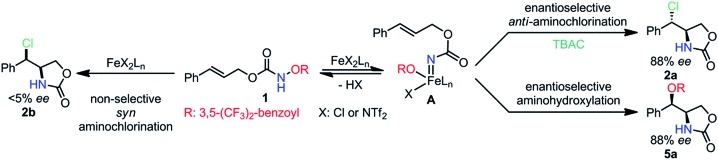
Proposed mechanistic working hypothesis for the iron-catalyzed asymmetric aminochlorination of *trans*-olefin **1**.

With the accumulated mechanistic evidence, we propose a plausible mechanistic working hypothesis for the iron-catalyzed asymmetric aminochlorination of *trans*-olefin **1** (Scheme 4). First, the iron catalyst reversibly cleaves the N–O bond in the acyloxyl carbamate **1**, generating iron-nitrenoid **A** with chloride as a counter ion. From there, **A** may participate in enantioselective and diastereoselective aminochlorination and aminohydroxylation to afford **2a** and **5a**, respectively. Since the aminochlorination–aminohydroxylation competition occurs after the ee-determining step, **2a** is obtained with essentially the same ee as **5a**. At the same time, **1** may be converted to **2b***via* a non-stereoselective pathway which is distinct from the one leading to the formation of **2a** and **5a**. Further mechanistic studies are required to elucidate the details.

## Conclusions

In conclusion, we have described an iron-catalyzed enantioselective and diastereoselective aminochlorination method for internal, non-chalconic olefins. This method tolerates a range of synthetically valuable olefins that are all incompatible with existing asymmetric olefin aminochlorination methods. It also provides a complementary approach for the asymmetric synthesis of amino chlorides with contiguous stereogenic centers. Our preliminary mechanistic studies revealed that an FeCl_2_-derived nitrenoid may be a feasible reactive intermediate and that Fe–Cl bond cleavage may be relevant for stereoselective chlorine atom-transfer. Our current efforts are focused on the mechanistic investigation of this new reaction and method development for the enantioselective intermolecular olefin aminochlorination.

## Supplementary Material

Supplementary information

Crystal structure data

## References

[cit1] Denmark S. E., Kuester W. E., Burk M. T. (2012). Angew. Chem., Int. Ed..

[cit2] Kang S. H., Lee S. B., Park C. M. (2003). J. Am. Chem. Soc..

[cit3] Cai Y., Liu X., Hui Y., Jiang J., Wang W., Chen W., Lin L., Feng X. (2010). Angew. Chem., Int. Ed..

[cit4] Cai Y. F., Liu X. H., Jiang J., Chen W. L., Lin L. L., Feng X. M. (2011). J. Am. Chem. Soc..

[cit5] Liu G.-S., Zhang Y.-Q., Yuan Y.-A., Xu H. (2013). J. Am. Chem. Soc..

[cit6] Lu D.-F., Liu G.-S., Zhu C.-L., Yuan B., Xu H. (2014). Org. Lett..

[cit7] Bach T., Schlummer B., Harms K. (2000). Chem. Commun..

[cit8] See ESI for details of substrate synthesis. Acyloxyl carbamates are reactive, while tosyloxyl and alkoxyl carbamates are non-reactive and fully recovered under the reaction conditions

[cit9] The relative stereochemistry of **2a** was determined by comparison of the experimental NMR data with those reported in ref. 7. It was further corroborated by ^1^H NMR and X-ray crystallographic analysis of a structural analog of **2a**. See ESI for details

[cit10] The relative stereochemistry was assigned based on the ^1^H NMR and X-ray crystallographic analysis of a structural analog described in ref. 6; see ESI for details

[cit11] Complementary stereochemistry was achieved (in entry 15 of Table 2) compared with the known method reported in ref. 7, where the syn-aminochlorination product was isolated. This substrate did not undergo kinetic resolution with a chiral catalyst, the complex Fe(NTf_2_)_2_–**L5**. Both the starting material and product were isolated as racemates

[cit12] Evans D. A., Woerpel K. A., Hinman M. M., Faul M. M. (1991). J. Am. Chem. Soc..

[cit13] The absolute stereochemistry of **2a** was determined by X-ray crystallographic analysis of a structural analog of **2a**. See ESI for details

[cit14] For detailed procedure and HPLC traces of **4**, see ESI.

[cit15] For the synthesis of **L9**, see ref. 6

[cit16] The iron–**L5** complex catalyzed the reaction favoring the syn-addition product (dr (anti/syn): 0.47 : 1); ee for the anti-addition product was 60% and ee for the syn-addition product was <5%. The relative stereochemistry was assigned based on the ^1^H NMR and X-ray crystallographic analysis of a structural analog described in ref. 6; see ESI for details

[cit17] When a chloroacetyl group was used as the activating group, a different result was obtained. For details, see entry 8 of Table 3

[cit18] For an example of stepwise atom transfer reactions with different reaction profiles for *cis*/*trans* isomeric olefins, see: Lee N. H. Jacobsen E. N., Tetrahedron Lett., 1991, 32 , 6533 .

[cit19] Kharasch M. S., Sosnovsky G. (1958). J. Am. Chem. Soc..

