# Supplementary material for: Iron(ii)-catalyzed asymmetric intramolecular olefin aminochlorination using chloride ion
Source: Chem Sci. 2015 Mar 13;6(5):3044–50. doi: 10.1039/c5sc00221d (PMC4719767; doi:10.1039/c5sc00221d)
Supplement: Supplementary file 1 [file SC-006-C5SC00221D-s001.pdf]

Electronic Supplementary Material (ESI) for Chemical Science.
This journal is © The Royal Society of Chemistry 2015

Iron(II) Catalyzed Asymmetric Intramolecular Olefin Aminochlorination with Chloride Ion

Cheng-Liang Zhu,[a] Jun-Shan Tian,[a] Zhen-Yuan Gu,[a,b] Guo-Wen Xing,[b] and Hao Xu*[a]

[a]*Department of Chemistry, Georgia State University, Atlanta GA 30303, United States*

[b]*Department of Chemistry, Beijing Normal University, Beijing, 100875, China*

Supporting Material

A. General Information

B. Catalyst Discovery and Procedures for the Iron-Catalyzed Diastereoselective Olefin Aminochlorination Reaction

a. Synthesis of New Nitrogen-Based Ligands and Catalyst Discovery for the Iron-Catalyzed Diastereoselective Olefin Aminochlorination

b. Synthesis and Characterization of New Substrates

c. General Procedure for the Iron-Catalyzed Diastereoselective Olefin Aminochlorination and Product Characterization

C. Catalyst Discovery and Procedures for the Iron-Catalyzed Asymmetric Olefin Aminochlorination Reaction

a. Catalyst Discovery for the Iron-Catalyzed Asymmetric Olefin Aminochlorination

b. Synthesis and Characterization of New Substrates

c. General Procedure for the Iron-Catalyzed Asymmetric Olefin Aminochlorination and Product Characterization

D. Mechanistic Investigation of the Iron-Catalyzed Asymmetric Olefin Aminochlorination

E. References

F. NMR Spectra

A. General Information

General Procedures. All reactions were performed in flame-dried round-bottom flasks and vials. Stainless steel syringes and cannula were used to transfer air- and moisture-sensitive liquids. Flash chromatography was performed using silica gel 60 (230-400 mesh) from Sigma–Aldrich.

Materials. Tetra-*n*-butylammonium chloride (TBAC) was purchased from Sigma–Aldrich. It was further purified through recrystallization in the diethyl ether/acetone mixture and stored in a glove box under N_2 atmosphere. Other reagents were purchased from Sigma–Aldrich, Fluka, EM Science, and Lancaster and used directly as received. All solvents were used after being freshly distilled.

Instrumentation. Proton nuclear magnetic resonance (^{1}H NMR) spectra, carbon nuclear magnetic resonance (^{13}C NMR) spectra were recorded on Bruker UltraShield-400 (400 MHz). Chemical shifts for protons are reported in parts per million downfield from tetramethylsilane and are referenced to the NMR solvent residual peak ($CHCl_3$: δ 7.26). Chemical shifts for carbons are reported in parts per million downfield from tetramethylsilane and are referenced to the carbon resonances of the NMR solvent ($CDCl_3$: δ 77.0). Data are represented as follows: chemical shift, multiplicity (br = broad signal, s = singlet, d = doublet, t = triplet, q = quartet, m = multiplet, dd = doublet of doublets, dt = doublet of triplets), coupling constants in Hertz (Hz), and integration. The mass spectroscopic data were obtained at the Georgia State University mass spectrometry facility using a Micromass Platform II single quadruple instrument. Infrared (IR) spectra were obtained using a Perkin Elmer Spectrum 100 FT-IR spectrometer. Data are represented as follows: frequency of absorption (cm^{-1}) and absorption strength (s = strong, m = medium, w = weak).

Abbreviations. EtOH–ethanol, EtOAc–ethyl acetate, THF–tetrahydrofuran, MeOH–methanol, Et_2O–diethyl ether, CH_2Cl_2–dichloromethane, TEA–triethylamine, MS–molecular sieves, CDI–1,1′-carbonyldiimidazole, DCC–*N*,*N*′-dicyclohexylcarbodiimide, DCU–*N*,*N*′-dicyclohexylurea, TLC–thin layer chromatography, DMAP−4-dimethylaminopyridine, TBAC−tetra-*n*-butylammonium chloride, Bz−benzoyl.

B. Catalyst Discovery and Procedures for the Iron-Catalyzed Diastereoselective Olefin Aminochlorination Reaction

a. Synthesis of New Nitrogen-Based Ligands and Catalyst Discovery for the Iron-Catalyzed Diastereoselective Olefin Aminochlorination

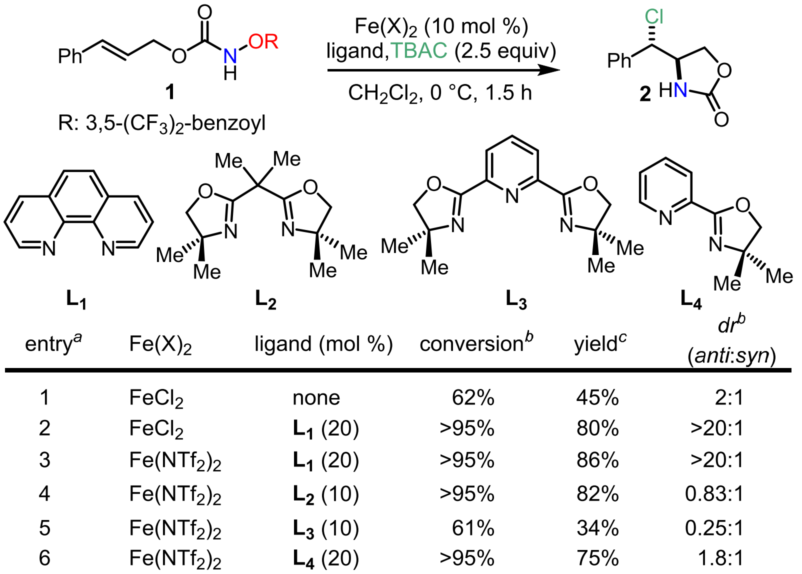

entry[a]	Fe(X)$_2$	ligand (mol %)	conversion[b]	yield[c]	dr[b] (anti:syn)
1	$FeCl_2$	none	62%	45%	2:1
2	$FeCl_2$	**L$_1$** (20)	>95%	80%	>20:1
3	Fe(NTf$_2$)$_2$	**L$_1$** (20)	>95%	86%	>20:1
4	Fe(NTf$_2$)$_2$	**L$_2$** (10)	>95%	82%	0.83:1
5	Fe(NTf$_2$)$_2$	**L$_3$** (10)	61%	34%	0.25:1
6	Fe(NTf$_2$)$_2$	**L$_4$** (20)	>95%	75%	1.8:1

[a]Unless stated otherwise, the reactions were carried out under N_2 atmosphere. [b]Conversion and *dr* are determined by ^{1}H NMR. [c]Isolated yield. TBAC: tetra-*n*-butylammonium chloride.

Table S1. Catalyst discovery for the iron-catalyzed diastereoselective olefin aminochlorination reaction

L1 was purchased from Sigma–Aldrich and used directly without further purification. **L2**, **L3**, and **L4** were synthesized according to literature procedures.[1-3]

Procedure for Catalyst Discovery. To a flame-dried sealable 2-dram vial (vial **A**) equipped with a magnetic stir bar were added an iron catalyst (0.02 mmol) and a ligand (0.02 or 0.04 mmol). After the vial was evacuated and backfilled with N_2 for three times, anhydrous CH_2Cl_2 (1.0 mL) was added and the mixture was stirred at room temperature for 20 min. During this time, substrate **1** (0.2 mmol, 86 mg) and anhydrous TBAC (139 mg, 0.5 mmol) were dissolved in CH_2Cl_2 (4.0 mL) in a second flame-dried 3-dram vial (vial **B**) with a magnetic stir bar under N_2 atmosphere. Both vials were degassed by brief evacuation and back filling with N_2 twice. The vial **B** was cooled down to 0 ° C, and the solution in vial **A** was added to vial **B** drop wise via a syringe. The resulting solution was stirred at the same temperature until **1** was fully consumed

monitored by TLC. The reaction was quenched with 1 mL saturated $NaHCO_3$ solution and extracted with CH_2Cl_2 (1.5 mL × 3). The combined organic phase was concentrated and the residue was purified through a gradient silica gel flash column chromatography (hexanes/acetone: from 15:1 to 4:1) to afford the aminochlorination product **2** as a white solid. The *dr* was determined by ^{1}H NMR analysis of the crude reaction mixture.

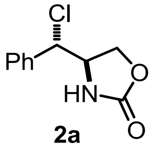

2a

4-(Chloro(phenyl)methyl)oxazolidin-2-one (2a): by following the general procedure under the condition described in entry 3, **2a** was obtained as a white solid (36 mg, 86% yield, *dr* >20:1, m.p. 90–93 °C). ^{1}H NMR (400 MHz, $CDCl_3$): δ 7.44–7.38 (m, 5H), 4.93 (s, 1H), 4.72 (d, *J* = 9.1 Hz, 1H), 4.62 (dd, *J* = 9.3, 8.1 Hz, 1H), 4.48 (dd, *J* = 9.4, 4.8 Hz, 1H), 4.27 (td, *J* = 8.6, 4.8 Hz, 1H); ^{13}C NMR (100 MHz, $CDCl_3$): δ 158.1, 136.6, 129.7, 129.3, 127.7, 68.6, 63.4, 58.3; IR ν_{max} (neat)/cm^{-1}: 3237 (m), 3139 (w), 2918 (w), 2853 (w), 1736 (s), 1715 (s), 1480 (m), 1402(m), 1237 (s), 1034 (s), 1012 (s), 931 (m), 768 (m); HRMS (ESI, m/z): calcd for $C_{10}H_{11}NO_2Cl^+$ (M + H^+), 212.0478, found 212.0485.

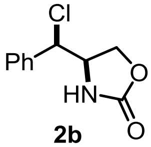

2b

4-(Chloro(phenyl)methyl)oxazolidin-2-one (2b): by following the general procedure under the condition described in entry 4, **2a** and **2b** were obtained as a mixture (34 mg, 82% yield, *dr*: 0.83:1, m.p. 91–99 °C). **2b** is characterized as following: ^{1}H NMR (400 MHz, $CDCl_3$): δ 7.47–7.32 (m, 5H), 6.21 (s, 1H), 4.80 (d, *J* = 8.7 Hz, 1H), 4.29 (td, *J* = 8.4, 4.7 Hz, 1H), 4.20 (dd, *J* = 8.5, 1H), 4.00 (dd, *J* = 9.3, 4.8 Hz, 1H); ^{13}C NMR (100 MHz, $CDCl_3$): δ 158.1, 136.6, 129.7, 129.3, 127.7, 68.6, 63.4, 58.3; IR ν_{max} (neat)/cm^{-1}: 3230 (m), 3131 (w), 2915 (w), 2853 (w), 1734 (s), 1713 (s), 1475 (m), 1409(m), 1234 (s), 1034 (s), 1012 (s), 930 (m), 770 (m); HRMS (ESI, m/z): calcd for $C_{10}H_{11}NO_2Cl^+$ (M + H^+), 212.0478, found 212.0485.

Relative Stereochemistry Determination. The relative stereochemistry of **2** was determined by comparison of the NMR spectra of **2a** and **2b** with literature precedents, in which **2a** and **2b** were both characterized.[4]

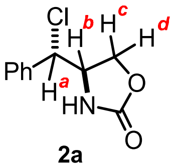

2a

compound	**2a**	**2-*anti*** (literature data)[4]
^{1}H NMR	^{1}H NMR (400 MHz, $CDCl_3$): δ 7.44–7.38 (m, 5H), 4.93 (s, 1H), 4.72 (d, J = 9.1 Hz, H_a), 4.62 (dd, J = 9.3, 8.1 Hz, H_c), 4.48 (dd, J = 9.4, 4.8 Hz, H_d), 4.27 (td, J = 8.6, 4.8 Hz, H_b).	^{1}H NMR (200 MHz, $CDCl_3$): δ 7.70–7.40 (m, 5H), 5.90 (s, 1H), 4.89 (d, J = 8.5 Hz, H_a), 4.66 (dd, J = 8.5 Hz, H_c), 4.58 (dd, J = 8.5, J = 5.0 Hz, H_d), 4.40 (ddd, J = 8.5, 5.0 Hz, H_b).
^{13}C NMR	^{13}C NMR (100 MHz, $CDCl_3$): δ 158.1, 136.6, 129.7, 129.3, 127.7, 68.6, 63.4, 58.3.	^{13}C NMR (50 MHz, $CDCl_3$): δ 158.7 (s), 136.4 (s), 129.4 (d), 129.1 (d), 127.6 (d), 68.1 (t), 63.5 (d), 58.2 (d).

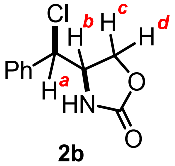

2b

compound	**2b**	**2-*syn*** (literature data)[4]
^{1}H NMR	^{1}H NMR (400 MHz, $CDCl_3$): δ 7.47–7.32 (m, 5H), 6.21 (s, 1H), 4.80 (d, J = 8.7 Hz, H_a), 4.29 (td, J = 8.4, 4.7 Hz, H_b), 4.20 (dd, J = 8.5 Hz, H_c), 4.00 (dd, J = 9.3, 4.8 Hz, H_d).	^{1}H NMR (200 MHz, $CDCl_3$): δ 7.50–7.25 (m, 5H), 5.95 (s, 1H), 4.82 (d, J = 8.5 Hz, H_a), 4.34 (ddd, J = 8.5, 8.5, 4.7 Hz, H_b), 4.24 (dd, J = 8.5 Hz, H_c), 4.03 (dd, J = 8.5, 4.7 Hz, H_d).
^{13}C NMR	^{13}C NMR (100 MHz, $CDCl_3$): δ 158.4, 135.9, 129.6, 129.2, 127.6, 66.9, 65.0, 58.2.	^{13}C NMR (50 MHz, $CDCl_3$): δ 158.9 (s), 135.9 (s), 129.4 (d), 129.0 (d), 127.5 (d), 66.9 (t), 64.8 (d), 58.6 (d).

Summary: the diagnostic ^{1}H NMR signal to differentiate **2a** (*anti*-addition product) and **2b** (*syn*-addition product) is the δ H_d in both compounds: δ H_d in **2a** is 4.49 ppm and δ H_d in **2b** is 4.00 ppm. The chemical shift difference between two diastereomeric compounds is consistent with a broad range of products. This stereochemistry assignment is further corroborated through X-ray crystallographic analysis of **S37**, a structural analogue of **2a**.

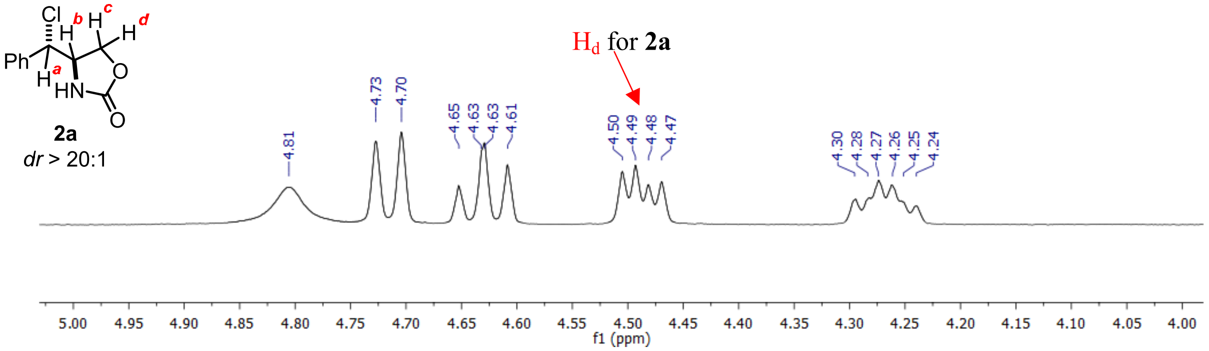

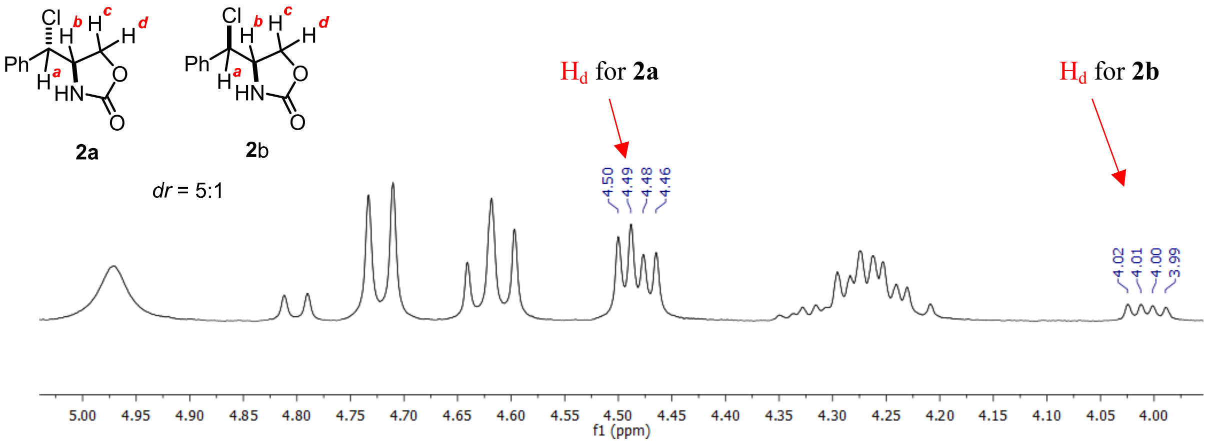

b. Synthesis and Characterization of New Substrates (S1–S8)

The substrates were synthesized by following a known procedure.[2] All new compounds have been characterized.

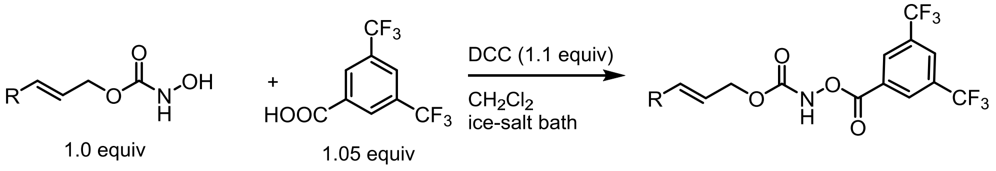

General procedures. To a flame-dried round bottom flask equipped with a magnetic stir bar were added hydroxyl carbamate (5.0 mmol, 1.0 equiv), 3,5-bis(trifluoromethyl)benzoic acid (5.25 mmol, 1.05 equiv) and anhydrous CH_2Cl_2 (50 mL). After stirring at -15 °C for 5 min, DCC (5.5 mmol, 1.1 equiv) in CH_2Cl_2 (10 mL) was added drop wise. The reaction was then stirred at the same temperature until all the hydroxyl carbamate was fully consumed monitored by TLC. The reaction solution was quenched by adding acetic acid (0.1 mL) and the mixture was filtered to remove *N, N′*-dicyclohexylurea (DCU). The filtrate was concentrated under reduced pressure, and then diethyl ether (20 mL) was added. The mixture was cooled in a refrigerator for 1 h and filtered again to remove additional DCU. The filtrate was concentrated *in vacuo* and the residue was purified through a gradient silica gel flash column chromatography (hexanes/EtOAc: from 20:1 to 7:1) or recrystallized directly with a hexanes/EtOAc mixture to afford the desired products (65–91% yield).

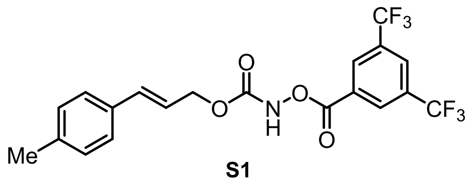

S1

***(E)*-3-(*p*-tolyl)allyl ((3,5-bis(trifluoromethyl)benzoyl)oxy)carbamate** (**S1**) : by following the general procedure, **S1** was obtained as a white solid (82% yield, m.p. 98–100 °C). ^{1}H NMR (400 MHz, $CDCl_3$): δ 8.55 (s, 2H), 8.43 (s, 1H), 8.14 (s, 1H), 7.29 (d, J = 8.1 Hz, 2H), 7.13 (d, J = 7.9 Hz, 2H), 6.68 (d, J = 15.8 Hz, 1H), 6.24 (dt, J = 15.8, 6.7 Hz, 1H), 4.89 (dd, J = 6.7, 0.9 Hz, 2H), 2.34 (s, 3H); ^{13}C NMR (100 MHz, $CDCl_3$): δ 163.4, 156.1, 138.4, 135.7, 133.0, 132.7 (q, J = 34.4 Hz), 130.2, 129.4, 129.0, 127.8–127.4(m), 126.7, 122.6 (q, J = 272.1 Hz), 120.7, 67.9, 21.2; IR ν_{max} (neat)/cm^{-1}: 3255 (m), 3085 (m), 2968 (m), 1764 (m), 1268 (s), 1134 (s), 680 (s); HRMS (ESI, m/z): calcd for $C_{20}H_{14}NO_4F_6^-$ (M - H^+), 446.0921, found 446.0924.

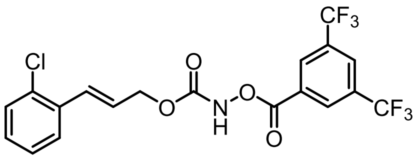

S2

(*E*)-3-(2-Chlorophenyl)allyl (3,5-bis(trifluoromethyl)benzoyl)oxycarbamate (**S2**): by following the general procedure, **S2** was obtained as a white solid (77% yield, m.p. 85–87 °C). ^{1}H NMR (400 MHz, $CDCl_3$): δ 8.55 (s, 2H), 8.49 (s, 1H), 8.15 (s, 1H), 7.54–7.51 (m, 1H), 7.36–7.34 (m, 1H), 7.25–7.19 (m, 2H), 7.09 (d, *J* = 15.9 Hz, 1H), 6.28 (dt, *J* = 15.8, 6.3 Hz, 1H), 4.93 (d, *J* = 6.3 Hz, 2H); ^{13}C NMR (100 MHz, $CDCl_3$): δ 163.4, 156.0, 134.0, 133.3, 132.7 (d, *J* = 34.4 Hz), 131.2, 130.0, 129.8, 129.3, 128.9, 127.6, 127.0, 126.9, 124.6, 122.6 (q, *J* = 273.0 Hz), 67.4; IR ν_{max} (neat)/cm^{-1}: 3265 (m), 3098 (w), 2940 (w), 1756 (m), 1280 (s), 1137 (s), 681 (s); HRMS (ESI, m/z): calcd for $C_{19}H_{11}NO_4F_6Cl^-$ (M - H^+), 466.0281, found 466.0284.

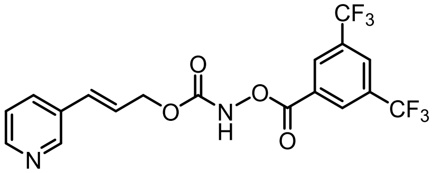

S3

(*E*)-3-(pyridin-3-yl)allyl (3,5-bis(trifluoromethyl)benzoyl)oxycarbamate (**S3**): by following the general procedure (purification through a gradient silica gel flash column chromatography with hexanes/Acetone: from 7:1 to 2.5:1), **S3** was obtained as a white solid (65% yield, m.p. 118–120 °C). ^{1}H NMR (400 MHz, $CDCl_3$) : δ 9.16 (s, 1H), 8.63 (s, 1H), 8.56 (s, 2H), 8.51 (d, *J* = 4.5 Hz, 1H), 8.15 (s, 1H), 7.72 (d, *J* = 7.9 Hz, 1H), 7.28 (dd, *J* = 4.8, 7.9 Hz, 1H), 6.70 (d, *J* = 16.0 Hz, 1H), 6.38 (dt, *J* = 16.0, 6.2 Hz, 1H), 4.92 (d, *J* = 6.1 Hz, 2H); ^{13}C NMR (100 MHz, $CDCl_3$) : δ 163.4, 156.0, 149.2, 148.3, 133.3, 132.7 (q, *J* = 34.3 Hz), 131.7, 131.2, 130.1, 129.0, 127.6, 124.6, 123.6, 122.6 (q, *J* = 273.2 Hz), 66.9; IR ν_{max} (neat)/cm^{-1}: 3093 (w), 2931 (w), 1750 (s), 1380 (m), 1277 (s), 1216 (s), 1137 (s), 706 (m), 682 (m); HRMS (ESI, m/z): calcd for $C_{18}H_{11}NO_4F_6^-$ (M - H^+), 433.0634, found 433.0623.

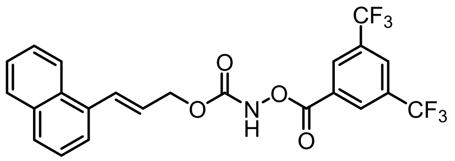

S4

(*E*)-3-(naphthalen-1-yl)allyl ((3,5-bis(trifluoromethyl)benzoyl)oxy)carbamate (**S4**): by following the general procedure, **S4** was obtained as a white solid (91% yield, m.p. 115–117 °C). ^{1}H NMR (400 MHz, $CDCl_3$): δ 8.55 (s, 2H), 8.46 (s, 1H), 8.15 (s, 1H), 8.08 (d, *J* = 7.8 Hz, 1H), 7.86 (d, *J* = 8.1 Hz, 1H), 7.81 (d, *J* = 8.1 Hz, 1H), 7.60 (d, *J* = 7.0 Hz, 1H), 7.56–7.42 (m, 4H), 6.34 (dt, *J* = 13.3, 6.4 Hz, 1H), 5.02 (d, *J* = 6.4 Hz, 2H); ^{13}C NMR (100 MHz, $CDCl_3$) δ 163.4, 156.1, 133.6, 133.6, 132.8, 132.7 (q, *J* = 34.3 Hz), 131.1, 130.1 (q, *J* = 4.2 Hz), 129.0, 128.7, 128.6, 127.7, 126.3, 125.9, 125.5, 125.0, 124.2, 123.6, 122.6 (q, *J* = 271.0 Hz), 67.8; IR v_{max} (neat)/cm^{-1}: 3674 (m), 2988 (m), 2901 (m), 1765 (m), 1755 (m), 1381 (m), 1278 (s), 1210 (m), 1141 (s), 904 (s), 710 (s); HRMS (ESI, *m/z*): calcd for $C_{23}H_{15}O_4NF_6ClNa^+$ (M + Na$^+$), 506.0911, found 506.0910.

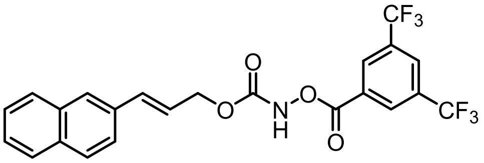

S5

(*E*)-3-(naphthalen-2-yl)allyl ((3,5-bis(trifluoromethyl)benzoyl)oxy)carbamate (**S5**): by following the general procedure, **S5** was obtained as a white solid (88% yield, m.p. 116–118 °C). ^{1}H NMR (400 MHz, $CDCl_3$): δ 8.56 (s, 2H), 8.39 (s, 1H), 8.15 (s, 1H), 7.85 – 7.78 (m, 3H), 7.76 (d, *J* = 1.6 Hz, 1H), 7.60 (dd, *J* = 8.6, 1.8 Hz, 1H), 7.52–7.44 (m, 2H), 6.88 (d, *J* = 15.9 Hz, 1H), 6.43 (dt, *J* = 15.9, 6.6 Hz, 1H), 4.96 (dd, *J* = 6.6, 1.3 Hz, 2H); ^{13}C NMR (100 MHz, $CDCl_3$): δ 163.4, 156.0, 135.7, 133.4, 133.3, 133.2, 132.7 (q, *J* = 34.4 Hz), 130.1 (q, *J* = 3.2 Hz), 129.0, 128.4, 128.1, 127.7, 127.2, 126.4, 126.3, 123.4, 122.6 (q, *J* = 272.2 Hz), 122.1, 67.8; IR v_{max} (neat)/cm^{-1}: 3688 (m), 2988 (s), 2901 (s), 1776 (m), 1755(m), 1380 (m), 1278 (s), 1213 (s), 1110 (m), 1076 (s), 1051 (s), 681 (m); HRMS (ESI, *m/z*): calcd for $C_{23}H_{15}O_4NF_6ClNa^+$ (M + Na$^+$), 506.0911, found 506.0914.

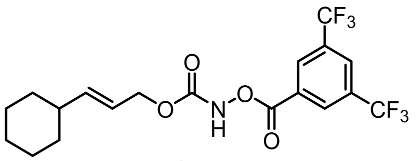

S6

(*E*)-3-Cyclohexylallyl (3,5-bis(trifluoromethyl)benzoyl)oxycarbamate (**S6**): by following the general procedure, **S6** was obtained as a white solid (83% yield, m.p. 60–62 °C). ^{1}H NMR (400 MHz, $CDCl_3$): δ 8.54 (s, 2H), 8.41 (s, 1H), 8.14 (s, 1H), 5.78 (dd, *J* = 15.5, 6.6 Hz, 1H), 5.60–5.46 (m, 1H), 4.67 (d, *J* = 6.6 Hz, 2H), 2.08–1.91 (m, 1H), 1.81–1.55 (m, 4H), 1.37–0.98 (m, 6H); ^{13}C NMR (100 MHz, $CDCl_3$): δ 163.4, 156.1, 143.8, 132.7 (q, *J* = 34.3 Hz), 130.0, 129.0, 127.5, 122.6 (q, *J* = 273.0 Hz), 120.2, 68.2, 40.3, 32.4, 26.0, 25.9; IR ν_{max} (neat)/cm^{-1}: 3070 (w), 2971 (w), 1720 (m), 1282 (m), 1265 (s), 1211 (m), 1145 (m), 896 (m), 728 (s), 703 (s); HRMS (ESI, m/z): calcd for $C_{19}H_{18}NO_4F_6^-$ (M - H^+), 438.1140, found 438.1126.

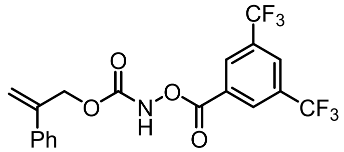

S7

2-Phenylallyl (3,5-bis(trifluoromethyl)benzoyl)oxycarbamate (**S7**): by following the general procedure, **S7** was obtained as a white solid (75% yield, m.p. 70–72 °C). ^{1}H NMR (400 MHz, $CDCl_3$): δ 8.47 (s, 2H), 8.16 (s, 1H), 7.40 (d, *J* = 7.2 Hz, 2H), 7.32–7.25 (m, 3H), 5.59 (s, 1H), 5.43 (s, 1H), 5.16 (s, 2H); ^{13}C NMR (100 MHz, $CDCl_3$): δ 163.3, 156.1, 141.6, 137.3, 132.5 (q, *J* = 34.4 Hz), 130.0, 128.9, 128.4, 128.1, 127.5, 125.9, 122.6 (q, *J* = 273.1 Hz), 116.3, 68.2; IR ν_{max} (neat)/cm^{-1}: 3320 (m), 3060 (w), 1758 (m), 1281 (s), 1185 (s), 737 (s); HRMS (ESI, m/z): calcd for $C_{19}H_{12}NO_4F_6^-$ (M - H^+), 432.0671, found 432.0660.

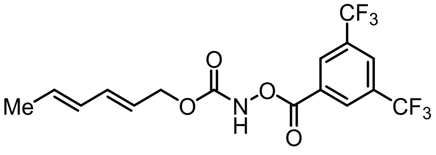

S8

(2*E*,4*E*)-hexa-2,4-dien-1-yl (3,5-bis(trifluoromethyl)benzoyl)oxycarbamate (**S8**): by following the general procedure, **S8** was obtained as a white solid (90% yield, m.p. 84–86 °C).

^{1}H NMR (400 MHz, $CDCl_3$): δ 8.54 (s, 2H), 8.38 (s, 1H), 8.14 (s, 1H), 6.30 (dd, *J* = 15.1, 10.6 Hz, 1H), 6.12–6.00 (m, 1H), , 5.78 (dq, *J* = 13.7, 6.5 Hz, 1H), 5.63 (dt, *J* = 14.4, 6.8 Hz, 1H), 4.73 (d, *J* = 6.7 Hz, 2H), 1.77 (d, *J* = 6.6 Hz, 3H); ^{13}C NMR (100 MHz, $CDCl_3$): δ 163.4, 156.0, 136.3, 132.6 (q, *J* = 34.3 Hz), 132.4, 127.6, 122.5 (q, *J* = 271.3 Hz), 122.2, 67.7, 18.2; IR ν_{max} (neat)/cm^{-1}: 3261 (w), 3022 (w), 2918 (w), 2856 (w), 1753 (m), 1281 (s); HRMS (ESI, m/z): calcd for $C_{16}H_{12}NO_4F_6^-$ (M - H^+), 396.0671, found 396.0677.

c. General Procedure for the Iron-Catalyzed Diastereoselective Olefin Aminochlorination and Product Characterization

General procedure. To a flame-dried sealable 2-dram vial (vial **A**) equipped with a magnetic stir bar were added $Fe(NTf_2)_2$ (12.3 mg, 0.02 mmol, 10 mol %) and 1,10-phenanthroline (7.2 mg, 0.04 mmol, 20 mol %). After the vial was evacuated and backfilled with N_2 for three times, anhydrous CH_2Cl_2 (1.0 mL) was added and the mixture was stirred at room temperature for 20 min. During this time, the substrate (0.2 mmol) and anhydrous TBAC (139 mg, 0.5 mmol) were dissolved in CH_2Cl_2 (4.0 mL) in a second flame-dried 3-dram vial (vial **B**) with a magnetic stir bar under N_2 atmosphere. Both vials were degassed by brief evacuation and back filling with N_2 twice. The vial **B** was cooled down to 0 °C, and the solution in vial **A** was added to vial **B** drop wise via a syringe. The resulting solution was stirred at the same temperature until all the starting material was fully consumed monitored by TLC. The reaction was quenched by 2 mL saturated $NaHCO_3$ solution. After being extracted with CH_2Cl_2 (1.5 mL × 3), the combined organic phase was concentrated and the residue was purified through a gradient silica gel flash column chromatography (hexanes/acetone: from 15:1 to 4:1) to afford the aminochlorination product. The *dr* was determined by ^{1}H NMR analysis of the crude reaction mixture.

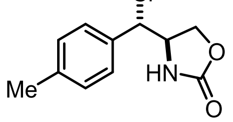

S9

4-(Chloro(*p*-tolyl)methyl)oxazolidin-2-one (**S9**) : by following the general procedure and carrying out reaction at -15 °C, **S9** was obtained as a white solid (39 mg, 86% yield, *dr* >20:1, m.p. 126–129 °C). ^{1}H NMR (400 MHz, $CDCl_3$): δ 7.27 (d, *J* = 8.0 Hz, 2H), 7.22 (d, *J* = 8.1 Hz,

2H), 4.84 (s, 1H), 4.69 (d, J = 9.2 Hz, 1H), 4.62 (dd, J = 9.3, 8.3 Hz, 1H), 4.47 (dd, J = 9.4, 4.7 Hz, 1H), 4.25 (td, J = 8.5, 5.0 Hz, 1H), 2.37 (s, 3H); ^{13}C NMR (100 MHz, $CDCl_3$): δ 158.0, 139.9, 133.6, 130.0, 127.6, 68.7, 63.4, 58.3, 21.2; IR ν_{max} (neat)/cm^{-1}: 3272 (m), 3154 (w), 3037 (w), 2918 (w), 2340 (w), 1749 (s), 1404 (m), 1280 (m), 1239 (m), 1026 (m), 766 (m); HRMS (ESI, m/z): calcd for $C_{11}H_{13}NO_2Cl^+$ (M + H$^+$), 226.0635, found 226.0640.

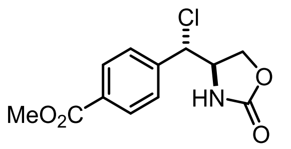

S10

Methyl 4-(chloro(2-oxooxazolidin-4-yl)methyl)benzoate (**S10**): by following the general procedure, **S10** and its diastereomer were obtained as a white solid (38 mg, 70% yield, *dr*: 7:1, m.p. 136–139 °C). **S10**: ^{1}H NMR (400 MHz, $CDCl_3$): δ 8.06 (d, J = 8.4 Hz, 2H), 7.47 (d, J = 8.3 Hz, 2H), 5.58 (s, 1H), 4.79 (d, J = 8.6 Hz, 1H), 4.58 (dd, J = 9.5, 8.3 Hz, 1H), 4.45 (dd, J = 9.5, 4.7 Hz, 1H), 4.27 (td, J = 8.4, 4.7 Hz, 1H), 3.92 (s, 3H); ^{13}C NMR (100 MHz, $CDCl_3$): δ 166.2, 158.4, 141.2, 131.3, 130.4, 127.8, 68.2, 62.8, 58.2, 52.4; its *syn*-diastereomer: ^{1}H NMR (400 MHz, $CDCl_3$): δ 8.08 (d, J = 8.4 Hz, 2H), 7.46 (d, J = 8.3 Hz, 2H), 5.62 (s, 1H), 4.85 (d, J = 8.3 Hz, 1H), 4.33 (td, J = 8.4, 5.2 Hz, 1H), 4.27 (dd, J = 9.1 Hz, 1H), 4.04 (dd, J = 9.2, 4.6 Hz, 1H), 3.96 (s, 3H); ^{13}C NMR (100 MHz, $CDCl_3$): δ 166.1, 157.9, 141.2, 131.4, 130.5, 127.7, 68.4, 66.8, 64.3, 62.7, 58.5, 58.1, 52.4; IR ν_{max} (neat)/cm^{-1}: 3229 (m), 3132(w), 2957 (w), 2919 (w), 2849 (w), 1731 (s), 1714 (s), 1434 (m), 1278 (s), 1243 (s), 1110 (s), 1033 (s), 1018 (s), 770 (m); HRMS (ESI, m/z): calcd for $C_{12}H_{13}NO_4Cl^+$ (M + H$^+$), 270.0533, found 270.0535.

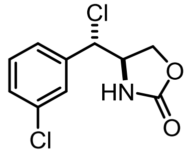

S11

4-(Chloro(3-chlorophenyl)methyl)oxazolidin-2-one (**S11**) : by following the general procedure, **S11** was obtained as a white solid (33 mg, 67% yield, *dr*: 10:1, m.p. 107–109 °C). ^{1}H NMR (400 MHz, $CDCl_3$): δ 7.38–7.32 (m, 3H), 7.24 (s, 1H), 5.10 (s, 1H), 4.66 (d, J = 9.0 Hz, 1H), 4.59 (t, J = 8.8 Hz, 1H), 4.44 (dd, J = 9.7, 4.8 Hz, 1H), 4.21 (td, J = 8.8, 5.1 Hz, 1H); ^{13}C NMR (100 MHz,

CDCl$_3$): δ 158.0, 138.5, 135.3, 130.6, 129.9, 127.8, 125.9, 68.4, 62.5, 58.2; IR ν_{max} (neat)/cm^{-1}: 3271 (m), 2918 (w), 1749 (s), 1233 (w), 1027 (m), 700 (m); HRMS (ESI, m/z): calcd for $C_{10}H_{10}NO_2Cl_2^+$ (M + H$^+$), 246.0089, found 246.0081.

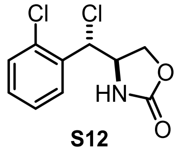

S12

4-(Chloro(2-chlorophenyl)methyl)oxazolidin-2-one (**S12**) : by following the general procedure, **S12** was obtained as a white solid (37 mg, 76% yield, *dr*: 10:1, m.p. 93–95 °C). ^{1}H NMR (400 MHz, CDCl$_3$): δ 7.56 (dd, *J* = 7.5, 2.0 Hz, 1H), 7.43 (dd, *J* = 7.6, 1.7 Hz, 1H), 7.40–7.30 (m, 2H), 5.48 (s, 1H), 5.42 (d, *J* = 8.0 Hz, 1H), 4.55 (dd, *J* = 9.3, 8.2 Hz, 1H), 4.47 (dd, *J* = 9.3, 4.4 Hz, 1H), 4.39 (td, *J* = 8.1, 4.5 Hz, 1H); ^{13}C NMR (100 MHz, CDCl$_3$): δ 158.4, 134.0, 133.7, 130.6, 130.2, 129.0, 127.9, 67.9, 58.7, 57.4; IR ν_{max} (neat)/cm^{-1}: 3255 (m), 3020 (w), 2982 (w), 2917 (w), 1742 (s), 1475 (m), 1402 (m), 1234 (m), 1023 (m), 736 (m), 698 (m); HRMS (ESI, m/z): calcd for $C_{10}H_{10}NO_2Cl_2^+$ (M + H$^+$), 246.0089, found 246.0081.

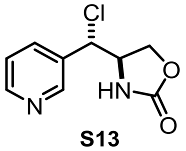

S13

4-(Chloro(pyridin-3-yl)methyl)oxazolidin-2-one (**S13**) : by following the general procedure, **S13** was obtained as a white solid (32 mg, 76% yield, *dr* : 12:1, m.p. 121–124 °C). ^{1}H NMR (400 MHz, CDCl$_3$) δ 8.62 (d, *J* = 2.2 Hz, 1H), 8.59 (dd, *J* = 4.8, 1.4 Hz, 1H), 7.77 (dt, *J* = 7.9, 1.9 Hz, 1H), 7.37 (dd, *J* = 7.9, 4.8 Hz, 1H), 5.64 (s, 1H), 4.77 (d, *J* = 8.8 Hz, 1H), 4.63 (dd, *J* = 9.4, 8.4 Hz, 1H), 4.47 (dd, *J* = 9.5, 4.6 Hz, 1H), 4.30 (tdd, *J* = 8.6, 4.6, 1.1 Hz, 1H); ^{13}C NMR (100 MHz, CDCl$_3$): δ 158.2, 150.8, 149.0, 135.4, 132.6, 124.1, 68.3, 60.8, 58.2; IR ν_{max} (neat)/cm^{-1}: 3238 (m), 3133 (w), 2957 (w), 2924 (w), 2854 (w), 1742 (s), 1428 (m), 1406 (m), 1234 (m), 1026 (m), 711 (m); HRMS (ESI, *m/z*): calcd for $C_9H_{10}N_2O_2Cl^+$ (M + H$^+$), 213.0431, found 213.0437.

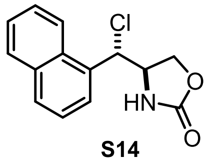

S14

4-(Chloro(naphthalen-1-yl)methyl)oxazolidin-2-one (S14) : by following the general procedure and carrying out reaction at -15 °C, **S14** was obtained as a white solid (32 mg, 61% yield, *dr* >20:1, m.p. 134–136 °C). ^{1}H NMR (400 MHz, CD_3CN): δ 8.24 (d, *J* = 8.5 Hz, 1H), 8.00 (t, *J* = 7.6 Hz, 2H), 7.75 (d, *J* = 7.2 Hz, 1H), 7.71–7.64 (m, 1H), 7.64–7.57 (m, 2H), 5.97 (s, 1H), 5.88 (d, *J* = 7.9 Hz, 1H), 4.76–4.68 (m, 1H), 4.61 (t, *J* = 8.6 Hz, 1H), 4.55 (dd, *J* = 9.1, 5.2 Hz, 1H); ^{13}C NMR (100 MHz, CD_3CN): δ 158.3, 134.0, 133.0, 130.9, 129.8, 129.0, 127.0, 126.3, 126.0, 125.6, 122.8, 67.7, 56.5; IR ν_{max} (neat)/cm^{-1}: 3250 (m), 2923 (w), 1756(s), 1712 (s), 1485 (m), 1416 (m), 1230(s), 1027(s), 760 (s); HRMS (ESI, *m/z*): calcd for $C_{14}H_{13}O_2NCl^+$ (M + H^+), 262.0629, found 262.0623.

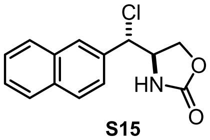

S15

4-(Chloro(naphthalen-2-yl)methyl)oxazolidin-2-one (**S15**): by following the general procedure and carrying out reaction at -15 °C, **S15** was obtained as a white solid (31 mg, 59% yield, *dr* > 20:1, m.p. 136–139 °C). ^{1}H NMR (400 MHz, $CDCl_3$): δ 7.92 (d, *J* = 8.6 Hz, 1H), 7.86 (dt, *J* = 6.4, 3.3 Hz, 2H), 7.83 (d, *J* = 1.8 Hz, 1H), 7.56 (dt, *J* = 6.2, 3.4 Hz, 2H), 7.50 (dd, *J* = 8.5, 1.9 Hz, 1H), 4.88 (d, *J* = 9.3 Hz, 1H), 4.85 (s, 1H), 4.66 (dd, *J* = 9.4, 8.1 Hz, 1H), 4.54 (dd, *J* = 9.4, 4.7 Hz, 1H), 4.37 (td, *J* = 8.8, 5.0 Hz, 1H); ^{13}C NMR (100 MHz, $CDCl_3$): δ 157.9, 133.7, 133.6, 132.9, 129.7, 128.1, 127.8, 127.7, 127.3, 127.2, 124.0, 68.6, 63.8, 58.2; IR ν_{max} (neat)/cm^{-1}: 3253 (m), 3144(w), 2918 (w), 1744 (s), 1709(s), 1479 (m), 1409 (m), 1244(s), 1017(s), 761 (s); HRMS (ESI, *m/z*): calcd for $C_{14}H_{13}O_2NClH^+$ (M + H^+), 262.0629, found 262.0624.

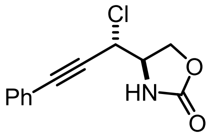

S16

4-(1-Chloro-3-phenylprop-2-yn-1-yl)oxazolidin-2-one (**S16**): by following the general procedure and carrying out reaction at -15 °C, **S16** and its *syn*-diastereomer were obtained as a white solid (44 mg, 93% yield, *dr*: 7:1). **S16**: ^{1}H NMR (400 MHz, $CDCl_3$): δ 7.47–7.45 (m, 2H), 7.39–7.32 (m, 3H), 5.83 (s, 1H), 4.76 (d, J = 6.4 Hz,1H), 4.60 (dd, J = 9.5, 8.5 Hz, 1H), 4.52 (dd, J = 9.6, 4.1 Hz, 1H), 4.23 (ddd, J = 8.3, 6.4, 4.1 Hz, 1H); ^{13}C NMR (100 MHz, $CDCl_3$): δ 158.3, 132.0, 129.6, 128.5, 120.8, 88.9, 81.8, 67.1, 57.6, 51.0; its *syn*-diastereomer: ^{1}H NMR (400 MHz, $CDCl_3$): δ 7.50–7.43 (m, 2H), 7.40–7.31 (m, 3H), 5.79 (s, 1H), 4.73 (d, J = 6.2 Hz, 1H), 4.55–4.61 (m, 1H), 4.45 (dd, J = 9.5, 3.9 Hz, 1H), 4.30–4.25 (m, 1H); ^{13}C NMR (100 MHz, $CDCl_3$): δ 158.3, 132.0, 129.6, 128.5, 120.8, 88.9, 81.8, 67.1, 57.5, 51.0; IR ν_{max} (neat)/cm^{-1}: 3270 (m), 3021 (w), 2980 (w), 2226 (w), 1754 (s), 1233 (s), 1039 (m), 934 (m), 758 (m); HRMS (ESI, m/z): calcd for $C_{12}H_{11}NO_2Cl^+$ (M + H$^+$), 236.0478, found 236.0487.

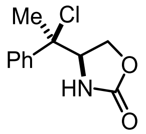

S17a

4-(1-Chloro-1-phenylethyl)oxazolidin-2-one (**S17a**) : by following the general procedure, **S17a** was obtained as a white solid (22 mg, 50% yield, *dr* >20:1, m.p. 75–77 °C). Its relative chemistry was determined by comparison of the ^{1}H NMR data with the literature data.[2] ^{1}H NMR (400 MHz, $CDCl_3$) δ 7.53 (d, J = 7.9 Hz, 2H), 7.47–7.31 (m, 3H), 5.18 (s, 1H), 4.46 (dd, J = 6.3, 1.9 Hz, 2H), 4.33 (t, J = 6.5 Hz, 1H), 1.98 (d, J = 1.9 Hz, 3H); ^{13}C NMR (100 MHz, $CDCl_3$) δ 158.7, 140.5, 128.9, 128.8, 126.6, 72.3, 66.8, 62.2, 24.7; IR ν_{max} (neat)/cm^{-1}: 3260 (m), 3120 (w), 2996 (w), 2915 (w), 1730(s), 1035 (w), 1232 (m), 1040 (m), 709 (m); HRMS (ESI, *m/z*): calcd for $C_{11}H_{13}NO_2Cl^+$ (M + H$^+$), 226.0635, found 226.0640.

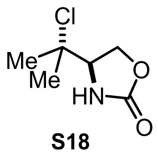

S18

4-(2-Chloropropan-2-yl)oxazolidin-2-one (**S18**) : by following the general procedure, **S18** was obtained as a white solid (25 mg, 76% yield, m.p. 53–56 °C). ^{1}H NMR (400 MHz, $CDCl_3$): δ

6.58 (s, 1H), 4.47 (t, J = 9.2 Hz, 1H), 4.35 (dd, J = 9.5, 4.6 Hz, 1H), 3.98 (dd, J = 8.9, 4.6 Hz, 1H), 1.56 (s, 6H); ^{13}C NMR (100 MHz, $CDCl_3$): δ 159.6, 69.4, 66.7, 61.8, 27.8, 26.8; IR ν_{max} (neat)/cm^{-1}: 2923 (s), 2860(s), 1763 (s), 1481 (w), 1340 (m), 1220(s), 1049(s), 809 (s); HRMS (ESI, *m/z*): calcd for $C_6H_{11}NO_2Cl^+$ (M + H^+), 164.0422, found 164.0417.

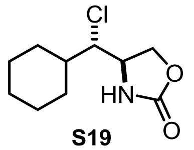

S19

(±)-4-(Chloro(cyclohexyl)methyl)oxazolidin-2-one (S19) : by following the general procedure and carrying out the reaction for 5 h, **S19** was obtained as a white solid (30 mg, 69% yield, *dr* >20:1, m.p. 115–118 °C). ^{1}H NMR (400 MHz, $CDCl_3$): δ 7.16 (s, 1H), 4.52 (t, J = 8.8 Hz, 1H), 4.34 (dd, J = 9.1, 5.4 Hz, 1H), 4.11 (td, J = 8.4, 5.4 Hz, 1H), 3.77 (dd, J = 8.4, 3.4 Hz, 1H), 1.84–1.62 (m, 6H), 1.51–1.01 (m, 5H); ^{13}C NMR (100 MHz, $CDCl_3$): δ 160.1, 69.7, 68.9, 54.5, 39.8, 30.7, 26.2, 26.0, 25.9, 25.5; IR ν_{max} (neat)/cm^{-1}: 2925 (s), 2851(s), 1759 (s), 1482 (w), 1375 (m), 1227(s), 1149(s), 1035 (s), 820 (s); HRMS (ESI, *m/z*): calcd for $C_{10}H_{17}O_2NCl^+$ (M + H^+), 218.0942, found 218.0937.

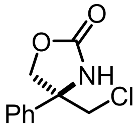

S20

4-(Chloromethyl)-4-phenyloxazolidin-2-one (**S20**): by following the general procedure and carrying out the reaction for 12 h, **S20** was obtained as a white solid (32 mg, 77% yield, m.p. 109–112 °C). ^{1}H NMR (400 MHz, $CDCl_3$): δ 7.46–7.42 (m, 2H), 7.39–7.36 (m, 1H), 7.32–7.30 (m, 2H), 6.50 (s, 1H), 4.66 (d, J = 8.9 Hz, 1H), 4.44 (d, J = 8.9 Hz, 1H), 3.93 (d, J = 11.7 Hz, 1H), 3.88 (d, J = 11.7 Hz, 1H); ^{13}C NMR (100 MHz, $CDCl_3$): δ 158.9, 139.6, 129.3, 128.7, 124.9, 74.1, 64.1, 51.3; IR ν_{max} (neat)/cm^{-1}: 3266 (m), 2912 (w), 2853 (w), 1753 (s), 1396 (w), 1094 (s), 1052 (w); HRMS (ESI, *m/z*): calcd for $C_{10}H_{11}NO_2Cl^+$ (M + H^+), 212.0478, found 212.0479.

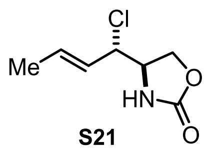

S21

4-(*E*-1-chlorobut-2-en-1-yl)oxazolidin-2-one (**S21**) : by following the general procedure, **S21** and its *syn*-diastereomer were obtained as white solids (31 mg, 88% yield, *dr*: 1.5:1). **S21**: ^{1}H NMR (400 MHz, $CDCl_3$): δ 6.15 (s, 1H), 5.88 (dd, *J* = 12.5, 6.0 Hz, 1H), 5.50–5.39 (m, 1H), 4.50 (t, *J* = 8.9 Hz, 1H), 4.32 (dd, *J* = 9.2, 4.9 Hz, 1H), 4.24 (dd, *J* = 17.6, 9.4 Hz, 1H), 4.04–3.95 (m, 1H), 1.77 (d, *J* = 5.1 Hz, 3H); ^{13}C NMR (100 MHz, $CDCl_3$): δ 159.1, 133.9, 126.4, 67.8, 63.5, 57.1, 17.8; its *syn*-diastereomer: ^{1}H NMR (400 MHz, $CDCl_3$): δ 6.26 (s, 1H), 5.93 (dd, *J* = 11.7, 6.4 Hz, 1H), 5.50–5.39 (m, 1H), 4.41 (t, *J* = 9.0 Hz, 1H), 4.21–4.16 (m, 1H), 4.04–3.95 (m, 1H), 1.75–1.73 (d, *J* = 5.0 Hz, 3H); ^{13}C NMR (100 MHz, $CDCl_3$): δ 158.9, 133.9, 125.7, 66.9, 64.3, 57.3, 17.8; IR ν_{max} (neat)/cm^{-1}: 3238 (m), 3135 (w), 2972 (w), 2925 (m), 2852 (w), 1760(s), 1667 (m), 1403 (m), 1234 (s), 1004 (m), 966 (s), 1027(s), 742 (s); HRMS (ESI, m/z): calcd for $C_7H_{10}NO_2NaCl^+$ (M + Na^+), 198.0298, found 198.0304.

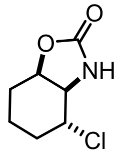

S22

4-Chlorohexahydrobenzo[*d*]oxazol-2(3H)-one (**S22**): by following the general procedure, **S22** was obtained as a white solid (22 mg, 64% yield, *dr* >20:1, m.p. 112–114 °C). ^{1}H NMR (400 MHz, $CDCl_3$): δ 5.63 (s, 1H), 4.71 (dt, *J* = 6.3, 3.2 Hz, 1H), 3.80 (ddd, *J* = 12.2, 8.6, 4.3 Hz, 1H), 3.62 (dd, *J* = 8.5, 6.1 Hz, 1H), 2.32-2.09 (m, 2H), 1.76–1.52 (m, 4H); ^{13}C NMR (100 MHz, $CDCl_3$): δ 159.1, 77.0, 62.2, 60.6, 31.8, 25.9, 19.7; IR ν_{max} (neat)/cm^{-1}: 3272 (m), 2948 (w), 2885 (w), 1751 (s), 1201 (w); HRMS (ESI, *m/z*): calcd for $C_7H_{11}NO_2Cl^+$ (M + H^+), 176.0478, found 176.0480.

C. Catalyst Discovery and Procedures for the Iron-Catalyzed Asymmetric Olefin Aminochlorination Reaction

a. Catalyst Discovery for the Iron-Catalyzed Asymmetric Olefin Aminochlorination

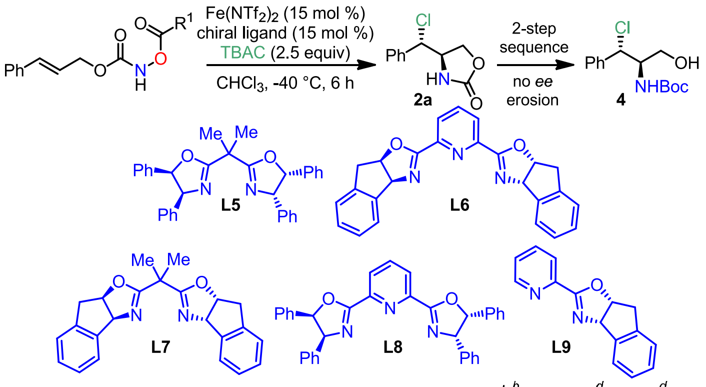

entry[a]	R	ligand	conversion[b]	yield[c]	dr[b] (*anti*:*syn*)	ee[d] (*anti*)	ee[d] (*syn*)
1	3,5-$(CF_3)_2$-Ph	**L5**	>95%	53%	9.9:1	84%	<5%
2	3,5-$(CF_3)_2$-Ph	**L6**	>95%	68%	0.48:1	24%	79%
3	3,5-$(CF_3)_2$-Ph	**L7**	88%	61%	1.74:1	<5%	<5%
4	3,5-$(CF_3)_2$-Ph	**L8**	>95%	32%	2.45:1	47%	30%
5	3,5-$(CF_3)_2$-Ph	**L9**	>95%	82%	0.51:1	8%	24%
6[e]	3,5-$(CF_3)_2$-Ph	**L5**	>95%	51%	10.5:1	90%	<5%
7[e]	CH_3	**L5**	>95%	42%	1.1:1	97%	<5%
8[e]	CH_2Cl	**L5**	>95%	67%	9.6:1	89%	<5%

Table S2. Catalyst discovery for the iron-catalyzed asymmetric olefin aminochlorination reaction

Chiral ligands **L5–L9** were synthesized by following literature procedures.[2, 5-7]

Procedure for the Catalyst Discovery. To a flame-dried sealable 2-dram vial (vial **A**) equipped with a magnetic stir bar were added $Fe(NTf_2)_2$ (9.2 mg, 0.015 mmol) and a chiral ligand (0.015 mmol). After the vial was evacuated and backfilled with N_2 for three times, re-distilled and anhydrous $CHCl_3$ (1.0 mL) was added and the mixture was stirred at room temperature for 20 min. During this time, substrate (0.1 mmol) and anhydrous TBAC (69.5 mg, 0.25 mmol) were dissolved in $CHCl_3$ (3.0 mL, re-distilled and anhydrous) in a second flame-dried 2-dram vial (vial **B**) with a magnetic stir bar and freshly activated 4 Å molecular sieves under N_2 atmosphere. Both vials were degassed by brief evacuation and back filling with N_2 twice. The vial **B** was cooled down to -60 °C, and the solution in vial **A** was added to vial **B** drop wise via a syringe. The resulting solution was stirred at the same temperature for 12 h and quenched with 1 mL saturated $NaHCO_3$ solution. The reaction mixture was extracted with CH_2Cl_2 (1.5 mL × 3), and the combined organic phase was concentrated *in vacuo*. The residue was purified through a gradient silica gel flash column chromatography (hexanes/acetone: from 15:1 to 4:1) to afford

the aminochlorination product as a white solid. The *dr* was determined by ^{1}H NMR analysis and the *ee* was measured by chiral HPLC analysis. The results are listed in the table (Table S2). The absolute stereochemistry was determined by X-ray crystallographic analysis of a structural analog of **2a** with heavy atoms.

The racemic products with low *dr* (for HPLC assay purposes) were obtained by following the general procedure of the iron-catalyzed diastereoselective olefin aminochlorination under the ligand-free condition (Table S1, entry 1).

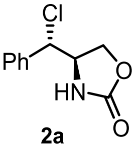

2a

(*S*)-4-((*R*)-chloro(phenyl)methyl)oxazolidin-2-one (**2a**): by following the general procedure under the condition described in entry 8, the product **2a** obtained as a white solid (15 mg, 67% yield, *dr*: 9.6:1). $[\alpha]_D^{20}$ = + 56.4° (*c* 1.0, CH_2Cl_2). The *ee* was determined by Chiral HPLC analysis (Chiral OD-H column, 10% EtOH in hexanes, flow rate = 1.0 mL/min, UV detection at 205 nm). The *anti*-diastereomer (**2a**): t_r (minor) = 19.5 min, t_r (major) = 22.4 min, 89% *ee*; the *syn*-diastereomer (**2b**): t_r (minor) = 23.8 min, t_r (major) = 30.9 min, <5% *ee*.

Racemic sample

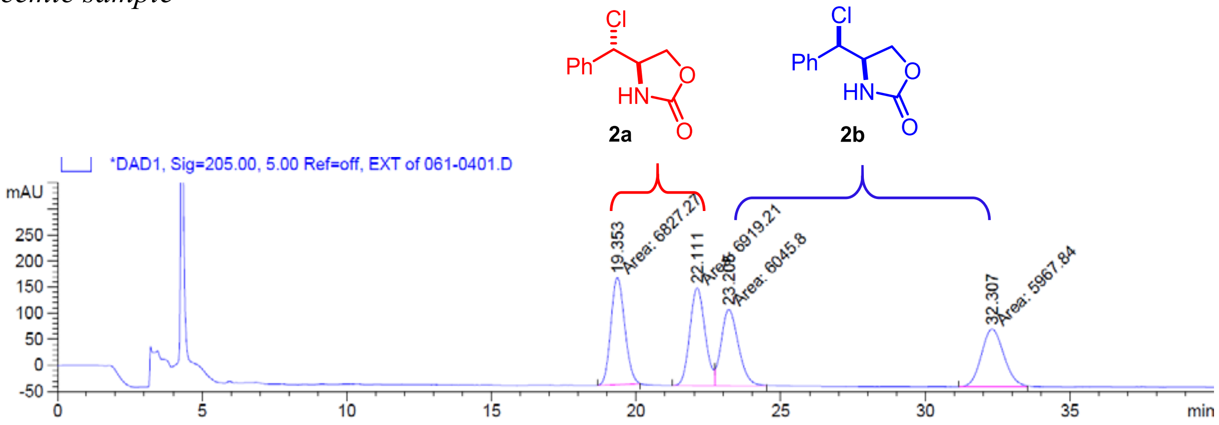

Peak #	RetTime [min]	Type	Width [min]	Area [mAU*s]	Height [mAU]	Area %
1	19.353	MM	0.5534	6827.27100	205.61459	26.5033
2	22.111	MF	0.6171	6919.21436	186.88011	26.8602
3	23.205	FM	0.6898	6045.79639	146.08553	23.4696
4	32.307	MF	0.9027	5967.84473	110.18359	23.1670

Enantio-enriched sample (*anti*-diastereomer, **2a**, 89% *ee*)

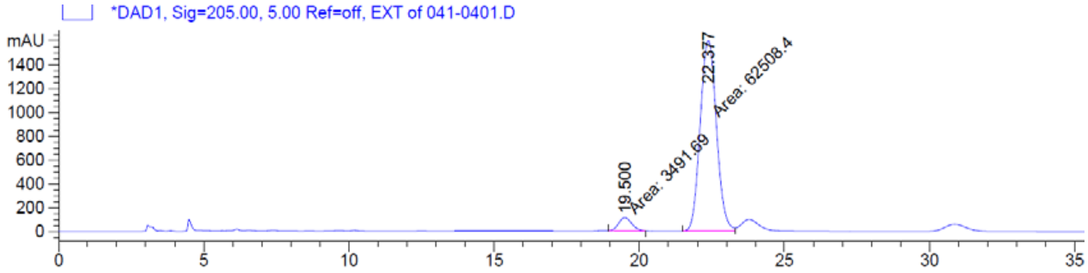

Peak #	RetTime [min]	Type	Width [min]	Area [mAU*s]	Height [mAU]	Area %
1	19.500	MM	0.5234	3491.69165	111.19152	5.2904
2	22.377	FM	0.6517	6.25084e4	1598.60620	94.7096

Enantio-enriched sample (*syn*-diastereomer, **2b**, <5% *ee*)

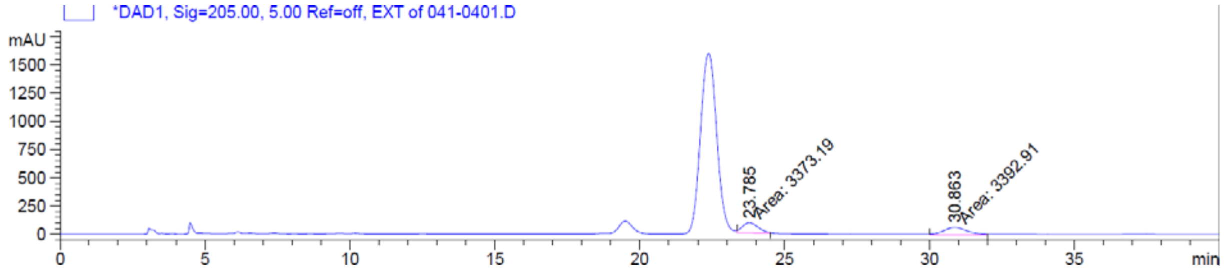

Peak #	RetTime [min]	Type	Width [min]	Area [mAU*s]	Height [mAU]	Area %
1	23.785	MM	0.6270	3373.19238	89.67115	49.8543
2	30.863	MM	0.8864	3392.90967	63.79350	50.1457

Totals :	6766.10205	153.46465

b. Synthesis and Characterization of New Substrates (S23–S34)

Chloroacetoxyl carbamates were synthesized by following a known procedure.[1]

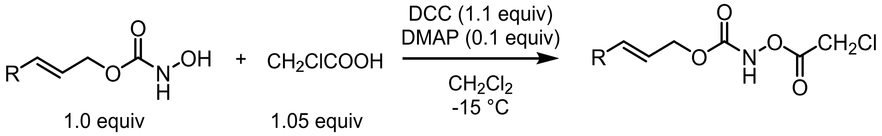

General Procedure. To a flame-dried round bottom flask equipped with a magnetic stir bar were added a hydroxyl carbamate (5.0 mmol, 1.0 equiv), chloroacetic acid (5.25 mmol, 1.05 equiv) and anhydrous CH_2Cl_2 (50 mL). After stirring at -15 °C for 2 min, DCC (5.5 mmol, 1.1 equiv) and DMAP (0.5 mmol, 0.1 equiv) in CH_2Cl_2 (10 mL) was added drop wise. The reaction mixture was stirred at the same temperature until all the hydroxyl carbamate was fully consumed monitored by TLC. The reaction mixture was filtered to remove *N, N'*-dicyclohexylurea (DCU). The filtrate was concentrated *in vacuo*, and then diethyl ether (20 mL) was added. The mixture was cooled in a refrigerator for 0.5 h and filtered again to remove the additional DCU. The filtrate was concentrated *in vacuo* and the residue was purified through a rapid gradient silica gel flash column chromatography (hexanes/acetone: from 10:1 to 3:1) and further recrystallization from a mixture of hexanes/EtOAc to afford the desired products (59–86% yield). *Note: Most chloroacetoxyl carbamates must be purified rapidly by flash columns (flash rate: ca. 50 mL/min) because they tend to undergo hydrolysis upon exposure to silica gel for an extended period of time.*

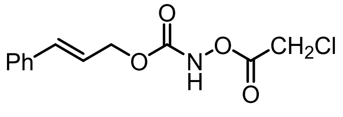

S23

***E*-Cinnamyl (2-chloroacetoxy)carbamate** (**S23**): by following the general procedure, **S23** was obtained as a white solid (79% yield, m.p. 50–51 °C). ^{1}H NMR (400 MHz, $CDCl_3$): δ 8.48 (s, 1H), 7.40–7.38 (m, 2H), 7.35–7.28 (m, 3H), 6.68 (d, J = 15.9 Hz, 1H), 6.26 (dt, J = 15.9, 6.5 Hz, 1H), 4.85 (dd, J = 6.5, 1.1 Hz, 2H), 4.21 (s, 2H); ^{13}C NMR (100 MHz, $CDCl_3$): δ 166.8, 156.1, 135.9, 135.4, 128.7, 128.4, 126.8, 121.9, 67.6, 38.6; IR ν_{max} (neat)/cm^{-1}: 3257 (m), 3027 (w),

2955 (w), 1793 (m), 1733 (s), 1448 (s), 1244 (m), 1214 (m), 1050 (s), 1025 (m), 804 (m), 770 (s), 698 (s); HRMS (ESI, *m/z*): calcd for $C_{12}H_{12}O_4NClNa^+$ (M + Na^+), 292.0347, found 292.0339.

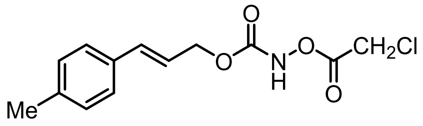

S24

(*E*)-3-(*p*-tolyl)allyl (2-chloroacetoxy)carbamate (**S24**): by following the general procedure, **S24** was obtained as a white solid (82% yield, m.p. 72–74 °C). ^{1}H NMR (400 MHz, $CDCl_3$): δ 8.19 (s, 1H), 7.29 (d, *J* = 8.0 Hz, 2H), 7.14 (d, *J* = 7.7 Hz, 2H), 6.66 (d, *J* = 15.8 Hz, 1H), 6.22 (dt, *J* = 15.8, 6.6 Hz, 1H), 4.84 (d, *J* = 6.6 Hz, 2H), 4.22 (s, 2H), 2.34 (s, 3H); ^{13}C NMR (100 MHz, $CDCl_3$): δ 166.7, 155.9, 138.4, 135.6, 133.0, 129.4, 126.7, 120.7, 67.8, 38.5, 21.3; IR ν_{max} (neat)/cm^{-1}: 3212 (m), 3007 (w), 2958 (w), 1807 (m), 1741 (m), 1716 (s), 1481 (s), 1268 (m), 1110 (s), 978 (m), 817 (m), 794 (m); HRMS (ESI, *m/z*): calcd for $C_{13}H_{14}O_4NClNa^+$ (M + Na^+), 306.0504, found 306.0496.

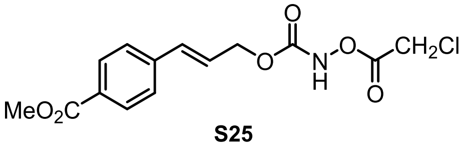

S25

Methyl (*E*)-4-(3-(((2-chloroacetoxy)carbamoyl)oxy)prop-1-en-1-yl)benzoate (**S25**): by following the general procedure, **S25** was obtained as a white solid (68% yield, m.p. 72–75 °C). ^{1}H NMR (400 MHz, $CDCl_3$): δ 8.20 (s, 1H), 8.02 (d, *J* = 8.4 Hz, 2H), 7.47 (d, *J* = 8.4 Hz, 2H), 6.74 (d, *J* = 15.8 Hz, 1H), 6.39 (dt, *J* = 15.9, 6.3 Hz, 1H), 4.90 (dd, *J* = 6.3, 1.4 Hz, 2H), 4.25 (s, 2H), 3.94 (s, 3H); ^{13}C NMR (100 MHz, $CDCl_3$): δ 166.8, 156.0, 136.7, 135.8, 134.6, 128.7, 128.0, 127.3, 126.6, 125.4, 67.4, 38.6; IR ν_{max} (neat)/cm^{-1}: 3262 (m), 3015 (w), 2954 (w), 1793 (w), 1747 (m), 1696 (s), 1435 (m), 1280 (s), 1252 (s), 1130 (s), 960 (m), 750 (s); HRMS (ESI, *m/z*): calcd for $C_{14}H_{14}O_6NClNa^+$ (M + Na^+), 350.0402, found 350.0391.

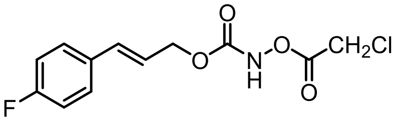

S26

(*E*)-3-(4-fluorophenyl)allyl (2-chloroacetoxy)carbamate (**S26**): by following the general procedure, **S26** was obtained as a white solid (86% yield, m.p. 68–70 °C). ^{1}H NMR (400 MHz, $CDCl_3$): δ 8.40 (s, 1H), 7.37 (dd, J = 8.4, 5.3 Hz, 2H), 7.03 (t, J = 8.5 Hz, 2H), 6.66 (d, J = 15.8 Hz, 1H), 6.20 (dt, J = 15.9, 6.6 Hz, 1H), 4.85 (d, J = 6.6 Hz, 2H), 4.24 (s, 2H); ^{13}C NMR (100 MHz, $CDCl_3$): δ 166.8, 162.7 (d, J = 247.9 Hz), 155.9, 134.2, 132.0 (d, J = 3.3 Hz), 128.4 (d, J = 8.1 Hz), 121.6 (d, J = 2.3 Hz), 115.6 (d, J = 21.7 Hz), 67.5, 38.5; ^{19}F NMR (376 MHz, $CDCl_3$): δ -113.2 (tt, J = 9.0, 5.1 Hz); IR ν_{max} (neat)/cm^{-1}: 3199 (m), 3006 (w), 2957 (w), 1805 (s), 1739 (s), 1715 (s), 1507 (s), 1481(s), 1266 (s), 1226 (s), 1115 (s), 982 (s), 821 (m), 763 (m); HRMS (ESI, *m/z*): calcd for $C_{12}H_{11}O_4NClFNa^+$ (M + Na^+), 310.0253, found 310.0244.

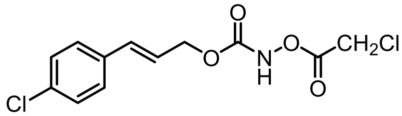

S27

(*E*)-3-(4-chlorophenyl)allyl (2-chloroacetoxy)carbamate (**S27**): by following the general procedure, **S27** was obtained as a white solid (76% yield, m.p. 80–82 °C). ^{1}H NMR (400 MHz, $CDCl_3$): δ 8.19 (s, 1H), 7.33–7.28 (m, 4H), 6.64 (d, J = 15.9 Hz, 1H), 6.24 (dt, J = 15.9, 6.5 Hz, 1H), 4.85 (dd, J = 6.5, 1.3 Hz, 2H), 4.23 (s, 2H); ^{13}C NMR (100 MHz, $CDCl_3$): δ 166.8, 155.8, 134.3, 134.1, 134.1, 128.9, 127.9, 122.5, 67.4, 38.5; IR ν_{max} (neat)/cm^{-1}: 3200 (s), 3007 (w), 2941 (w), 1805 (s), 1739 (m), 1714 (s), 1481(s), 1405 (m), 1264 (s), 1115 (s), 1089 (s), 978 (s), 799 (s); HRMS (ESI, *m/z*): calcd for $C_{12}H_{11}O_4NCl_2Na^+$ (M + Na^+), 325.9957, found 325.9948.

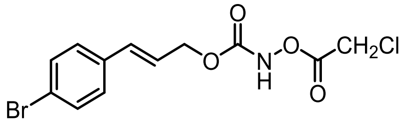

S28

(*E*)-3-(4-bromophenyl)allyl (2-chloroacetoxy)carbamate (**S28**): by following the general procedure, **S28** was obtained as a white solid (82% yield, m.p. 99–100 °C). ^{1}H NMR (400 MHz,

CDCl3): δ 8.18 (s, 1H), 7.45 (d, *J* = 8.5 Hz, 2H), 7.25 (d, *J* = 8.5 Hz, 2H), 6.62 (d, *J* = 15.8 Hz, 1H), 6.25 (dt, *J* = 15.9, 6.4 Hz, 1H), 4.84 (dd, *J* = 6.4, 1.3 Hz, 2H), 4.22 (s, 2H); ^{13}C NMR (100 MHz, CDCl3): δ 166.7, 155.8, 134.8, 134.1, 131.8, 128.2, 122.7, 122.3, 67.3, 38.5; IR ν_{max} (neat)/cm^{-1}: 3675 (m), 3205 (w), 2988 (s), 2901 (s), 1804 (m), 1716 (s), 1483 (m), 1405 (m), 1265 (s), 1117 (s), 1027 (s), 797 (s); HRMS (ESI, *m/z*): calcd for $C_{12}H_{11}O_4NBrClNa^+$ (M + Na$^+$), 369.9601, found 369.9596.

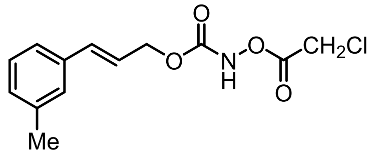

S29

(*E*)-3-(*m*-tolyl)allyl (2-chloroacetoxy)carbamate (**S29**): by following the general procedure, **S29** was obtained as a white solid (81% yield, m.p. 51–53 °C). ^{1}H NMR (400 MHz, CDCl3): δ 8.46 (s, 1H), 7.24–7.18 (m, 3H), 7.13–7.06 (m, 1H), 6.65 (d, *J* = 15.9 Hz, 1H), 6.25 (dt, *J* = 15.8, 6.6 Hz, 1H), 4.84 (dd, *J* = 6.6, 1.2 Hz, 2H), 4.21 (s, 2H), 2.35 (s, 3H); ^{13}C NMR (100 MHz, CDCl3): δ 166.8, 156.1, 138.3, 135.8, 135.6, 129.2, 128.6, 127.5, 123.9, 121.6, 67.7, 38.6, 21.4; IR ν_{max} (neat)/cm^{-1}: 3206 (m), 3006 (w), 2960 (w), 1800 (m), 1744 (m), 1719 (s), 1480 (s), 1266 (m), 1112 (s), 975 (m), 819 (m), 791 (m); HRMS (ESI, *m/z*): calcd for $C_{13}H_{14}O_4NClNa^+$ (M + Na$^+$), 306.0504, found 306.0496.

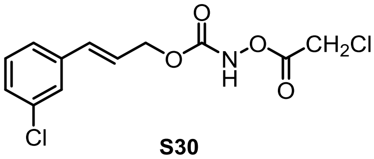

S30

(*E*)-3-(3-chlorophenyl)allyl (2-chloroacetoxy)carbamate (**S30**): by following the general procedure, **S30** was obtained as a white solid (77% yield, m.p. 49–51 °C). ^{1}H NMR (400 MHz, CDCl3): δ 8.29 (s, 1H), 7.37 (s, 1H), 7.28–7.22 (m, 3H), 6.62 (d, *J* = 16.0 Hz, 1H), 6.27 (dt, *J* = 15.8, 6.4 Hz, 1H), 4.85 (dd, *J* = 6.4, 1.2 Hz, 2H), 4.23 (s, 2H); ^{13}C NMR (100 MHz, CDCl3): δ 166.8, 155.8, 137.7, 134.6, 133.7, 129.9, 128.3, 126.6, 125.0, 123.5, 67.2, 38.5; IR ν_{max} (neat)/cm^{-1}: 3206 (m), 2956 (w), 1778 (s), 1752 (s), 1722 (s), 1594 (m), 1565(m), 1460 (m), 1244

(s), 1215 (s), 1112 (s), 965 (s), 773 (s); HRMS (ESI, *m/z*): calcd for $C_{12}H_{11}O_4NCl_2Na^+$ (M + Na^+), 325.9957, found 325.9947.

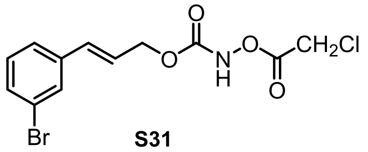

S31

(*E*)-3-(3-bromophenyl)allyl (2-chloroacetoxy)carbamate (**S31**): by following the general procedure, **S31** was obtained as a white solid (76% yield, m.p. 64–67 °C). ^{1}H NMR (400 MHz, $CDCl_3$): δ 8.28 (s, 1H), 7.53 (s, 1H), 7.40 (d, *J* = 7.8 Hz, 1H), 7.30 (d, *J* = 7.8 Hz, 1H), 7.20 (t, *J* = 7.8 Hz, 1H), 6.61 (d, *J* = 16.0 Hz, 1H), 6.27 (dt, *J* = 15.9, 6.4 Hz, 1H), 4.85 (dd, *J* = 6.4, 1.4 Hz, 2H), 4.22 (s, 2H); ^{13}C NMR (100 MHz, $CDCl_3$): δ 166.7, 155.8, 138.0, 133.6, 131.2, 130.2, 129.6, 125.4, 123.5, 122.8, 67.1, 38.5; IR ν_{max} (neat)/cm^{-1}: 3206 (m), 3032 (w), 2971 (w), 2955 (w), 2930 (w), 1777 (s), 1750(s), 1563 (m), 1238 (s), 1136 (s), 966 (s), 799 (s); HRMS (ESI, *m/z*): calcd for $C_{12}H_{11}O_4NBrClNa^+$ (M + Na^+), 369.9601, found 369.9596.

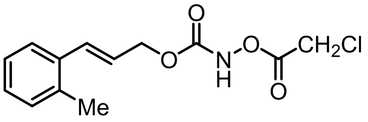

S32

(*E*)-3-(*o*-tolyl)allyl (2-chloroacetoxy)carbamate (**S32**): by following the general procedure, **S32** was obtained as a white solid (64% yield, m.p. 70–72 °C). ^{1}H NMR (400 MHz, $CDCl_3$): δ 8.24 (s, 1H), 7.45–7.42 (m, 1H), 7.21–7.14 (m, 3H), 6.92 (d, *J* = 15.7 Hz, 1H), 6.16 (dt, *J* = 15.7, 6.6 Hz, 1H), 4.87 (dd, *J* = 6.5, 1.0 Hz, 2H), 4.22 (s, 2H), 2.35 (s, 3H); ^{13}C NMR (100 MHz, $CDCl_3$): δ 166.7, 155.9, 135.8, 135.0, 133.4, 130.4, 128.2, 126.2, 125.9, 123.1, 67.8, 38.5, 19.7; IR ν_{max} (neat)/cm^{-1}: 3210 (m), 3004 (w), 2956 (w), 1807 (m), 1742 (m), 1715 (s), 1480 (s), 1272 (m), 1102 (s), 976 (m), 820 (m), 790 (m); HRMS (ESI, *m/z*): calcd for $C_{13}H_{14}O_4NClNa^+$ (M + Na^+), 306.0504, found 306.0494.

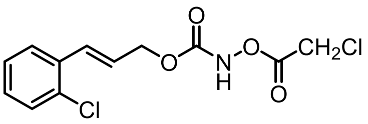

S33

(*E*)-3-(2-chlorophenyl)allyl (2-chloroacetoxy)carbamate (**S33**): by following the general procedure, **S33** was obtained as a white solid (69% yield, m.p. 78–80 °C). ^{1}H NMR (400 MHz, $CDCl_3$): δ 8.16 (s, 1H), 7.58–7.47 (m, 1H), 7.42–7.31 (m, 1H), 7.25–7.18 (m, 2H), 7.08 (dt, *J* = 15.9, 1.4 Hz, 1H), 6.26 (dt, *J* = 15.8, 6.4 Hz, 1H), 4.90 (dd, *J* = 6.4, 1.4 Hz, 2H), 4.23 (s, 2H); ^{13}C NMR (100 MHz, $CDCl_3$): δ 166.7, 155.7, 134.0, 133.4, 131.2, 129.8, 129.3, 127.1, 127.0, 124.7, 67.4, 38.5; IR ν_{max} (neat)/cm^{-1}: 3200 (m), 3029 (w), 2973 (w), 2929 (w), 1777 (s), 1754 (s), 1455 (m), 1235 (s), 1133 (s), 969 (s), 804 (m), 726 (s); HRMS (ESI, *m/z*): calcd for $C_{12}H_{11}O_4NCl_2Na^+$ (M + Na^+), 325.9957, found 325.9948.

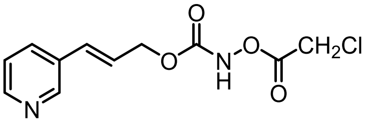

S34

(*E*)-3-(pyridin-3-yl)allyl (2-chloroacetoxy)carbamate (**S34**): by following the general procedure, **S34** was obtained as a white solid (55% yield, m.p. 69–71 °C). ^{1}H NMR (400 MHz, $CDCl_3$): δ 9.03 (s, 1H), 8.61 (d, *J* = 2.2 Hz, 1H), 8.50 (dd, *J* = 4.8, 1.6 Hz, 1H), 7.72 (dt, *J* = 8.0, 2.0 Hz, 1H), 7.30–7.27 (m, 1H), 6.67 (d, *J* = 16.0 Hz, 1H), 6.34 (dt, *J* = 16.0, 6.2 Hz, 1H), 4.87 (dd, *J* = 6.2, 1.4 Hz, 2H), 4.23 (s, 2H); ^{13}C NMR (100 MHz, $CDCl_3$): δ 166.7, 155.9, 149.1, 148.3, 133.4, 131. 7, 131.1, 124.6, 123.6, 66.8, 38.5; IR ν_{max} (neat)/cm^{-1}: 3225 (m), 3026 (w), 3006 (w), 2959 (w), 1750 (m), 1726 (s), 1460 (s), 1208 (m), 1112 (s), 960 (m), 820 (m), 790 (m); HRMS (ESI, *m/z*): calcd for $C_{11}H_{11}O_4N_2ClNa^+$ (M + Na^+), 293.0402, found 293.0410.

c. General Procedure for the Iron-Catalyzed Asymmetric Olefin Aminochlorination and Product Characterization

General Procedure. To a flame-dried sealable 2-dram vial (vial **A**) equipped with a magnetic stir bar were added $Fe(NTf_2)_2$ (9.2 mg, 0.015 mmol, 15 mol %) and ligand **L5** (7.3 mg, 0.015 mmol, 15 mol %). After the vial was evacuated and backfilled with N_2 for three times, $CHCl_3$

(1.0 mL, re-distilled and anhydrous) was added and the mixture was stirred at room temperature for 20 min. Meanwhile, a second flame-dried and N_2-protected 2-dram vial (vial **B**) with a magnetic stir bar was charged with the substrate (0.1 mmol), anhydrous TBAC (69.5 mg, 0.25 mmol), freshly activated 4 Å molecular sieves and $CHCl_3$ (3.0 mL, re-distilled and anhydrous). Both vials were degassed by brief evacuation and back filling with N_2 twice. Vial **B** was cooled down to -60 °C, and the catalyst solution in vial **A** was added to vial **B** drop wise via a syringe. The resulting solution was stirred at this temperature for 12 h and then gradually warmed to room temperature. The reaction was quenched with 1 mL saturated $NaHCO_3$ solution. The reaction mixture was extracted with CH_2Cl_2 (1.5 mL × 3), and the combined organic phase was concentrated *in vacuo*. The residue was purified through a gradient silica gel flash column chromatography (hexanes/acetone: from 15:1 to 4:1) to afford the aminochlorination product. The *dr* was determined by ^1H NMR analysis and the *ee* was measured by chiral HPLC analysis. The assignment of diastereomers on HPLC traces was based on ^1H NMR analysis.

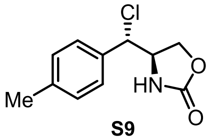

S9

(*R*)-4-((*R*)-chloro(*p*-tolyl)methyl)dihydrofuran-2(3H)-one (**S9**) : by following the general procedure, the product **S9** was obtained as a white solid (14 mg, 65% yield, *dr*: 15:1). The *ee* was determined by chiral HPLC analysis (Chiral OD-H column, 7% EtOH in hexanes, flow rate = 0.9 mL/min, UV detection at 230 nm). The *anti*-diastereomer: t_r (minor) = 35.1 min, t_r (major) = 32.3 min, 91% *ee*; the *syn*-diastereomer: t_r (minor) = 39.0 min, t_r (major) = 36.7 min, <5% *ee*.

Racemic sample

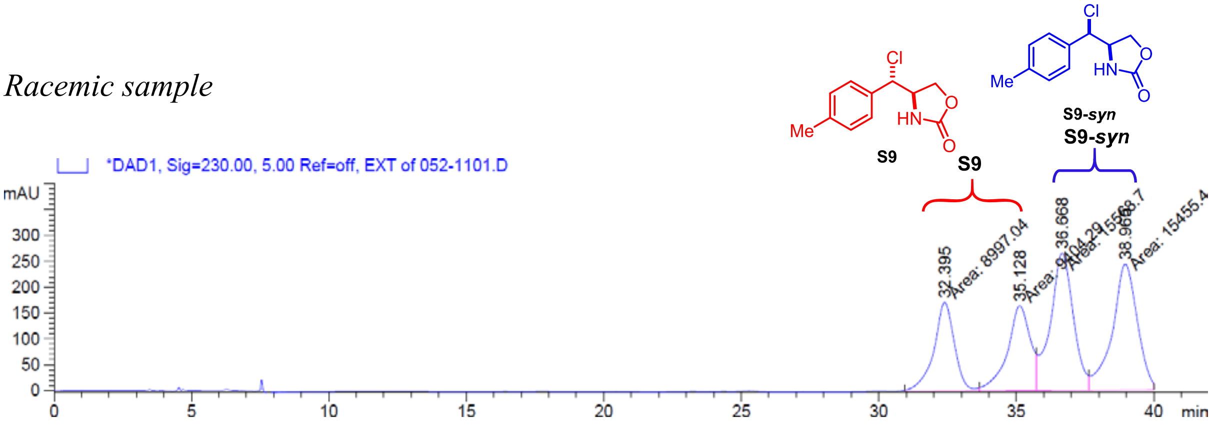

Peak #	RetTime [min]	Type	Width [min]	Area [mAU*s]	Height [mAU]	Area %
1	32.395	MF	0.8743	8997.03711	171.50110	18.2033
2	35.128	MF	0.9537	9404.28613	164.34309	19.0272
3	36.668	MF	0.9768	1.55687e4	265.64551	31.4993
4	38.965	FM	1.0559	1.54554e4	243.95905	31.2702
Totals :				4.94254e4	845.44875	

Enantio-enriched sample (91% ee): it was obtained by using chiral ligand ***ent*-L5**

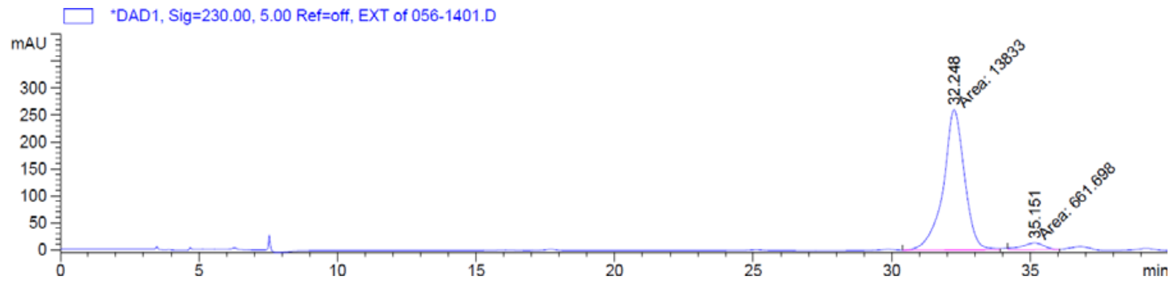

Peak #	RetTime [min]	Type	Width [min]	Area [mAU*s]	Height [mAU]	Area %
1	32.248	MF	0.8868	1.38330e4	259.96939	95.4349
2	35.151	FM	0.8816	661.69800	12.51001	4.5651
Totals :				1.44947e4	272.47940	

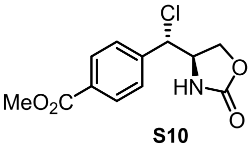

S10

Methyl 4-((*S*)-chloro((*R*)-2-oxooxazolidin-4-yl)methyl)benzoate (**S10**): by following the general procedure, the product **S10** was obtained as a white solid (19 mg, 69% yield, *dr*: 5.2:1). The *ee* was determined by chiral HPLC analysis (Chiral OD-H column, 10% EtOH in hexanes, flow rate = 1.0 mL/min, UV detection at 205 nm). The *anti*-diastereomer: t_r (minor) = 34.4 min, t_r (major) = 38.6 min, 86% *ee*; the *syn*-diastereomer: t_r (minor) = 42.2 min, t_r (major) = 45.1 min, <5% *ee*.

Racemic sample

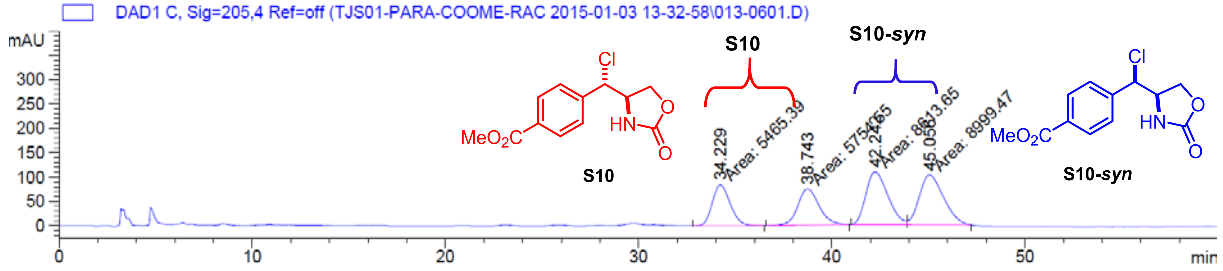

Peak #	RetTime [min]	Type	Width [min]	Area [mAU*s]	Height [mAU]	Area %
1	34.229	MM	1.0728	5465.39160	84.91122	18.9553
2	38.743	MM	1.2763	5754.54785	75.14704	19.9582
3	42.242	MF	1.3189	8613.65039	108.84999	29.8742
4	45.056	FM	1.4569	8999.46875	102.95222	31.2123

Totals : 2.88331e4 371.86047

Enantio-enriched sample (86% *ee*)

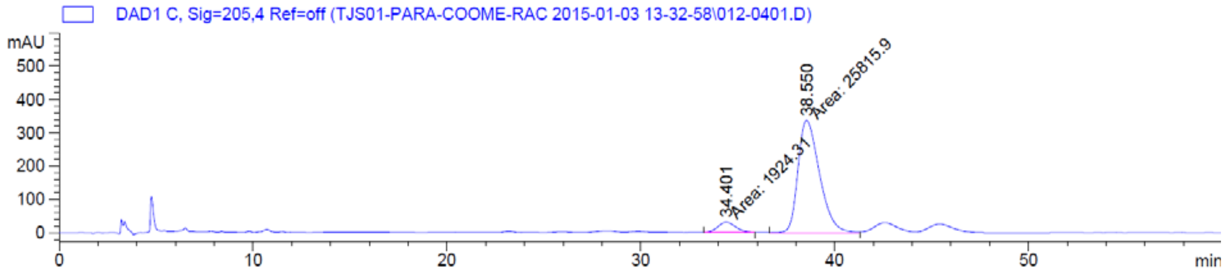

Peak #	RetTime [min]	Type	Width [min]	Area [mAU*s]	Height [mAU]	Area %
1	34.401	MM	1.0437	1924.31250	30.72984	6.9369
2	38.550	MM	1.2800	2.58159e4	336.15533	93.0631

Totals : 2.77402e4 366.88517

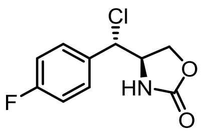

S35

(*R*)-4-((*S*)-chloro(4-fluorophenyl)methyl)oxazolidin-2-one (**S35**): by following the general procedure, the product **S35** was obtained as a white solid (20 mg, 84% yield, *dr*: 12:1, m.p. 109–111 °C). ^{1}H NMR (400 MHz, $CDCl_3$): δ 7.42–7.35 (m, 2H), 7.15–7.08 (m, 2H), 5.28 (s, 1H), 4.73 (d, J = 8.8 Hz, 1H), 4.60 (dd, J = 9.4, 8.2 Hz, 1H), 4.45 (dd, J = 9.5, 4.8 Hz, 1H), 4.24 (ddd, J = 8.7, 8.2, 5.3 Hz, 1H); ^{13}C NMR (100 MHz, $CDCl_3$): δ 163.2 (d, J = 249.8 Hz), 158.2, 132.5 (d, J = 3.6 Hz), 129.6 (d, J = 8.5 Hz), 116.4 (d, J = 21.7 Hz), 68.4, 62.7, 58.3; ^{19}F NMR (376 MHz, $CDCl_3$) δ -110.89 (td, J = 8.2, 4.4 Hz); IR v_{max} (neat)/cm^{-1}: 3236 (m), 3131 (w), 2929 (w), 2850 (w), 1727 (s), 1605 (m), 1510 (s), 1409 (m), 1394 (m), 1232 (s), 1023 (s), 838 (m); HRMS (ESI, *m/z*): calcd for $C_{10}H_{10}O_2NClFH^+$ (M + H^+), 230.0379, found 230.0374; The *ee* was determined by Chiral HPLC analysis (Chiral *S*, *S*, Whelk column, 5% EtOH in hexanes, flow rate = 1.0 mL/min, UV detection at 205 nm). The *anti*-diastereomer: t_r (minor) = 37.0 min, t_r (major) = 39.0 min, 90% *ee*; the *syn*-diastereomer: t_r (minor) = 41.8 min, t_r (major) = 43.6 min, <5% *ee*.

Racemic sample

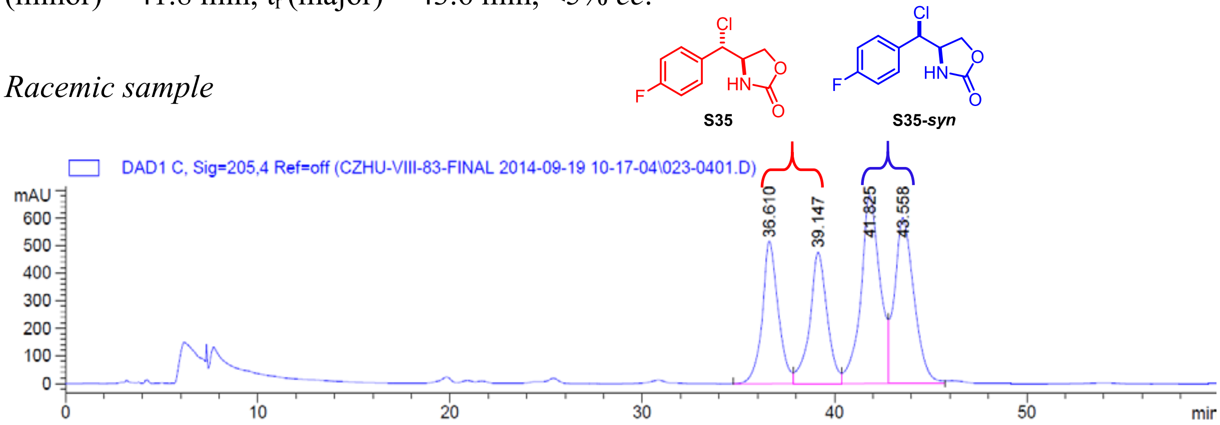

Peak #	RetTime [min]	Type	Width [min]	Area [mAU*s]	Height [mAU]	Area %
1	36.610	BV	0.8492	2.98748e4	516.22046	20.0044
2	39.147	VV	0.9226	3.00566e4	474.74084	20.1261
3	41.825	VV	0.9995	4.70431e4	681.33008	31.5004
4	43.558	VB	1.0435	4.23666e4	600.24622	28.3690
Totals :				1.49341e5	2272.53760	

Enantio-enriched sample (90% *ee*)

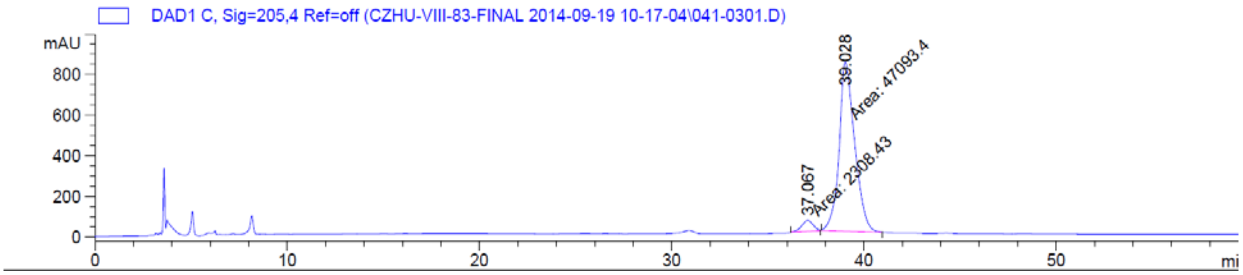

Signal 3: DAD1 C, Sig=205,4 Ref=off

Peak #	RetTime [min]	Type	Width [min]	Area [mAU*s]	Height [mAU]	Area %
1	37.067	MM	0.6858	2308.43481	56.09760	4.6728
2	39.028	MM	0.9420	4.70934e4	833.26031	95.3272

Totals : 4.94018e4 889.35791

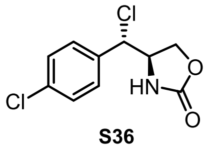

S36

(*R*)-4-((*S*)-chloro(4-chlorophenyl)methyl)oxazolidin-2-one (**S36**): by following the general procedure, the product **S36** was obtained as a white solid (15 mg, 62% yield, *dr*: 11:1, m.p. 123–126 °C). ^{1}H NMR (400 MHz, $CDCl_3$): δ 7.41 (d, J = 8.6 Hz, 2H), 7.34 (d, J = 8.5 Hz, 2H), 5.07 (s, 1H), 4.71 (d, J = 8.9 Hz, 1H), 4.61 (dd, J = 9.5, 8.2 Hz, 1H), 4.45 (dd, J = 9.5, 4.8 Hz, 1H), 4.27–4.17 (m, 1H); ^{13}C NMR (100 MHz, $CDCl_3$): δ 158.0, 135.7, 135.0, 129.5, 129.1, 68.4, 62.6, 58.2; IR ν_{max} (neat)/cm^{-1}: 3259 (m), 3012 (w), 2986 (w), 1765 (s), 1472 (m), 1239 (m), 1018 (m), 755 (m), 500 (s); HRMS (ESI, m/z): calcd for $C_{10}H_{10}NO_2Cl_2^+$ (M + H^+), 246.0089, found 246.0084. The *ee* was determined by Chiral HPLC analysis (Chiral OD-H column, 10% EtOH in hexanes, flow rate = 1.0 mL/min, UV detection at 230 nm). The *anti*-diastereomer: t_r (minor) = 20.1 min, t_r (major) = 18.7 min, 88% *ee*; the *syn*-diastereomer: t_r (minor) = 26.7 min, t_r (major) = 28.5 min, <5% *ee*.

Racemic sample

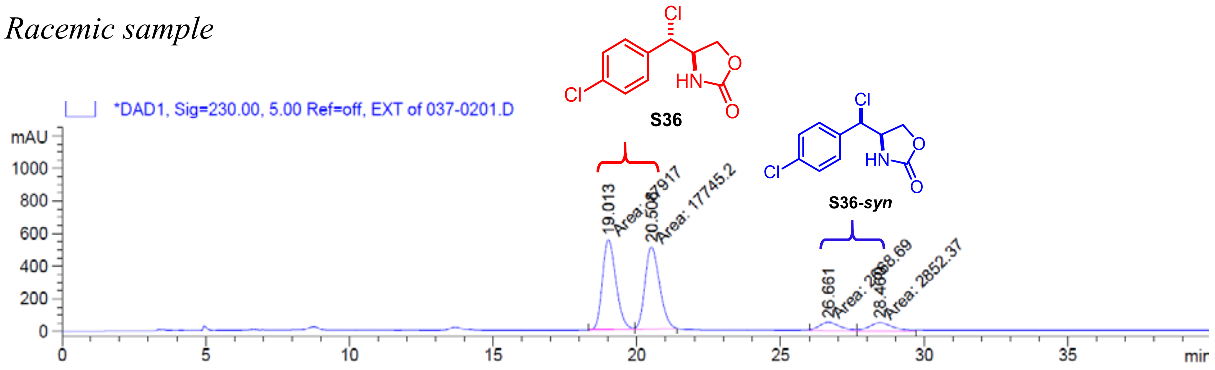

Peak #	RetTime [min]	Type	Width [min]	Area [mAU*s]	Height [mAU]	Area %
1	19.013	MF	0.5429	1.79170e4	550.01447	43.5055
2	20.508	FM	0.5875	1.77452e4	503.38525	43.0884
3	26.661	MF	0.8563	2668.69336	51.94394	6.4800
4	28.460	FM	0.9582	2852.37085	49.61445	6.9260
Totals :				4.11833e4	1154.95811	

Enantio-enriched sample (88% *ee*)

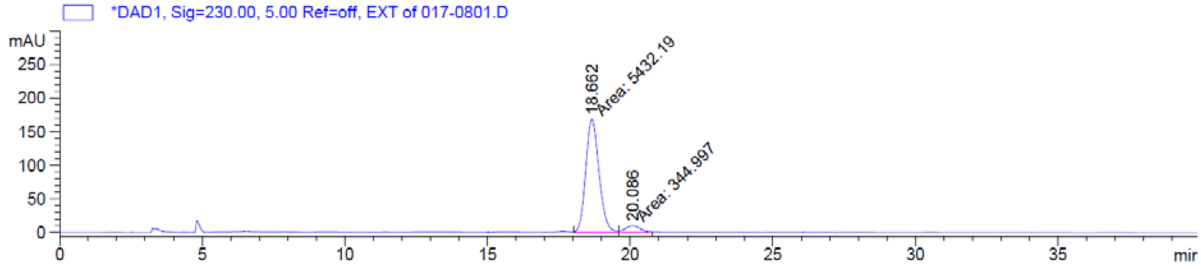

Peak #	RetTime [min]	Type	Width [min]	Area [mAU*s]	Height [mAU]	Area %
1	18.662	MF	0.5386	5432.18506	168.08821	94.0283
2	20.086	FM	0.5822	344.99683	9.87630	5.9717
Totals :				5777.18188	177.96451	

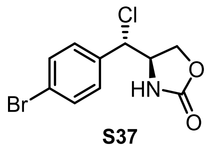

S37

(*R*)-4-((*S*)-chloro(4-bromophenyl)methyl)oxazolidin-2-one (**S37**): by following the general procedure, the product **S37** was obtained as a white solid (21 mg, 71% yield, *dr*: 11:1, m.p. 139–141°C). ^{1}H NMR (400 MHz, $CDCl_3$): δ 7.56 (d, *J* = 8.4 Hz, 2H), 7.27 (d, *J* = 8.4 Hz, 2H), 5.33 (s, 1H), 4.70 (d, *J* = 8.7 Hz, 1H), 4.58 (t, *J* = 8.8 Hz, 1H), 4.44 (dd, *J* = 9.4, 4.8 Hz, 1H), 4.23 (td, *J* = 8.6, 4.7 Hz, 1H); ^{13}C NMR (100 MHz, $CDCl_3$): δ 158.2, 135.6, 132.5, 129.3, 123.8, 68.3, 62.7, 58.2; IR ν_{max} (neat)/cm^{-1}: 3274 (m), 2923 (w), 2851 (w), 1757 (s), 1488 (m), 1405 (m), 1239 (m), 1010 (m); HRMS (ESI, *m/z*): calcd for $C_{10}H_{10}O_2NBrCl^+$ (M + H^+), 289.9578, found 289.9574. $[\alpha]_D^{20}$ = + 11.4 ° (*c* 1.0, CH_2Cl_2). The *ee* was determined by Chiral HPLC analysis (Chiral OD-H column, 10% EtOH in hexanes, flow rate = 1.0 mL/min, UV detection at 205 nm). The *anti*-diastereomer: t_r (minor) = 21.9 min, t_r (major) = 19.6 min, 86% *ee*; the *syn*-diastereomer: t_r (minor) = 26.6 min, t_r (major) = 29.6 min, <5% *ee*.

Racemic sample

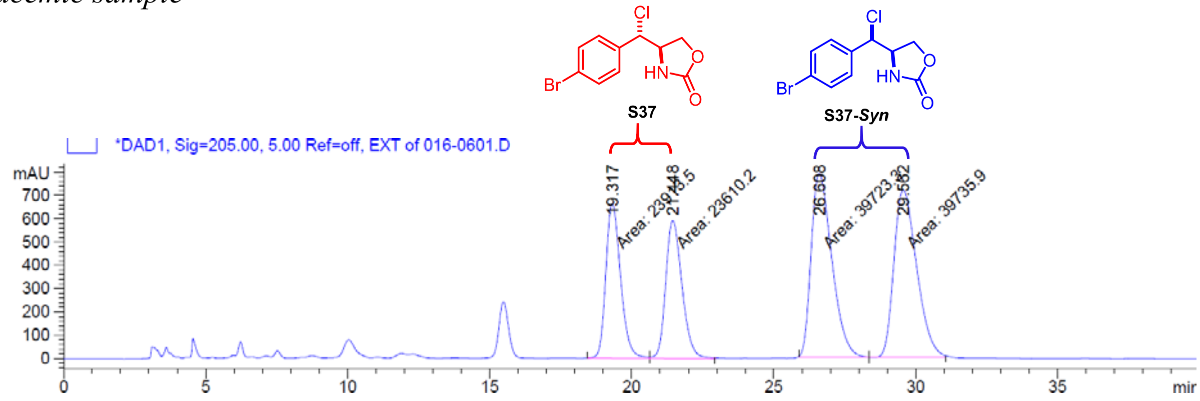

Peak #	RetTime [min]	Type	Width [min]	Area [mAU*s]	Height [mAU]	Area %
1	19.317	MF	0.5991	2.39135e4	665.23224	18.8321
2	21.448	FM	0.6637	2.36102e4	592.91406	18.5932
3	26.608	MF	0.8405	3.97233e4	787.70642	31.2824
4	29.562	FM	0.9245	3.97359e4	716.35968	31.2923
Totals :				1.26983e5	2762.21240	

Enantio-enriched sample (86% *ee*)

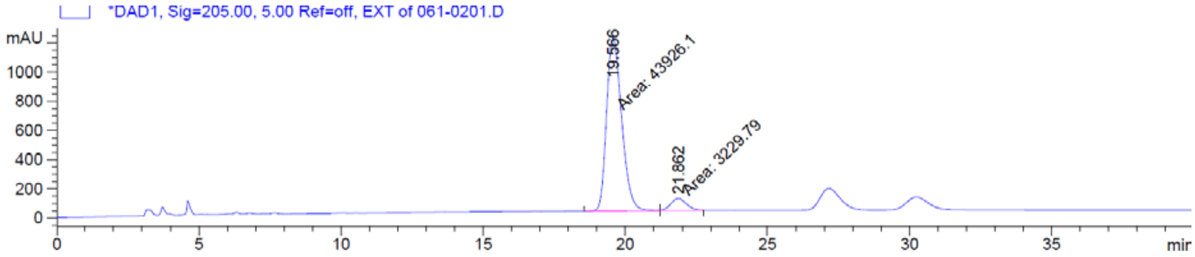

Peak #	RetTime [min]	Type	Width [min]	Area [mAU*s]	Height [mAU]	Area %
1	19.566	MF	0.6162	4.39261e4	1188.04236	93.1508
2	21.862	FM	0.6504	3229.79492	82.76894	6.8492
Totals :				4.71559e4	1270.81130	

The absolute stereochemistry of **S37** was determined by X-ray crystallographic analysis. The crystal structure has been deposited in The Cambridge Crystallographic Data Centre as CCDC 1041826.

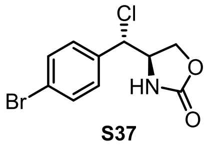

S37

Structure Plots

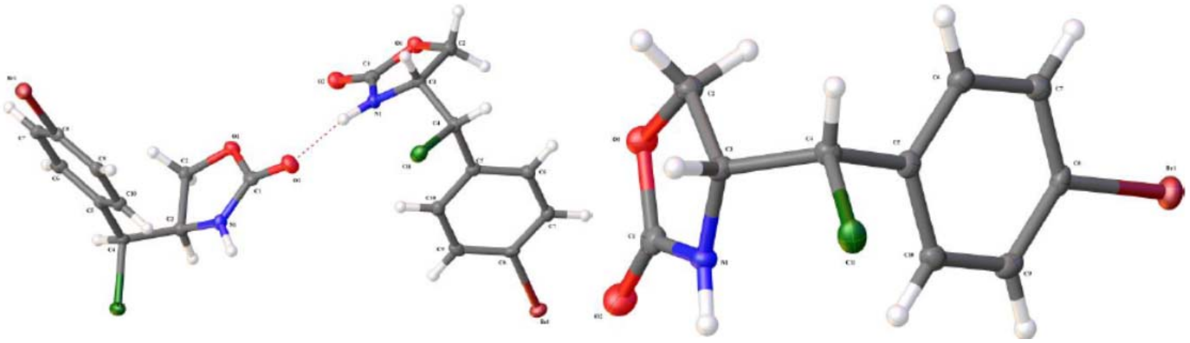

Crystal Data and Experimental

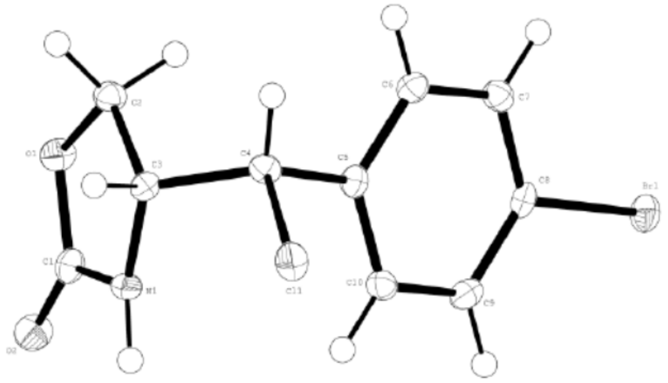

Experimental. Single colorless prism-shaped crystals of (**czhu-para-br-acl**) were recrystallised from DCM by slow evaporation. A suitable crystal (0.32 × 0.20 × 0.14 mm^3) was selected and mounted on a loop with paratone oil on a Bruker APEX-II CCD diffractometer. The crystal was cooled to T = 110(2) K during data collection. The structure was solved with the **ShelXD** (Sheldrick, 2008) structure solution program using **Olex2** (Dolomanov et al., 2009), using the Dual Space solution method. The structure was refined with version 2013-4 of **ShelXL-97** (Sheldrick, 2008) using Least Squares minimisation.

Crystal Data. $C_{10}H_9BrClNO_2$, M_r = 290.54, monoclinic, P2$_1$ (No. 4), a = 5.9280(4) Å, b = 7.6682(5) Å, c = 11.3533(8) Å, β = 95.944(3)°, α = γ = 90°, V = 513.31(6) Å^3, T = 110(2) K, Z = 2, Z' = 1, μ (MoK$_\alpha$) = 4.240, 5260 reflections measured, 2561 unique (R_{int} = 0.0271) which were used in all calculations. The final wR_2 was 0.0544 (all data) and R_1 was 0.0264 (I > 2(I)).

**Compound**	**czhu-para-br-acl**
Formula	$C_{10}H_9BrClNO_2$
$D_{calc.}$/ g cm^{-3}	1.880
μ/mm^{-1}	4.240
Formula Weight	290.54
Colour	colourless
Shape	prism
Max Size/mm	0.32
Mid Size/mm	0.20
Min Size/mm	0.14
T/K	110(2)
Crystal System	monoclinic
Space Group	P2$_1$
a/Å	5.9280(4)
b/Å	7.6682(5)
c/Å	11.3533(8)
α/°	90
β/°	95.944(3)
γ/°	90
V/Å^3	513.31(6)
Z	2
Z'	1
Θ_{min}/°	3.211
Θ_{max}/°	28.700
Measured Refl.	5260
Independent Refl.	2561
Reflections Used	2404
R_{int}	0.0271
Parameters	144
Restraints	2
Largest Peak	0.587
Deepest Hole	-0.370
GooF	0.986
wR_2 (all data)	0.0544
wR_2	0.0540
R_1 (all data)	0.0283
R_1	0.0264

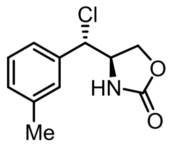

S38

(*R*)-4-((*S*)-chloro(m-tolyl)methyl)oxazolidin-2-one (**S38**): by following the general procedure, the product **S38** was obtained as a white solid (17 mg, 75% yield, *dr*: 12:1, m.p.126–129 °C). ^{1}H NMR (400 MHz, $CDCl_3$): δ 7.33–7.29 (m, 1H), 7.21–7.17 (m, 3H), 4.83 (s, 1H), 4.67 (d, *J* = 9.3 Hz, 1H), 4.62 (dd, *J* = 9.3, 8.3 Hz, 1H), 4.48 (dd, *J* = 9.4, 4.8 Hz, 1H), 4.26 (td, *J* = 8.4, 4.8 Hz, 1H), 2.38 (s, 3H); ^{13}C NMR (100 MHz, $CDCl_3$): δ 158.3, 139.2, 136.5, 130.4, 129.2, 128.3, 124.7, 68.5, 63.6, 58.2, 21.4; IR ν_{max} (neat)/cm^{-1}: 3270 (m), 3144 (w), 3041 (w), 2928 (w), 2341 (w), 1752 (s), 1402 (m), 1276 (m), 1239 (m), 1028 (m), 760 (m); HRMS (ESI, m/z): calcd for $C_{11}H_{13}NO_2Cl^+$ (M + H^+), 226.0635, found 226.0640. The *ee* was determined by Chiral HPLC analysis (Chiral *S*, *S*, Whelk column, 5% EtOH in hexanes, flow rate = 1.0 mL/min, UV detection at 205 nm). The *anti*-diastereomer: t_r (minor) = 18.0 min, t_r (major) = 15.4 min, 87% *ee*; the *syn*-diastereomer: t_r (minor) = 19.8 min, t_r (major) = 22.5 min, <5% *ee*.

Racemic sample

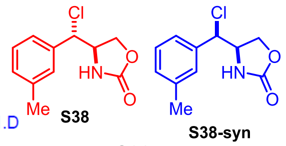

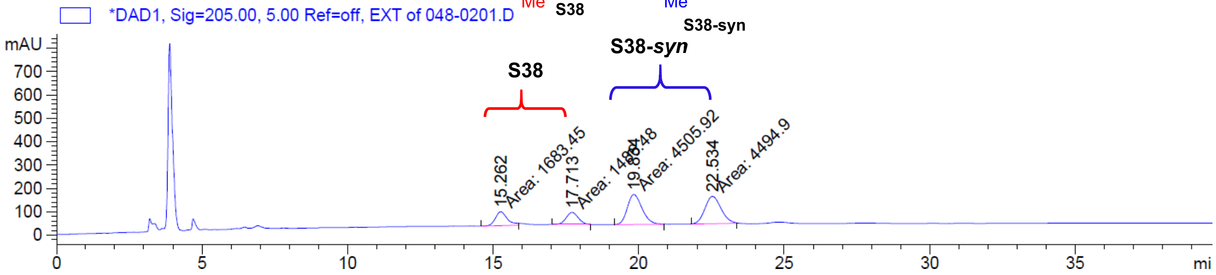

Peak #	RetTime [min]	Type	Width [min]	Area [mAU*s]	Height [mAU]	Area %
1	15.262	MF	0.4749	1683.45300	59.07664	13.8320
2	17.713	MM	0.4815	1486.47937	51.45514	12.2135
3	19.834	MM	0.5866	4505.92090	128.02838	37.0225
4	22.534	MM	0.6419	4494.89893	116.70497	36.9320

Totals : 1.21708e4 355.26514

Enantio-enriched sample (87% *ee*): it was obtained by using ***ent*-L5** ligand.

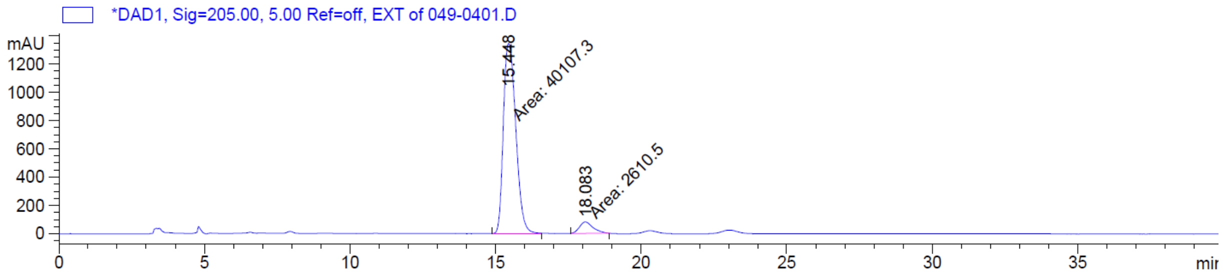

Peak #	RetTime [min]	Type	Width [min]	Area [mAU*s]	Height [mAU]	Area %
1	15.448	MM	0.4958	4.01073e4	1348.12842	93.8890
2	18.083	MM	0.5387	2610.50269	80.75871	6.1110
Totals :				4.27178e4	1428.88713	

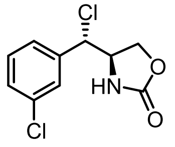

S11

(*R*)-4-((*S*)-chloro(3-chlorophenyl)methyl)oxazolidin-2-one (**S11**): by following the general procedure, the product **S11** was obtained as a white solid (16 mg, 63% yield, *dr*: 10:1). The *ee* was determined by Chiral HPLC analysis (Chiral OD-H column, 10% EtOH in hexanes, flow rate = 1.0 mL/min, UV detection at 205 nm). The *anti*-diastereomer: t_r (minor) = 24.2min, t_r (major) = 20.4 min, 80% *ee*; the *syn*-diastereomer: t_r (minor) = 25.2 min, t_r (major) = 35.2 min, <5% *ee*.

Racemic sample

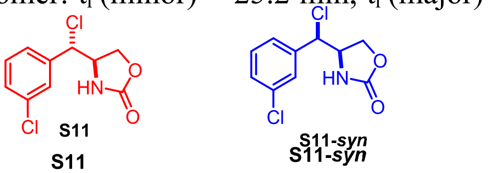

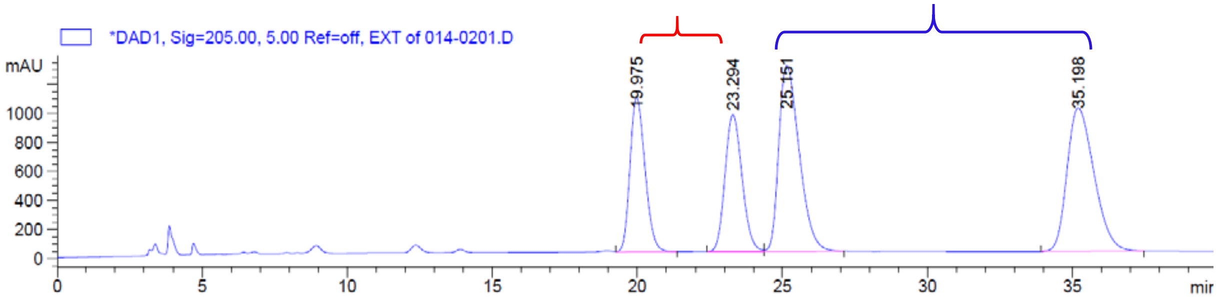

Peak #	RetTime [min]	Type	Width [min]	Area [mAU*s]	Height [mAU]	Area %
1	19.975	VB	0.5620	3.82381e4	1062.09363	18.8698
2	23.294	BV	0.6381	3.84101e4	941.26605	18.9548
3	25.151	VB	0.7544	6.23812e4	1275.87036	30.7841
4	35.198	BB	0.9952	6.36118e4	985.25012	31.3913

Totals : 2.02641e5 4264.48016

Enantio-enriched sample (80% *ee*): it was obtained by using ***ent*-L5** ligand.

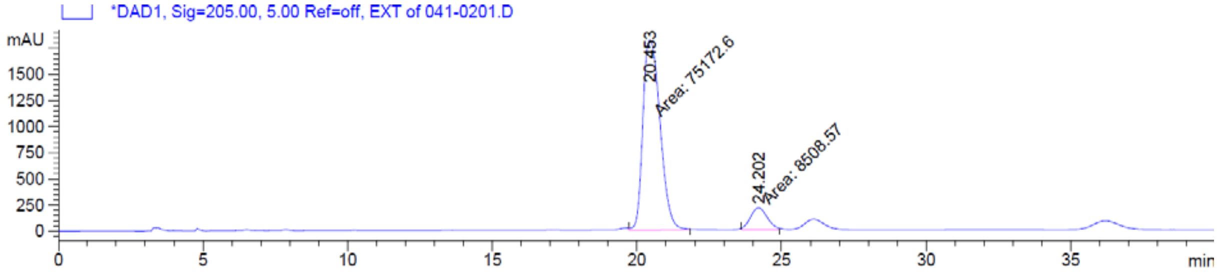

Peak #	RetTime [min]	Type	Width [min]	Area [mAU*s]	Height [mAU]	Area %
1	20.453	MF	0.6884	7.51726e4	1820.07776	89.8322
2	24.202	MF	0.6768	8508.57422	209.52779	10.1678

Totals : 8.36812e4 2029.60555

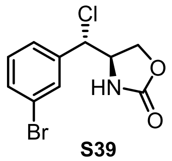

S39

(*R*)-4-((*S*)-chloro(3-bromophenyl)methyl)oxazolidin-2-one (**S39**): by following the general procedure under the condition described in entry 8, the product **S39** was obtained as a white solid (21 mg, 71% yield, *dr*: 15:1, m.p. 113–115 °C). ^{1}H NMR (400 MHz, $CDCl_3$): δ 7.58–7.51 (m, 2H), 7.34–7.28 (m, 1H), 7.32 (s, 1H), 5.05 (s, 1H), 4.67 (d, J = 9.1 Hz, 1H), 4.62 (dd, J = 9.5, 8.2

Hz, 1H), 4.47 (dd, J = 9.5, 4.7 Hz, 1H), 4.24 (ddd, J = 8.7, 8.1, 4.7 Hz, 1H) ; ^{13}C NMR (100 MHz, $CDCl_3$): δ 158.0, 138.8, 132.9, 130.8, 130.7, 126.4, 123.3, 68.4, 62.4, 58.2; IR ν_{max} (neat)/cm^{-1}: 3272 (m), 2923 (w), 2952 (w), 1747 (s), 1476 (m), 1428 (m), 1237(m), 1025(m), 732 (m); HRMS (ESI, *m/z*): calcd for $C_{10}H_{10}O_2NBrCl^+$ (M + H$^+$), 289.9578, found 289.9573. The *ee* was determined by Chiral HPLC analysis (Chiral OD-H column, 10% EtOH in hexanes, flow rate = 1.0 mL/min, UV detection at 205 nm). The *anti*-diastereomer: t_r (minor) = 21.9 min, t_r (major) = 25.6 min, 80% *ee*; the *syn*-diastereomer: t_r (minor) = 26.9 min, t_r (major) = 39.5 min, <5% *ee*.

Racemic sample

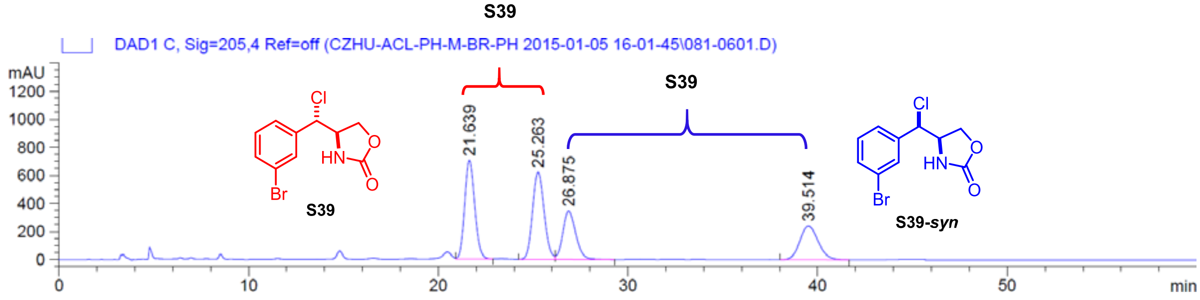

Peak #	RetTime [min]	Type	Width [min]	Area [mAU*s]	Height [mAU]	Area %
1	21.639	VB	0.5793	2.59986e4	703.30060	30.6557
2	25.263	BV	0.6620	2.63187e4	621.50104	31.0332
3	26.875	VB	0.7283	1.63597e4	344.31677	19.2903
4	39.514	BB	1.0267	1.61312e4	240.46060	19.0208
Totals :				8.48081e4	1909.57901	

Enantio-enriched sample (80% *ee*) it was obtained by using **ent-L5** ligand.

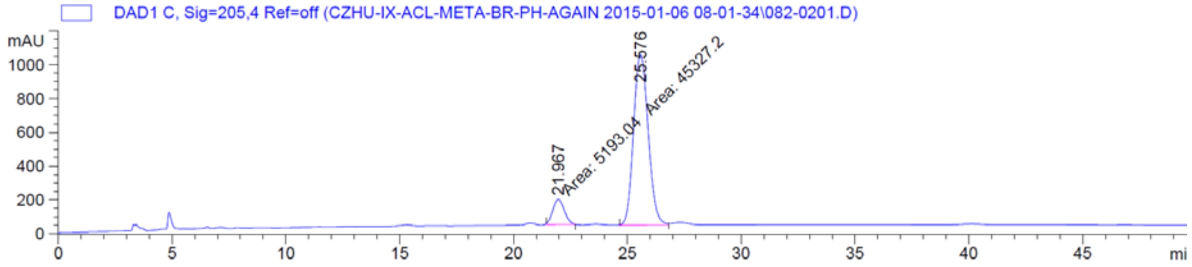

Peak #	RetTime [min]	Type	Width [min]	Area [mAU*s]	Height [mAU]	Area %
1	21.967	MM	0.5812	5193.03906	148.92203	10.2791
2	25.576	MM	0.7473	4.53272e4	1010.88354	89.7209
Totals :				5.05203e4	1159.80557	

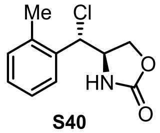

S40

(*R*)-4-((*S*)-chloro(o-tolyl)methyl)oxazolidin-2-one (**S40**): by following the general procedure under the condition described in entry 8, the product **S40** was obtained as a white solid (18 mg, 78% yield, *dr*: 4.5:1, m.p.114–116 °C). ^{1}H NMR (400 MHz, $CDCl_3$): δ 7.32–7.28 (m, 1H), 7.21–7.17 (m, 3H), 7.23–7.20 (m, 1H), 5.01 (s, 1H), 4.68 (d, *J* = 9.2 Hz, 1H), 4.61 (dd, *J* = 9.4, 8.2 Hz, 1H), 4.48 (dd, *J* = 9.4, 4.8 Hz, 1H), 4.29–4.23(m, 1H), 2.38 (s, 3H); ^{13}C NMR (100 MHz, $CDCl_3$): δ 158.4, 136.7, 131.3, 129.4, 127.2, 126.6, 68.8, 59.1, 57.5, 19.4; IR ν_{max} (neat)/cm^{-1}: 3269 (m), 3144 (w), 3035 (w), 2922 (w), 2340 (w), 1750 (s), 1410 (m), 1276 (m), 1235 (m), 1021 (m), 765 (m); HRMS (ESI, m/z): calcd for $C_{11}H_{13}NO_2Cl^+$ (M + H$^+$), 226.0635, found 226.0640. The *ee* was determined by Chiral HPLC analysis (Chiral *S*, *S*, Whelk column, 5% EtOH in hexanes, flow rate = 1.0 mL/min, UV detection at 205 nm). The *anti*-diastereomer: t_r (minor) = 37.8 min, t_r (major) = 34.9 min, 77% *ee*; the *syn*-diastereomer: t_r (minor) = 47.3 min, t_r (major) = 52.5 min, <5% *ee*.

Racemic sample

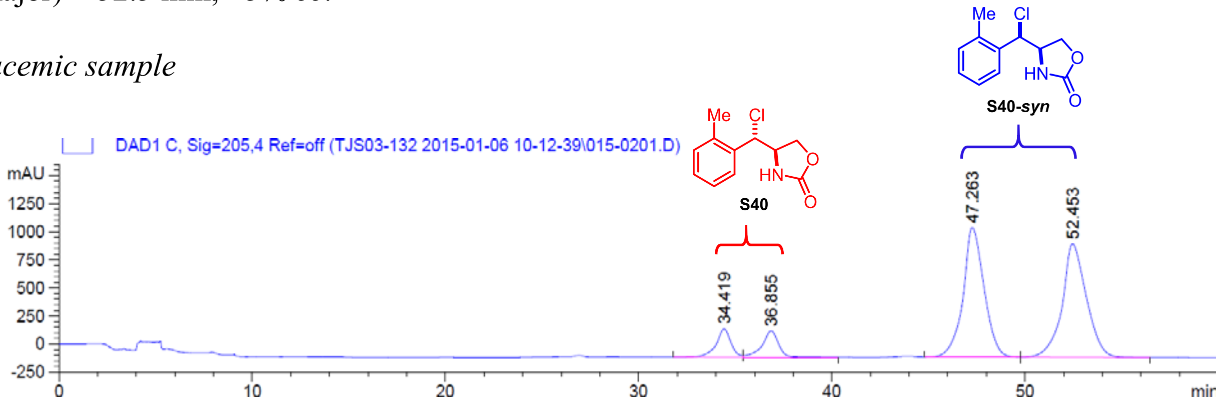

Peak #	RetTime [min]	Type	Width [min]	Area [mAU*s]	Height [mAU]	Area %
1	34.419	BV	0.7849	1.35278e4	252.55251	6.6802
2	36.855	VB	0.8558	1.39221e4	236.16118	6.8750
3	47.263	BB	1.0879	8.70846e4	1154.61365	43.0037
4	52.453	BB	1.2384	8.79705e4	1011.78650	43.4411
Totals :				2.02505e5	2655.11383	

Enantio-enriched sample (77 % *ee*): it was obtained by using ***ent*-L5** ligand.

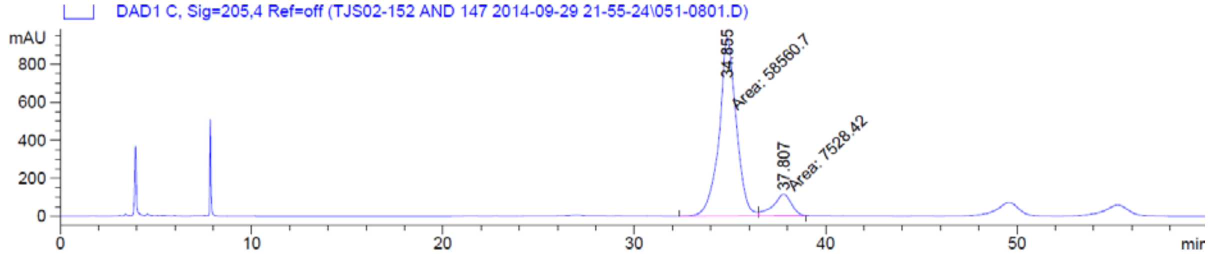

Peak #	RetTime [min]	Type	Width [min]	Area [mAU*s]	Height [mAU]	Area %
1	34.855	MF	1.0397	5.85607e4	938.72003	88.6087
2	37.807	FM	1.1122	7528.41846	112.82027	11.3913
Totals :				6.60891e4	1051.54031	

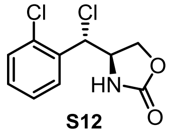

S12

(*R*)-4-((*S*)-chloro(2-chlorophenyl)methyl)oxazolidin-2-one (**S12**): by following the general procedure, the product **S1** was obtained as a white solid (14 mg, 55% yield, *dr*: 12:1). The *ee* was determined by Chiral HPLC analysis (Chiral OD-H column, 10% EtOH in hexanes, flow rate = 1.0 mL/min, UV detection at 225 nm). The *anti*-diastereomer: t_r (minor) = 19.2 min, t_r (major) = 17.1 min, 79% *ee*; the *syn*-diastereomer: t_r (minor) = 23.3 min, t_r (major) = 24.4 min, <5% *ee*.

Racemic sample

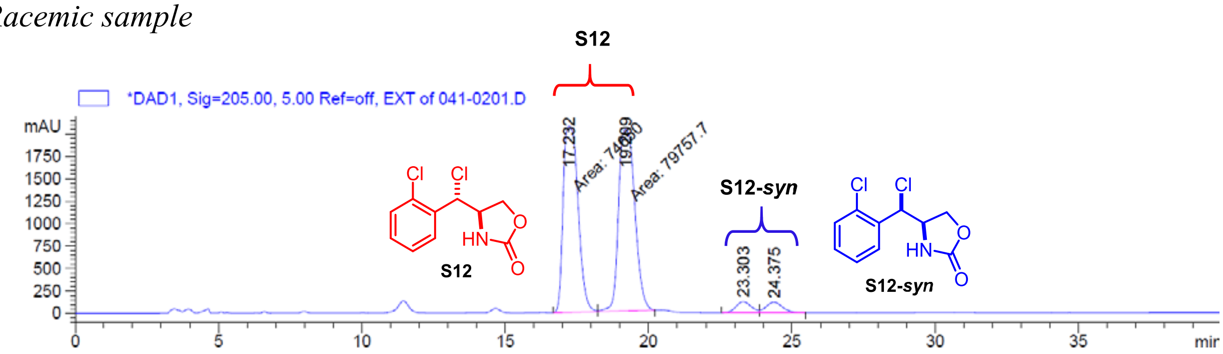

Peak #	RetTime [min]	Type	Width [min]	Area [mAU*s]	Height [mAU]	Area %
1	17.232	MF	0.6003	7.46500e4	2072.44897	45.7309
2	19.209	FM	0.6564	7.97577e4	2024.98523	48.8600
3	23.303	BV	0.5579	4340.15088	119.44707	2.6588
4	24.375	VB	0.5964	4489.55664	114.25739	2.7503

Totals : 1.63237e5 4331.13866

Enantio-enriched sample (79% *ee*): it was obtained by using ***ent*-L5** ligand.

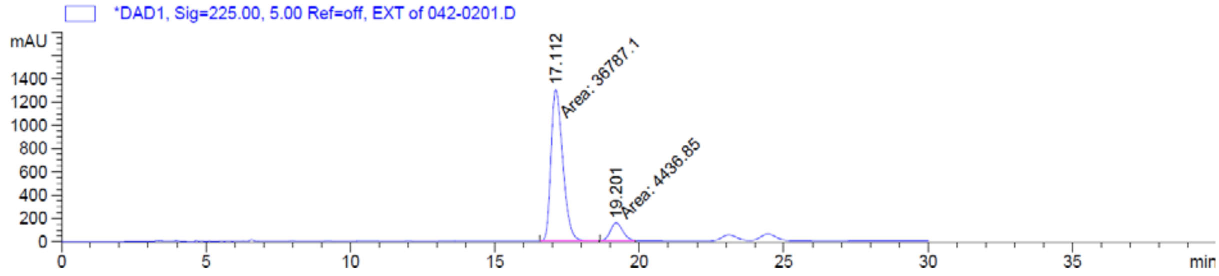

Peak #	RetTime [min]	Type	Width [min]	Area [mAU*s]	Height [mAU]	Area %
1	17.112	MM	0.4713	3.67871e4	1301.02917	89.2372
2	19.201	MM	0.4896	4436.85156	151.04352	10.7628

Totals : 4.12240e4 1452.07269

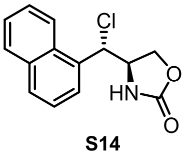

S14

(*R*)-4-((*S*)-chloro(naphthalen-1-yl)methyl)oxazolidin-2-one (**S14**): by following the general procedure, the product **S14** was obtained as a white solid (17 mg, 63% yield, *dr*: 10:1). The *ee* was determined by Chiral HPLC analysis (Chiral *S*, *S*, column, 10% EtOH in hexanes, flow rate = 1.0 mL/min, UV detection at 205 nm). The *anti*-diastereomer: t_r (minor) = 36.8 min, t_r (major) = 33.1 min, 92% *ee*; the *syn*-diastereomer: t_r (minor) = 41.5 min, t_r (major) = 40.5 min, 26% *ee*.

Racemic sample

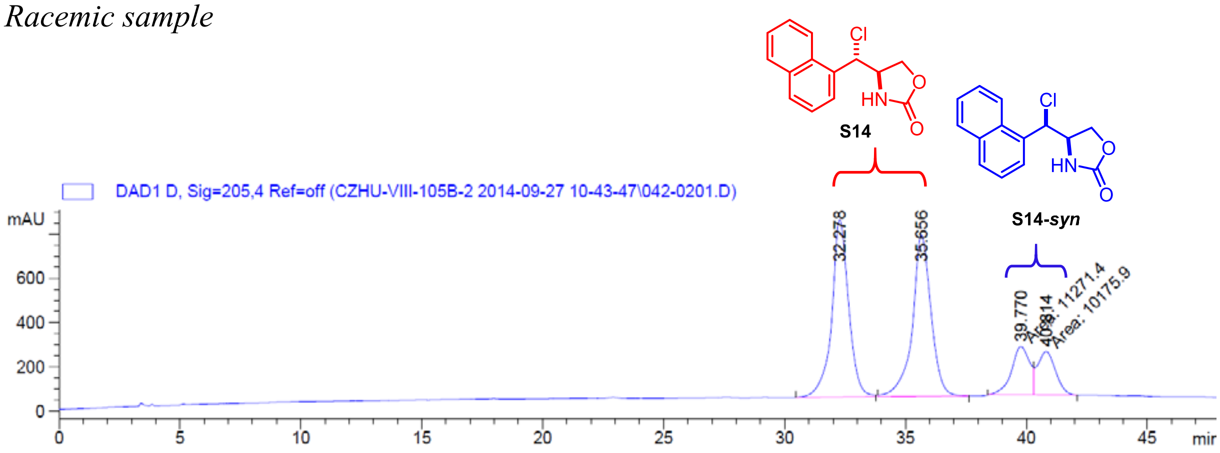

Peak #	RetTime [min]	Type	Width [min]	Area [mAU*s]	Height [mAU]	Area %
1	32.278	BB	0.7245	3.91964e4	801.58344	39.2017
2	35.656	BB	0.7933	3.93426e4	729.40009	39.3480
3	39.770	MF	0.8633	1.12714e4	217.60928	11.2730
4	40.814	FM	0.8658	1.01759e4	195.89568	10.1773

Totals : 9.99863e4 1944.48848

Enantio-enriched sample (92% *ee*)

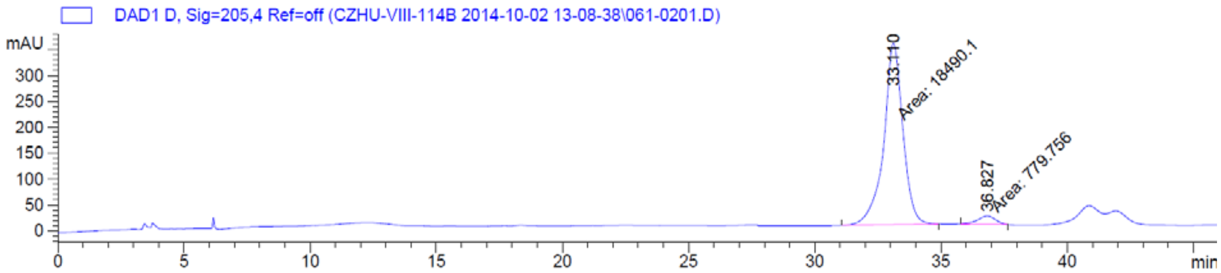

Peak #	RetTime [min]	Type	Width [min]	Area [mAU*s]	Height [mAU]	Area %
1	33.110	MM	0.8787	1.84901e4	350.69656	95.9535
2	36.827	FM	0.8160	779.75604	15.92570	4.0465

Totals : 1.92699e4 366.62226

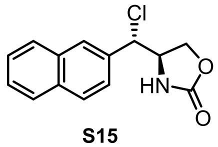

S15

(*R*)-4-((*S*)-chloro(naphthalen-2-yl)methyl)oxazolidin-2-one (**S15**): by following the general procedure under the condition described in entry 8, the product **S15** was obtained as a white solid (14 mg, 53% yield, *dr*: 4.5:1). The *ee* was determined by Chiral HPLC analysis (Chiral OD-H column, 15% EtOH in hexanes, flow rate = 1.0 mL/min, UV detection at 225 nm). The *anti*-diastereomer: t_r (minor) = 21.7 min, t_r (major) = 24.8 min, 89% *ee*; the *syn*-diastereomer: t_r (minor) = 28.5 min, t_r (major) = 32.0 min, <5% *ee*.

Racemic sample

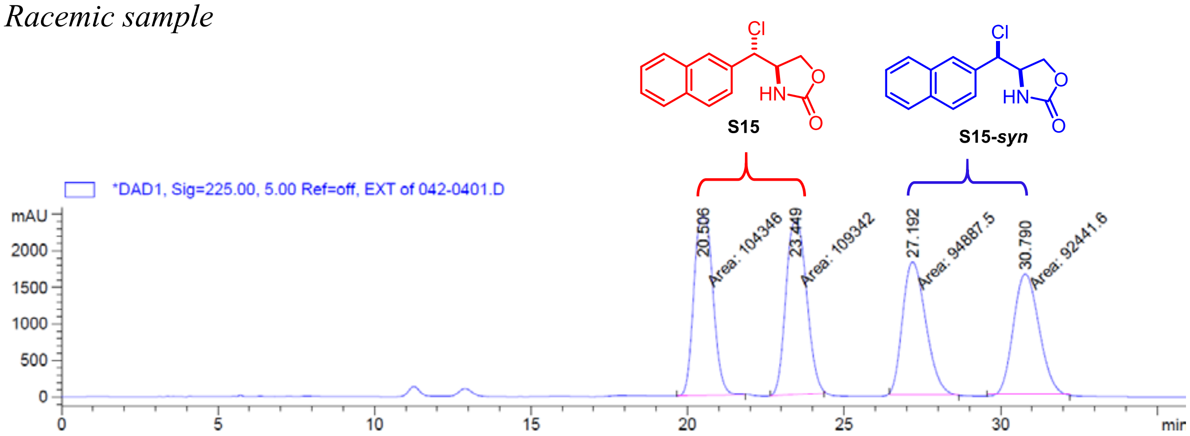

Peak #	RetTime [min]	Type	Width [min]	Area [mAU*s]	Height [mAU]	Area %
1	20.506	MM	0.7091	1.04346e5	2452.68799	26.0204
2	23.449	MM	0.7675	1.09342e5	2374.41357	27.2661
3	27.192	MM	0.8668	9.48875e4	1824.49622	23.6617
4	30.790	MM	0.9385	9.24416e4	1641.62427	23.0518
Totals :				4.01017e5	8293.22205	

Enantio-enriched sample (89% *ee*): it was obtained by using ***ent*-L5** ligand.

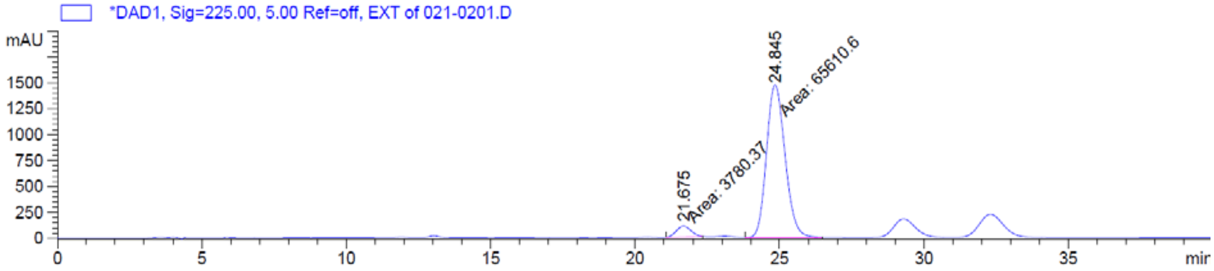

Peak #	RetTime [min]	Type	Width [min]	Area [mAU*s]	Height [mAU]	Area %
1	21.675	MM	0.5975	3780.36719	105.45399	5.4479
2	24.845	MF	0.7444	6.56106e4	1468.95325	94.5521
Totals :				6.93910e4	1574.40724	

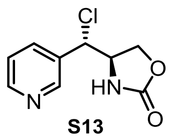

S13

(*R*)-4-((*S*)-chloro(pyridin-3-yl)methyl)oxazolidin-2-one (**S13**): by following the general procedure, the product **S13** and its diastereomer were obtained as a white solid (11 mg, 51% yield, *dr*: 1.8:1). Its *syn*-diastereomer : ^{1}H NMR (400 MHz, $CDCl_3$) δ 8.62 (d, *J* = 22.0 Hz, 2H), 7.74 (d, *J* = 8.0 Hz, 1H), 7.35 (t, *J* = 6.2 Hz, 1H), 6.04 (s, 1H), 4.88 (d, *J* = 7.6 Hz, 1H), 4.38 (td, *J* = 8.4, 4.2 Hz, 1H), 4.31 (dd, *J* = 8.4, 8.4 Hz, 1H), 4.06 (dd, *J* = 8.6, 4.0 Hz, 1H). ^{13}C NMR (100 MHz, $CDCl_3$) δ 158.3, 150.9, 148.8, 135.3, 132.6, 124.1, 66.7, 62.1, 58.5. The *ee* was determined by Chiral HPLC analysis (Chiral AS-H column, 25% EtOH in hexanes, flow rate = 1.0 mL/min, UV detection at 205 nm). The *anti*-diastereomer: t_r (minor) = 42.6 min, t_r (major) = 34.0 min, 70% *ee*; the *syn*-diastereomer: t_r (minor) = 23.1 min, t_r (major) = 25.9 min, <5% *ee*.

Racemic sample

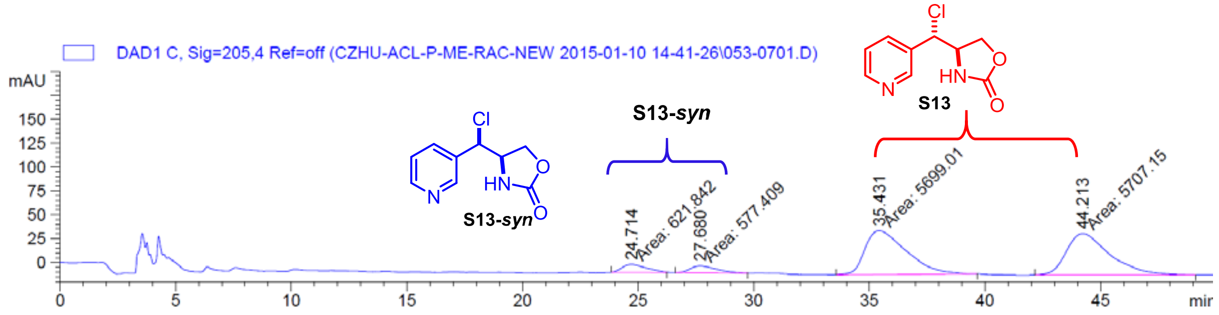

Peak #	RetTime [min]	Type	Width [min]	Area [mAU*s]	Height [mAU]	Area %
1	24.714	MM	1.2220	621.84192	8.48125	4.9331
2	27.680	MM	1.3079	577.40948	7.35821	4.5806
3	35.431	MM	2.0750	5699.00635	45.77486	45.2108
4	44.213	MM	2.1854	5707.15283	43.52435	45.2754
Totals :				1.26054e4	105.13867	

Enantio-enriched sample (70% *ee*): it was obtained by using ***ent*-L5** ligand.

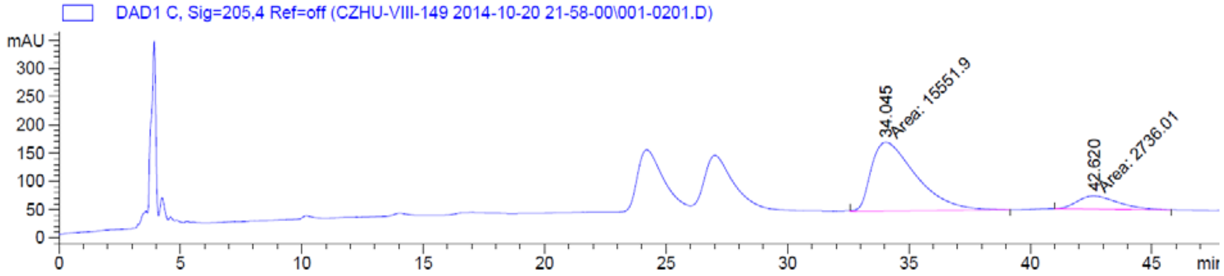

Peak #	RetTime [min]	Type	Width [min]	Area [mAU*s]	Height [mAU]	Area %
1	34.045	FM	2.1449	1.55519e4	120.84544	85.0392
2	42.620	MM	1.9542	2736.00684	23.33451	14.9608
Totals :				1.82879e4	144.17995	

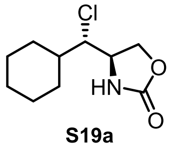

S19a

(*R*)-4-((*S*)-chloro(o-tolyl)methyl)oxazolidin-2-one (**S19a**): by following the general procedure, the product **S19a** and its diastereomer were obtained as a white solid (18 mg, 78% yield, *dr*: 2.0:1). The *ee* was determined by Chiral HPLC analysis after benzoylation (Chiral OD-H column, 10% isopropanol in hexanes, flow rate = 1.0 mL/min, UV detection at 254 nm). The *anti*-diastereomer: t_r (minor) = 26.7 min, t_r (major) = 24.9 min, 54% *ee*.

Racemic sample of the ***S19c***

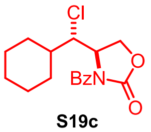

S19c

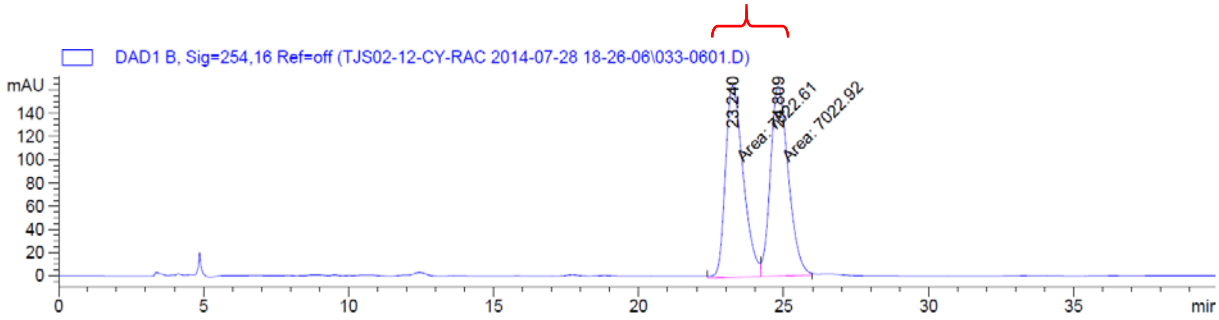

Peak #	RetTime [min]	Type	Width [min]	Area [mAU*s]	Height [mAU]	Area %
1	23.240	MF	0.7101	7022.60693	164.81929	49.9989
2	24.809	FM	0.7173	7022.91943	163.17218	50.0011

Totals : 1.40455e4 327.99147

Enantio-enriched sample of ***S19c*** (54% *ee*)

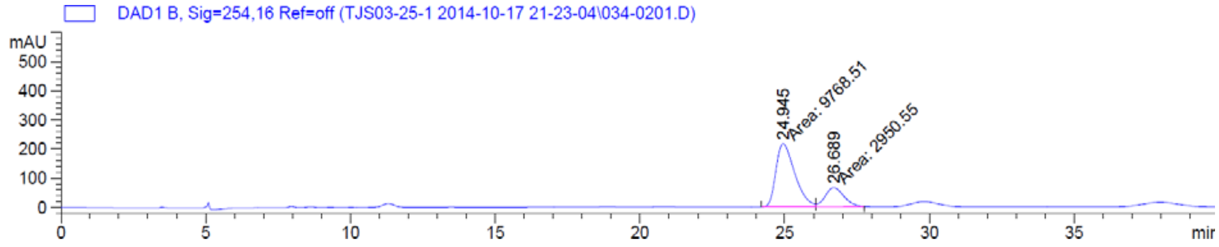

Peak #	RetTime [min]	Type	Width [min]	Area [mAU*s]	Height [mAU]	Area %
1	24.945	MF	0.7546	9768.51172	215.75325	76.8022
2	26.689	FM	0.7509	2950.54517	65.48528	23.1978

Totals : 1.27191e4 281.23853

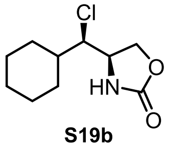

S19b

(*R*)-4-((*R*)-chloro(o-tolyl)methyl)oxazolidin-2-one (**S19b**, separable from **S19a**): ^{1}H NMR (400 MHz, $CDCl_3$): δ 6.04 (s, 1H), 4.55 – 4.39 (m, 1H), 4.21 (q, J = 5.4, 5.0 Hz, 2H), 3.73 (t, J = 5.6 Hz, 1H), 1.86–1.71 (m, 3H), 1.72–1.51 (m, 3H), 1.24 (dddd, J = 31.7, 20.2, 8.0, 3.2 Hz, 5H); ^{13}C NMR (100 MHz, $CDCl_3$): δ 158.9, 70.5, 67.6, 55.0, 40.6, 30.7, 27.9, 25.9, 25.8, 25.7; IR ν_{max} (neat)/cm^{-1}: 2920 (s), 2857(s), 1765 (s), 1434 (w), 1352 (m), 1245(s), 1130(s), 1030 (s), 824 (s); HRMS (ESI, *m/z*): calcd for $C_{10}H_{17}O_2NCl^+$ (M + H$^+$), 218.0942, found 218.0937. The *ee* was determined by Chiral HPLC analysis after benzoylation (Chiral OD-H column, 10% EtOH in hexanes, flow rate = 1.0 mL/min, UV detection at 254 nm). The *anti*-diastereomer: t_r (minor) = 12.0 min, t_r (major) = 21.3 min, 10% *ee*.

*Racemic sample of the **S19d***

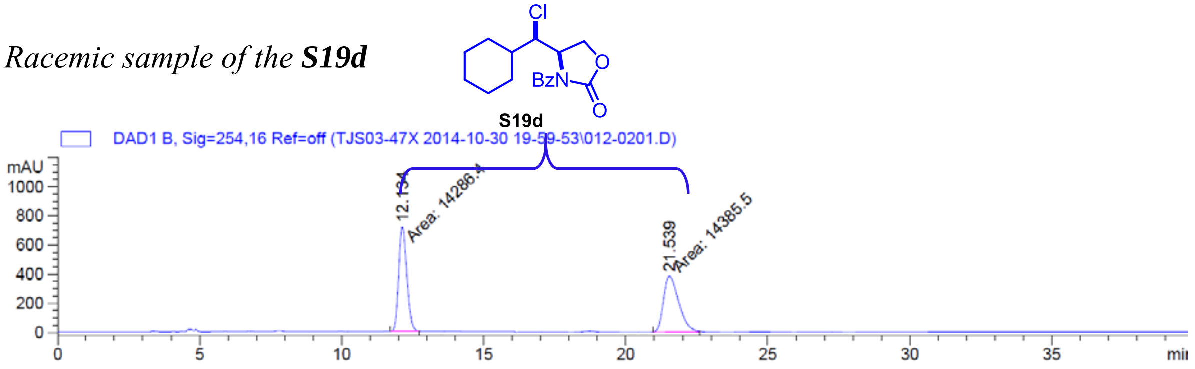

Peak #	RetTime [min]	Type	Width [min]	Area [mAU*s]	Height [mAU]	Area %
1	12.134	MM	0.3313	1.42864e4	718.77948	49.8271
2	21.539	MM	0.6226	1.43855e4	385.07678	50.1729

Totals : 2.86719e4 1103.85626

*Enantio-enriched sample of **S19d** (10% ee)*

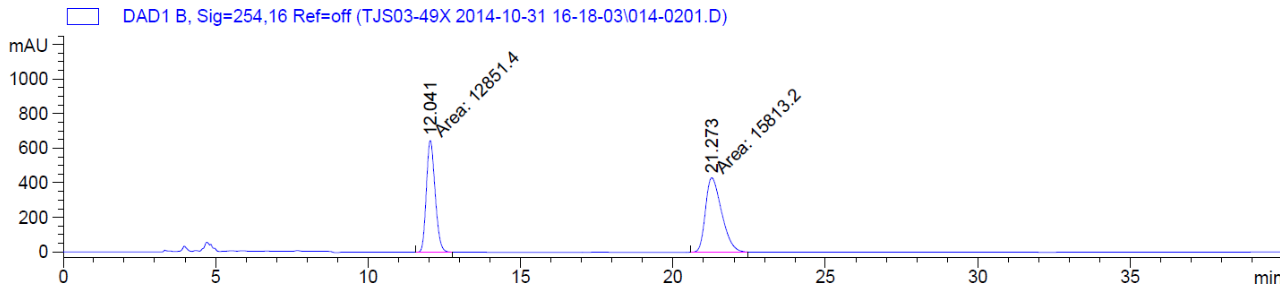

Peak #	RetTime [min]	Type	Width [min]	Area [mAU*s]	Height [mAU]	Area %
1	12.041	MM	0.3325	1.28972e4	646.45203	44.8627
2	21.273	MM	0.6148	1.58510e4	429.67621	55.1373
Totals :				2.87482e4	1076.12823	

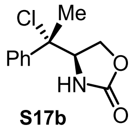

S17b

(*R*)-4-((*S*)-1-chloro-1-phenylethyl)oxazolidin-2-one (S17b): by following the general procedure with ligand **L6** and carrying out reaction at -40 °C, the product **S17b** and its *syn*-diastereomer **S17a** were obtained as a white solid (10 mg, 45% yield, *dr*: 2.3:1). ^{1}H NMR (400 MHz, $CDCl_3$): δ 7.57–7.49 (m, 2H), 7.44–7.32 (m, 3H), 4.41 (dd, *J* = 9.0, 4.3 Hz, 1H), 4.26 (t, *J* = 9.2 Hz, 1H), 4.09 (dd, *J* = 9.6, 4.3 Hz, 1H), 1.97 (s, 3H); ^{13}C NMR (100 MHz, $CDCl_3$): δ 158.5, 140.1, 129.0, 128.9, 126.5, 73.6, 66.2, 62.6, 29.7, 24.3; IR ν_{max} (neat)/cm^{-1}: 3260 (m), 3136 (w), 2984 (w), 2921 (w), 1749(s), 1040 (w), 1236 (m), 1046 (m), 701 (m); HRMS (ESI, *m/z*): calcd for $C_{11}H_{13}NO_2Cl^+$ (M + H^+), 226.0635, found 226.0640.The *ee* was determined by Chiral HPLC analysis (Chiral OD-H column, 10% EtOH in hexanes, flow rate = 1.0 mL/min, UV detection at 205 nm). The *anti*-diastereomer: t_r (minor) = 18.3 min, t_r (major) = 25.8 min, 86% *ee*; the *syn*-diastereomer: t_r (minor) = 23.0 min, t_r (major) = 20.8 min, 50% *ee*.

Racemic sample

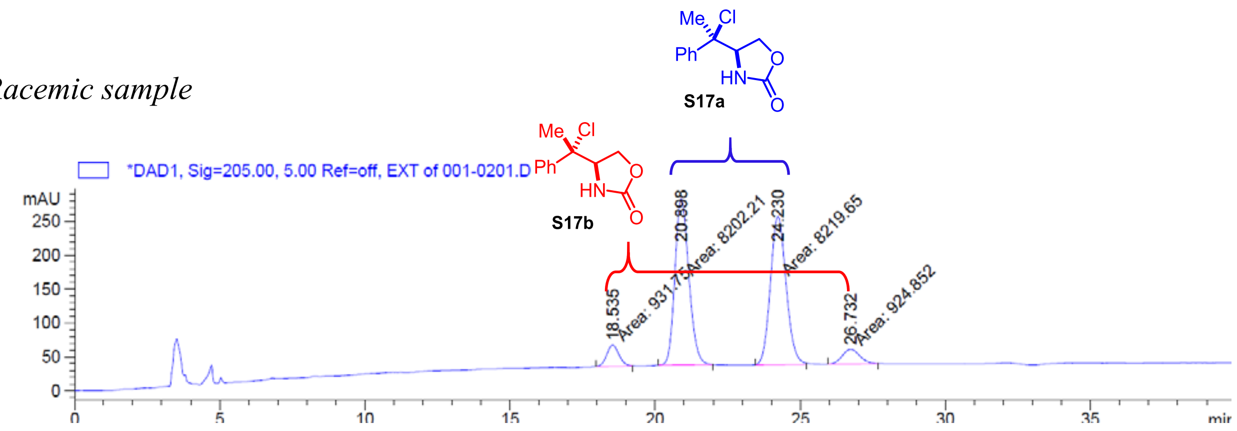

Peak #	RetTime [min]	Type	Width [min]	Area [mAU*s]	Height [mAU]	Area %
1	18.535	MM	0.4920	931.75006	31.56405	5.0975
2	20.898	MM	0.5573	8202.20996	245.29901	44.8736
3	24.230	MM	0.6271	8219.65137	218.46324	44.9691
4	26.732	MM	0.7016	924.85156	21.97011	5.0598

Totals : 1.82785e4 517.29642

Enantio-enriched sample **S17b** (86% *ee*): it was obtained by using **L6** ligand.

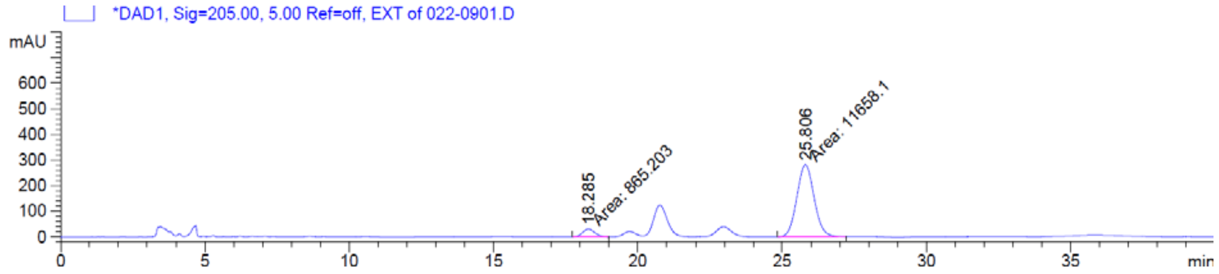

Peak #	RetTime [min]	Type	Width [min]	Area [mAU*s]	Height [mAU]	Area %
1	18.285	MM	0.4802	865.20325	30.02811	6.9087
2	25.806	MM	0.6897	1.16581e4	281.70096	93.0913

Totals : 1.25233e4 311.72907

Enantio-enriched sample **S17a** (50 % *ee*): it was obtained by using **L6** ligand.

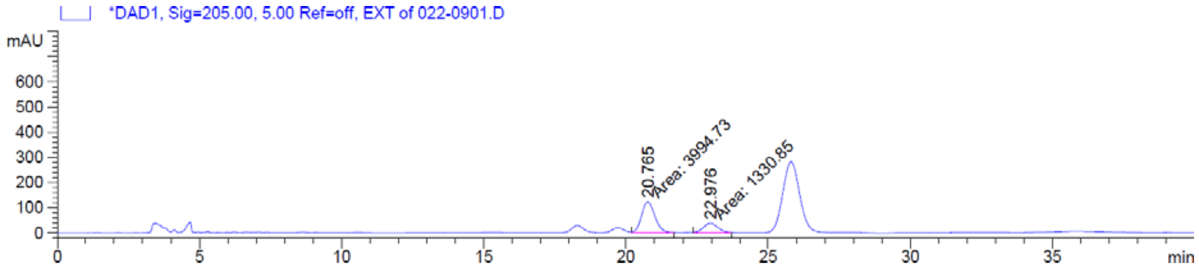

Peak #	RetTime [min]	Type	Width [min]	Area [mAU*s]	Height [mAU]	Area %
1	20.765	FM	0.5415	3994.73242	122.94210	75.0102
2	22.976	MM	0.5796	1330.85083	38.26603	24.9898

Totals :				5325.58325	161.20813	

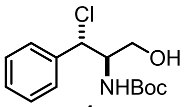
4

***tert*-Butyl ((1*S*,2*R*)-1-chloro-3-hydroxy-1-phenylpropan-2-yl)carbamate** (**4**): by following a literature procedure, **4** was obtained from **2a** (87% yield for two steps).[2] At room temperature, to a round bottom flask equipped with a magnetic stir bar were added **2a** (0.2 mmol, 1.0 equiv), anhydrous CH_2Cl_2 (3 mL), Et_3N (0.24 mmol, 1.2 equiv), DMAP (0.02 mmol, 0.1 equiv) and Boc_2O (0.3 mmol, 1.5 equiv). The reaction mixture was then stirred at room temperature until **2a** was fully consumed monitored by TLC. After evaporating the solvent, the residue was purified through gradient silica gel flash column chromatography (hexanes/EtOAc: from 6:1 to 2:1) to afford the *N*-Boc-protected intermediate (61 mg, 99% yield). The obtained *N*-Boc-protected intermediate (0.2 mmol, 1.0 equiv) was dissolved in MeOH (2 mL); Cs_2CO_3 (0.02 mmol) was added and the mixture was stirred at room temperature until all the starting material was consumed. The reaction was quenched with saturated NH_4Cl solution. After removal of MeOH, the aqueous phase was extracted with CH_2Cl_2 (4 × 5 mL) and then dried over anhydrous Na_2SO_4. After evaporating the solvent, the residue was purified through gradient silica gel flash column chromatography (hexanes/EtOAc: from 6:1 to 3:1) to afford chloro amino alcohol **4** (51 mg, 88% yield). ^{1}H NMR (400 MHz, $CDCl_3$) δ 7.43 (d, *J* = 7.2 Hz, 2H), 7.40–7.27 (m, 3H), 5.15 (d, *J* = 5.5 Hz, 1H), 4.96 (d, *J* = 8.8 Hz, 1H), 4.15–4.09 (m, 1H), 4.04 (dd, *J* = 11.3, 4.7 Hz, 1H), 3.75 (dd, *J* = 11.3, 3.6 Hz, 1H), 2.04 (s, 1H), 1.32 (s, 9H); ^{13}C NMR (100 MHz, $CDCl_3$) δ 155.4, 138.0, 128.5, 128.5, 127.9, 80.0, 62.2, 61.9, 57.6, 28.2; IR ν_{max} (neat)/cm^{-1}: 3316 (br), 2977 (m), 2970 (w), 1744 (s), 1691(s), 1498 (m), 1455 (m), 1392 (m), 1367 (m), 1250 (m), 1168 (s), 1052 (m), 698 (m); HRMS (ESI, *m/z*): calcd for $C_{14}H_{21}NO_3Cl^+$ (M + H^+), 286.1192, found 286.1195.

The enantio-enriched **4** was obtained from enantio-enriched **2a** (obtained by using ***ent*-L5** ligand) by following the above procedure. (*dr*: 15:1, 85% yield over two steps). $[\alpha]_D^{20}$ = -31 ° (*c* 1.0, CH_2Cl_2). The *ee* was determined by Chiral HPLC analysis (Chiral OD-H column, 5% EtOH in hexanes, flow rate = 1.0 mL/min, UV detection at 215 nm): t_r (minor) = 9.9 min, t_r (major) = 7.1 min, 88% *ee*.

*Racemic sample of **4** (dr: 1:1)*

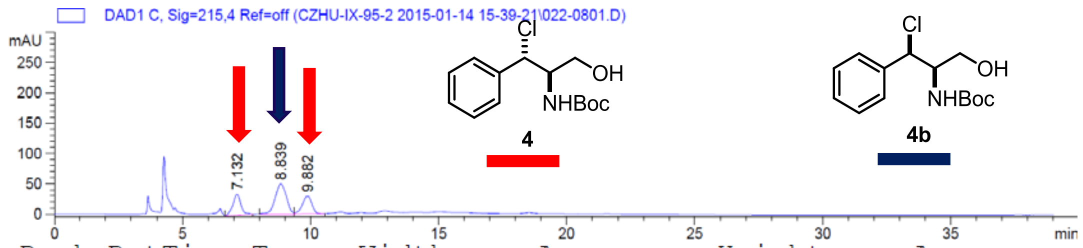

Peak #	RetTime [min]	Type	Width [min]	Area [mAU*s]	Height [mAU]	Area %
1	7.132	VB	0.3206	758.65222	34.76191	25.0030
2	8.839	BV	0.5054	1514.52441	49.99388	49.9144
3	9.882	VB	0.4009	761.06787	29.70838	25.0826
Totals :				3034.24451	114.46416	

*Racemic sample of **4** (dr >20:1)*

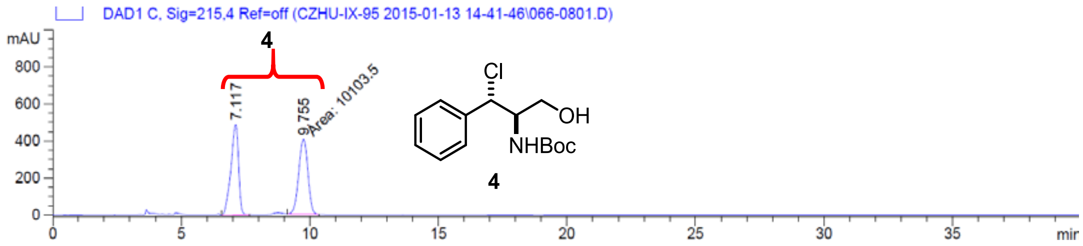

Peak #	RetTime [min]	Type	Width [min]	Area [mAU*s]	Height [mAU]	Area %
1	7.117	VB	0.3129	1.01061e4	489.47833	50.0064
2	9.755	MM	0.4151	1.01035e4	405.66406	49.9936
Totals :				2.02095e4	895.14240	

*Enantio-enriched sample of **4** (dr: 15:1, 88% ee.)*

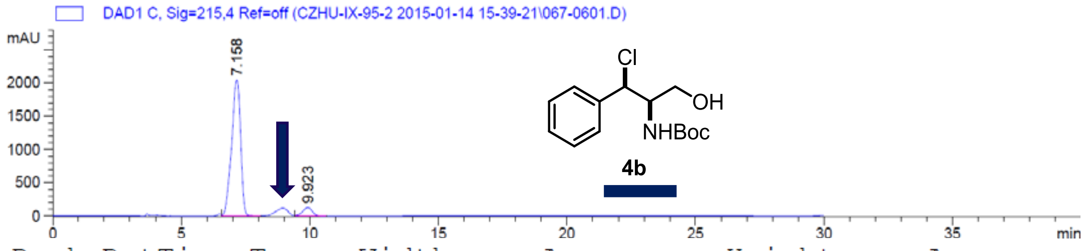

Peak #	RetTime [min]	Type	Width [min]	Area [mAU*s]	Height [mAU]	Area %
1	7.158	VB	0.3712	5.00335e4	2047.01538	93.9525
2	9.923	VB	0.4086	3220.56372	124.99698	6.0475

Totals : 5.32541e4 2172.01236

D. Mechanistic Investigation of the Iron-Catalyzed Asymmetric Olefin Aminochlorination

a. Fe(NTf$_2$)$_2$-Catalyzed Asymmetric Aminochlorination and Aminohydroxylation with Isomeric Olefins

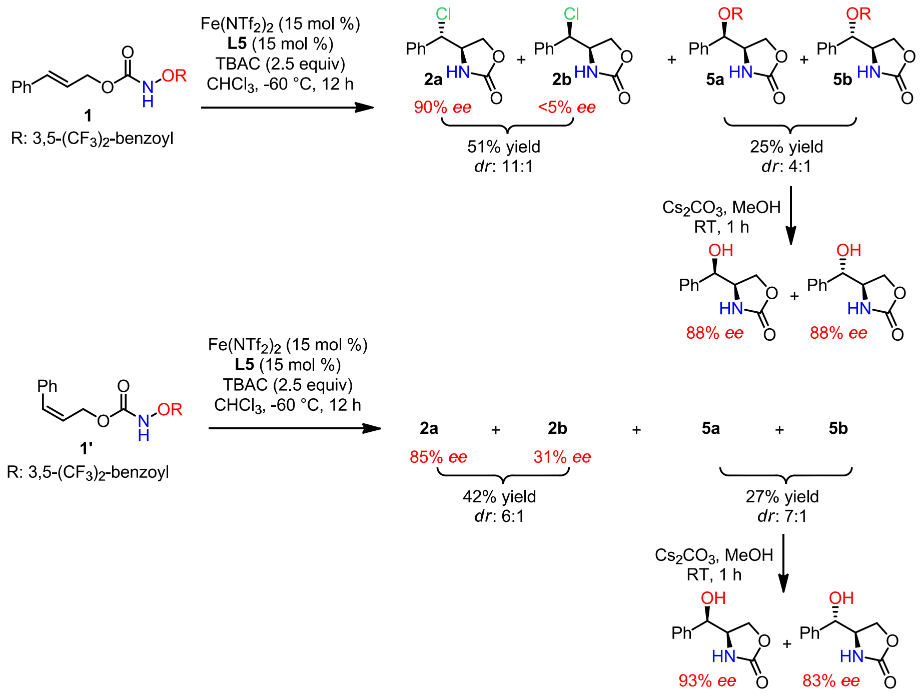

Procedure. To a flame-dried sealable 2-dram vial (vial **A**) equipped with a magnetic stir bar were added Fe(NTf$_2$)$_2$ (9.2 mg, 0.015 mmol, 15 mol %) and ligand **L5** (7.3 mg, 0.015 mmol, 15 mol %). After the vial was evacuated and backfilled with N_2 for three times, $CHCl_3$ (1.0 mL, re-distilled and anhydrous) was added and the mixture was stirred at room temperature for 20 min. Meanwhile, a second flame-dried and N_2-protected 2-dram vial (vial **B**) with a magnetic stir bar was charged with the substrate (**1** or **1′**, 0.1 mmol), anhydrous TBAC (69.5 mg, 0.25 mmol), freshly activated 4 Å molecular sieves and $CHCl_3$ (3.0 mL, re-distilled and anhydrous). Both vials were degassed by brief evacuation and back filling with N_2 twice. Vial **B** was cooled down to -60 ^{o}C, and the catalyst solution in vial **A** was added to vial **B** drop wise via a syringe. The resulting solution was stirred at this temperature for 12 h and then quenched with 1 mL saturated $NaHCO_3$ solution. The reaction mixture was extracted with CH_2Cl_2 (1.5 mL × 3), and the combined organic phase was concentrated *in vacuo*. The residue was purified through a gradient

silica gel flash column chromatography (hexanes/acetone: from 15:1 to 4:1) to afford both the aminochlorination product **2a/b** and the aminohydroxylation product **5a/b**. The *dr* was determined by ^{1}H NMR analysis and the *ee* of **2a/b** was directly measured by chiral HPLC analysis. The *ee* of **5a/b** was measured after the hydrolysis.[1]

b. Rate Acceleration Effect of External Chloride Ion

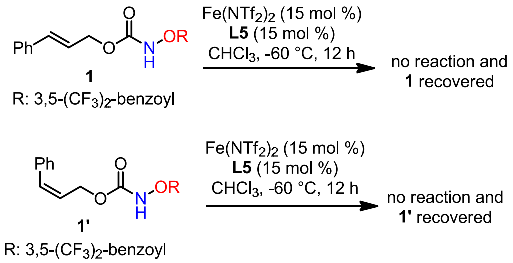

Procedure. These experiments were carried out under the condition described above, except in the absence of TBAC. Under this condition, both **1** and **1′** were fully recovered.

c. $FeCl_2$-Catalyzed and Mediated Asymmetric Olefin Aminochlorination Reactions

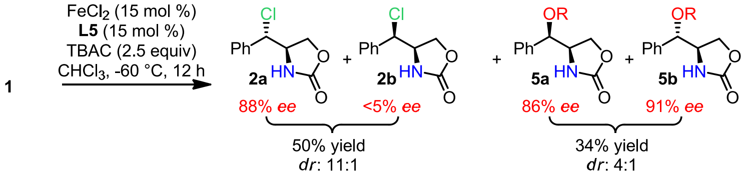

Procedure. To a flame-dried sealable 2-dram vial (vial **A**) equipped with a magnetic stir bar were added $FeCl_2$ (0.015 mmol, 15 mol %) and ligand **L5** (7.3 mg, 0.015 mmol, 15 mol %). After the vial was evacuated and backfilled with N_2 for three times, $CHCl_3$ (1.0 mL, re-distilled and anhydrous) was added and the mixture was stirred at room temperature for 20 min. Meanwhile, a second flame-dried and N_2-protected 2-dram vial (vial **B**) with a magnetic stir bar was charged with **1** (0.1 mmol), anhydrous TBAC (69.5 mg, 0.25 mmol), freshly activated 4 Å molecular sieves and $CHCl_3$ (3.0 mL, re-distilled and anhydrous). Both vials were degassed by brief evacuation and back filling with N_2 twice. Vial **B** was cooled down to -60 ^{o}C, and the catalyst solution in vial **A** was added to vial **B** drop wise via a syringe. The resulting solution

was stirred at this temperature for 12 h and then quenched with 1 mL saturated $NaHCO_3$ solution. The reaction mixture was extracted with CH_2Cl_2 (1.5 mL × 3), and the combined organic phase was concentrated *in vacuo*. The residue was purified through a gradient silica gel flash column chromatography (hexanes/acetone: from 15:1 to 4:1) to afford both the aminochlorination product **2a/b** and the aminohydroxylation product **5a/b**. The *dr* was determined by ^{1}H NMR analysis and the *ee* of **2a/b** was directly measured by chiral HPLC analysis. The *ee* of **5a/b** was measured after the hydrolysis.

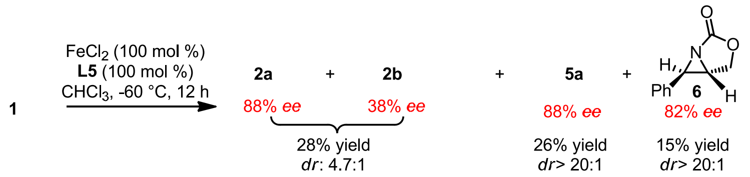

Procedure. To a flame-dried sealable 2-dram vial (vial **A**) equipped with a magnetic stir bar were added $FeCl_2$ (0.1 mmol, 100 mol %) and ligand **L5** (0.1 mmol, 100 mol %). After the vial was evacuated and backfilled with N_2 for three times, $CHCl_3$ (1.0 mL, re-distilled and anhydrous) was added and the mixture was stirred at room temperature for 20 min. Meanwhile, a second flame-dried and N_2-protected 2-dram vial (vial **B**) with a magnetic stir bar was charged with **1** (0.1 mmol), freshly activated 4 Å molecular sieves and $CHCl_3$ (3.0 mL, re-distilled and anhydrous). Both vials were degassed by brief evacuation and back filling with N_2 twice. Vial **B** was cooled down to -60 °C, and the catalyst solution in vial **A** was added to vial **B** drop wise via a syringe. The resulting solution was stirred at this temperature for 12 h and then quenched with 1 mL saturated $NaHCO_3$ solution. The reaction mixture was extracted with CH_2Cl_2 (1.5 mL × 3), and the combined organic phase was concentrated *in vacuo*. The residue was purified through a gradient silica gel flash column chromatography (hexanes/acetone: from 15:1 to 4:1) to afford both the aminochlorination product **2a/b**, the aminohydroxylation product **5a**, and the aziridine **6**. The *dr* was determined by ^{1}H NMR analysis and the *ee* of **2a/b** and **6** was directly measured by chiral HPLC analysis.[1] The *ee* of **5a** was measured after the hydrolysis.

E. References

1. G.-S. Liu, Y.-Q. Zhang, Y.-A. Yuan and H. Xu, *J. Am. Chem. Soc.*, 2013, **135**, 3343.

2. D.-F. Lu, G.-S. Liu, C.-L. Zhu, B. Yuan and H. Xu, *Org. Lett.*, 2014, **16**, 2912.

3. D.-F. Lu, C.-L. Zhu, Z.-X. Jia and H. Xu, *J. Am. Chem. Soc.*, 2014, **136**, 13186.

4. T. Bach, B. Schlummer and K. Harms, *Chem.-Eur. J.*, 2001, **7**, 2581.

5. E. G. Gutierrez, C. J. Wong, A. H. Sahin and A. K. Franz, *Org. Lett.*, 2011, **13**, 5754.

6. G. Desimoni, G. Faita, M. Guala and C. Pratelli, *Tetrahedron Asymmetr.*, 2002, **13**, 1651.

7. A. Cornejo, J. M. Fraile, J. I. García, M. J. Gil, V. Martínez-Merino, J. A. Mayoral, E. Pires and I. Villalba, *Synlett*, 2005, 2321.

F. NMR Spectra

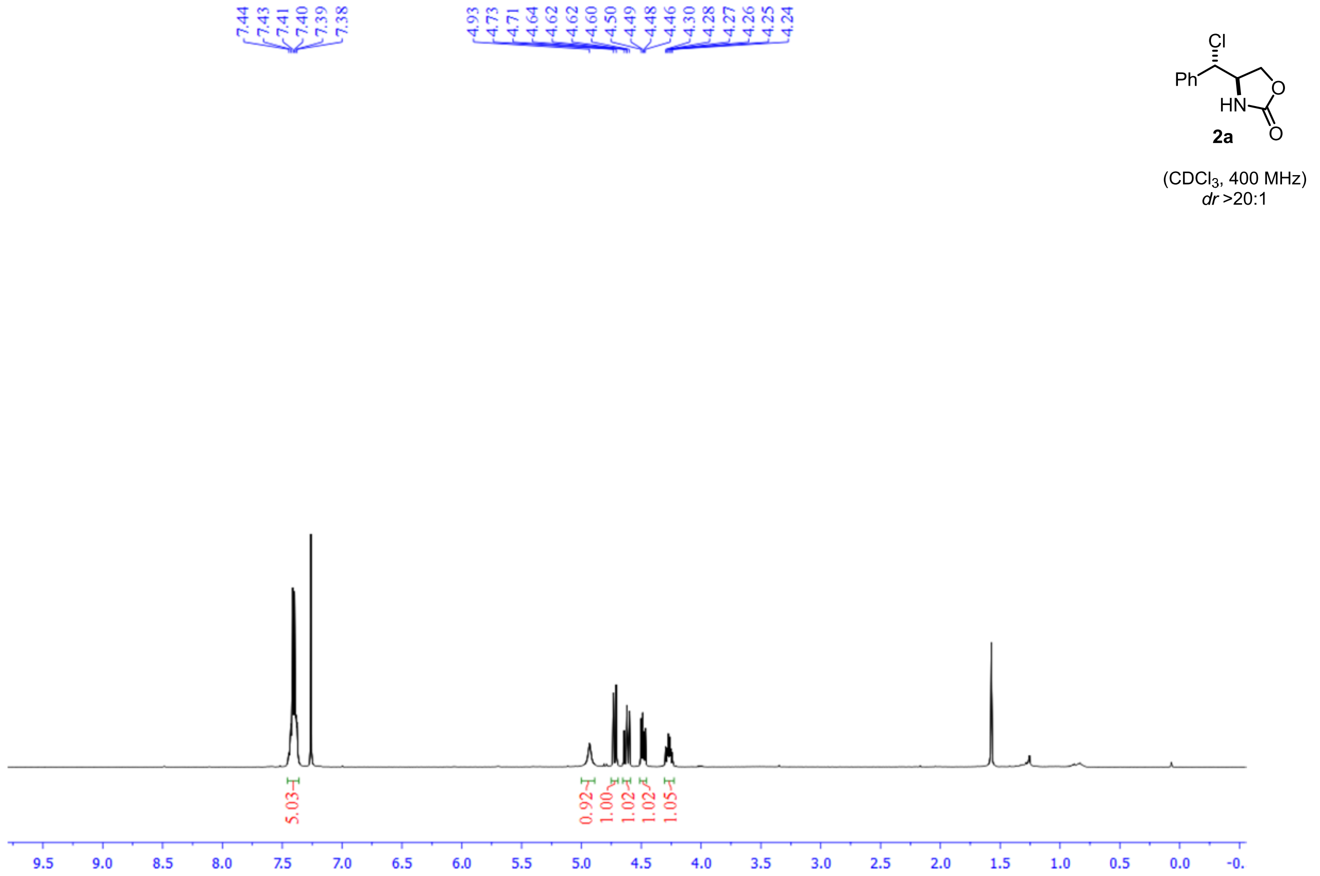

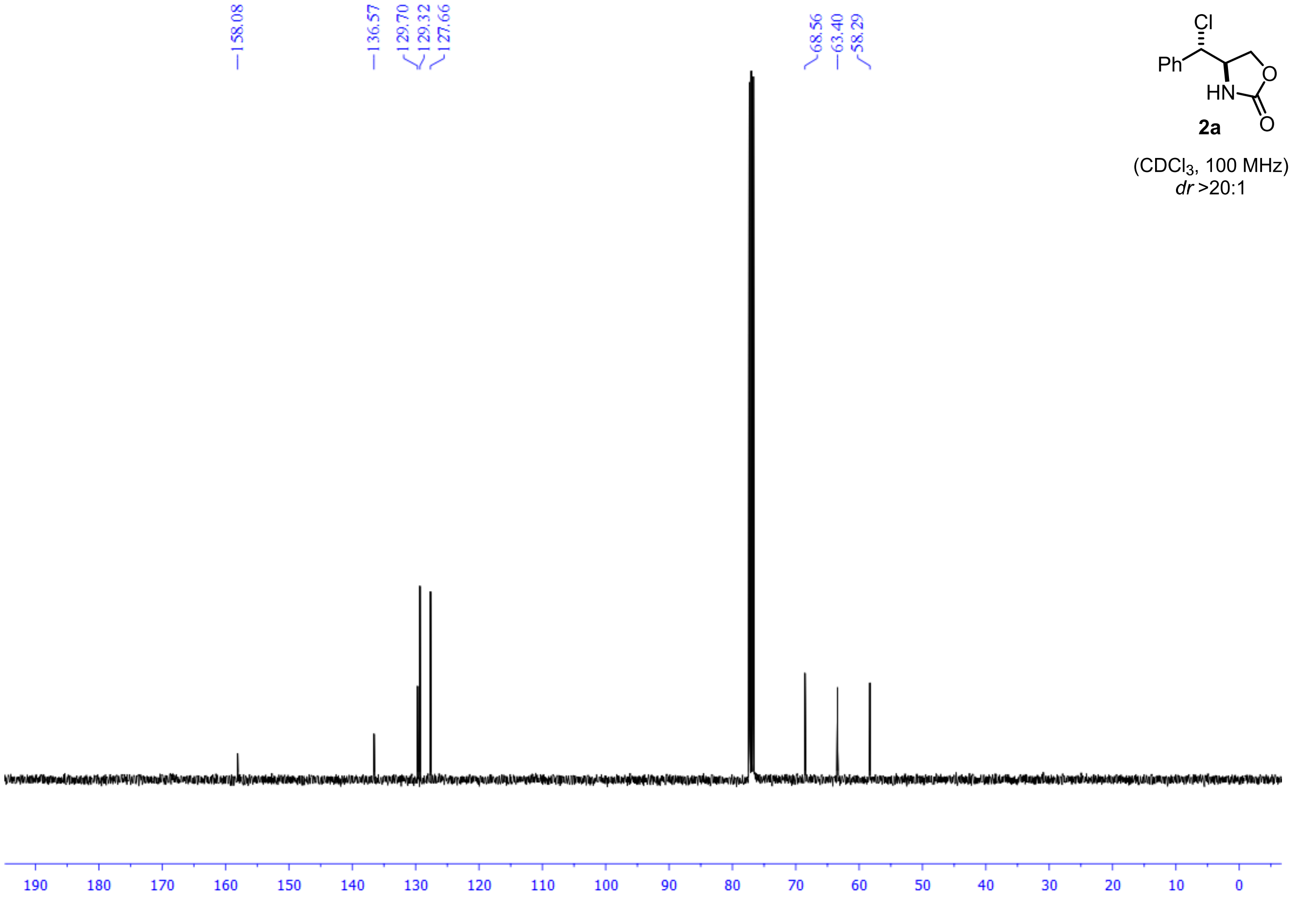

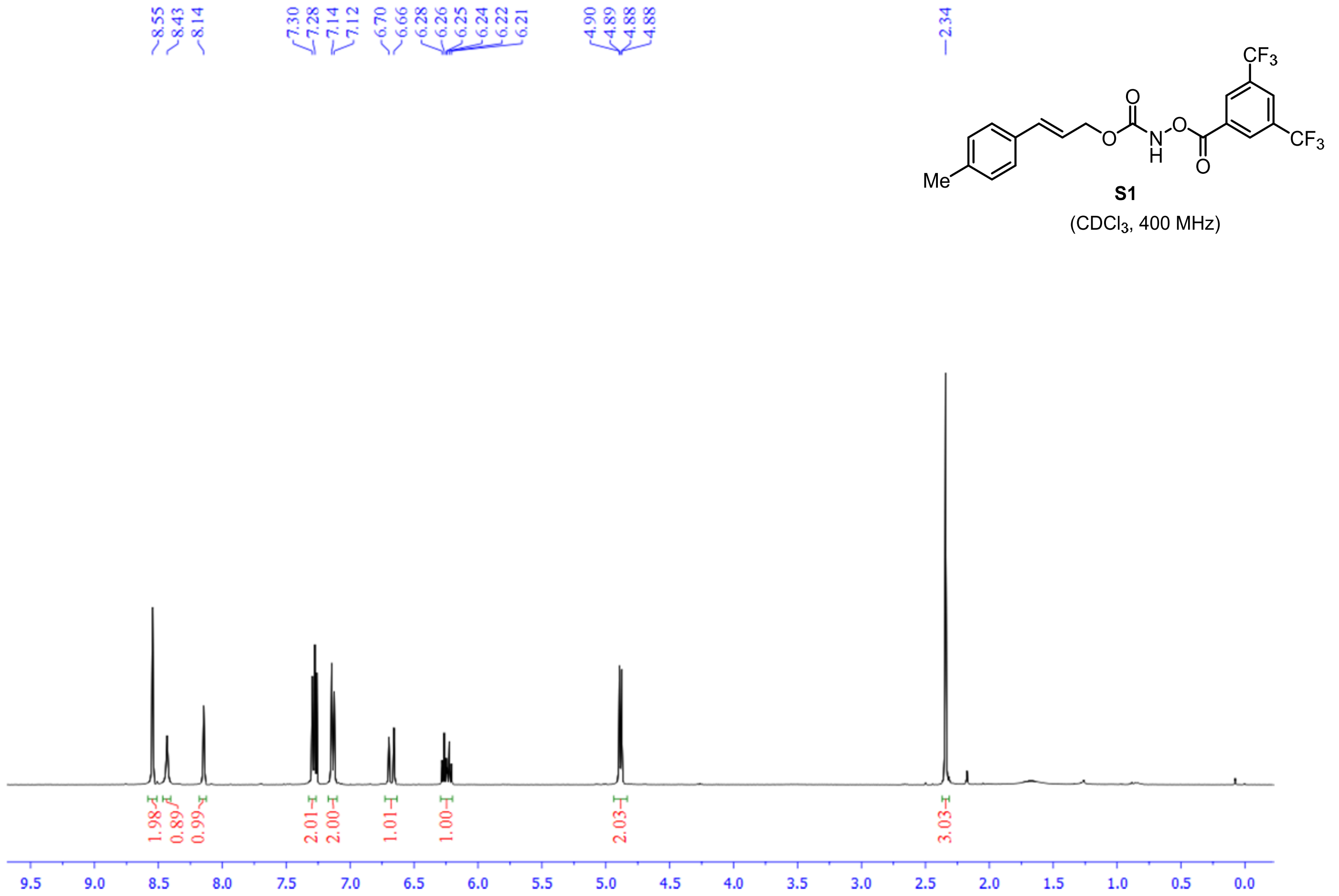
8.55 8.43 8.14 7.30 7.28 7.14 7.12 6.70 6.66 6.28 6.26 6.25 6.24 6.22 6.21 4.90 4.89 4.88 4.88 2.34
CF3 CF3 O O O N H O O Me
S1
(CDCl3, 400 MHz)
1.98 0.89 0.99 2.01 2.00 1.01 1.00 2.03 3.03
9.5 9.0 8.5 8.0 7.5 7.0 6.5 6.0 5.5 5.0 4.5 4.0 3.5 3.0 2.5 2.0 1.5 1.0 0.5 0.0

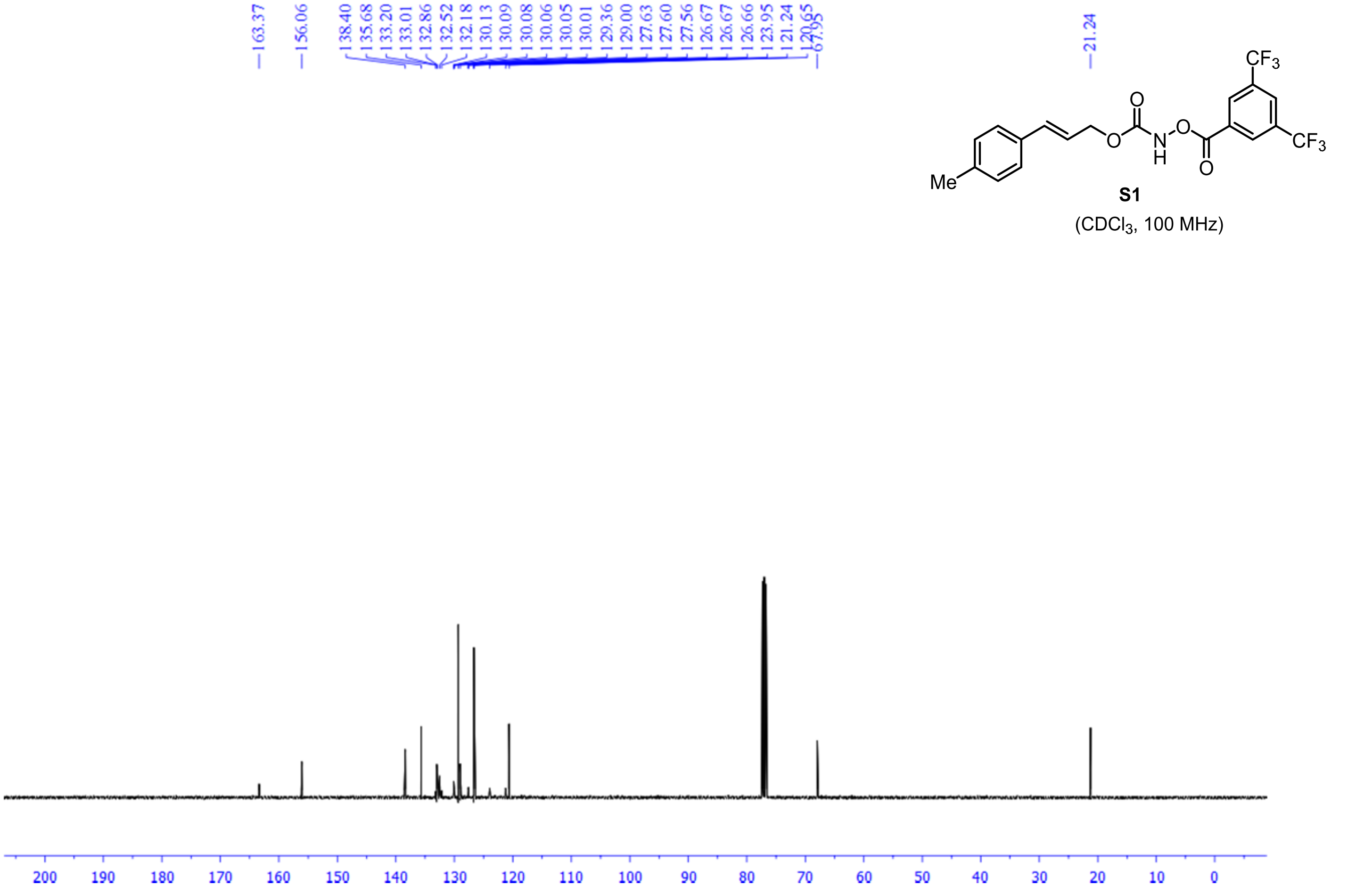
163.37 156.06 138.40 135.68 133.20 133.01 132.86 132.52 132.18 130.13 130.09 130.08 130.06 130.05 130.01 129.36 129.00 127.63 127.60 127.56 126.67 126.67 126.66 123.95 121.24 120.65 67.95 21.24
CF3 CF3 O O O N H O O Me
S1
(CDCl3, 100 MHz)
200 190 180 170 160 150 140 130 120 110 100 90 80 70 60 50 40 30 20 10 0

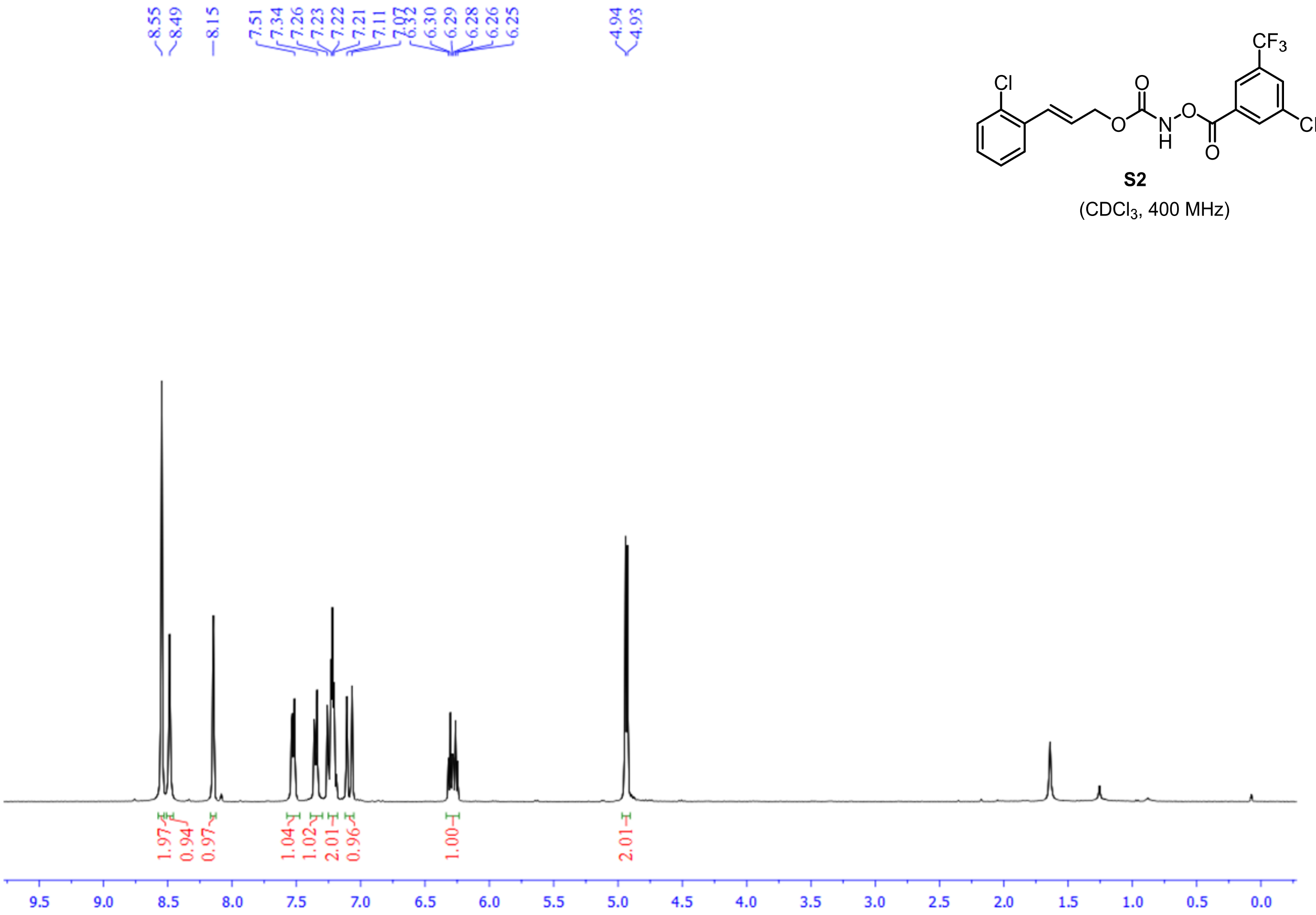

S2

($CDCl_3$, 400 MHz)

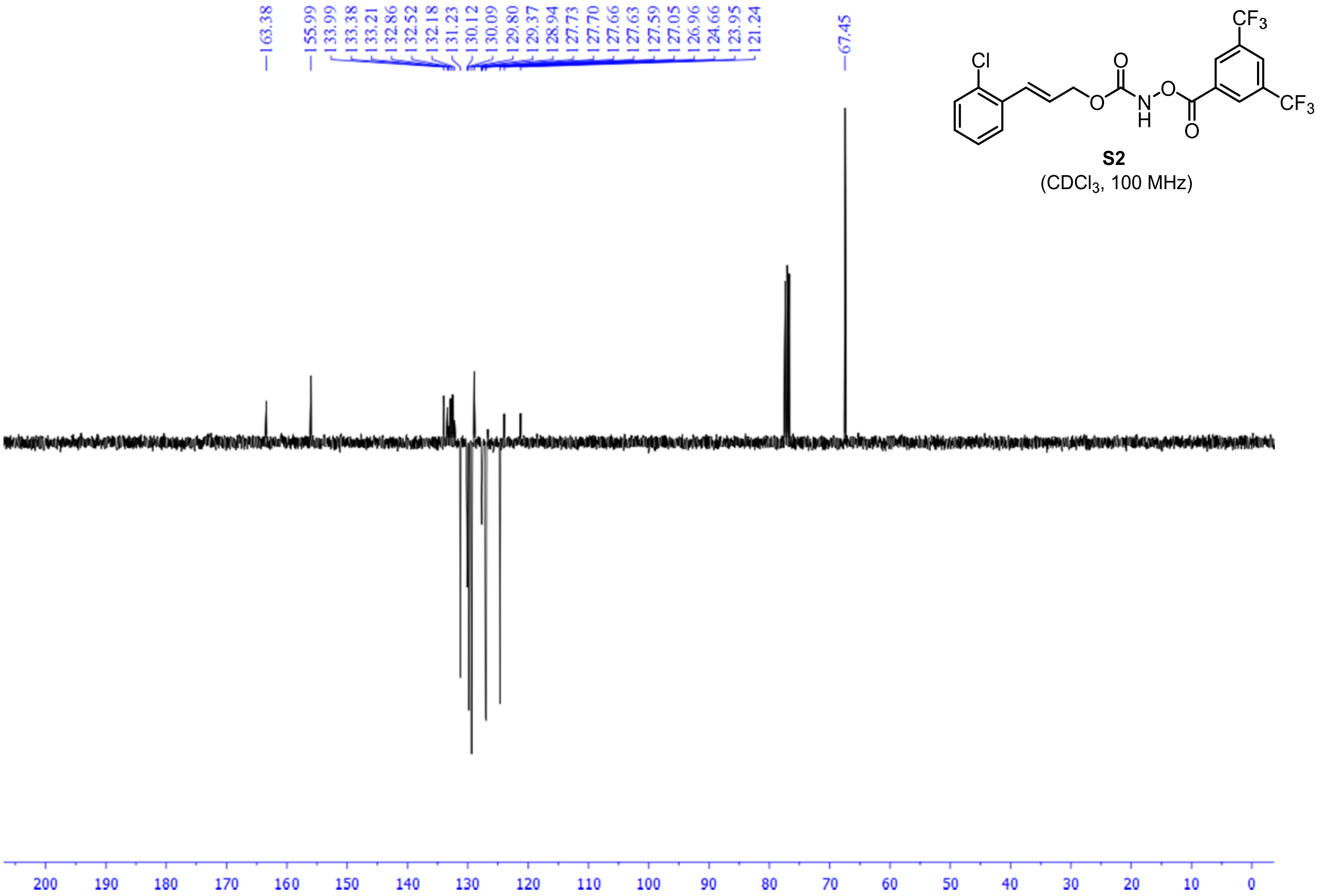

S2

($CDCl_3$, 100 MHz)

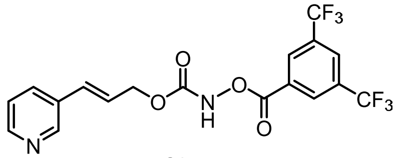

S3

($CDCl_3$, 400 MHz)

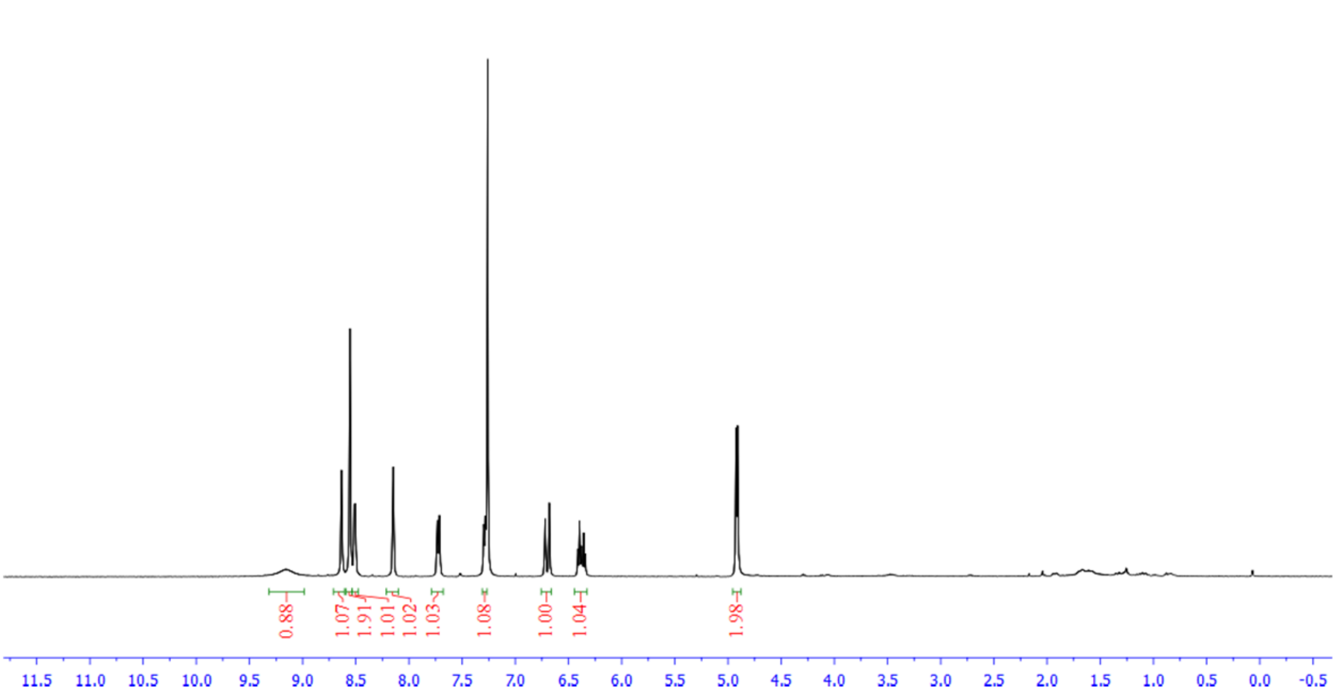

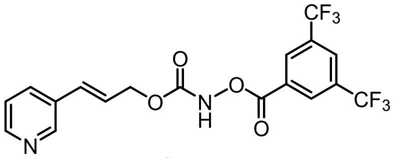

S3

($CDCl_3$, 100 MHz)

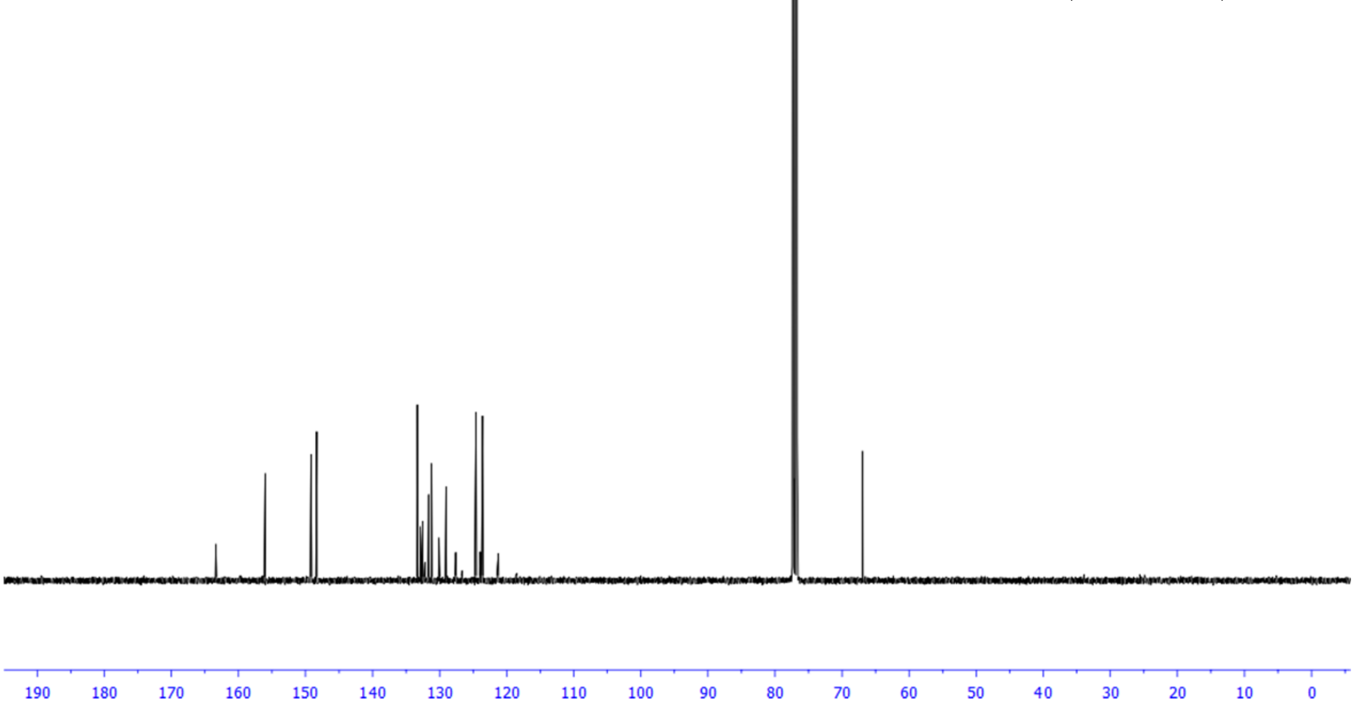

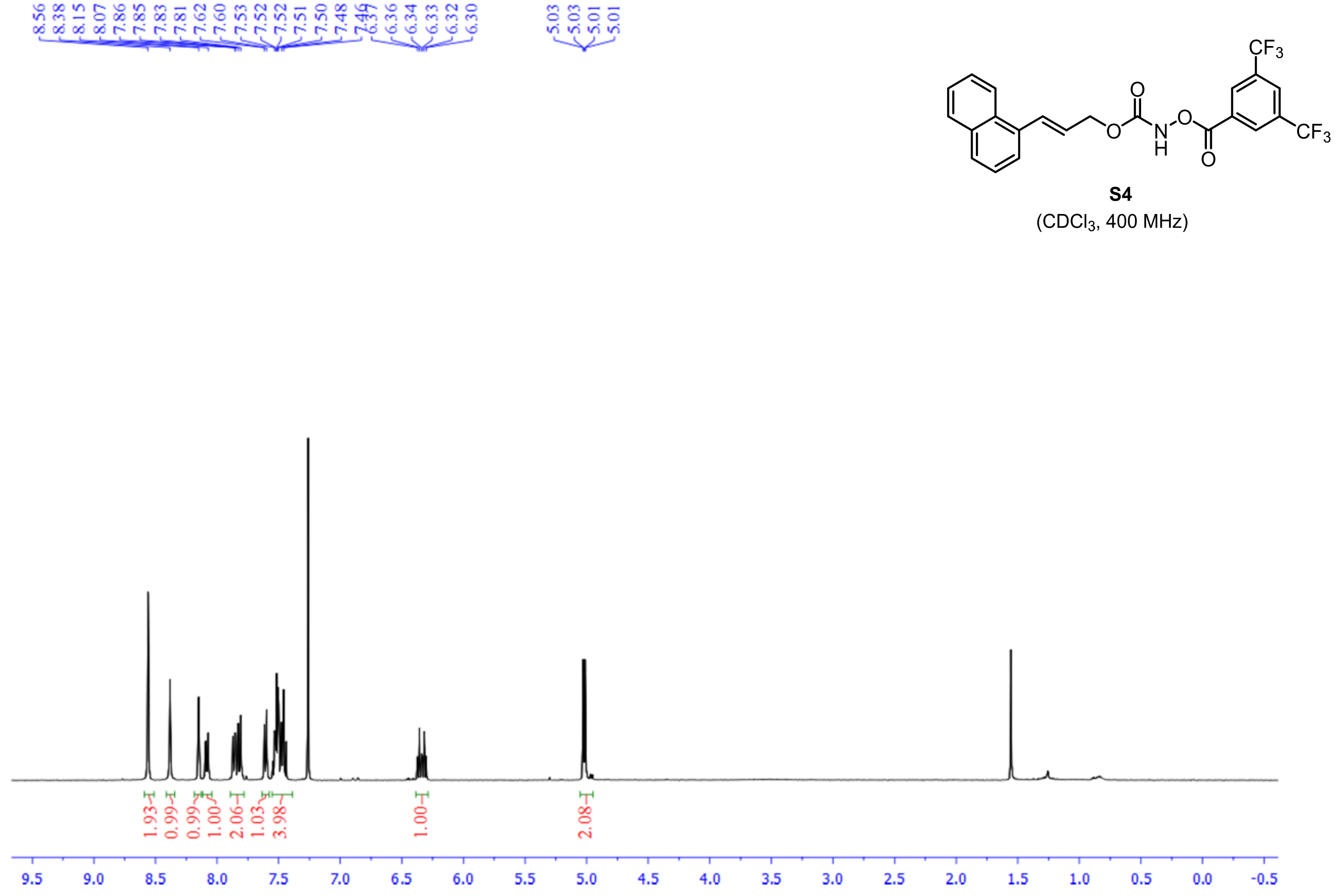

S4

($CDCl_3$, 400 MHz)

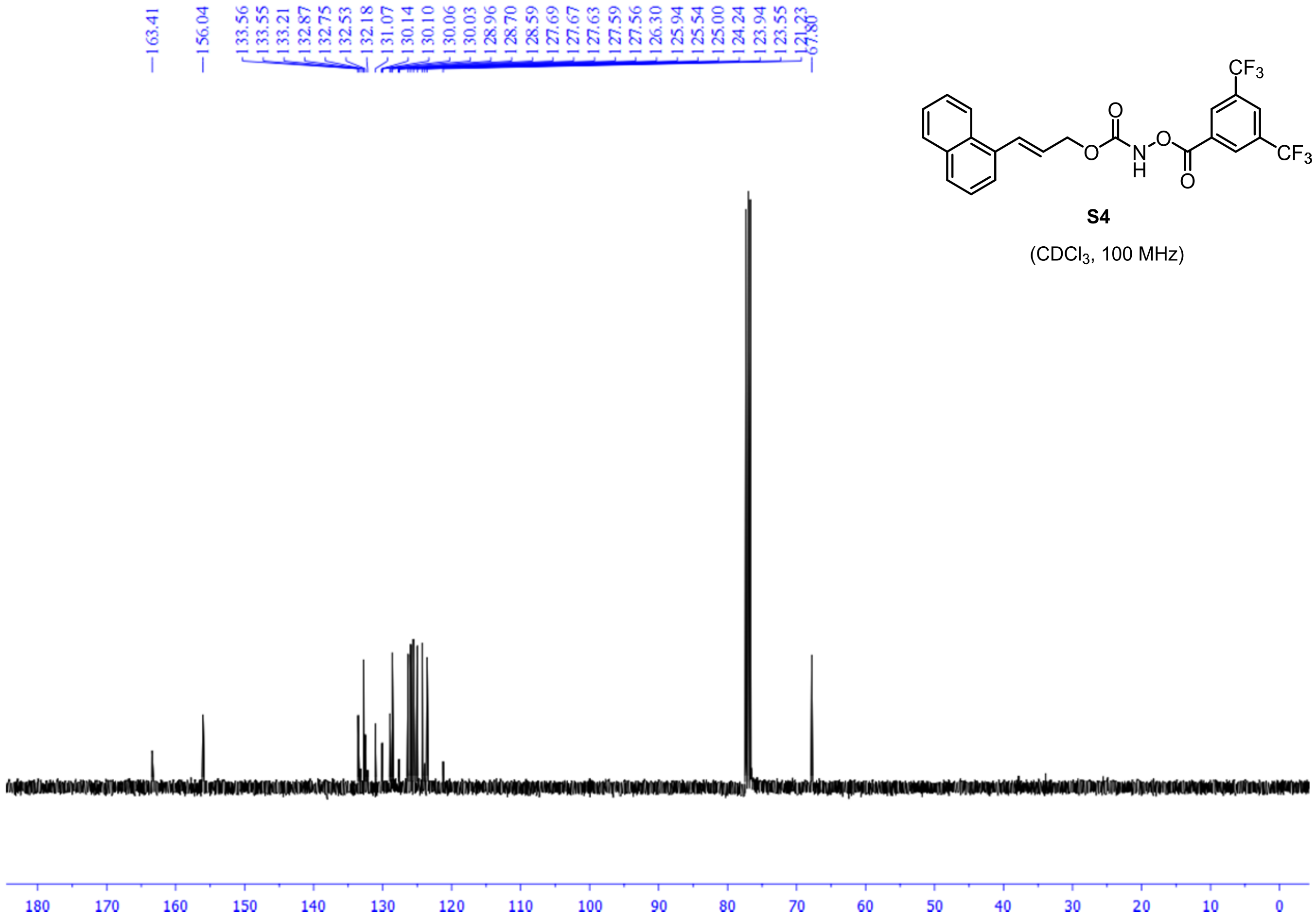

S4

($CDCl_3$, 100 MHz)

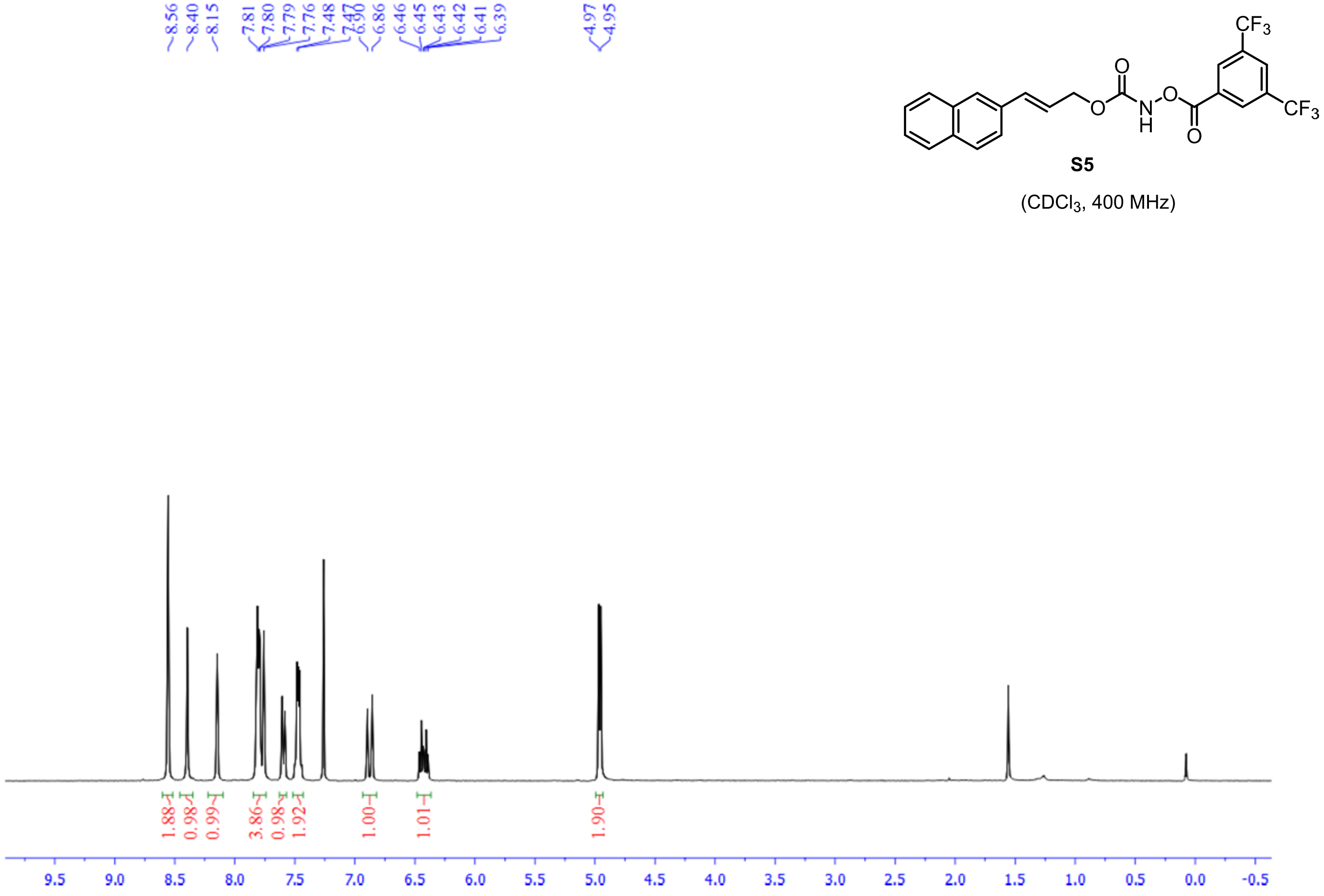
8.56
8.40
8.15
7.81
7.80
7.79
7.76
7.48
7.47
6.90
6.86
6.46
6.45
6.43
6.42
6.41
6.39
4.97
4.95
CF3
O
O
N
H
O
O
CF3
S5
(CDCl3, 400 MHz)
1.88
0.98
0.99
3.86
0.98
1.92
1.00
1.01
1.90
9.5
9.0
8.5
8.0
7.5
7.0
6.5
6.0
5.5
5.0
4.5
4.0
3.5
3.0
2.5
2.0
1.5
1.0
0.5
0.0
-0.5

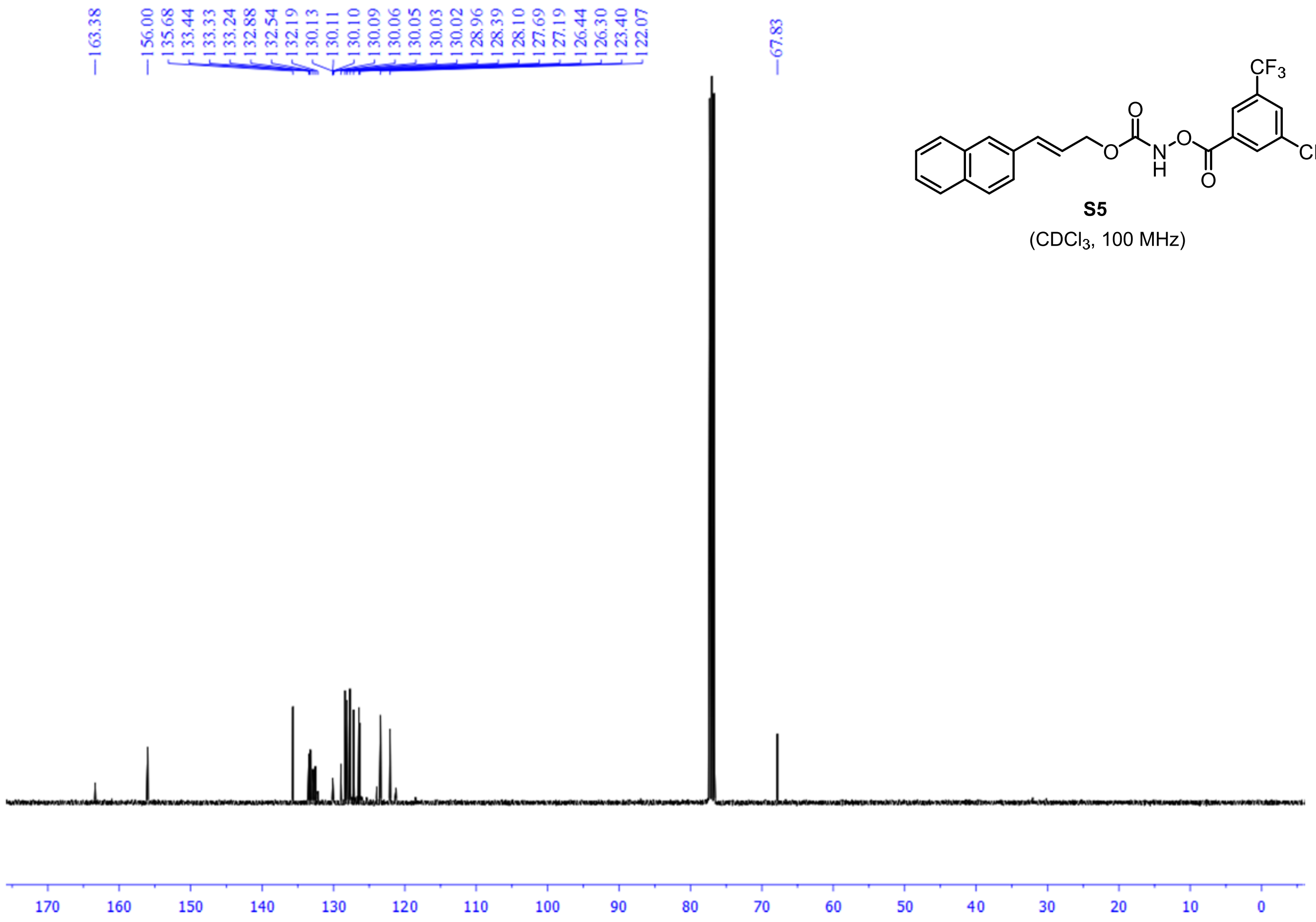
163.38
156.00
135.68
133.44
133.33
133.24
132.88
132.54
132.19
130.13
130.11
130.10
130.09
130.06
130.05
130.03
130.02
128.96
128.39
128.10
127.69
127.19
126.44
126.30
123.40
122.07
67.83
CF3
O
O
N
H
O
O
CF3
S5
(CDCl3, 100 MHz)
170
160
150
140
130
120
110
100
90
80
70
60
50
40
30
20
10
0

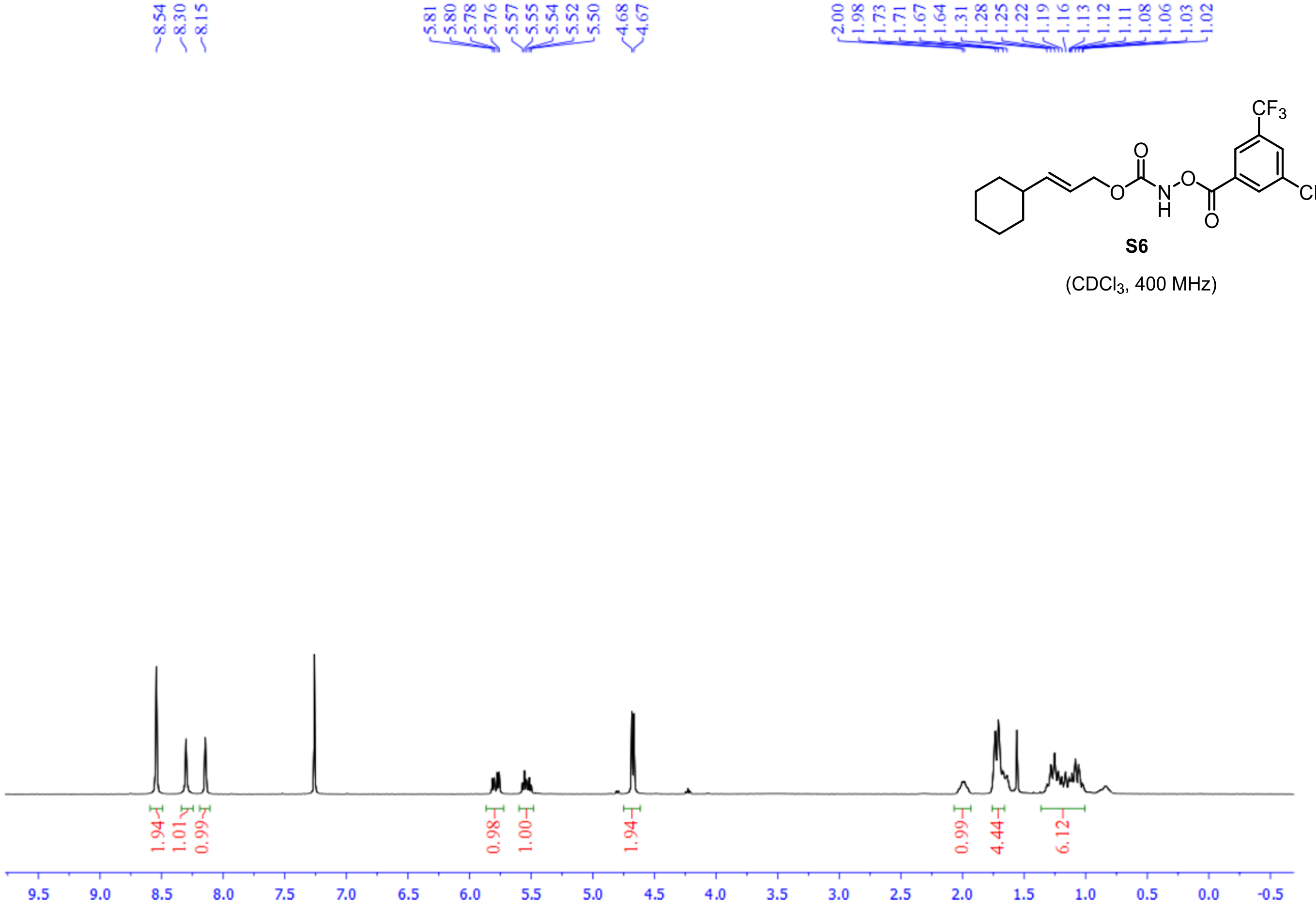
8.54
8.30
8.15
5.81
5.80
5.78
5.76
5.57
5.55
5.54
5.52
5.50
4.68
4.67
2.00
1.98
1.73
1.71
1.67
1.64
1.31
1.28
1.25
1.22
1.19
1.16
1.13
1.12
1.11
1.08
1.06
1.03
1.02
CF3
O
N
H
O
O
CF3
S6
(CDCl3, 400 MHz)
1.94
1.01
0.99
0.98
1.00
1.94
0.99
4.44
6.12
9.5 9.0 8.5 8.0 7.5 7.0 6.5 6.0 5.5 5.0 4.5 4.0 3.5 3.0 2.5 2.0 1.5 1.0 0.5 0.0 -0.5

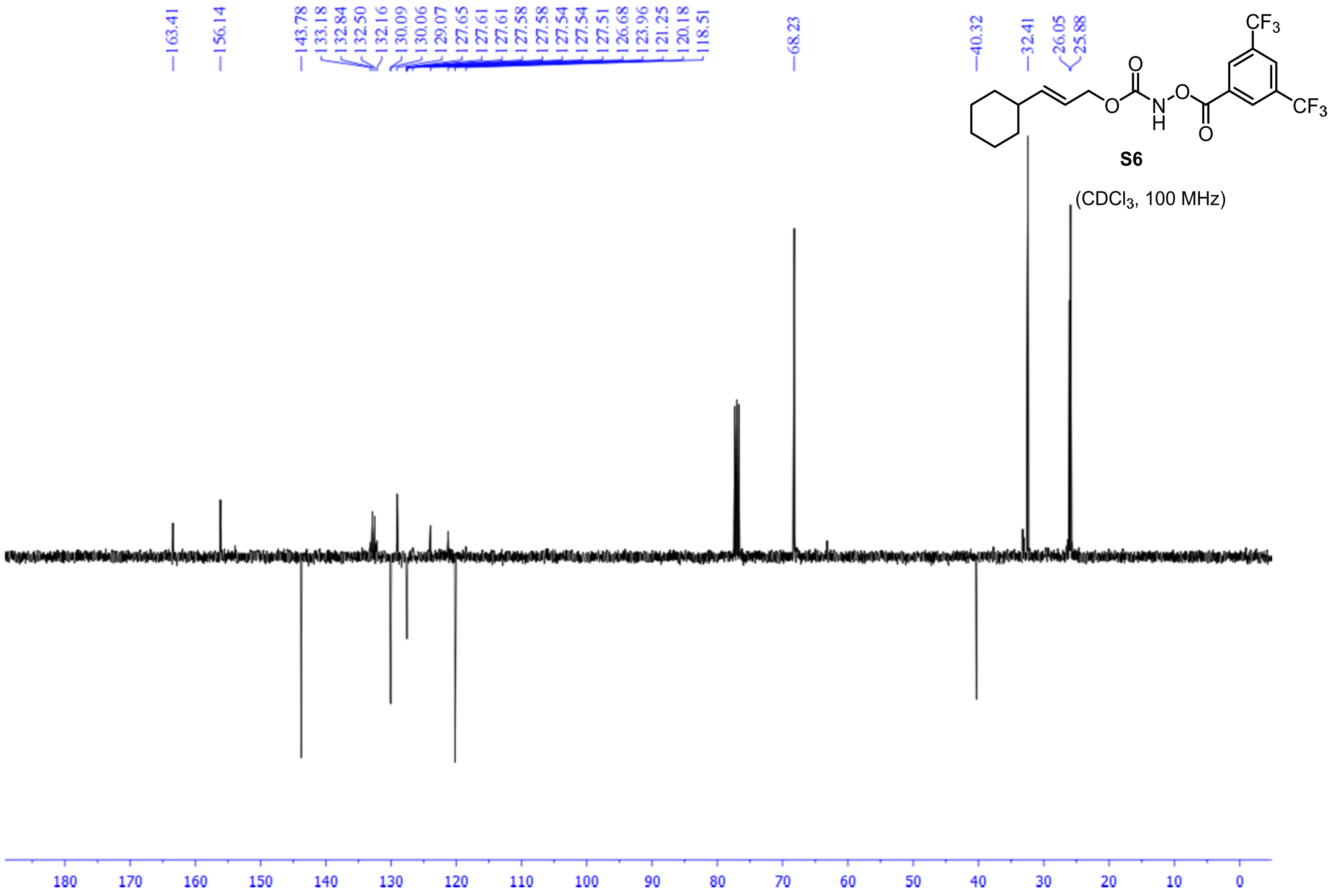
163.41
156.14
143.78
133.18
132.84
132.50
132.16
130.09
130.06
129.07
127.65
127.61
127.61
127.58
127.58
127.54
127.54
127.51
126.68
123.96
121.25
120.18
118.51
68.23
40.32
32.41
26.05
25.88
CF3
O
N
H
O
O
CF3
S6
(CDCl3, 100 MHz)
180 170 160 150 140 130 120 110 100 90 80 70 60 50 40 30 20 10 0

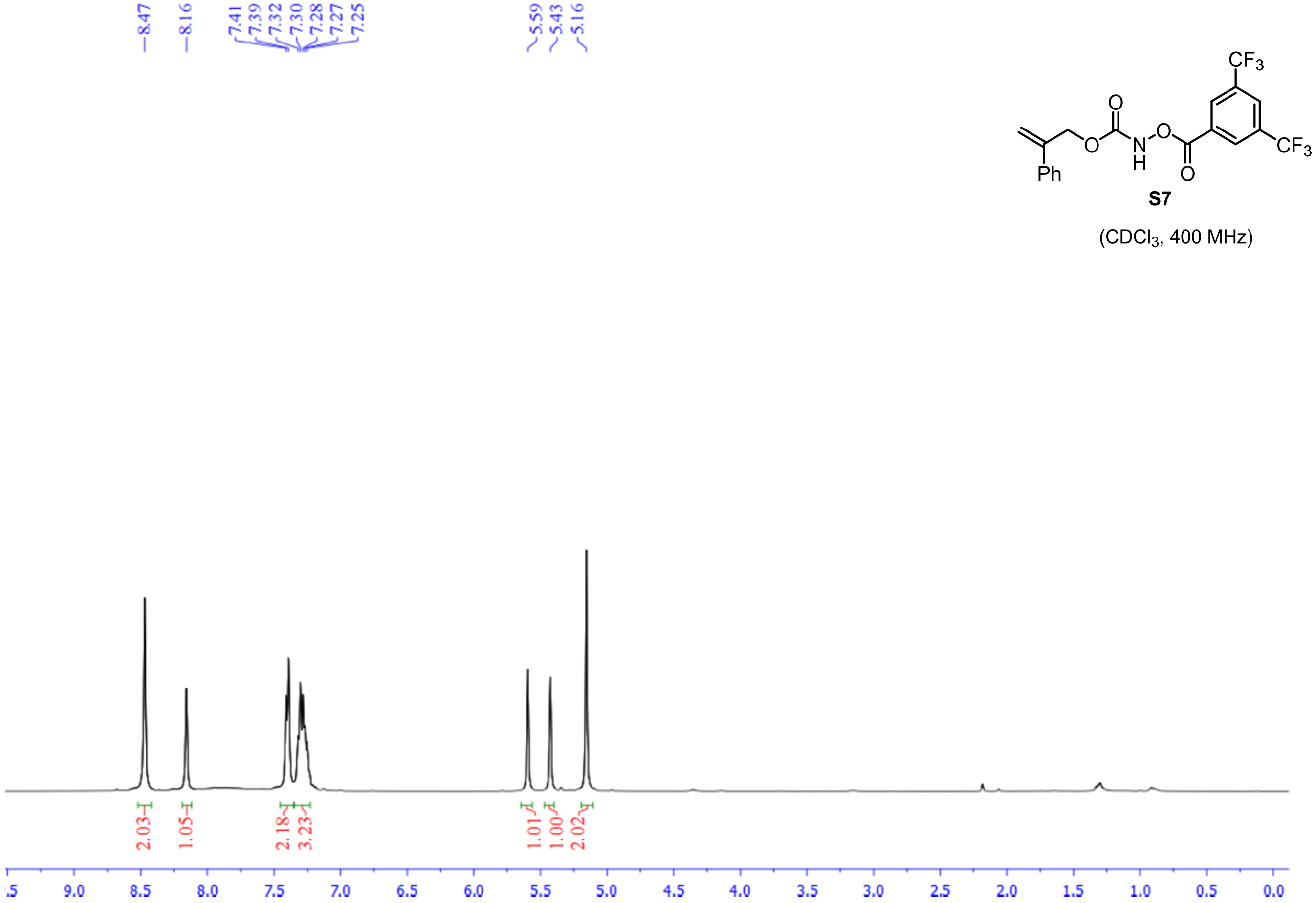

S7

($CDCl_3$, 400 MHz)

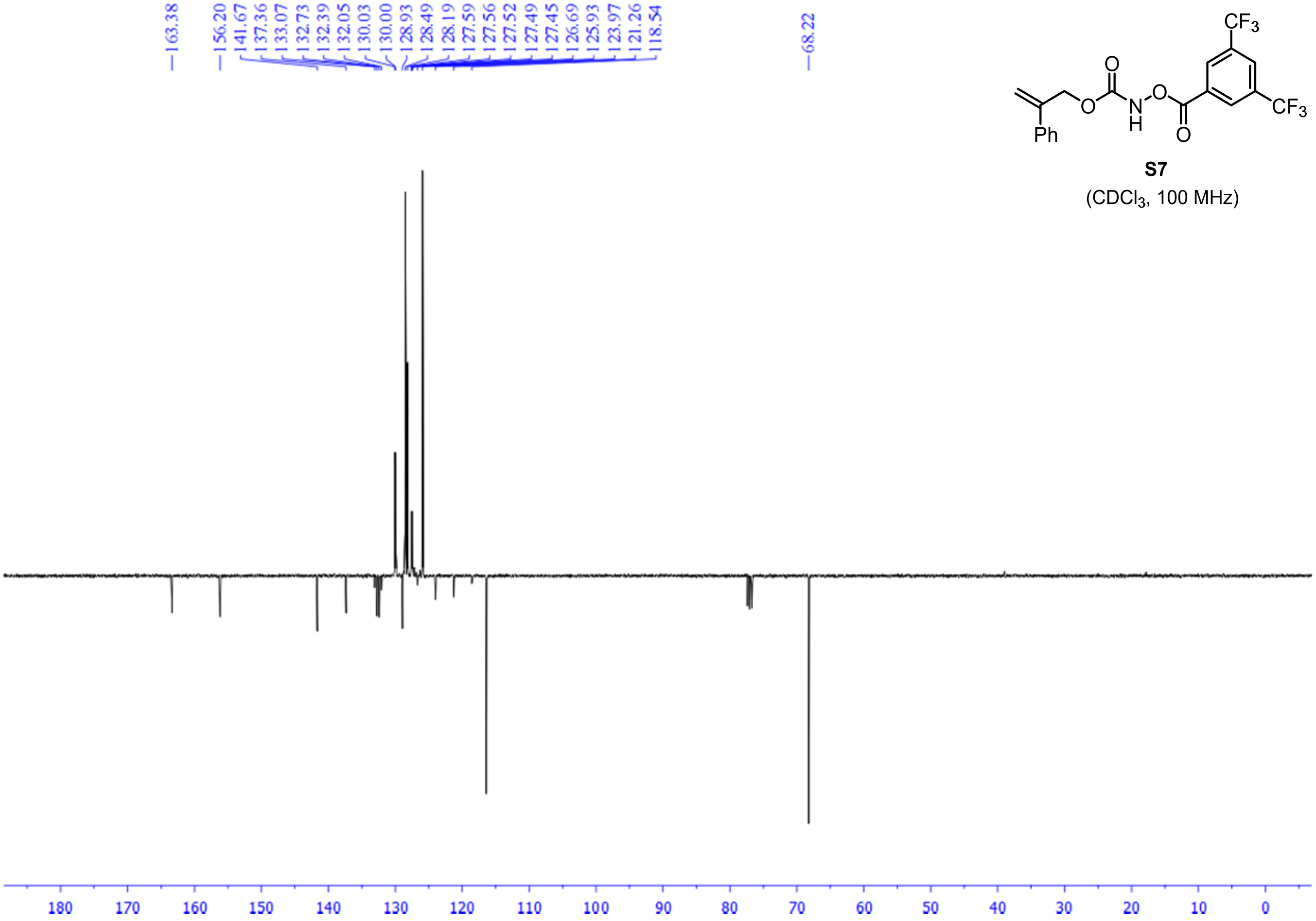

S7

($CDCl_3$, 100 MHz)

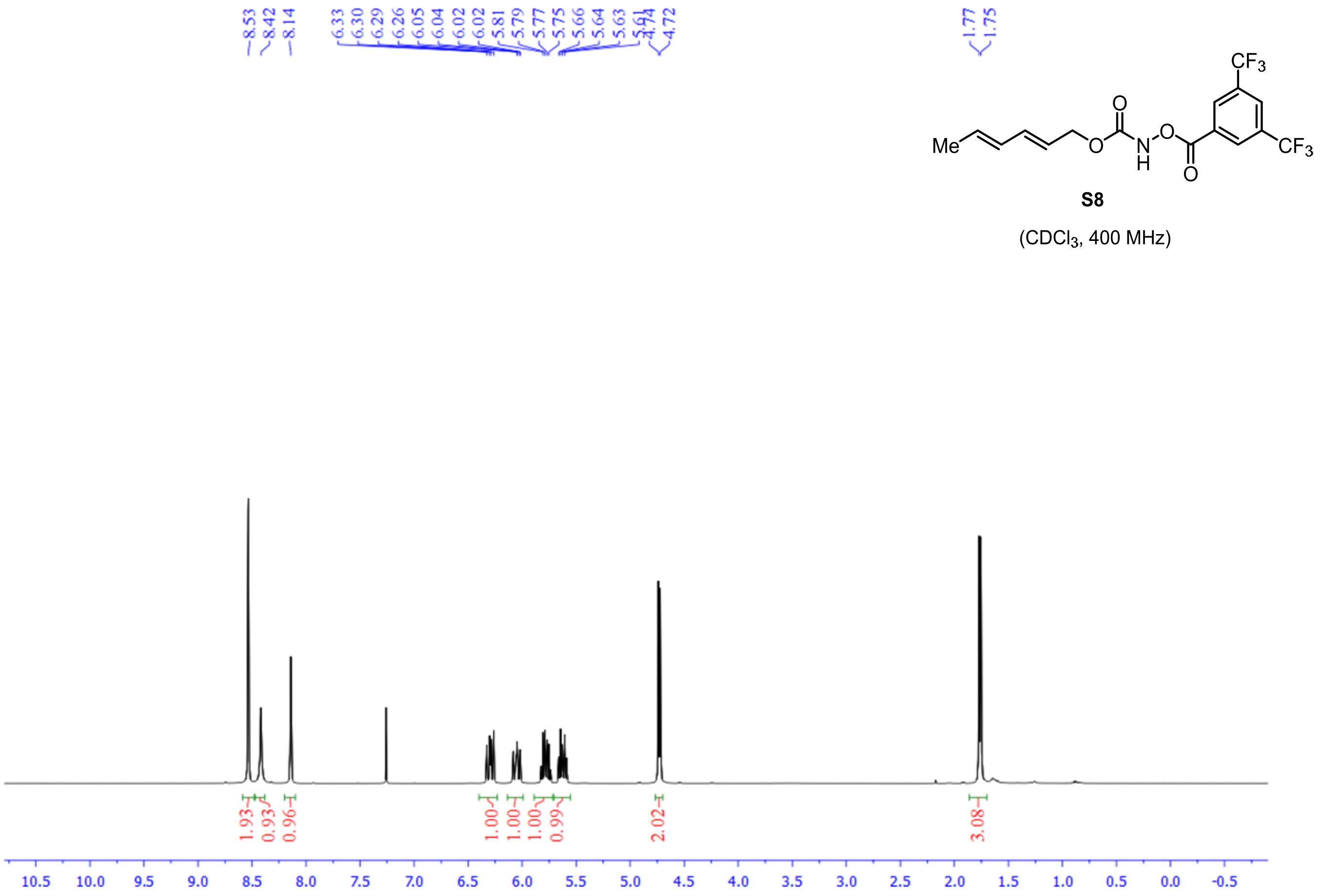
S8
(CDCl3, 400 MHz)

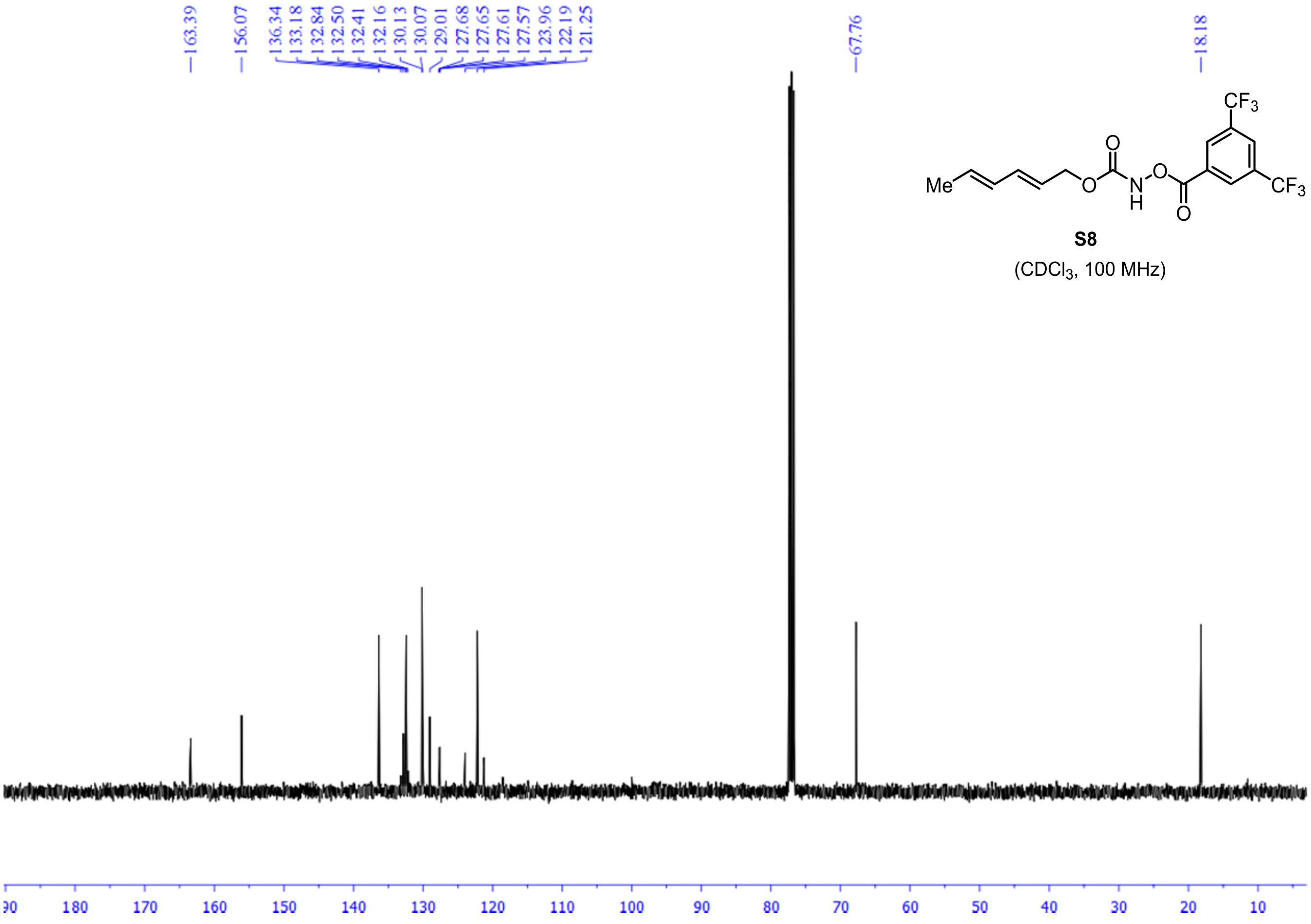
S8
(CDCl3, 100 MHz)

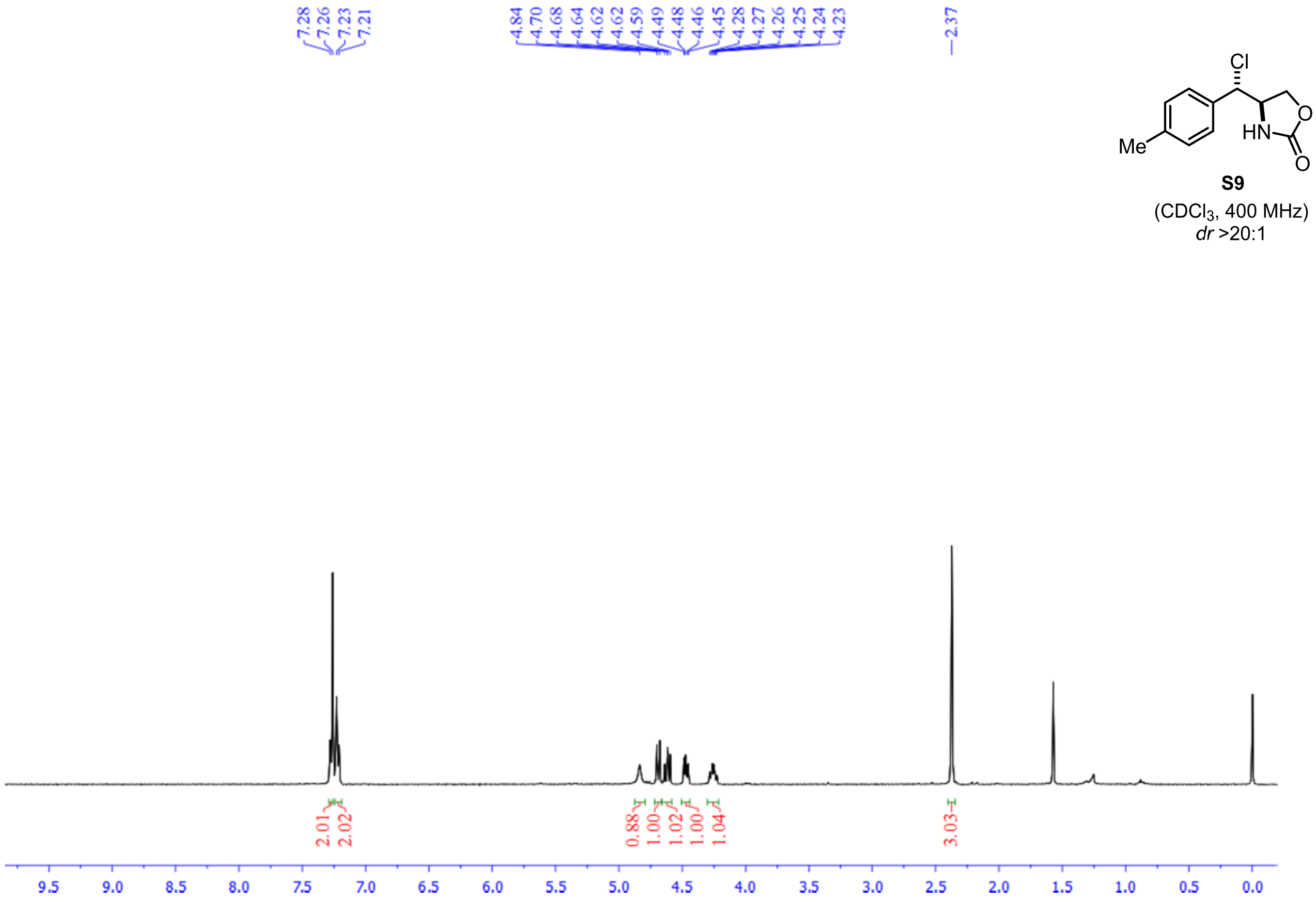
7.28
7.26
7.23
7.21
4.84
4.70
4.68
4.64
4.62
4.62
4.59
4.49
4.48
4.46
4.45
4.28
4.27
4.26
4.25
4.24
4.23
2.37
Cl
HN
O
O
Me
S9
(CDCl3, 400 MHz)
dr >20:1
2.01
2.02
0.88
1.00
1.02
1.00
1.04
3.03
9.5 9.0 8.5 8.0 7.5 7.0 6.5 6.0 5.5 5.0 4.5 4.0 3.5 3.0 2.5 2.0 1.5 1.0 0.5 0.0

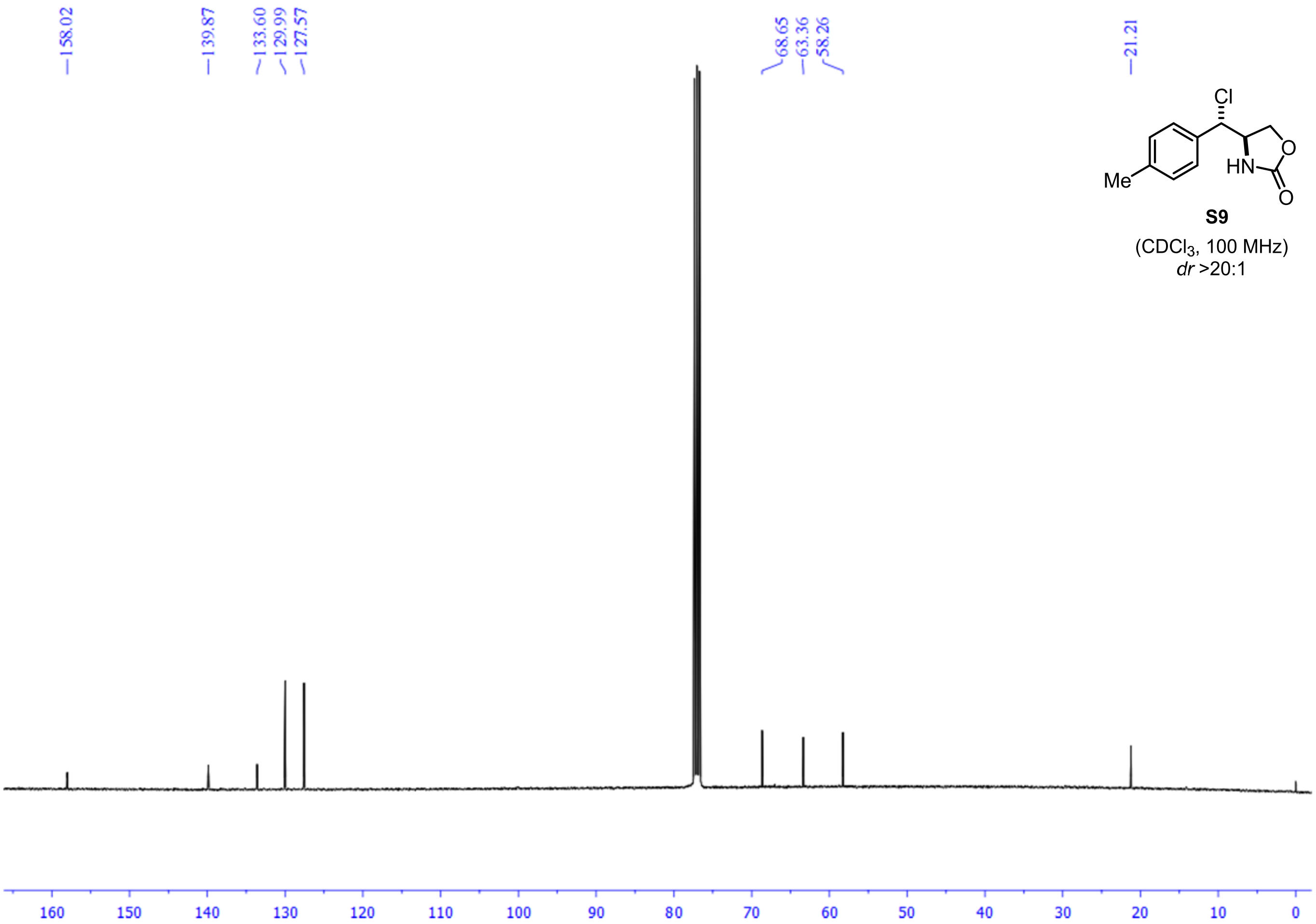
158.02
139.87
133.60
129.99
127.57
68.65
63.36
58.26
21.21
Cl
HN
O
O
Me
S9
(CDCl3, 100 MHz)
dr >20:1
160 150 140 130 120 110 100 90 80 70 60 50 40 30 20 10 0

S10

($CDCl_3$, 400 MHz)
dr >20:1

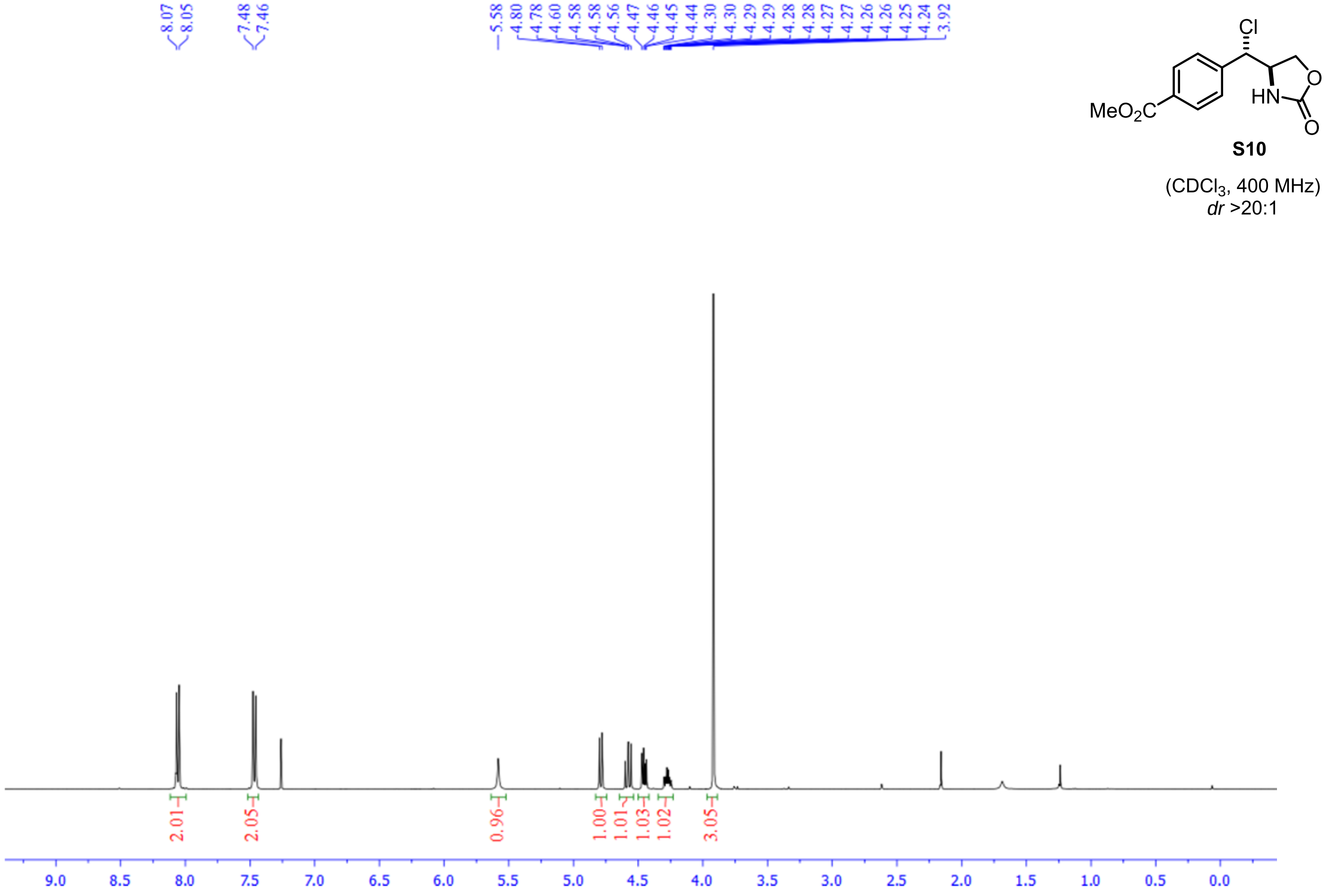

S10

($CDCl_3$, 100 MHz)
dr >20:1

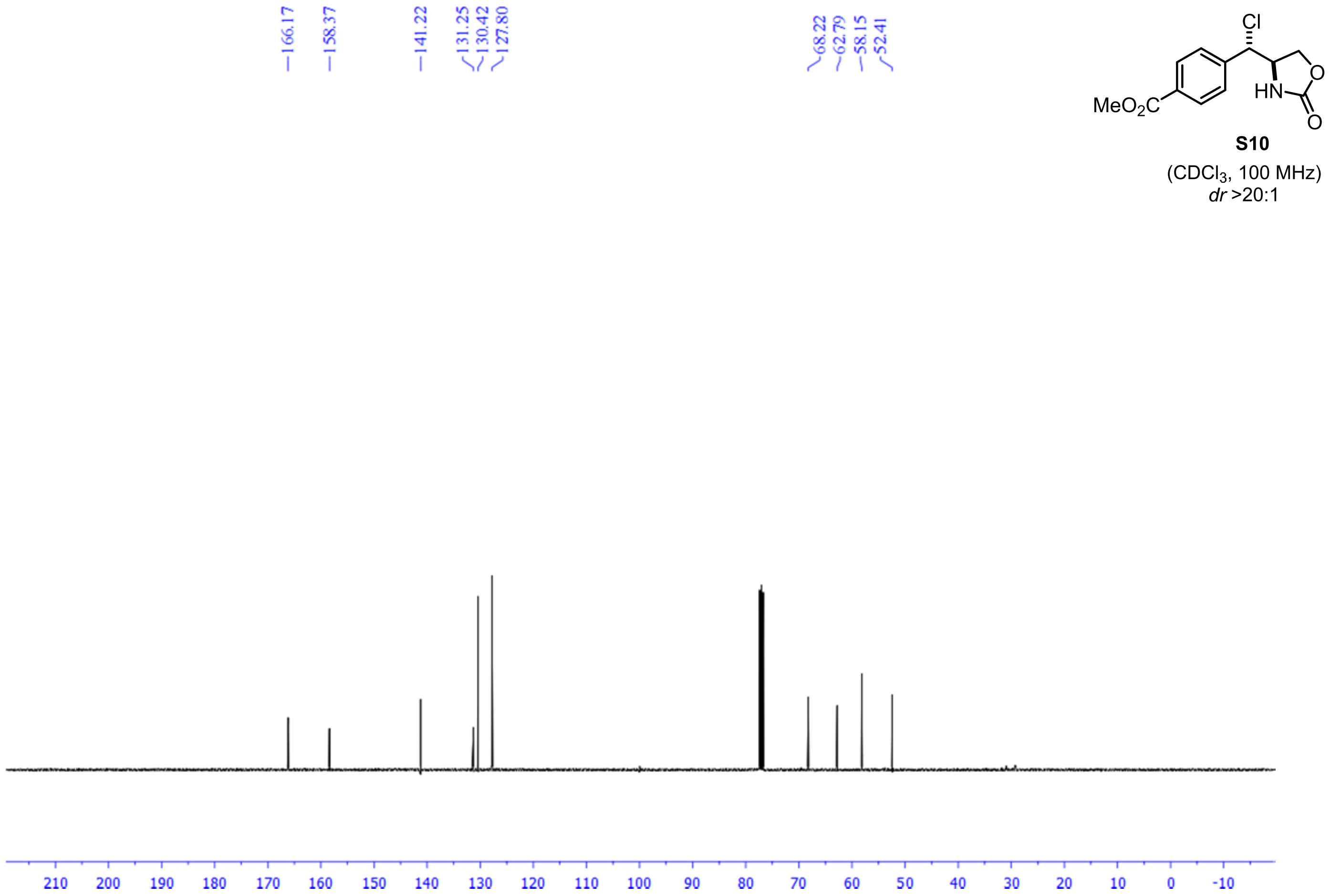

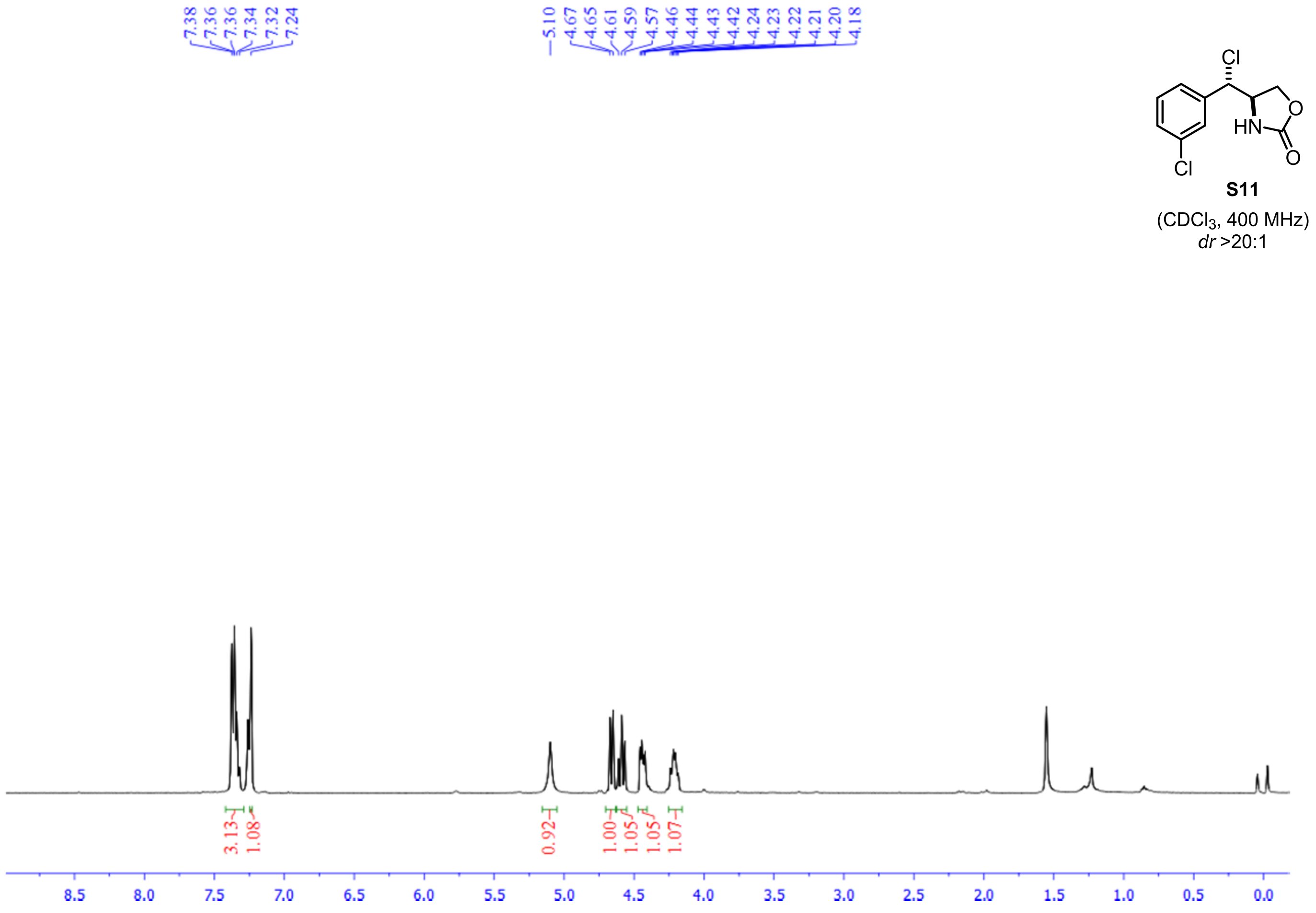
7.38
7.36
7.36
7.34
7.32
7.24
5.10
4.67
4.65
4.61
4.59
4.57
4.46
4.44
4.43
4.42
4.24
4.23
4.22
4.21
4.20
4.18
3.13
1.08
0.92
1.00
1.05
1.05
1.07
Cl
HN
O
O
Cl
S11
(CDCl3, 400 MHz)
dr >20:1

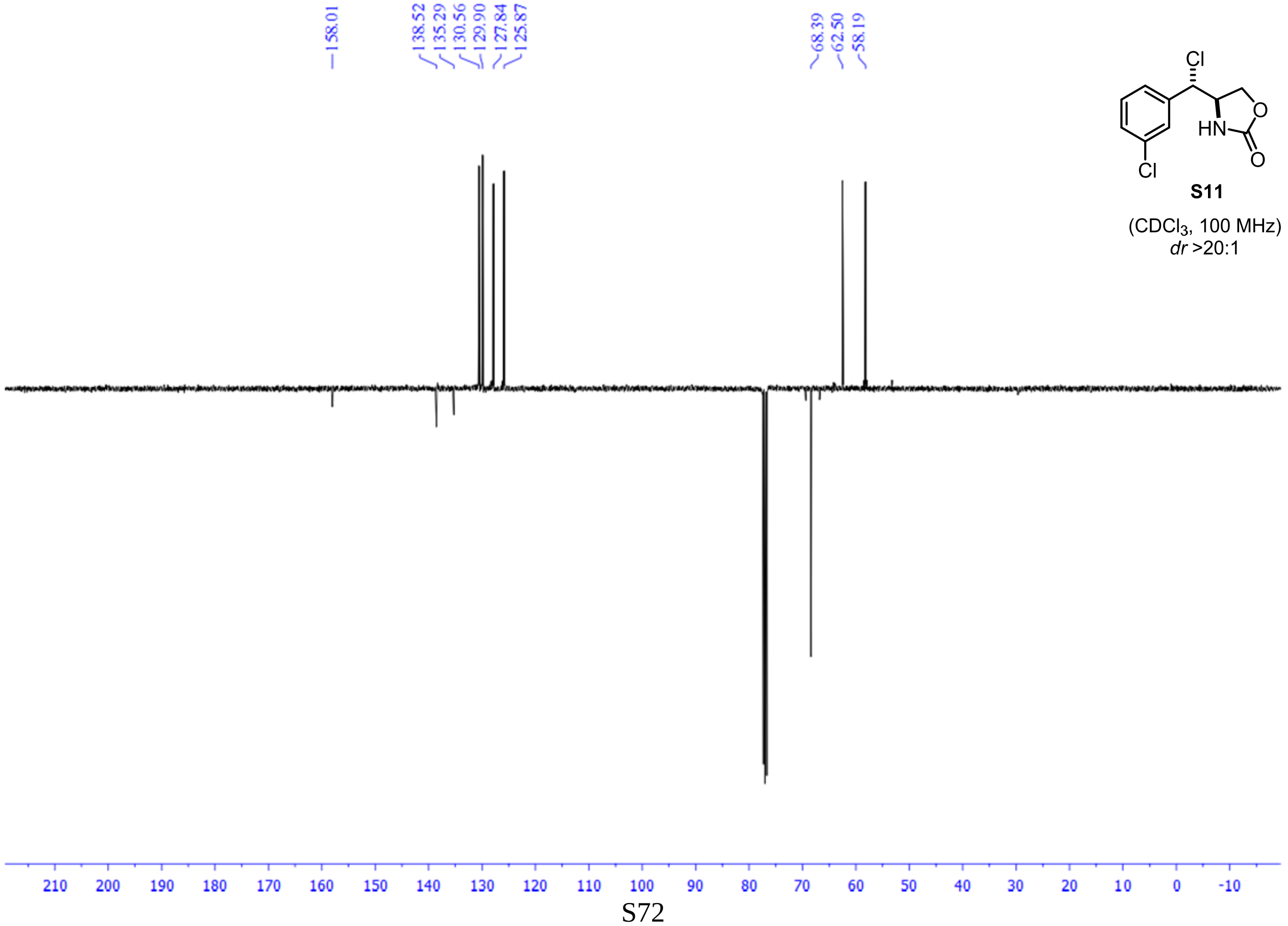
158.01
138.52
135.29
130.56
129.90
127.84
125.87
68.39
62.50
58.19
Cl
HN
O
O
Cl
S11
(CDCl3, 100 MHz)
dr >20:1

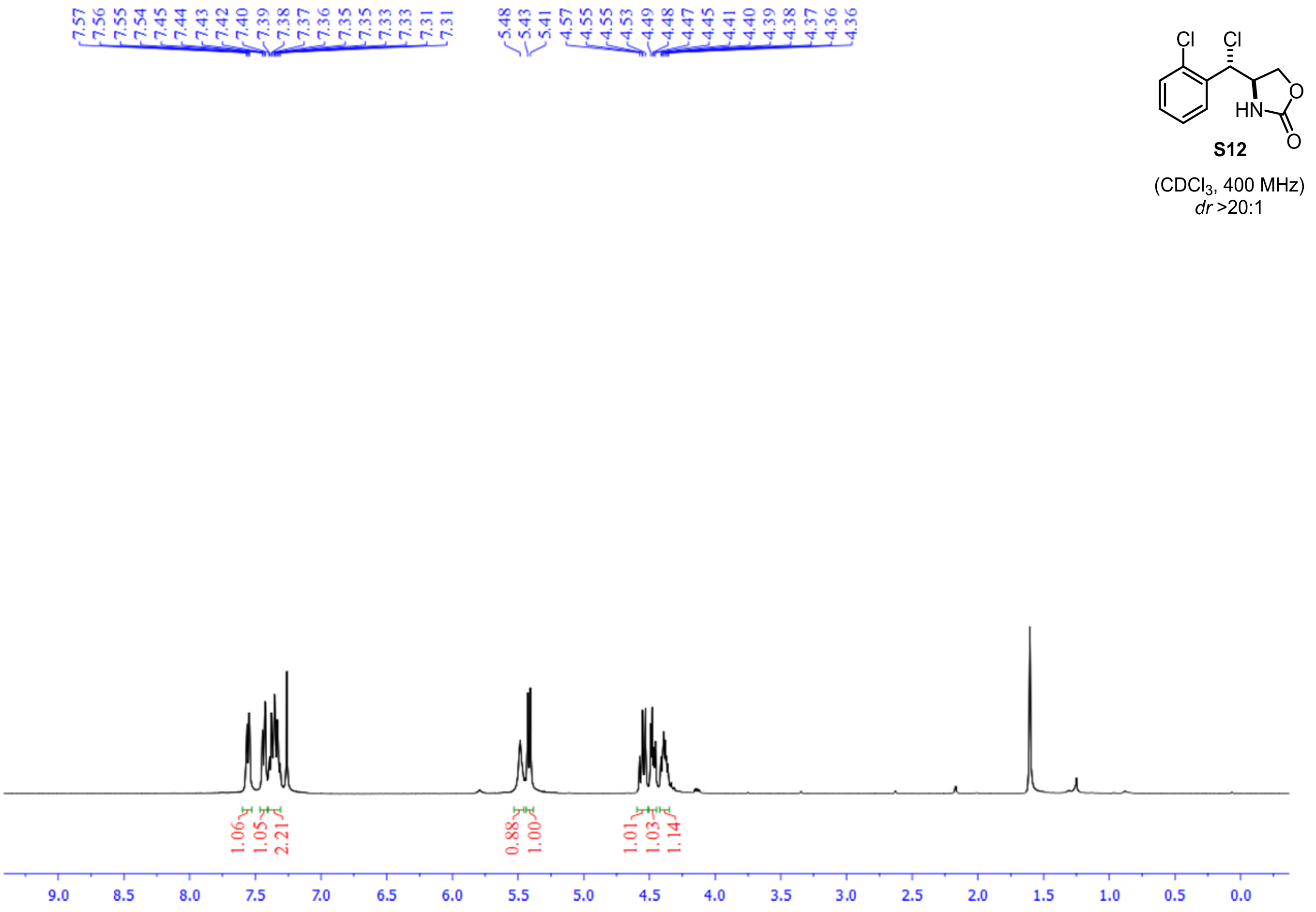
7.57
7.56
7.55
7.54
7.45
7.44
7.43
7.42
7.40
7.39
7.38
7.37
7.36
7.35
7.35
7.33
7.33
7.31
7.31
5.48
5.43
5.41
4.57
4.55
4.55
4.53
4.49
4.48
4.47
4.45
4.41
4.40
4.39
4.38
4.37
4.36
4.36
Cl
Cl
O
HN
O
S12
(CDCl3, 400 MHz)
dr >20:1
1.06
1.05
2.21
0.88
1.00
1.01
1.03
1.14
9.0
8.5
8.0
7.5
7.0
6.5
6.0
5.5
5.0
4.5
4.0
3.5
3.0
2.5
2.0
1.5
1.0
0.5
0.0

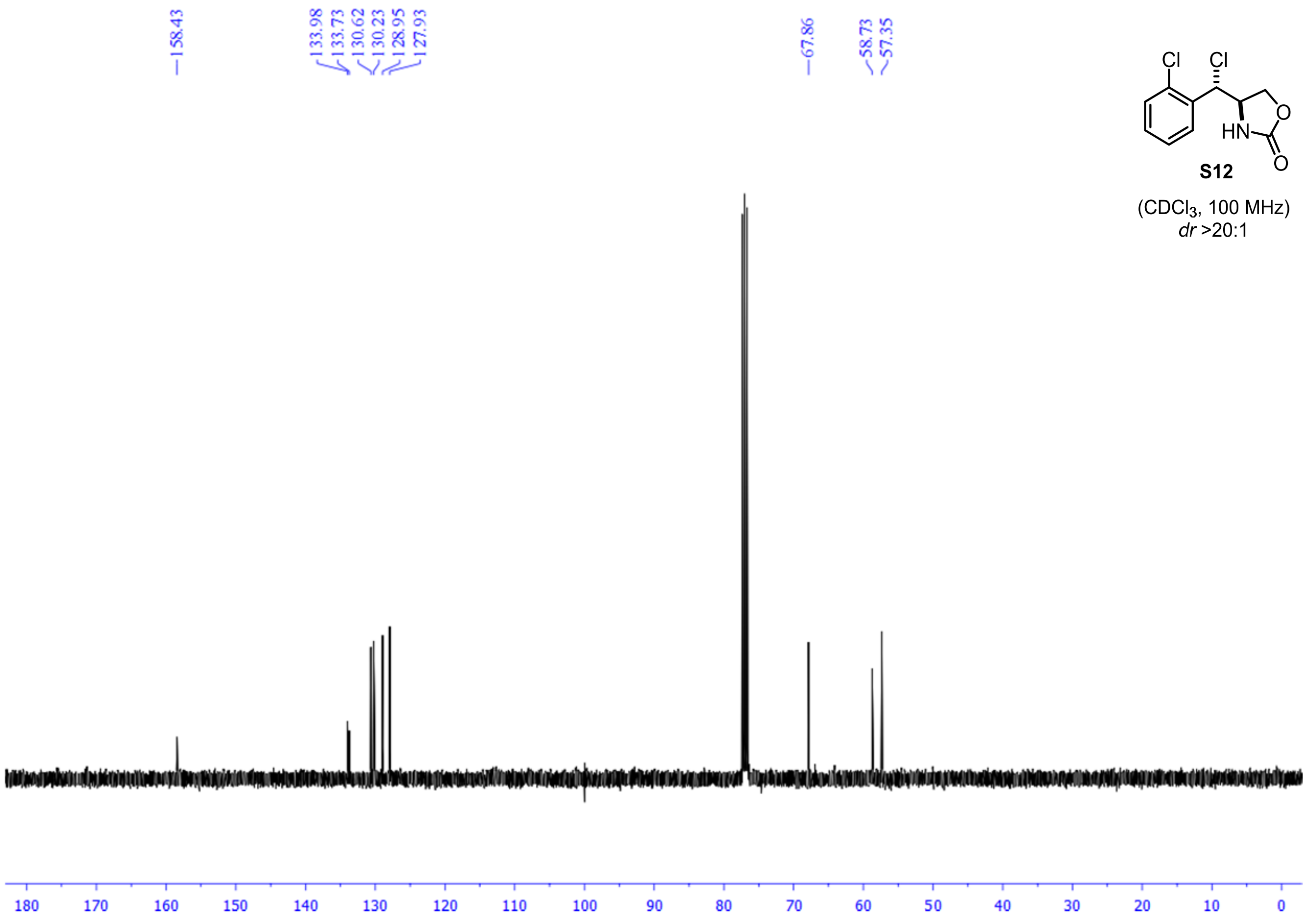
158.43
133.98
133.73
130.62
130.23
128.95
127.93
67.86
58.73
57.35
Cl
Cl
O
HN
O
S12
(CDCl3, 100 MHz)
dr >20:1
180
170
160
150
140
130
120
110
100
90
80
70
60
50
40
30
20
10
0

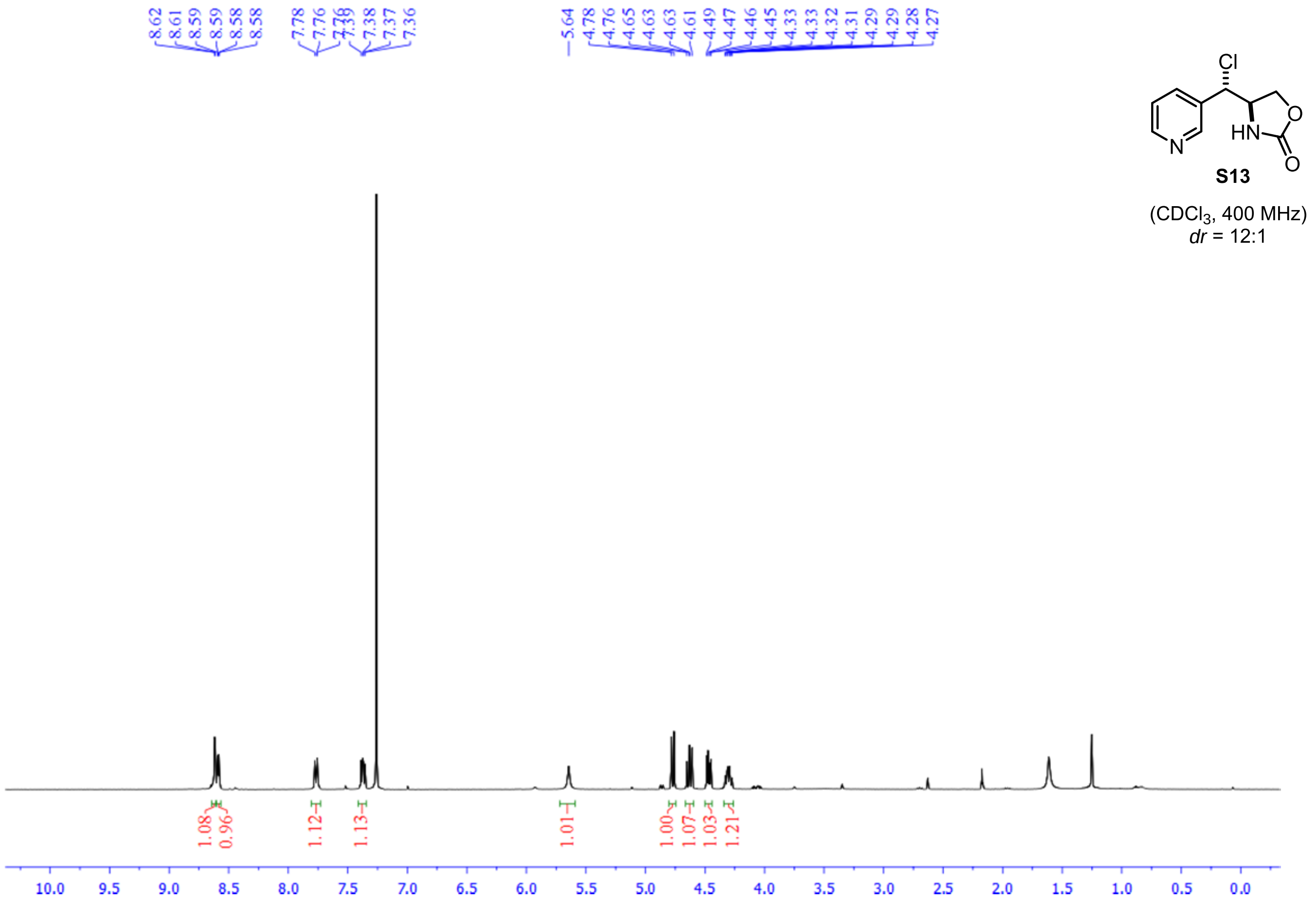
8.62
8.61
8.59
8.59
8.58
8.58
7.78
7.76
7.39
7.38
7.37
7.36
5.64
4.78
4.76
4.65
4.63
4.63
4.61
4.49
4.47
4.46
4.45
4.33
4.33
4.32
4.31
4.29
4.29
4.28
4.27
Cl
HN
O
O
N
S13
(CDCl3, 400 MHz)
dr = 12:1
1.08
0.96
1.12
1.13
1.01
1.00
1.07
1.03
1.21
10.0
9.5
9.0
8.5
8.0
7.5
7.0
6.5
6.0
5.5
5.0
4.5
4.0
3.5
3.0
2.5
2.0
1.5
1.0
0.5
0.0

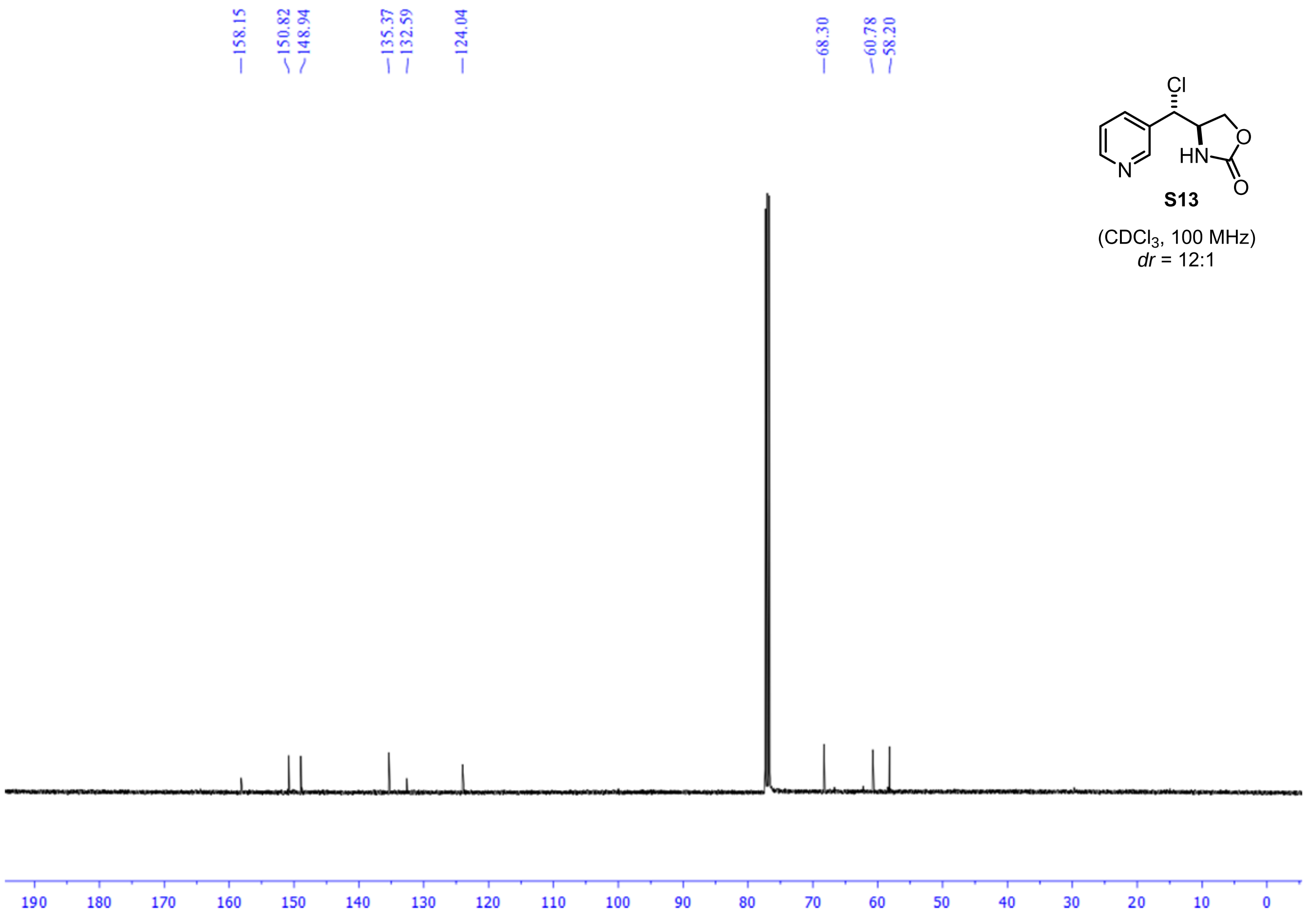
158.15
150.82
148.94
135.37
132.59
124.04
68.30
60.78
58.20
Cl
HN
O
O
N
S13
(CDCl3, 100 MHz)
dr = 12:1
190
180
170
160
150
140
130
120
110
100
90
80
70
60
50
40
30
20
10
0

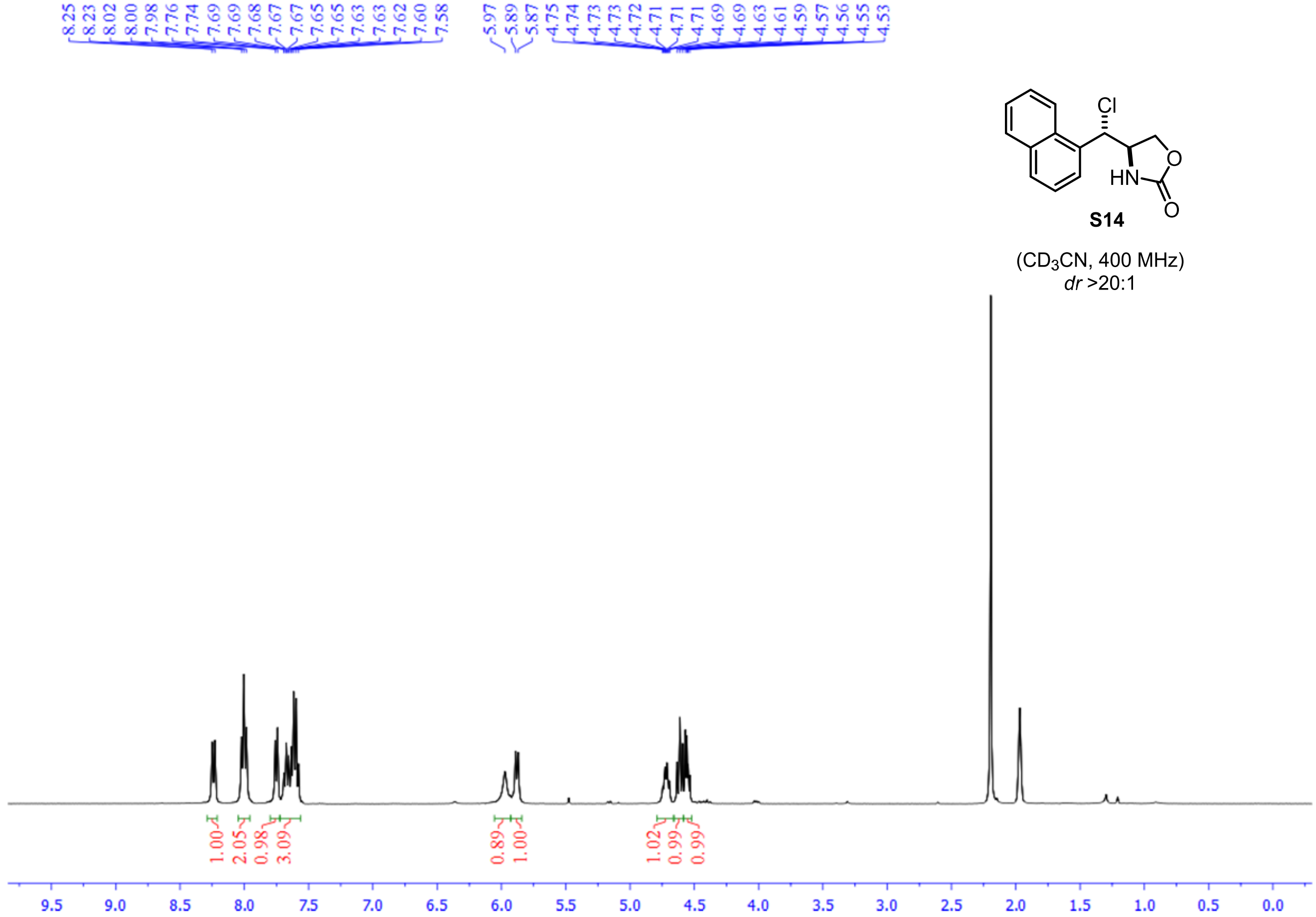
8.25 8.23 8.02 8.00 7.98 7.76 7.74 7.69 7.69 7.68 7.67 7.67 7.65 7.65 7.63 7.63 7.62 7.60 7.58
5.97 5.89 5.87 4.75 4.74 4.73 4.73 4.72 4.71 4.71 4.71 4.69 4.69 4.63 4.61 4.59 4.57 4.56 4.55 4.53
Cl
HN
O
O
S14
(CD3CN, 400 MHz)
dr >20:1
1.00 2.05 0.98 3.09 0.89 1.00 1.02 0.99 0.99
9.5 9.0 8.5 8.0 7.5 7.0 6.5 6.0 5.5 5.0 4.5 4.0 3.5 3.0 2.5 2.0 1.5 1.0 0.5 0.0

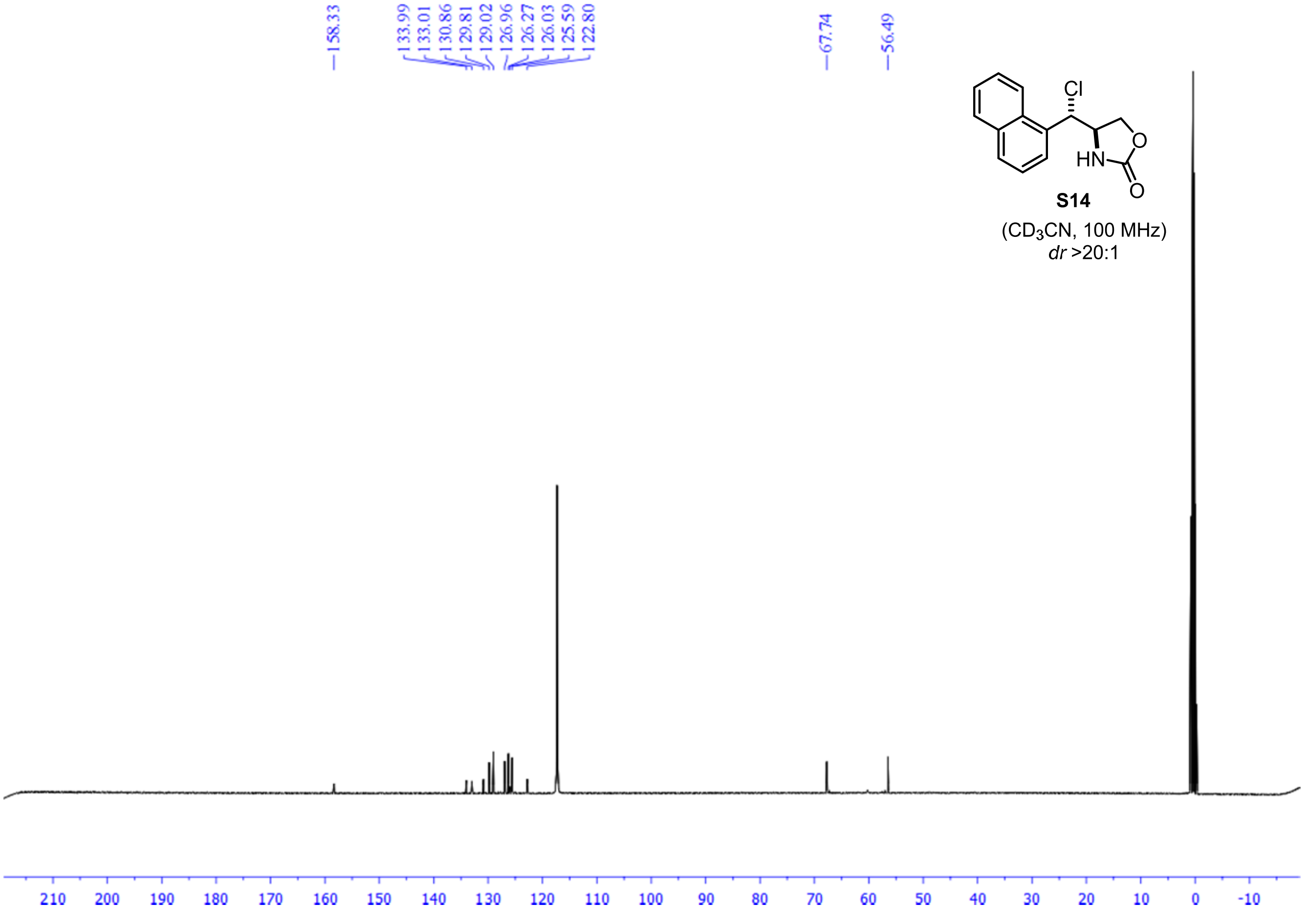
158.33
133.99 133.01 130.86 129.81 129.02 126.96 126.27 126.03 125.59 122.80
67.74
56.49
Cl
HN
O
O
S14
(CD3CN, 100 MHz)
dr >20:1
210 200 190 180 170 160 150 140 130 120 110 100 90 80 70 60 50 40 30 20 10 0 -10

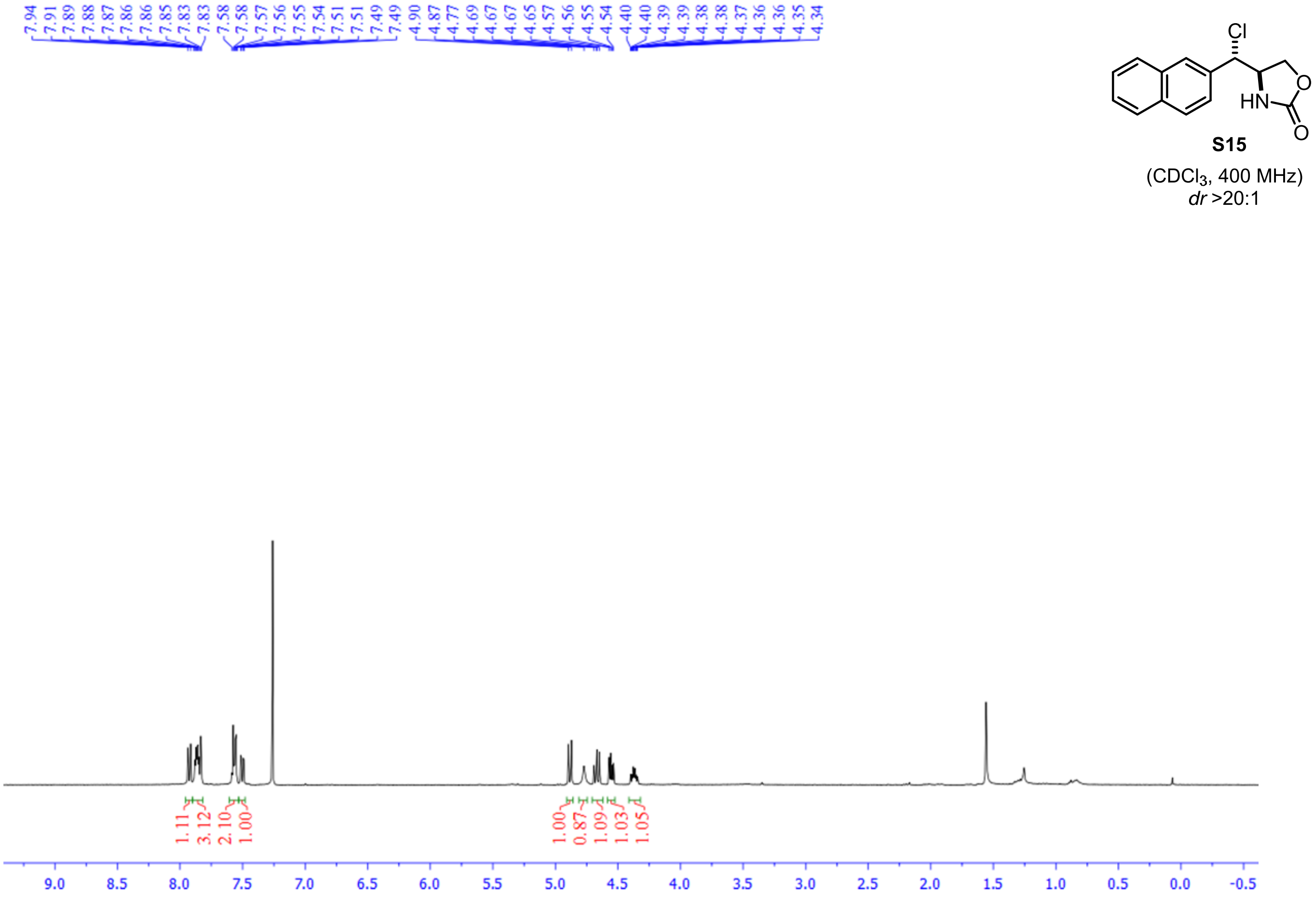

S15

($CDCl_3$, 400 MHz)
dr >20:1

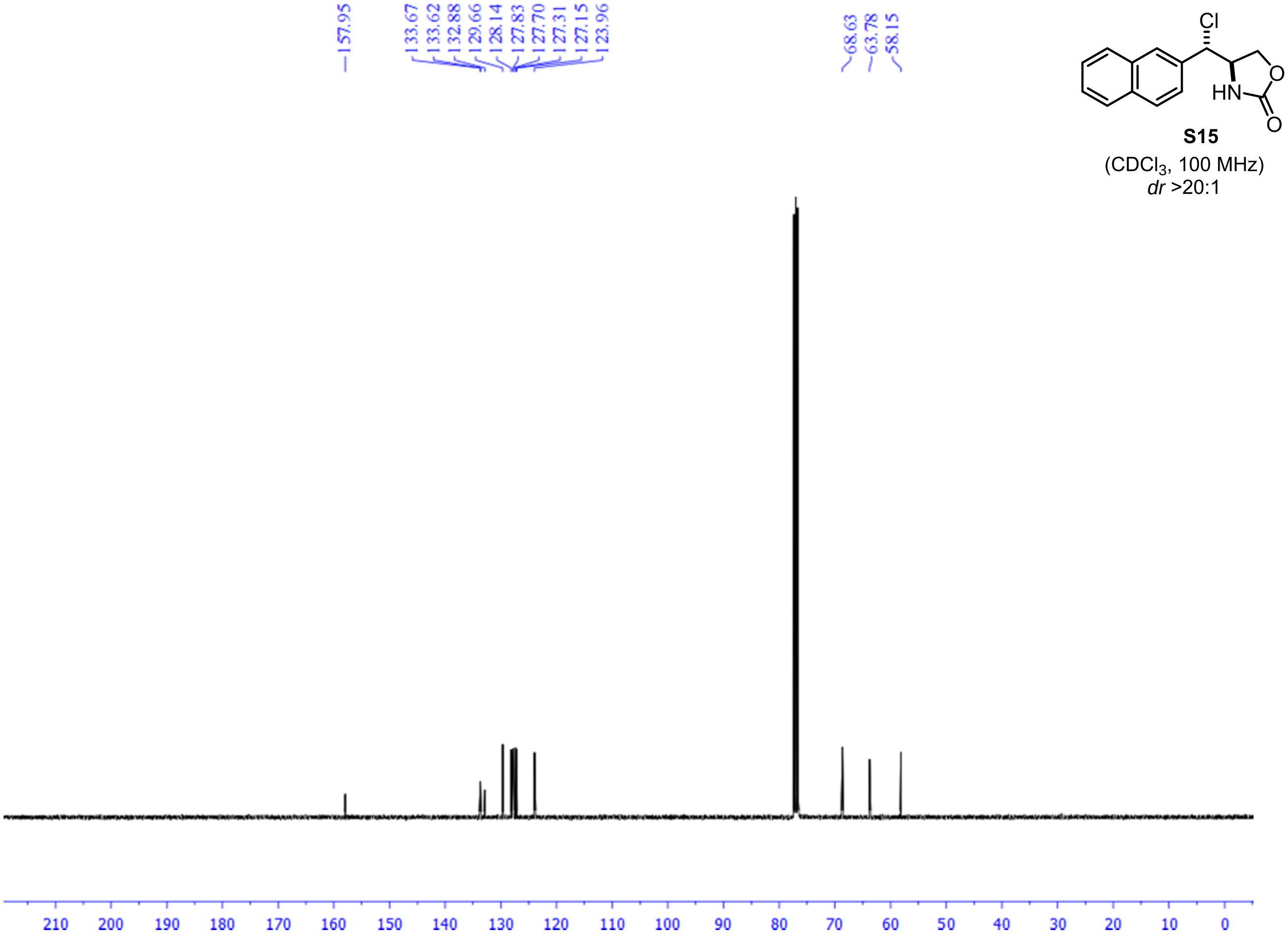

S15

($CDCl_3$, 100 MHz)
dr >20:1

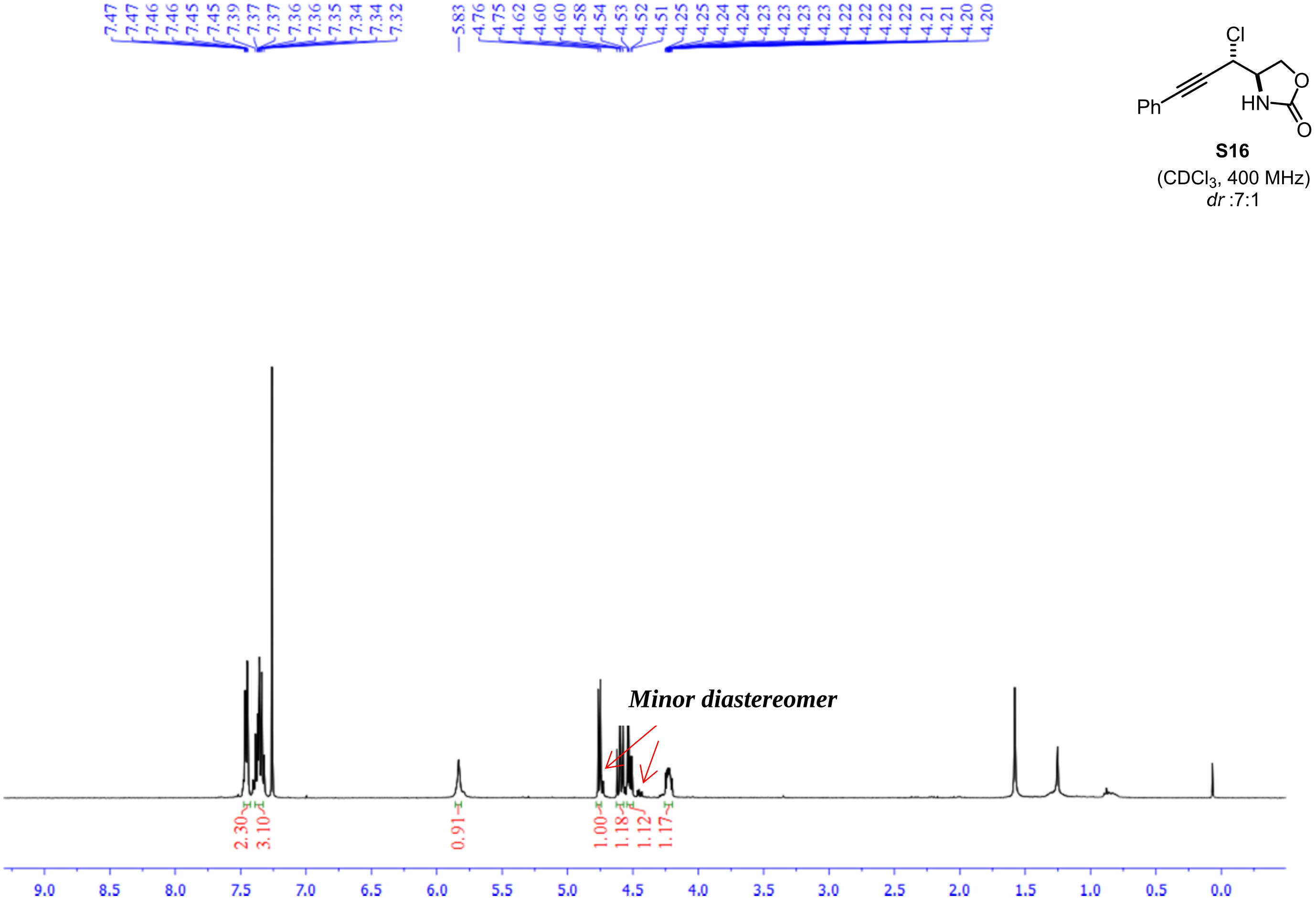

S16

($CDCl_3$, 400 MHz)

dr :7:1

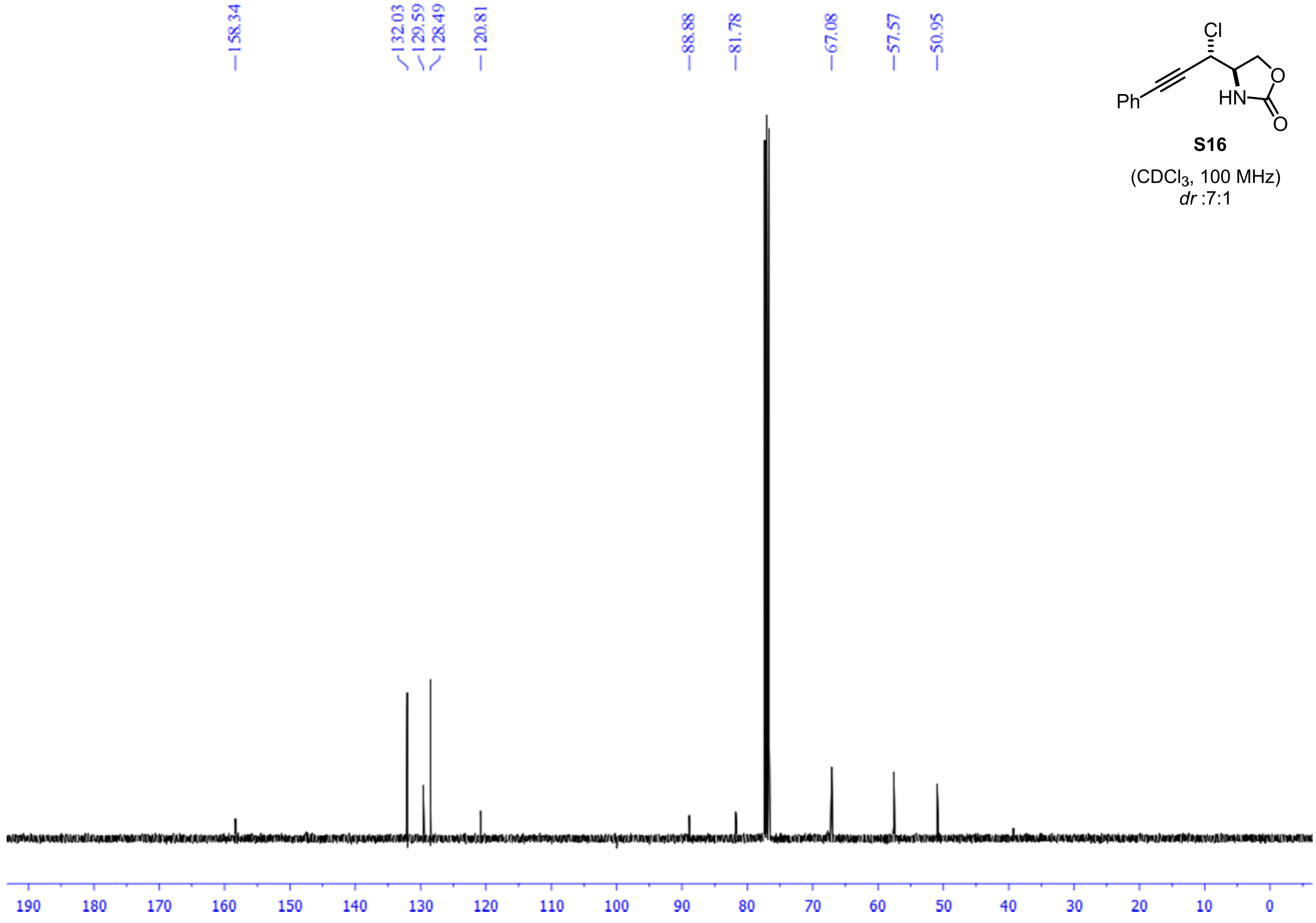

S16

($CDCl_3$, 100 MHz)

dr :7:1

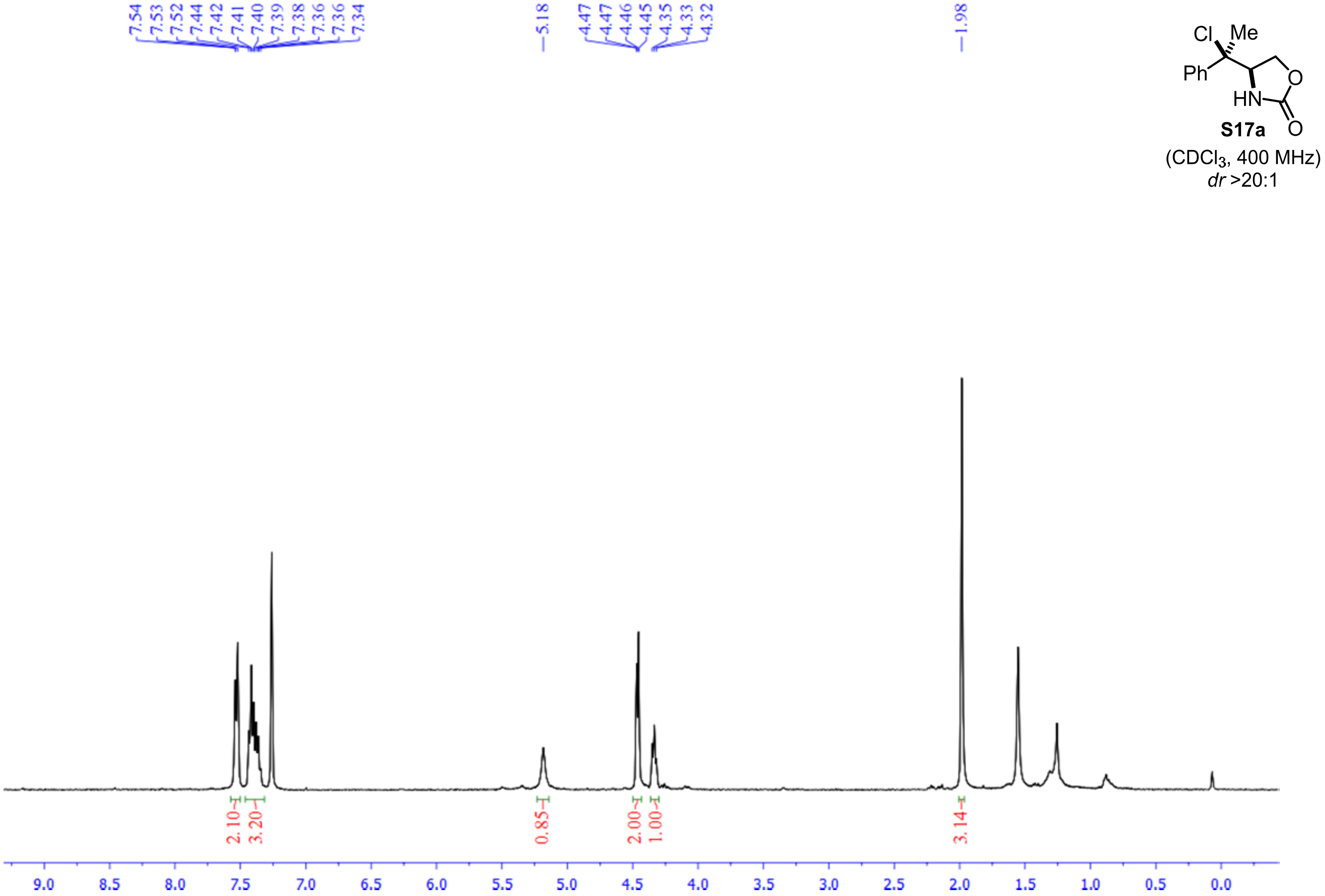

S17a
($CDCl_3$, 400 MHz)
dr >20:1

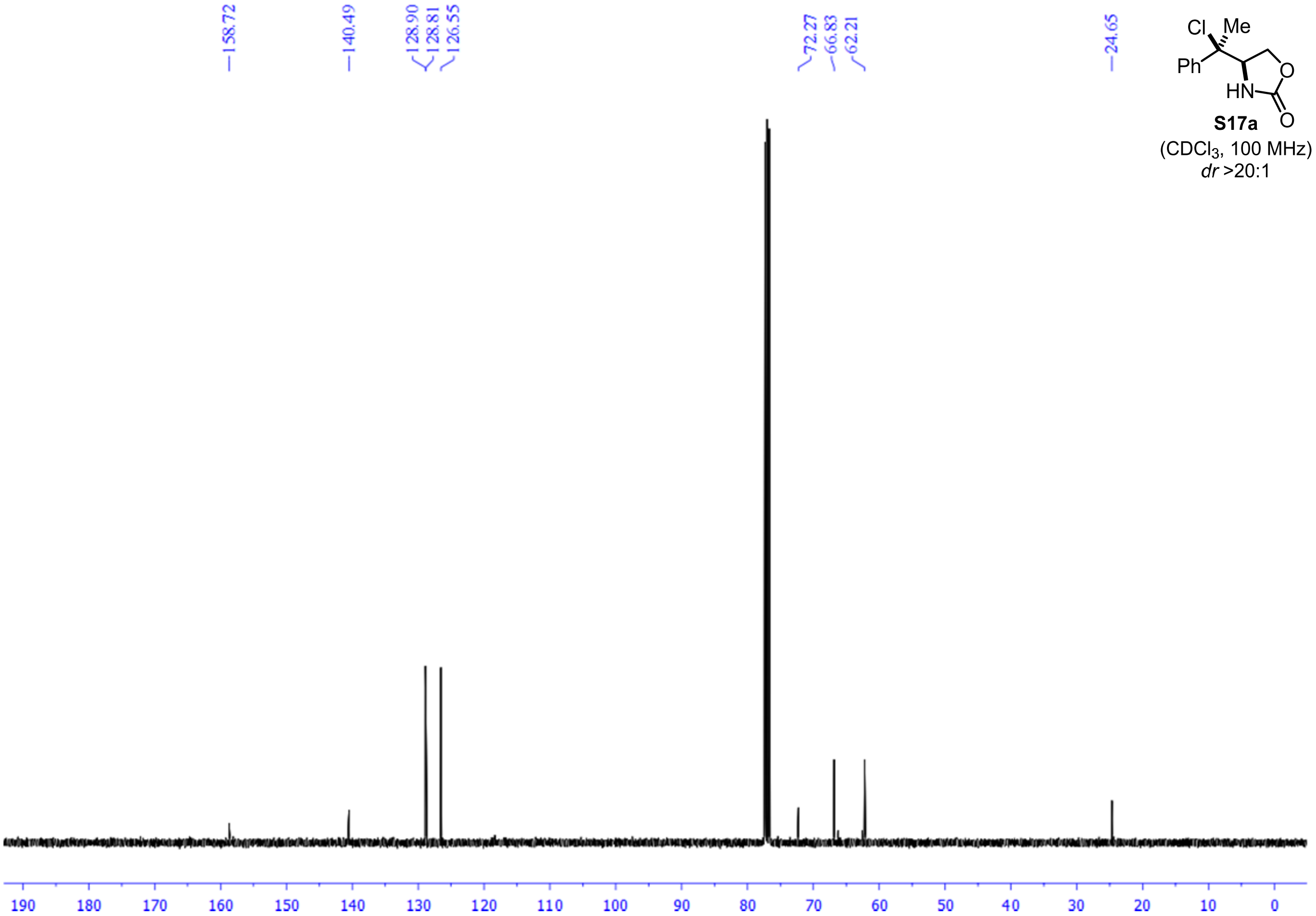

S17a
($CDCl_3$, 100 MHz)
dr >20:1

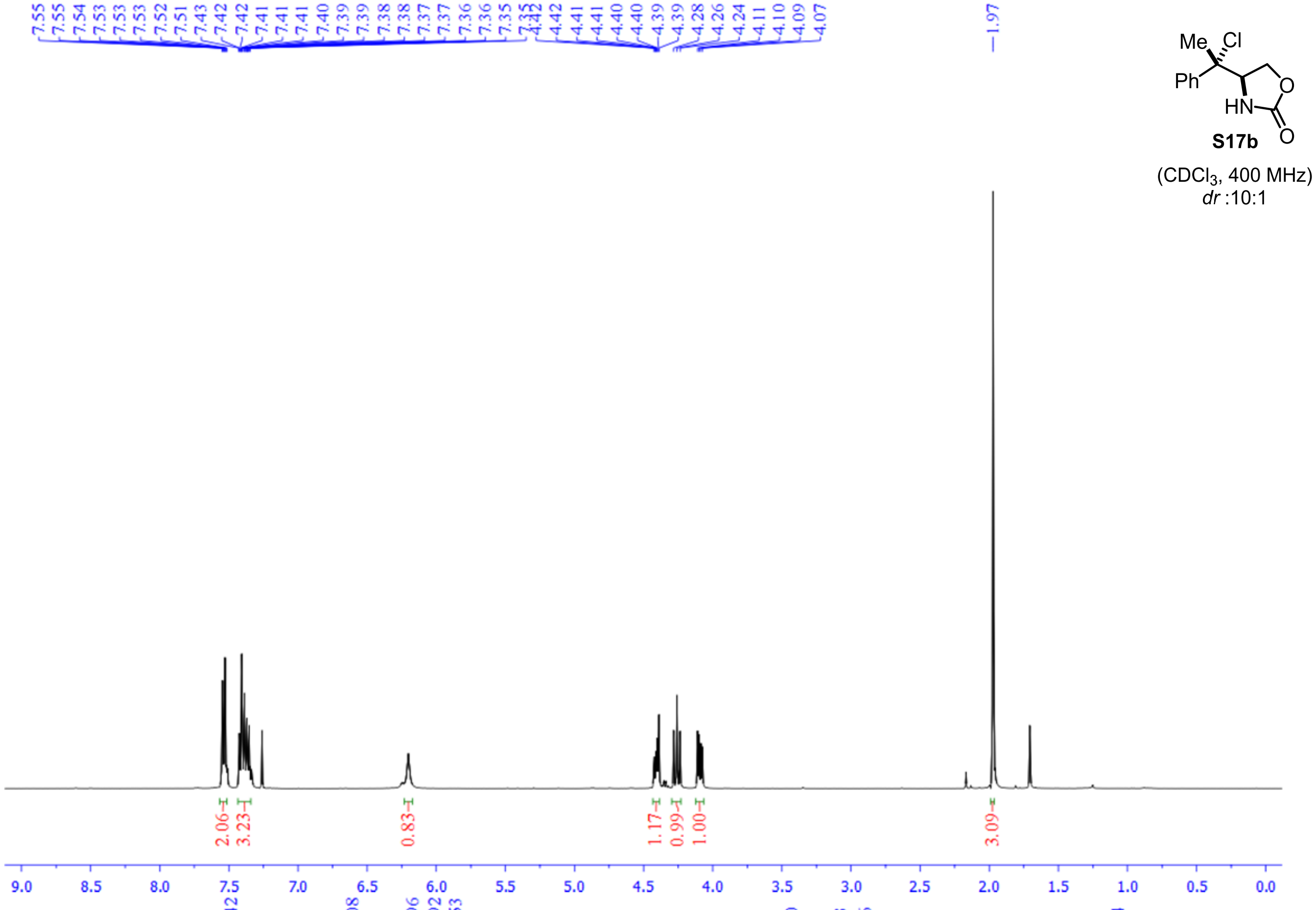
Me
Cl
Ph
HN
O
O
S17b
(CDCl3, 400 MHz)
dr :10:1
7.55 7.55 7.54 7.53 7.53 7.53 7.52 7.51 7.43 7.42 7.42 7.41 7.41 7.41 7.40 7.39 7.39 7.38 7.38 7.37 7.37 7.36 7.36 7.35 7.35 4.42 4.42 4.41 4.41 4.40 4.40 4.39 4.39 4.28 4.26 4.24 4.11 4.10 4.09 4.07
1.97
2.06 3.23 0.83 1.17 0.99 1.00 3.09

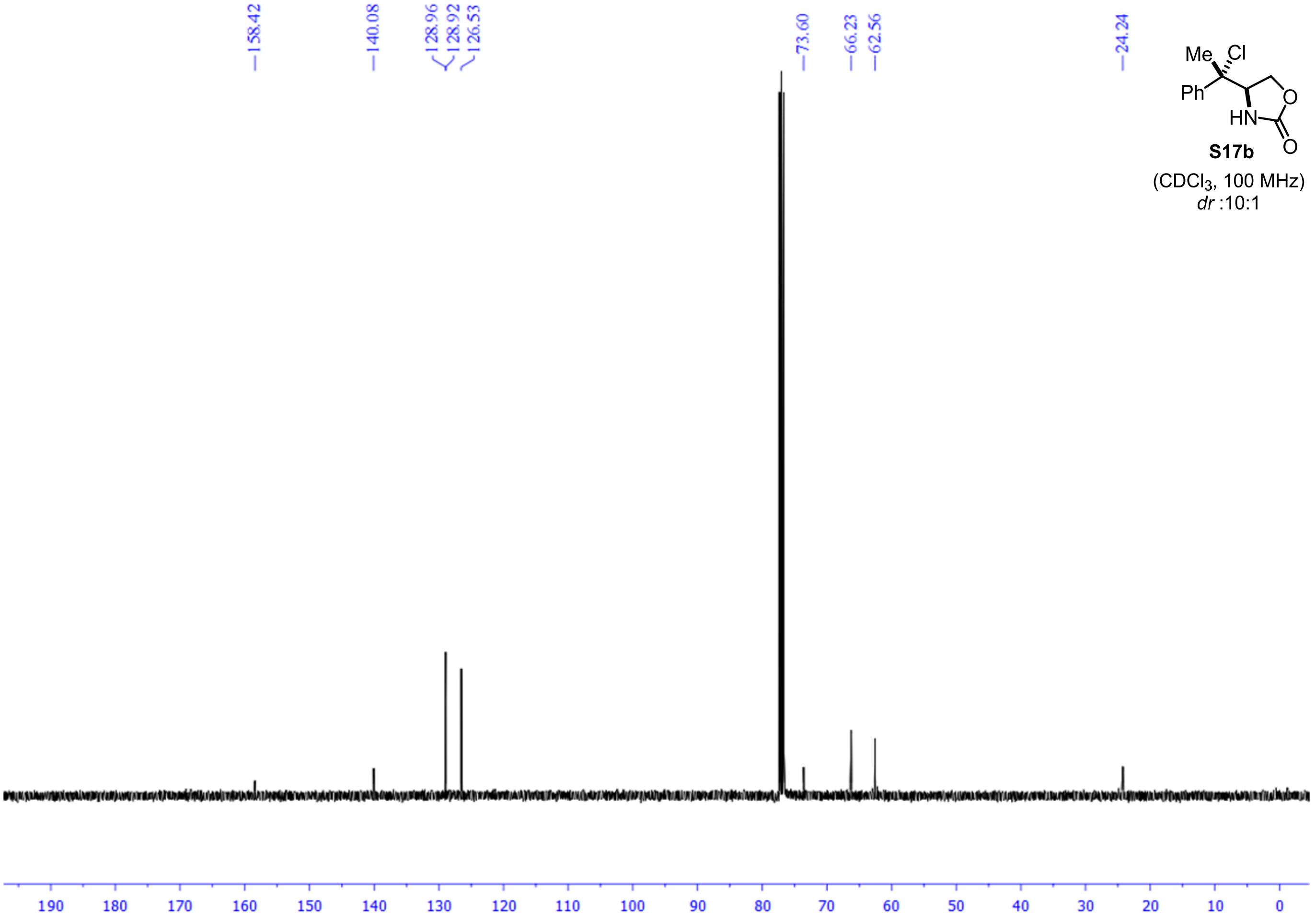
Me
Cl
Ph
HN
O
O
S17b
(CDCl3, 100 MHz)
dr :10:1
158.42
140.08
128.96 128.92 126.53
73.60
66.23 62.56
24.24

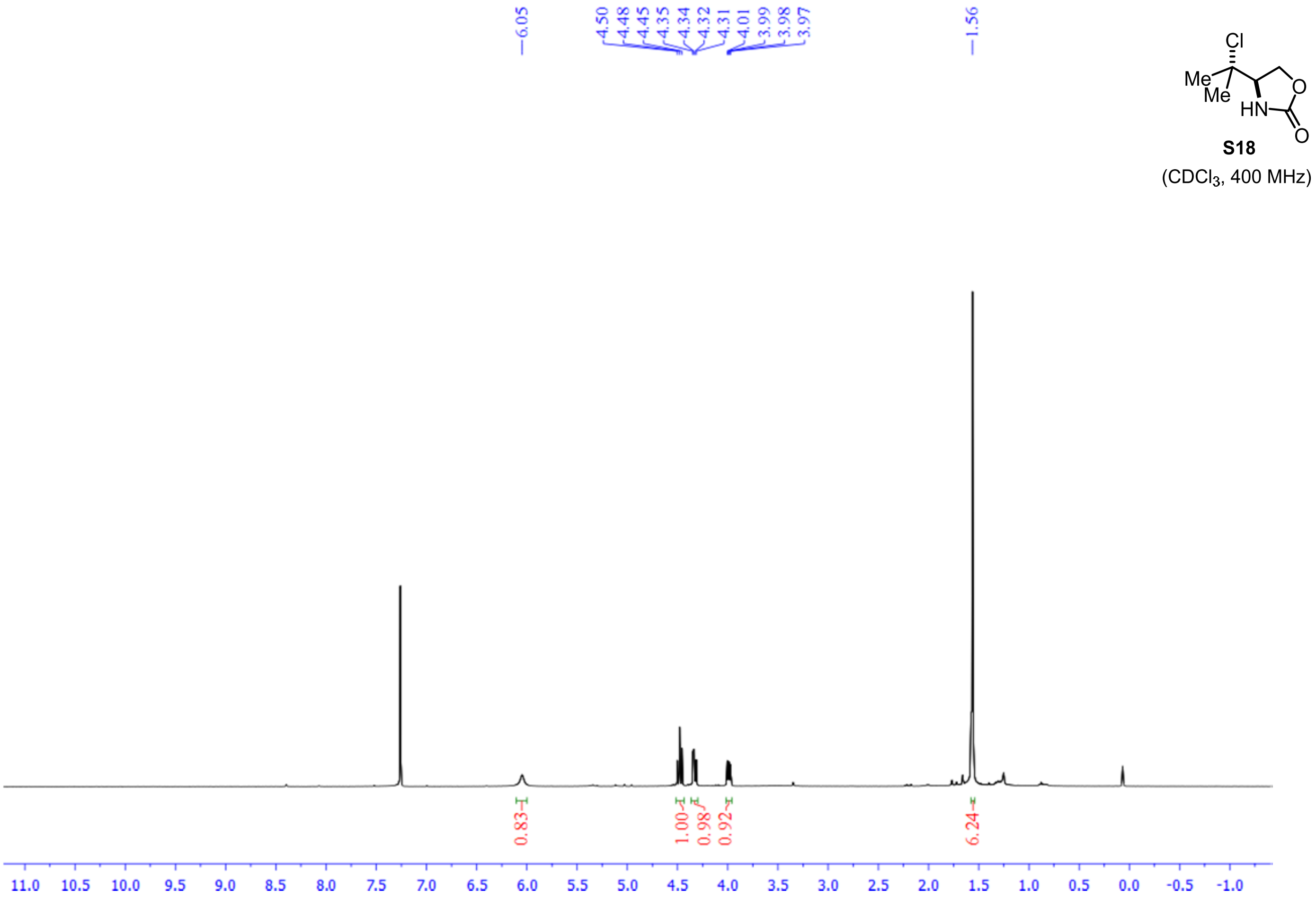
6.05
4.50
4.48
4.45
4.35
4.34
4.32
4.31
4.01
3.99
3.98
3.97
1.56
Cl
Me
Me
HN
O
O
S18
(CDCl$_3$, 400 MHz)
0.83
1.00
0.98
0.92
6.24
11.0 10.5 10.0 9.5 9.0 8.5 8.0 7.5 7.0 6.5 6.0 5.5 5.0 4.5 4.0 3.5 3.0 2.5 2.0 1.5 1.0 0.5 0.0 -0.5 -1.0

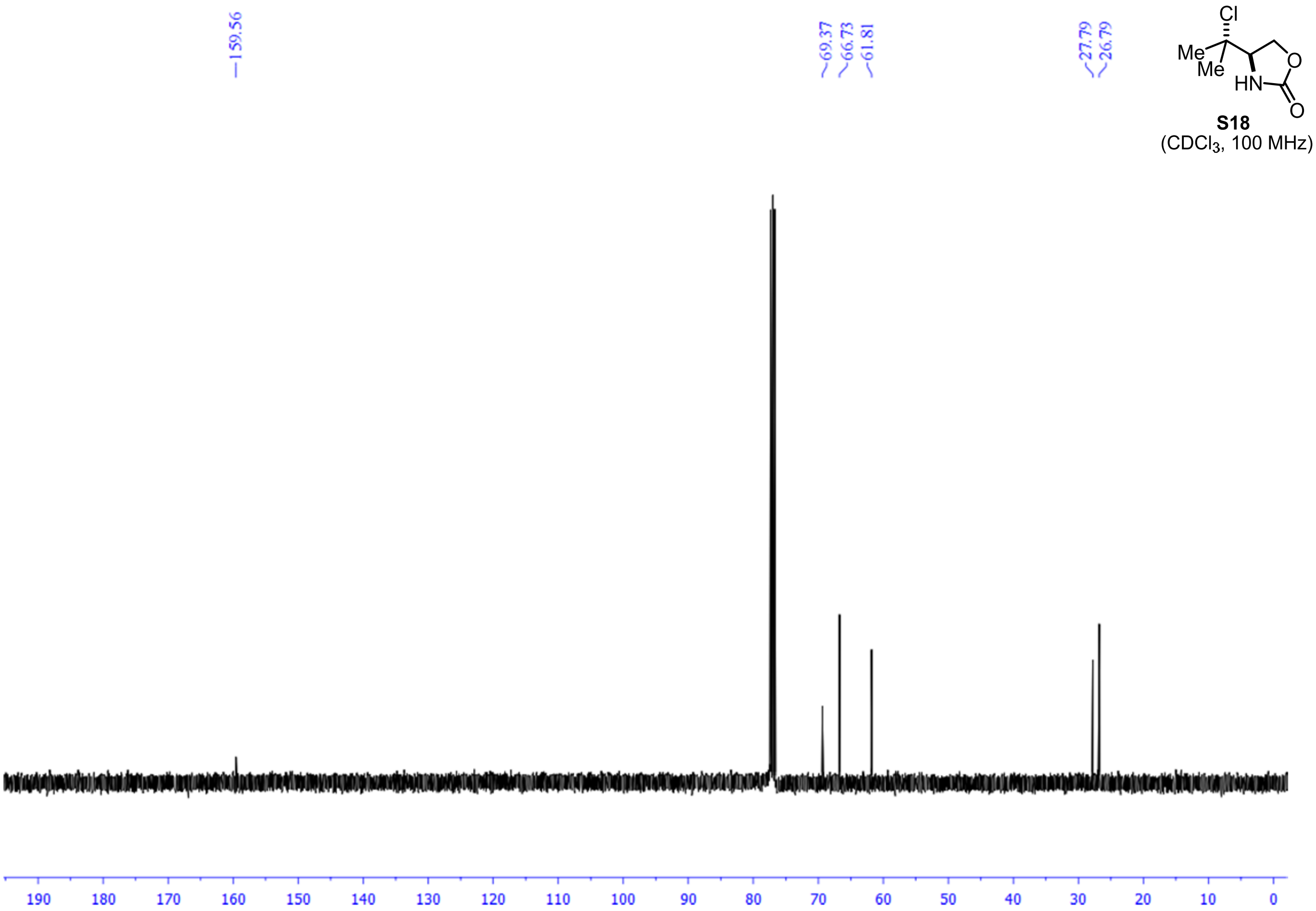
159.56
69.37
66.73
61.81
27.79
26.79
Cl
Me
Me
HN
O
O
S18
(CDCl$_3$, 100 MHz)
190 180 170 160 150 140 130 120 110 100 90 80 70 60 50 40 30 20 10 0

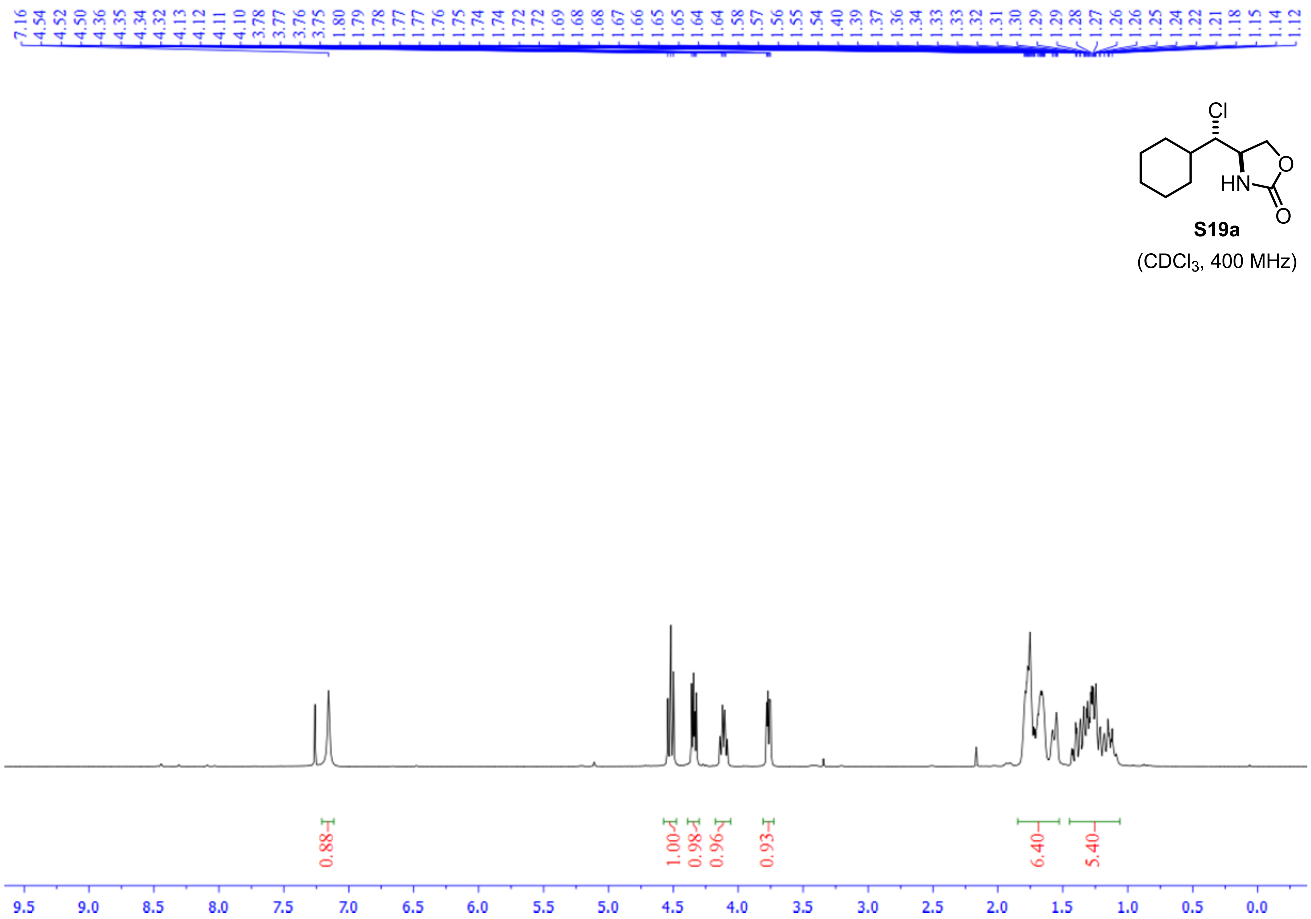
7.16 4.54 4.52 4.50 4.36 4.35 4.34 4.32 4.13 4.12 4.11 4.10 3.78 3.77 3.76 3.75 1.80 1.79 1.78 1.77 1.77 1.76 1.75 1.74 1.74 1.72 1.72 1.69 1.68 1.68 1.67 1.66 1.65 1.65 1.64 1.64 1.58 1.57 1.56 1.55 1.54 1.40 1.39 1.37 1.36 1.34 1.33 1.33 1.32 1.31 1.30 1.29 1.29 1.28 1.27 1.26 1.26 1.25 1.24 1.22 1.21 1.18 1.15 1.14 1.12
Cl
HN
O
O
S19a
(CDCl3, 400 MHz)
0.88
1.00
0.98
0.96
0.93
6.40
5.40
9.5 9.0 8.5 8.0 7.5 7.0 6.5 6.0 5.5 5.0 4.5 4.0 3.5 3.0 2.5 2.0 1.5 1.0 0.5 0.0

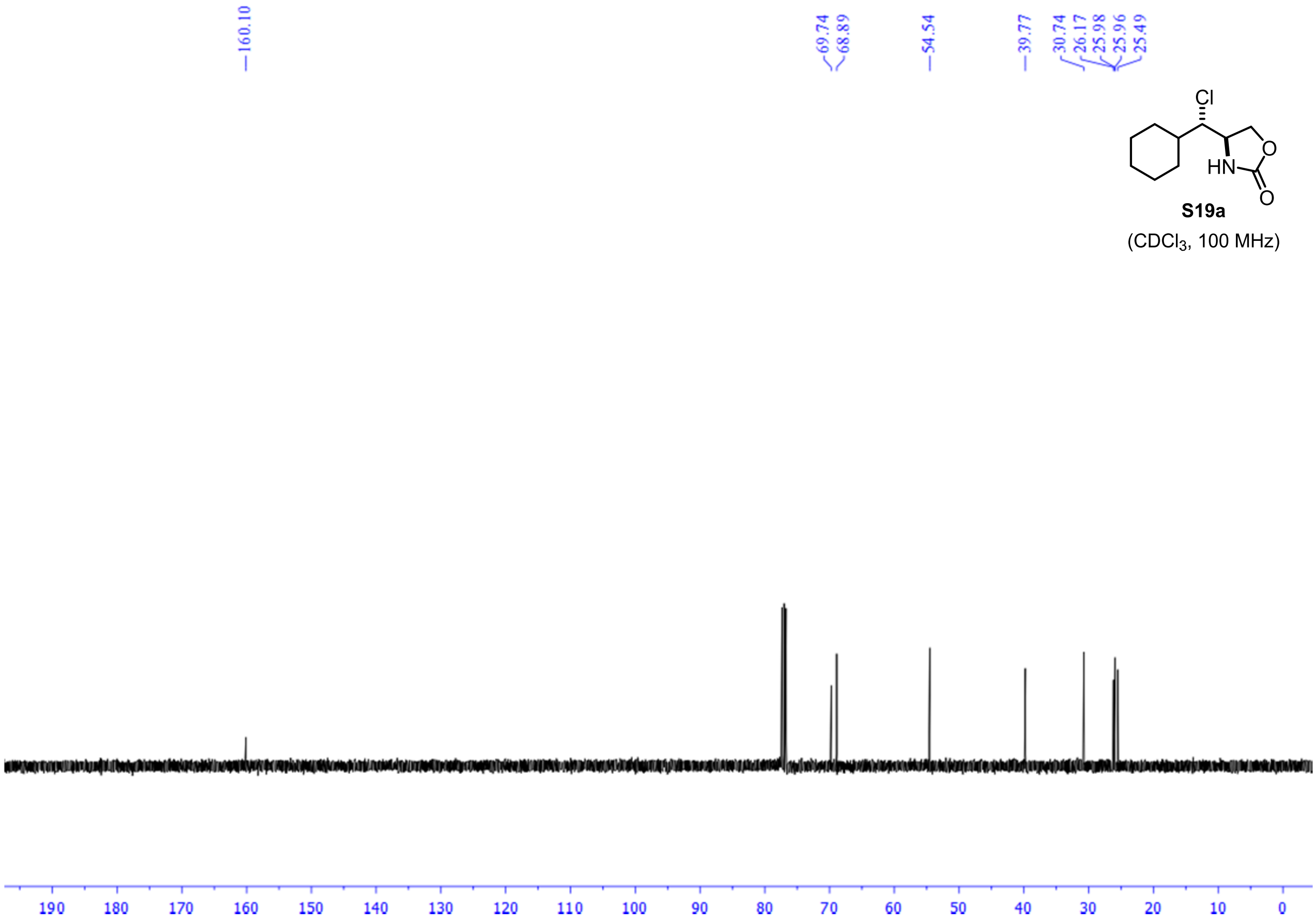
160.10
69.74 68.89
54.54
39.77
30.74 26.17 25.98 25.96 25.49
Cl
HN
O
O
S19a
(CDCl3, 100 MHz)
190 180 170 160 150 140 130 120 110 100 90 80 70 60 50 40 30 20 10 0

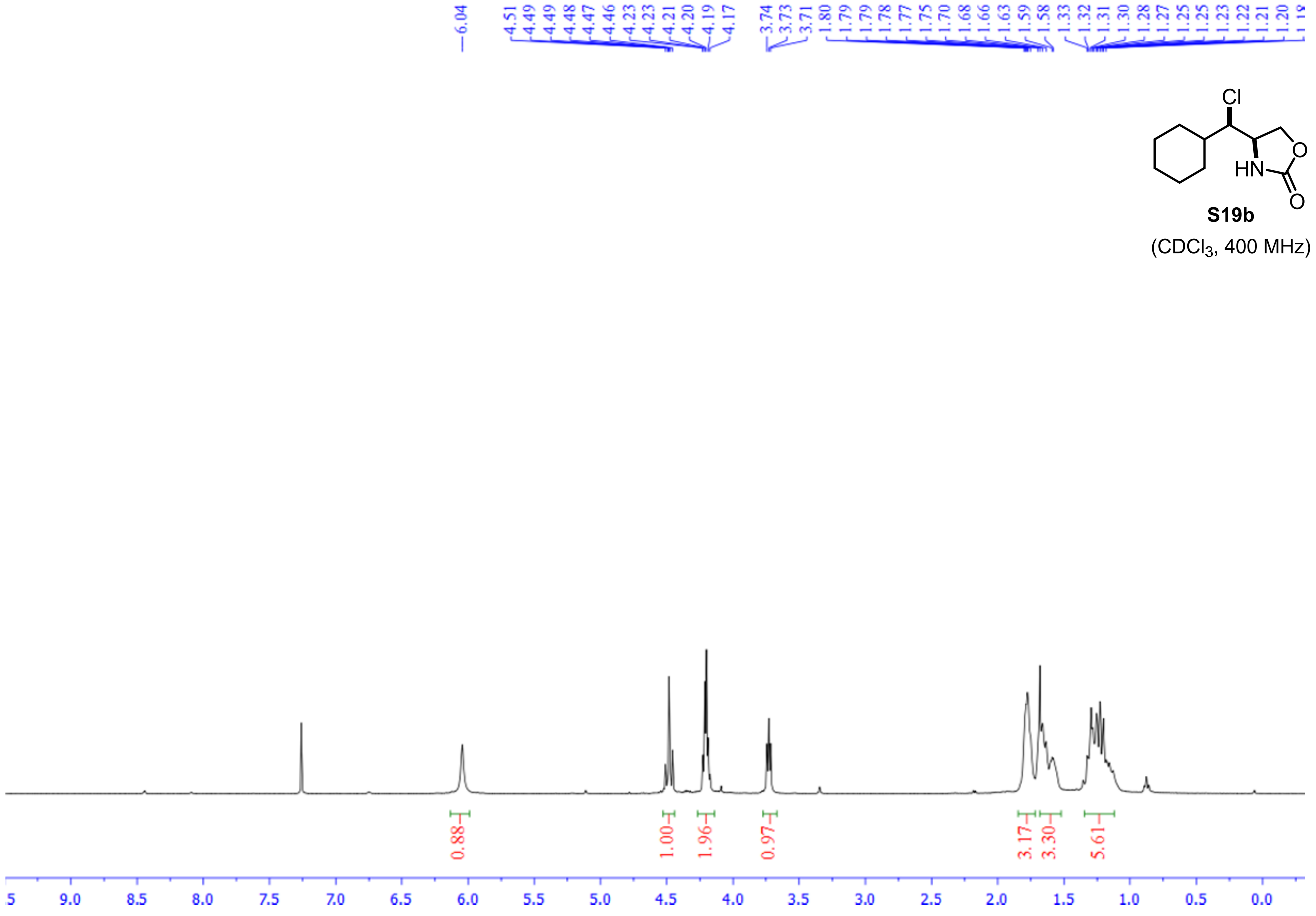

6.04
4.51
4.49
4.49
4.48
4.47
4.46
4.23
4.23
4.21
4.20
4.19
4.17
3.74
3.73
3.71
1.80
1.79
1.79
1.78
1.77
1.75
1.70
1.68
1.66
1.63
1.59
1.58
1.33
1.32
1.31
1.30
1.28
1.27
1.25
1.25
1.23
1.22
1.21
1.20
Cl
HN
O
O
S19b
(CDCl3, 400 MHz)
0.88
1.00
1.96
0.97
3.17
3.30
5.61
9.0
8.5
8.0
7.5
7.0
6.5
6.0
5.5
5.0
4.5
4.0
3.5
3.0
2.5
2.0
1.5
1.0
0.5
0.0

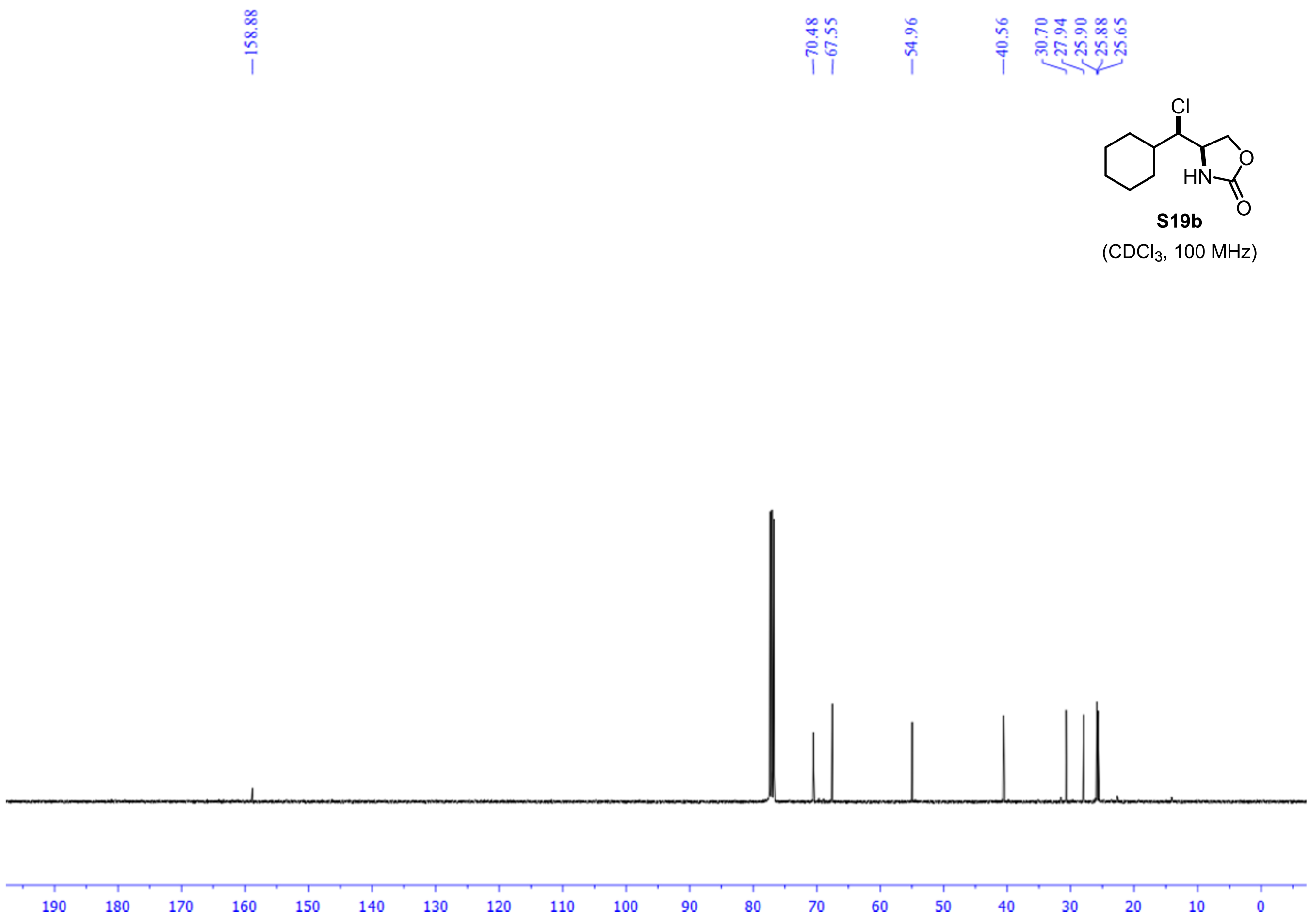

158.88
70.48
67.55
54.96
40.56
30.70
27.94
25.90
25.88
25.65
Cl
HN
O
O
S19b
(CDCl3, 100 MHz)
190
180
170
160
150
140
130
120
110
100
90
80
70
60
50
40
30
20
10
0

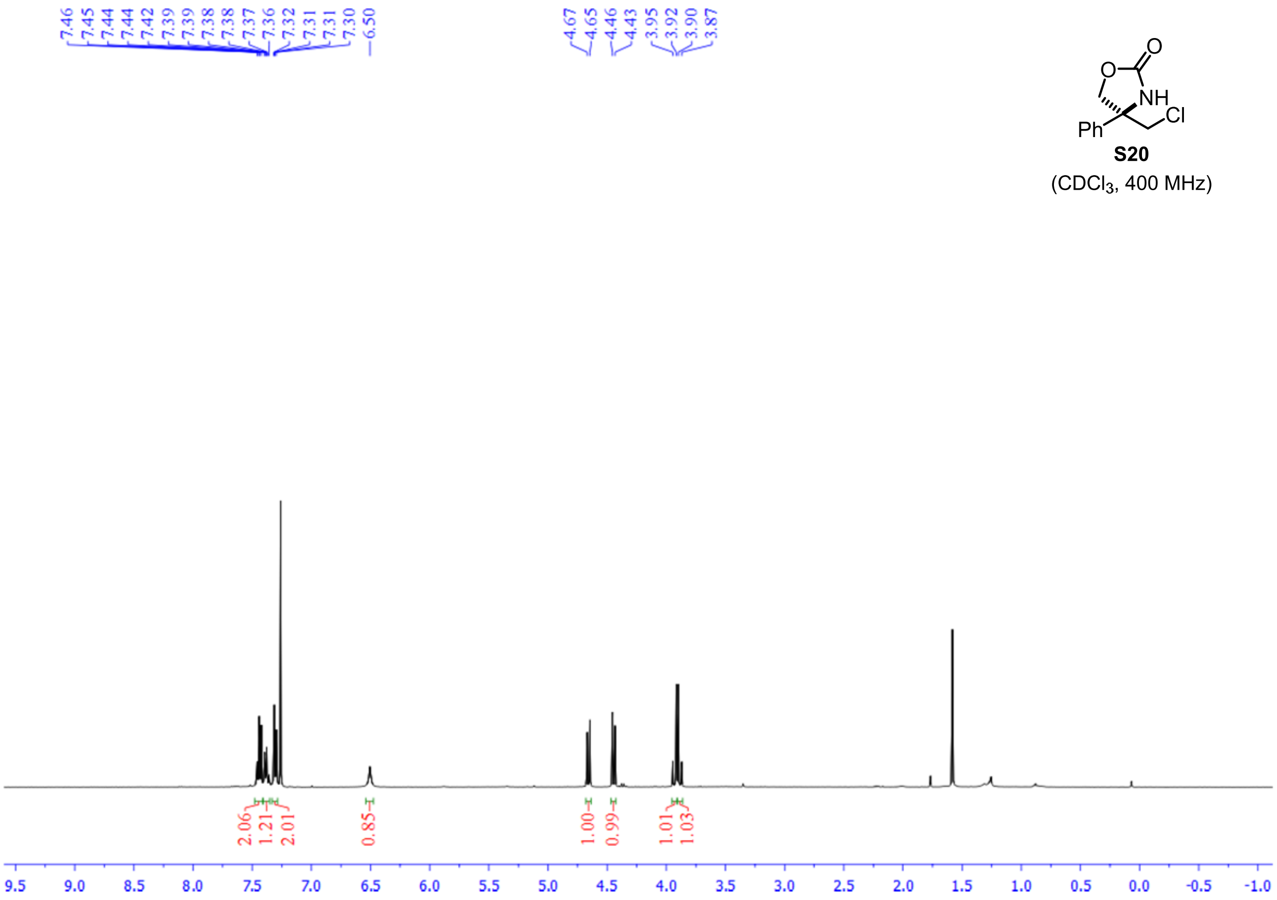
7.46
7.45
7.44
7.44
7.42
7.39
7.39
7.38
7.38
7.37
7.36
7.32
7.31
7.31
7.30
6.50
4.67
4.65
4.46
4.43
3.95
3.92
3.90
3.87
O
O
NH
Cl
Ph
S20
(CDCl3, 400 MHz)
2.06
1.21
2.01
0.85
1.00
0.99
1.01
1.03
9.5
9.0
8.5
8.0
7.5
7.0
6.5
6.0
5.5
5.0
4.5
4.0
3.5
3.0
2.5
2.0
1.5
1.0
0.5
0.0
-0.5
-1.0

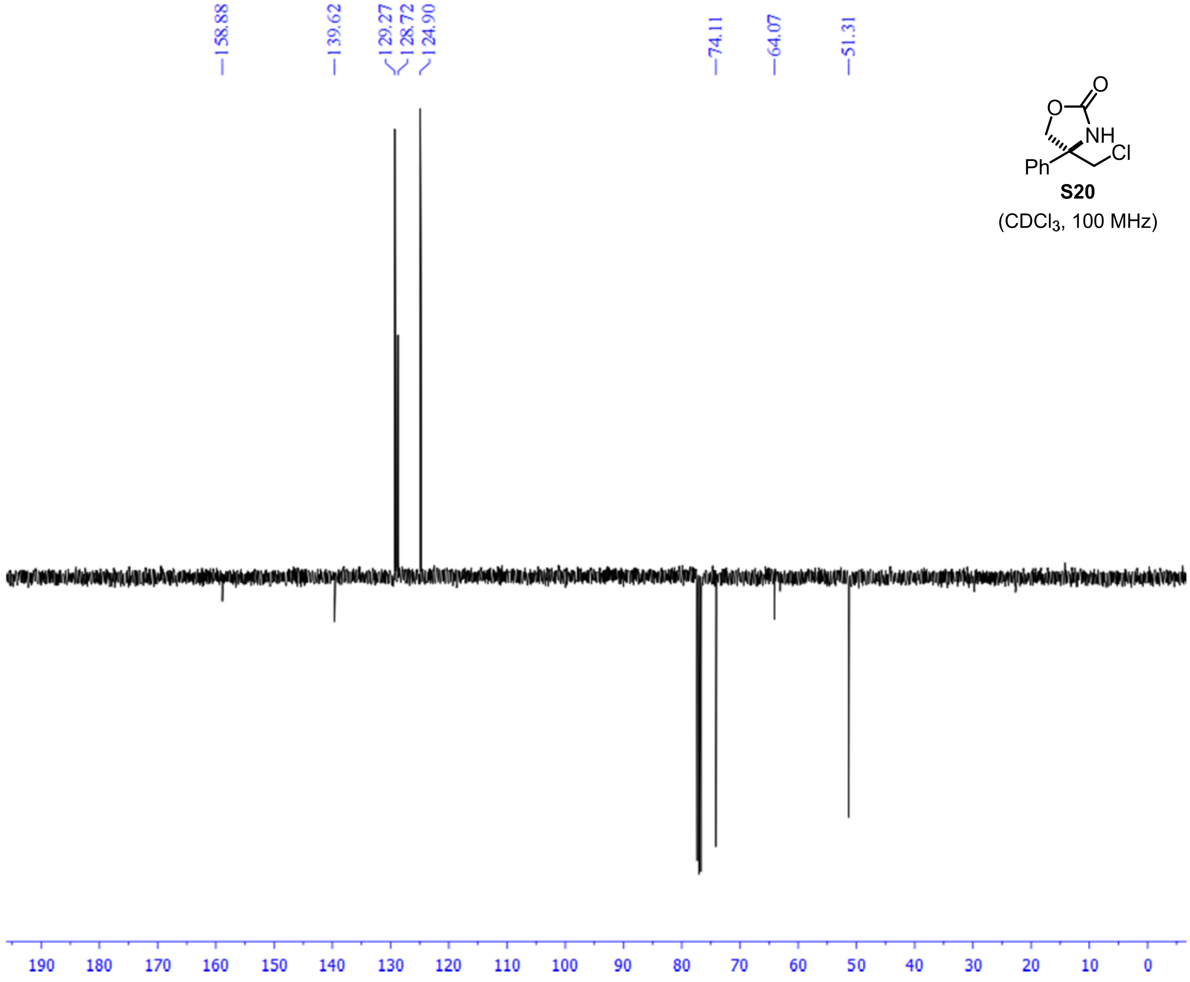
158.88
139.62
129.27
128.72
124.90
74.11
64.07
51.31
O
O
NH
Cl
Ph
S20
(CDCl3, 100 MHz)
190
180
170
160
150
140
130
120
110
100
90
80
70
60
50
40
30
20
10
0

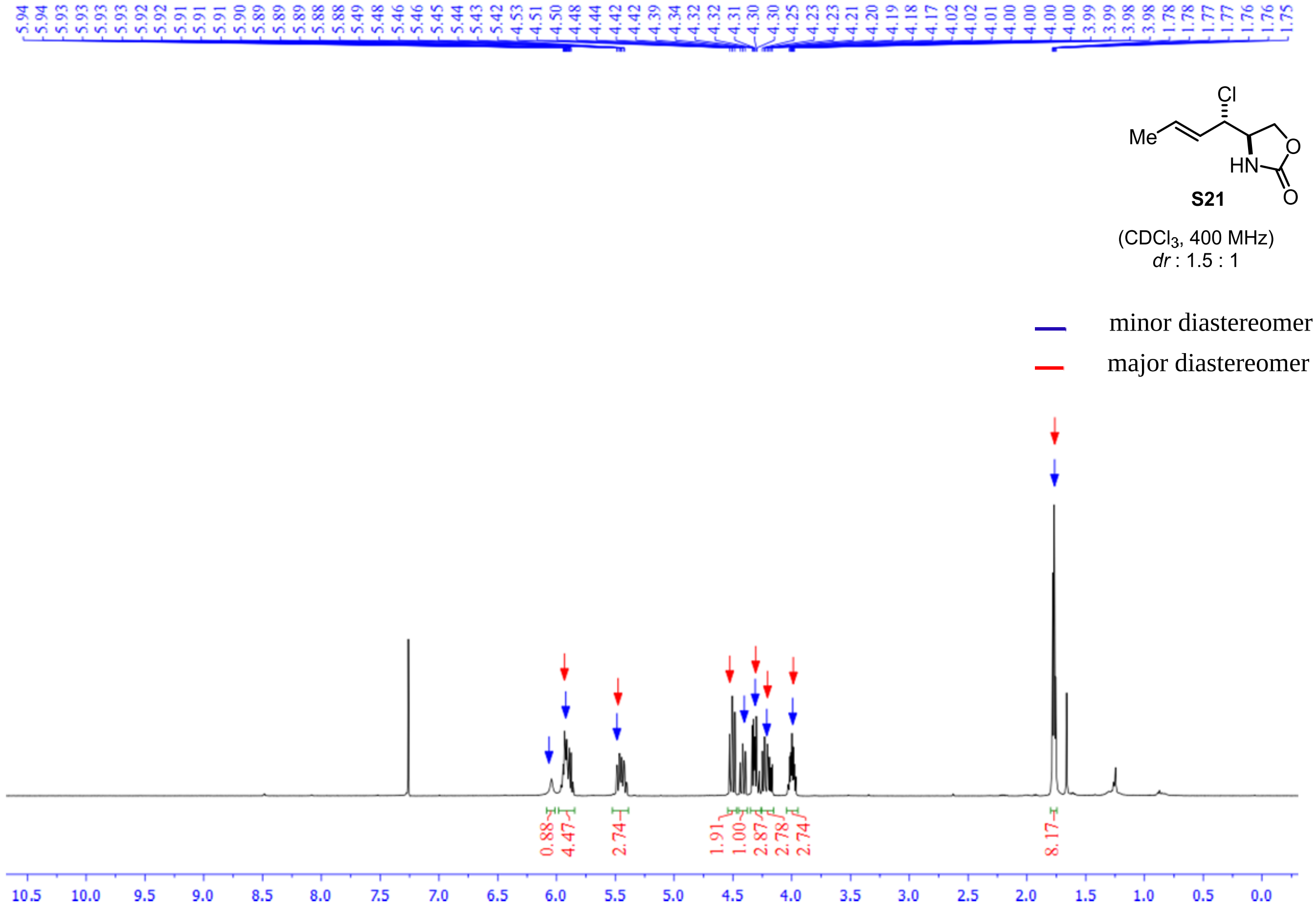
5.94 5.94 5.93 5.93 5.93 5.93 5.92 5.92 5.91 5.91 5.91 5.90 5.89 5.89 5.89 5.88 5.88 5.49 5.48 5.46 5.46 5.45 5.44 5.43 5.42 4.53 4.51 4.50 4.48 4.44 4.42 4.42 4.39 4.34 4.32 4.32 4.31 4.30 4.30 4.25 4.23 4.23 4.21 4.20 4.19 4.18 4.17 4.02 4.02 4.01 4.00 4.00 4.00 3.99 3.99 3.98 3.98 1.78 1.78 1.77 1.77 1.76 1.76 1.75
Cl
Me
HN
O
O
S21
($CDCl_3$, 400 MHz)
dr : 1.5 : 1
minor diastereomer
major diastereomer
0.88 4.47 2.74 1.91 1.00 2.87 2.78 2.74 8.17
10.5 10.0 9.5 9.0 8.5 8.0 7.5 7.0 6.5 6.0 5.5 5.0 4.5 4.0 3.5 3.0 2.5 2.0 1.5 1.0 0.5 0.0

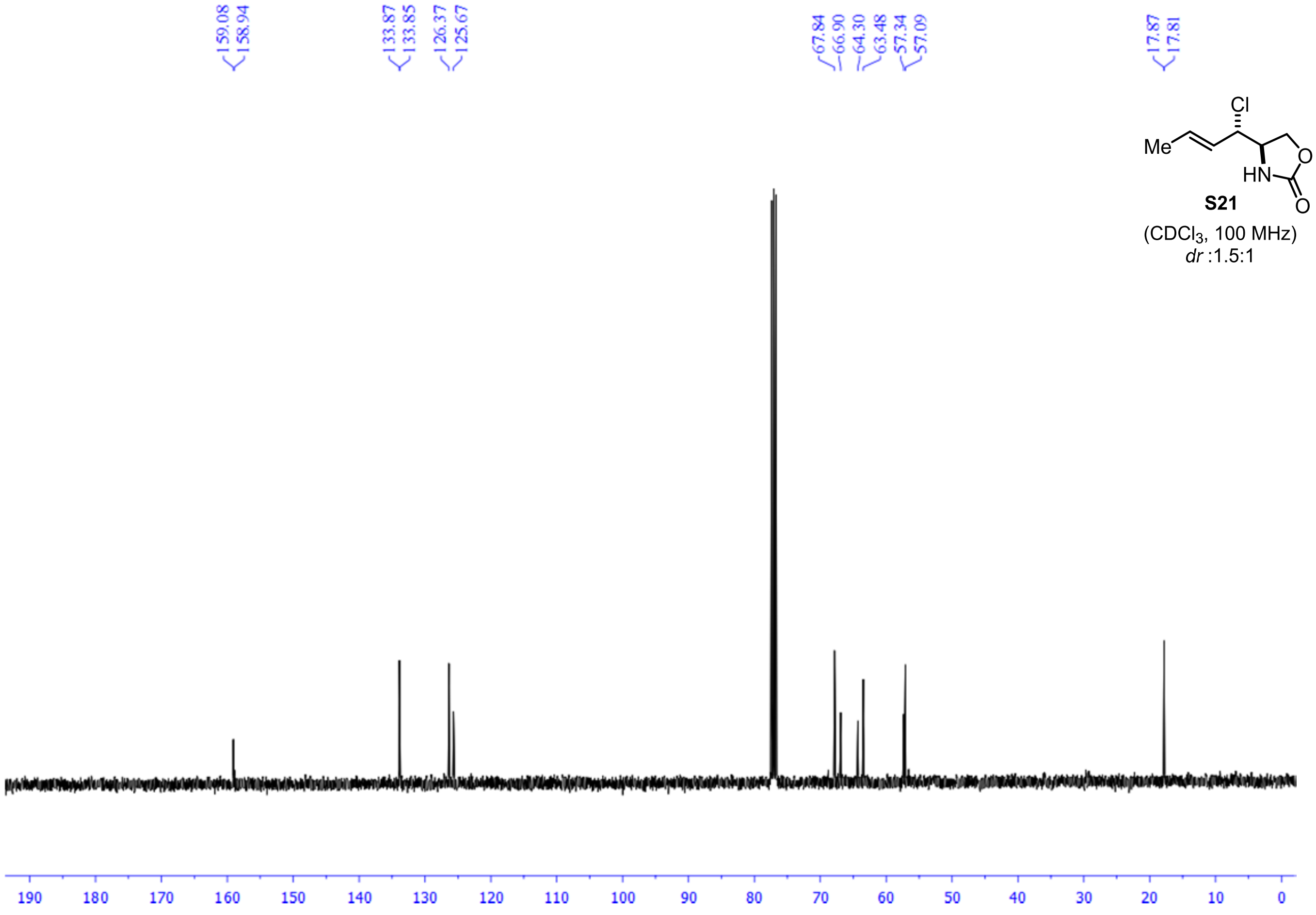
159.08 158.94 133.87 133.85 126.37 125.67 67.84 66.90 64.30 63.48 57.34 57.09 17.87 17.81
Cl
Me
HN
O
O
S21
($CDCl_3$, 100 MHz)
dr :1.5:1
190 180 170 160 150 140 130 120 110 100 90 80 70 60 50 40 30 20 10 0

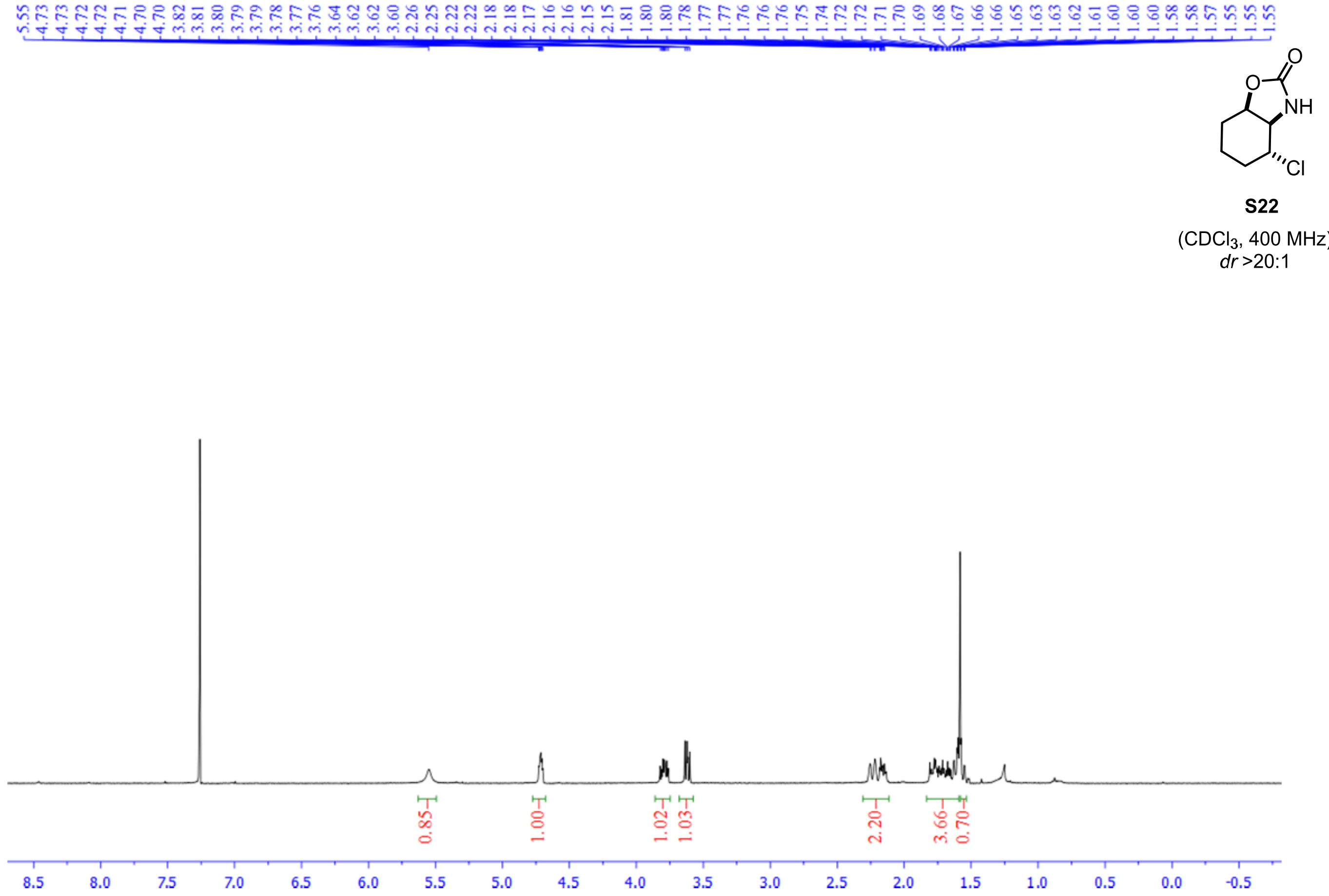

S22
(CDCl$_3$, 400 MHz)
dr >20:1

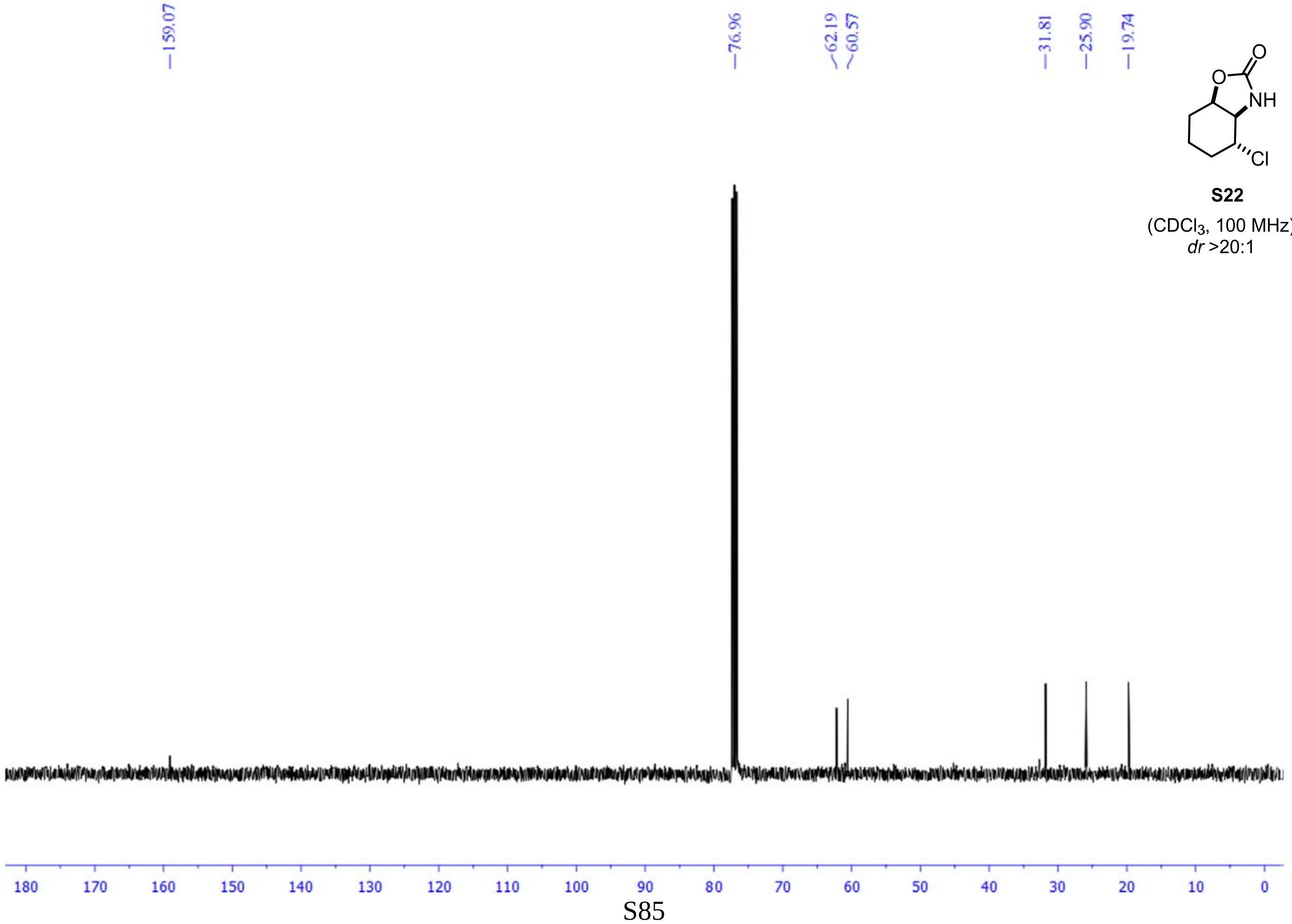

S22
(CDCl$_3$, 100 MHz)
dr >20:1

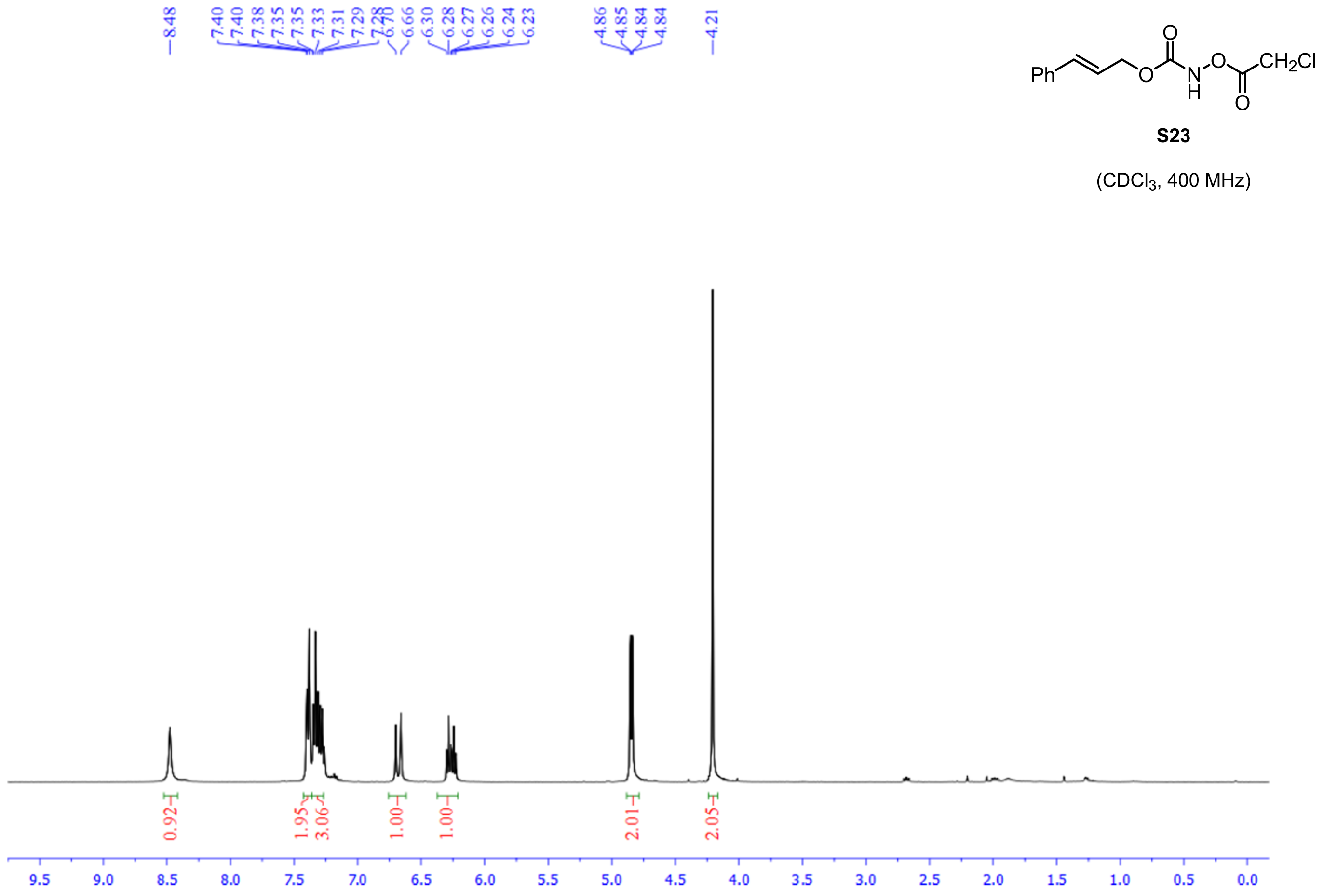

S23

($CDCl_3$, 400 MHz)

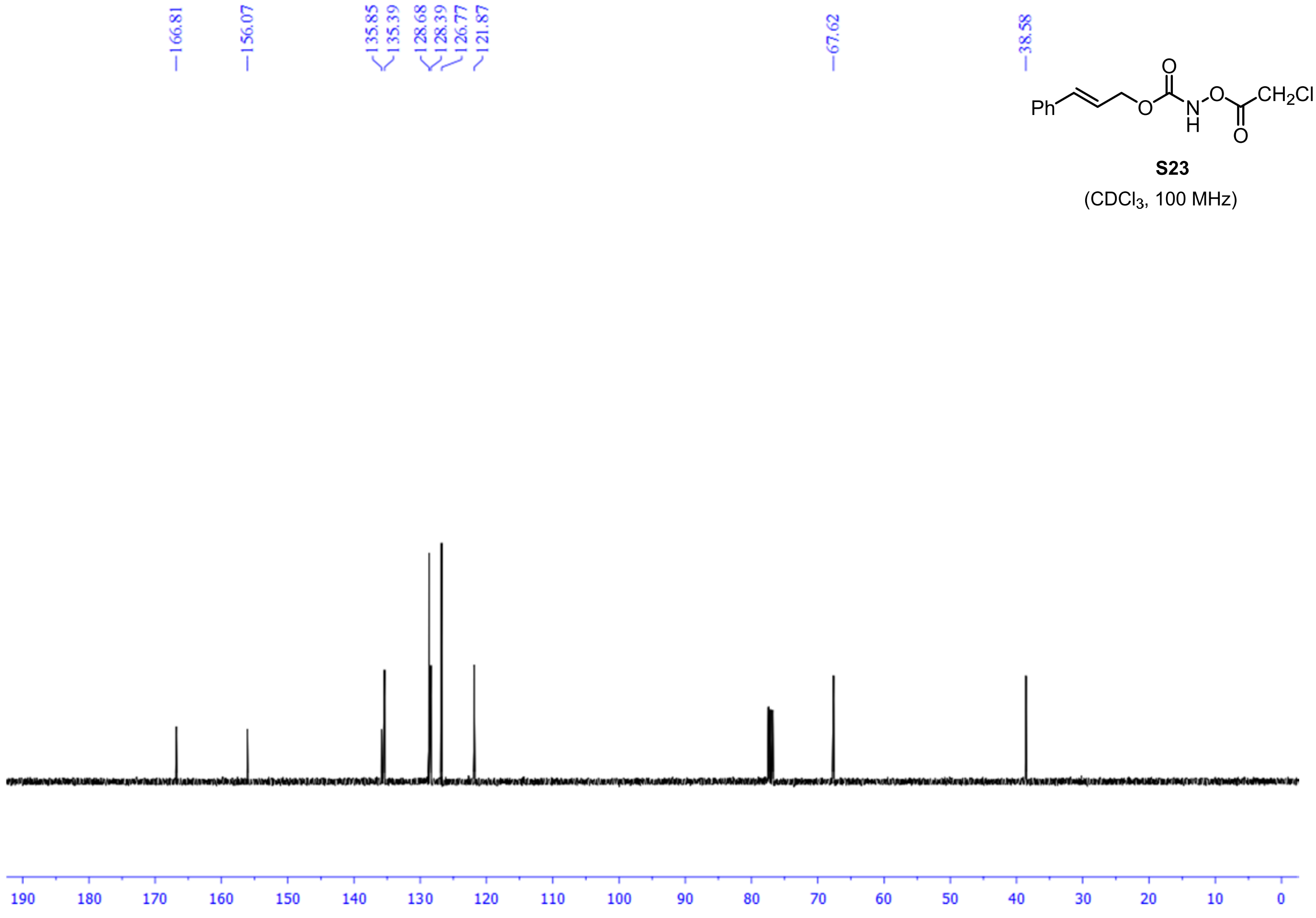

S23

($CDCl_3$, 100 MHz)

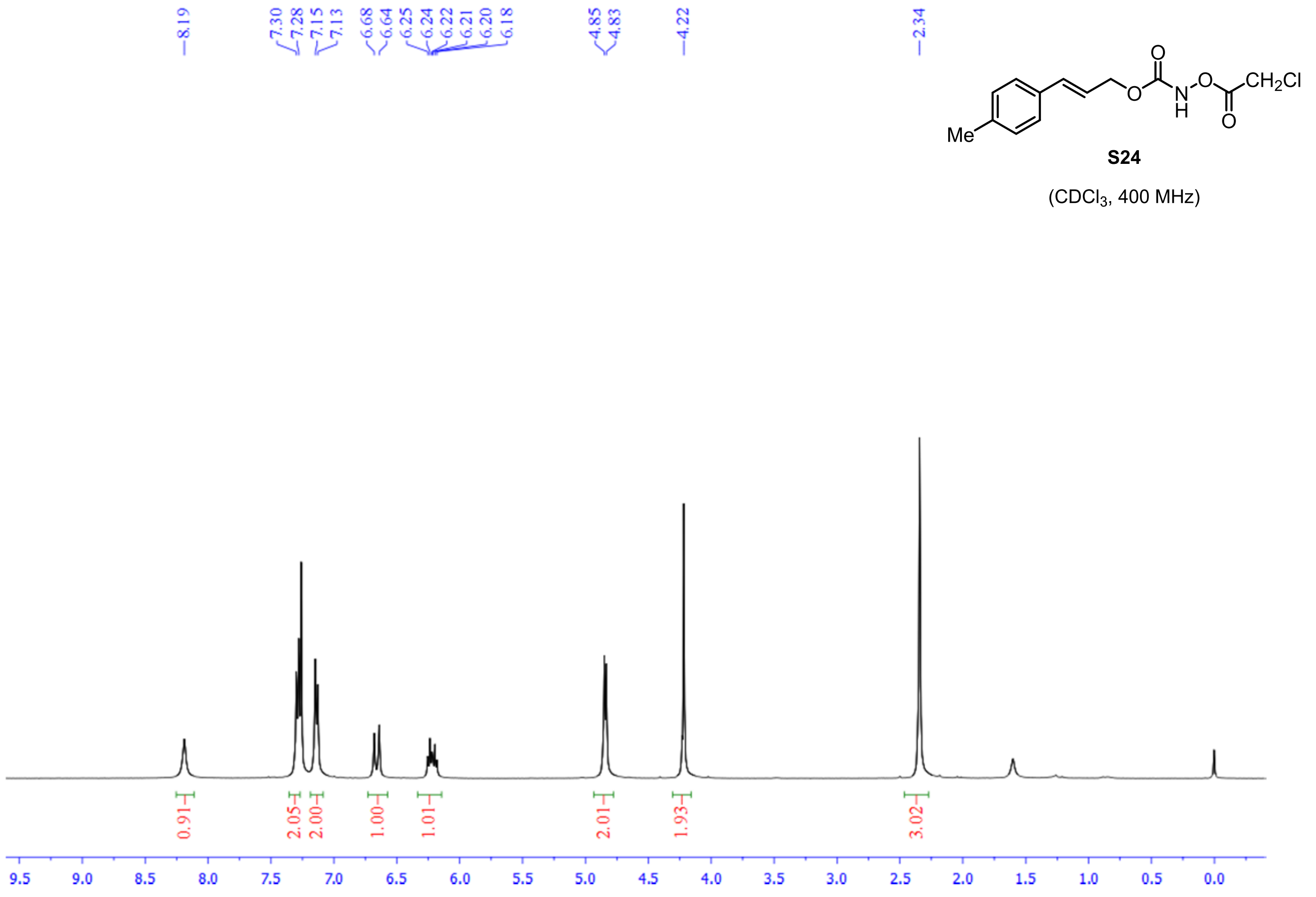
Me
O
N
H
O
CH2Cl
O
O
S24
(CDCl3, 400 MHz)
8.19
7.30
7.28
7.15
7.13
6.68
6.64
6.25
6.24
6.22
6.21
6.20
6.18
4.85
4.83
4.22
2.34
0.91
2.05
2.00
1.00
1.01
2.01
1.93
3.02

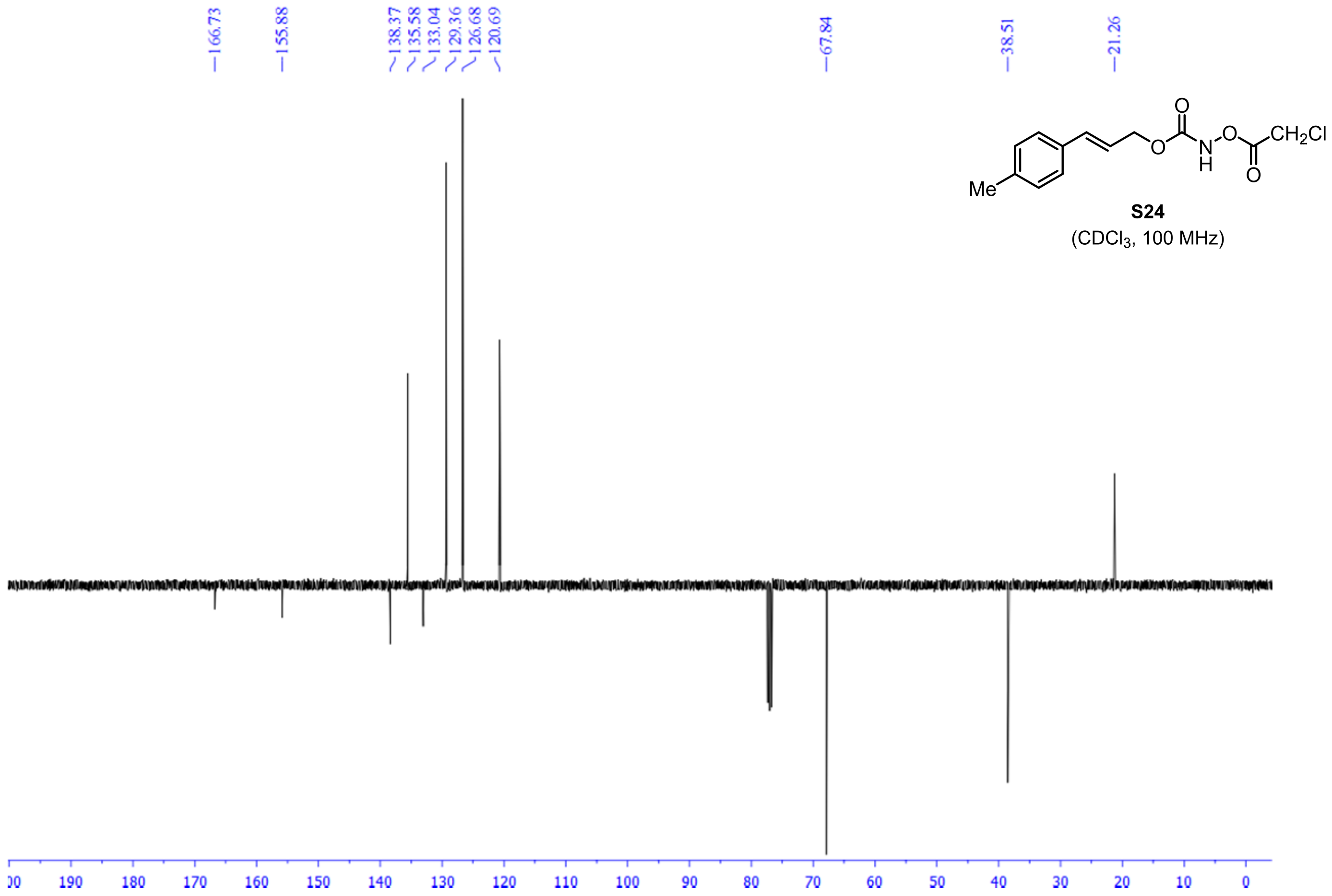
Me
O
N
H
O
CH2Cl
O
O
S24
(CDCl3, 100 MHz)
166.73
155.88
138.37
135.58
133.04
129.36
126.68
120.69
67.84
38.51
21.26

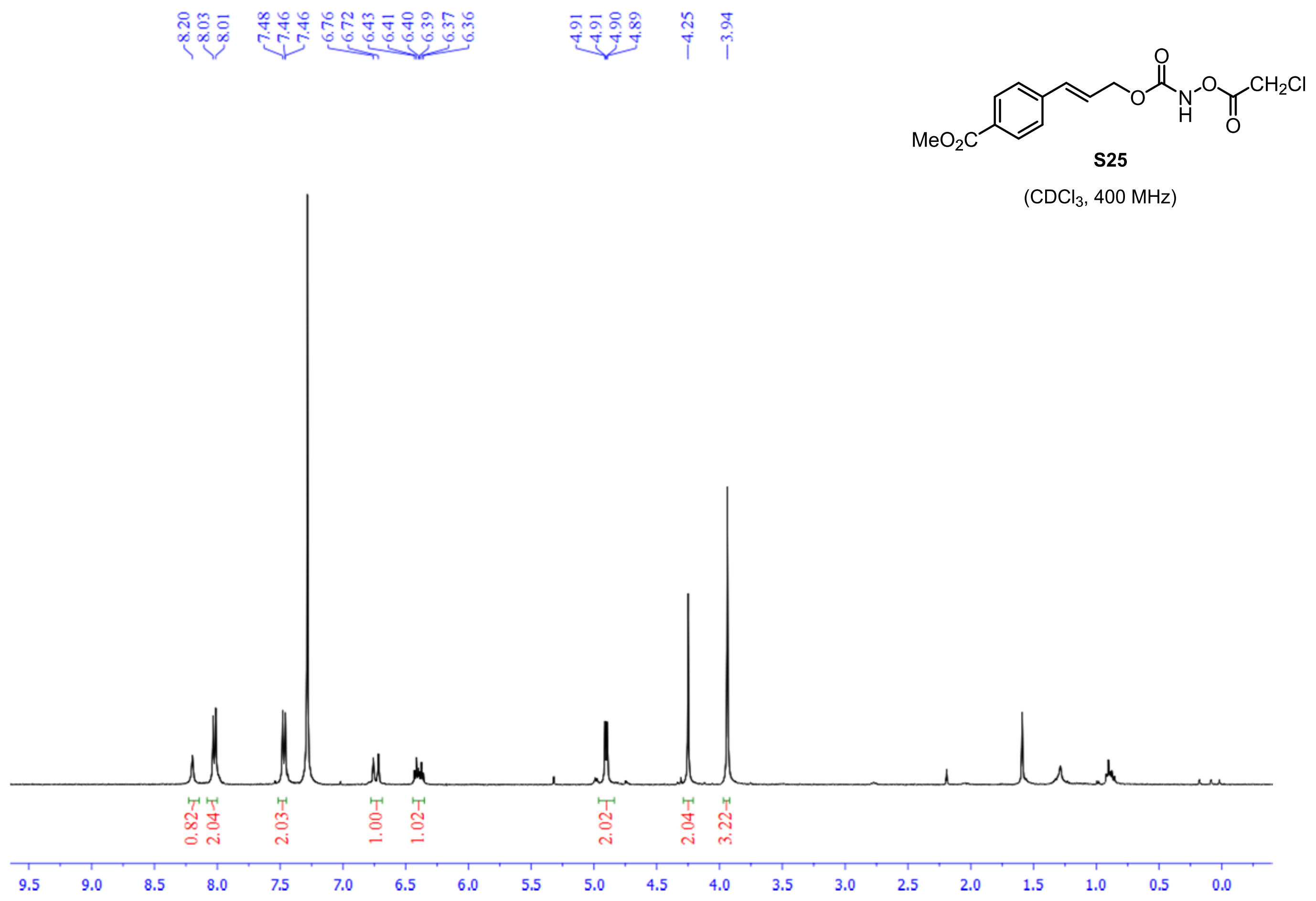
MeO2C
S25
(CDCl3, 400 MHz)

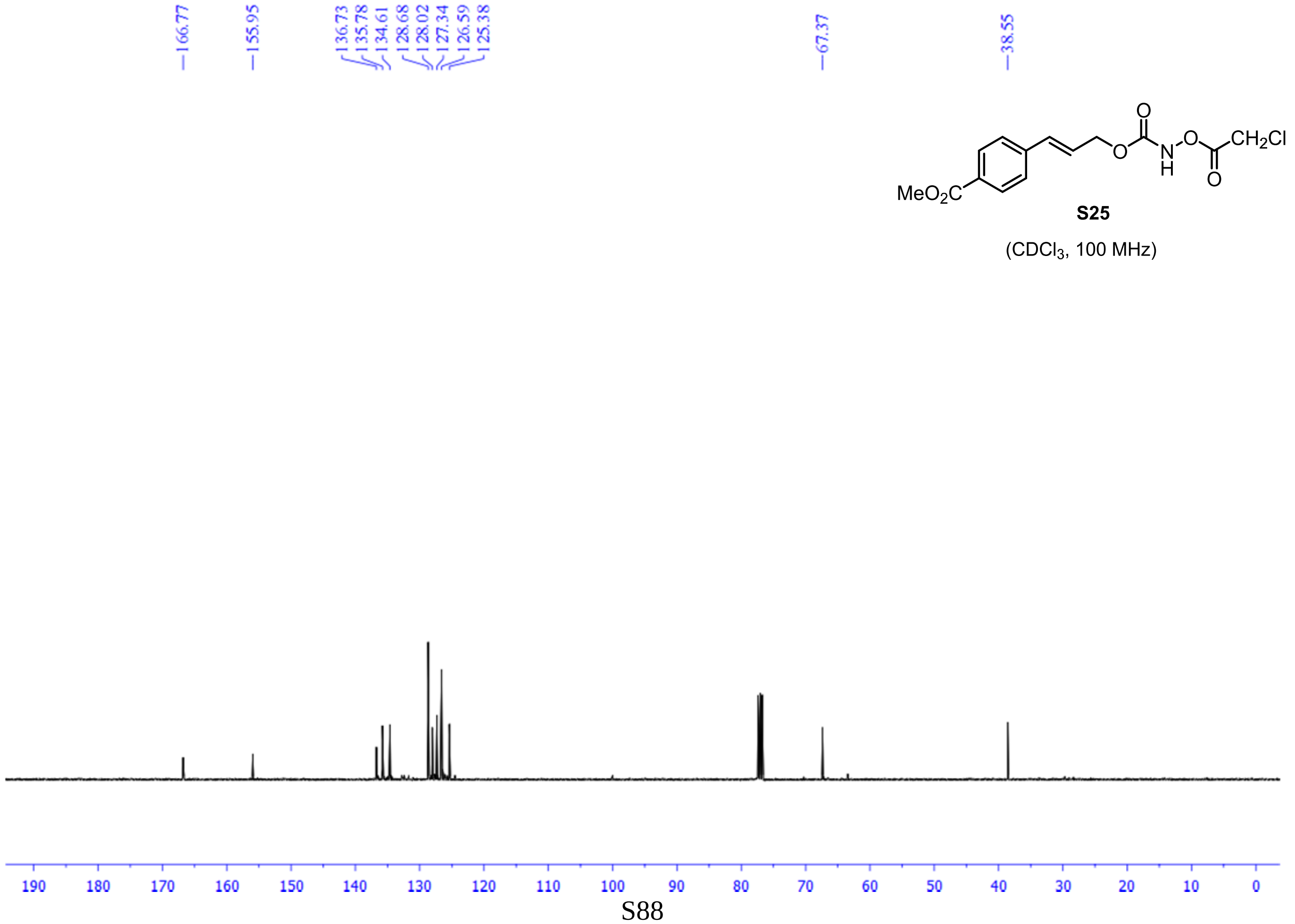
MeO2C
S25
(CDCl3, 100 MHz)

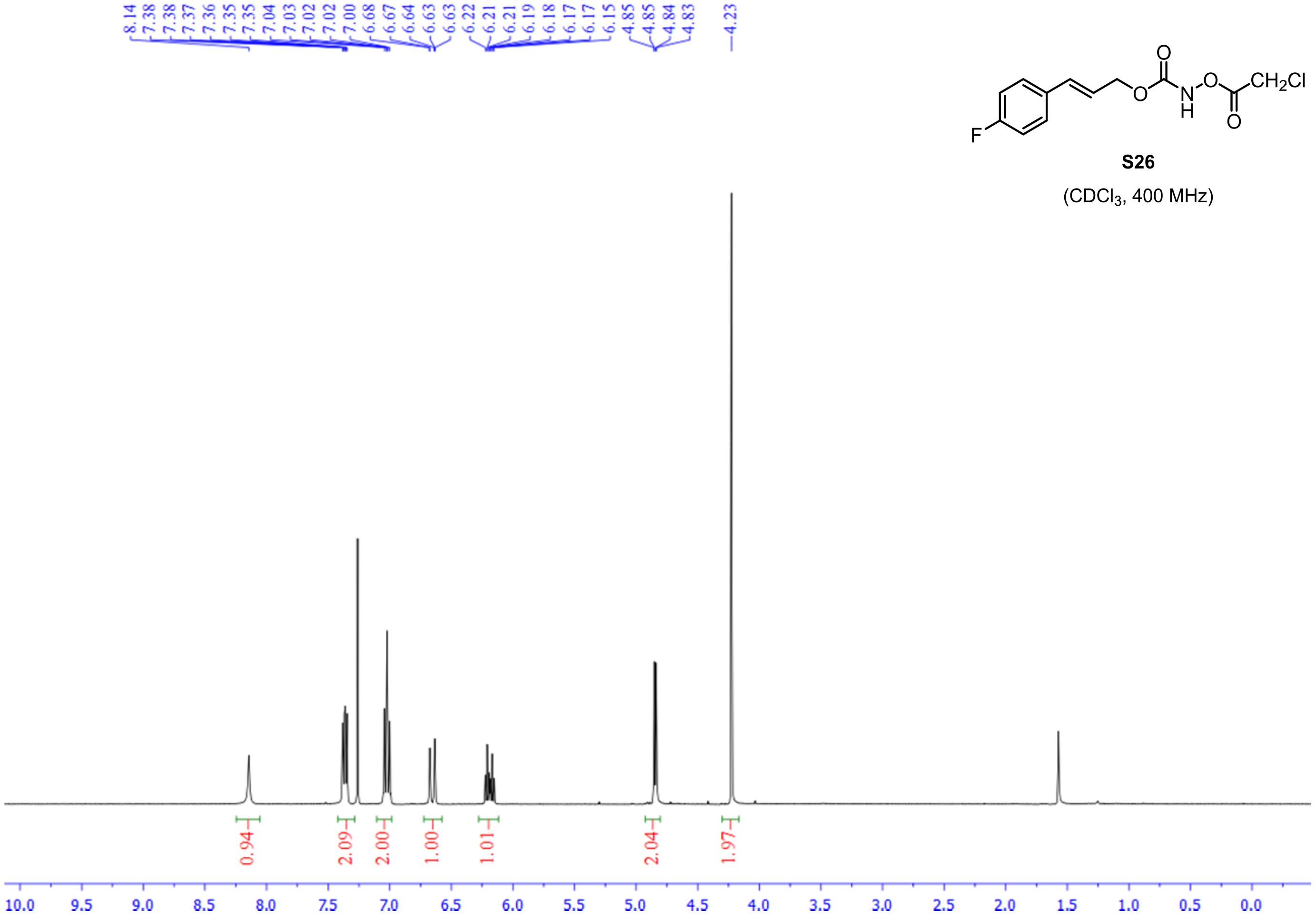

S26

($CDCl_3$, 400 MHz)

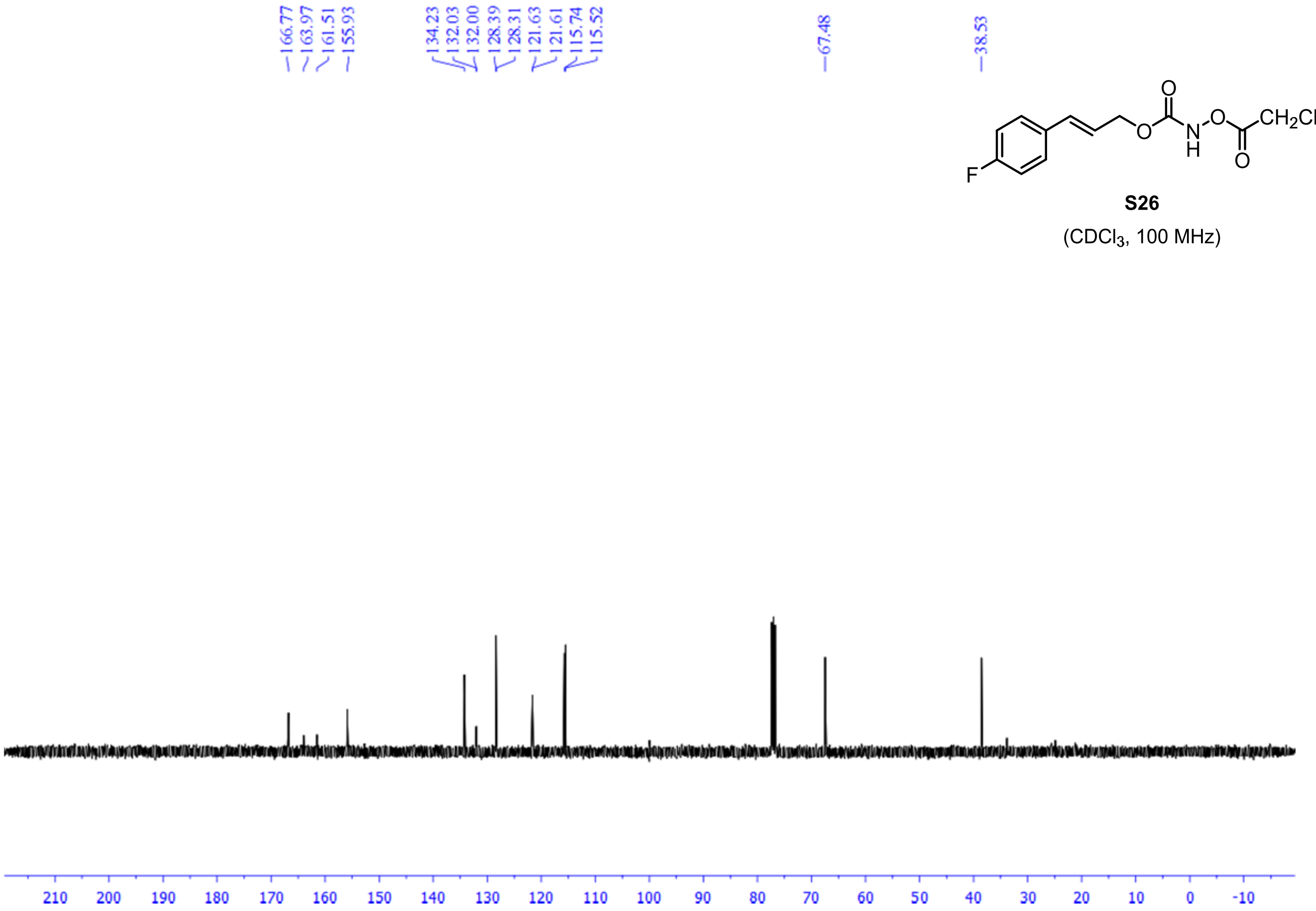

S26

($CDCl_3$, 100 MHz)

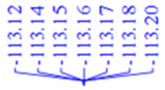

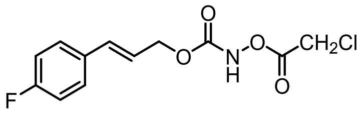

S26

($CDCl_3$, 376MHz)

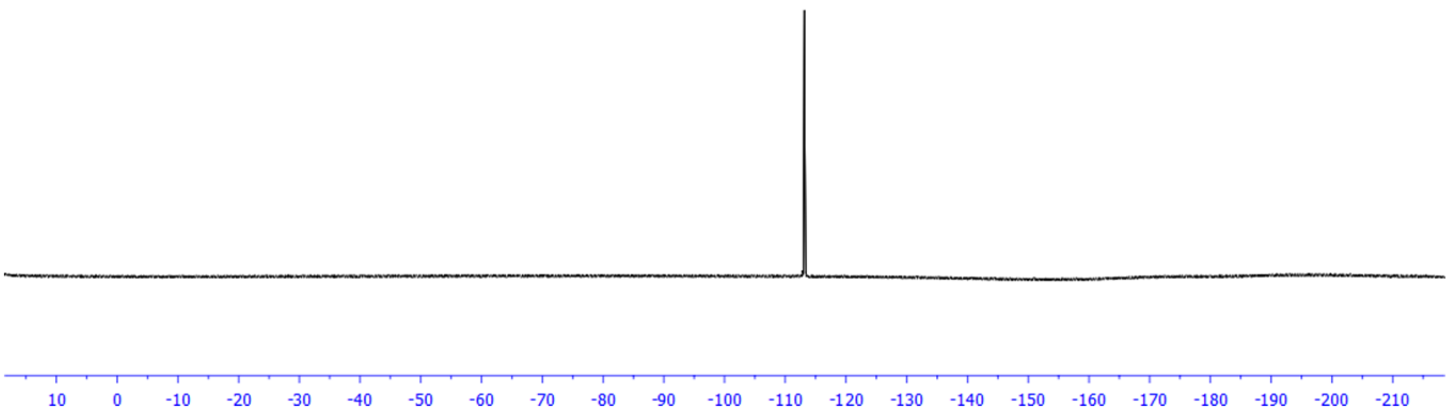

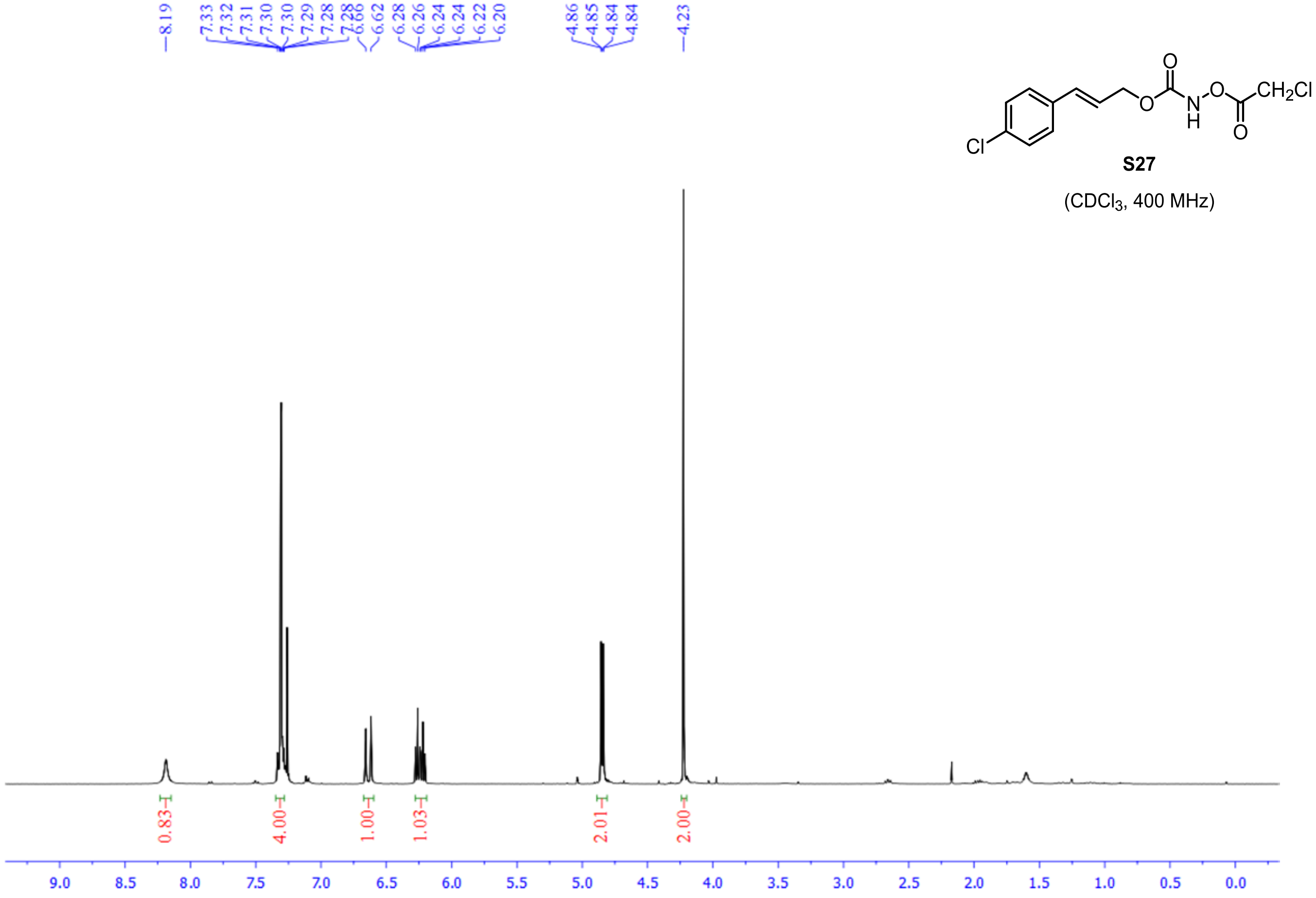

S27

($CDCl_3$, 400 MHz)

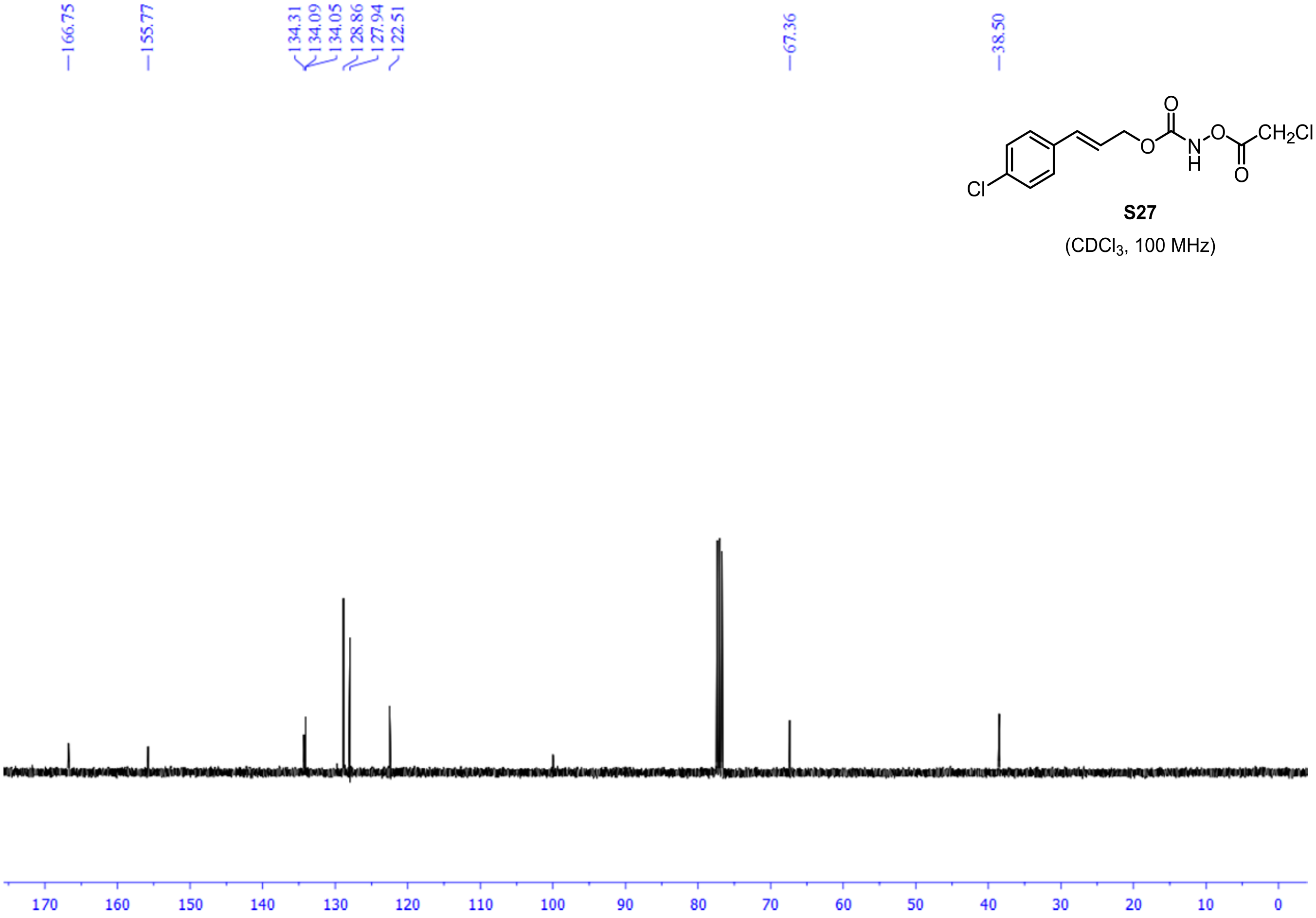

S27

($CDCl_3$, 100 MHz)

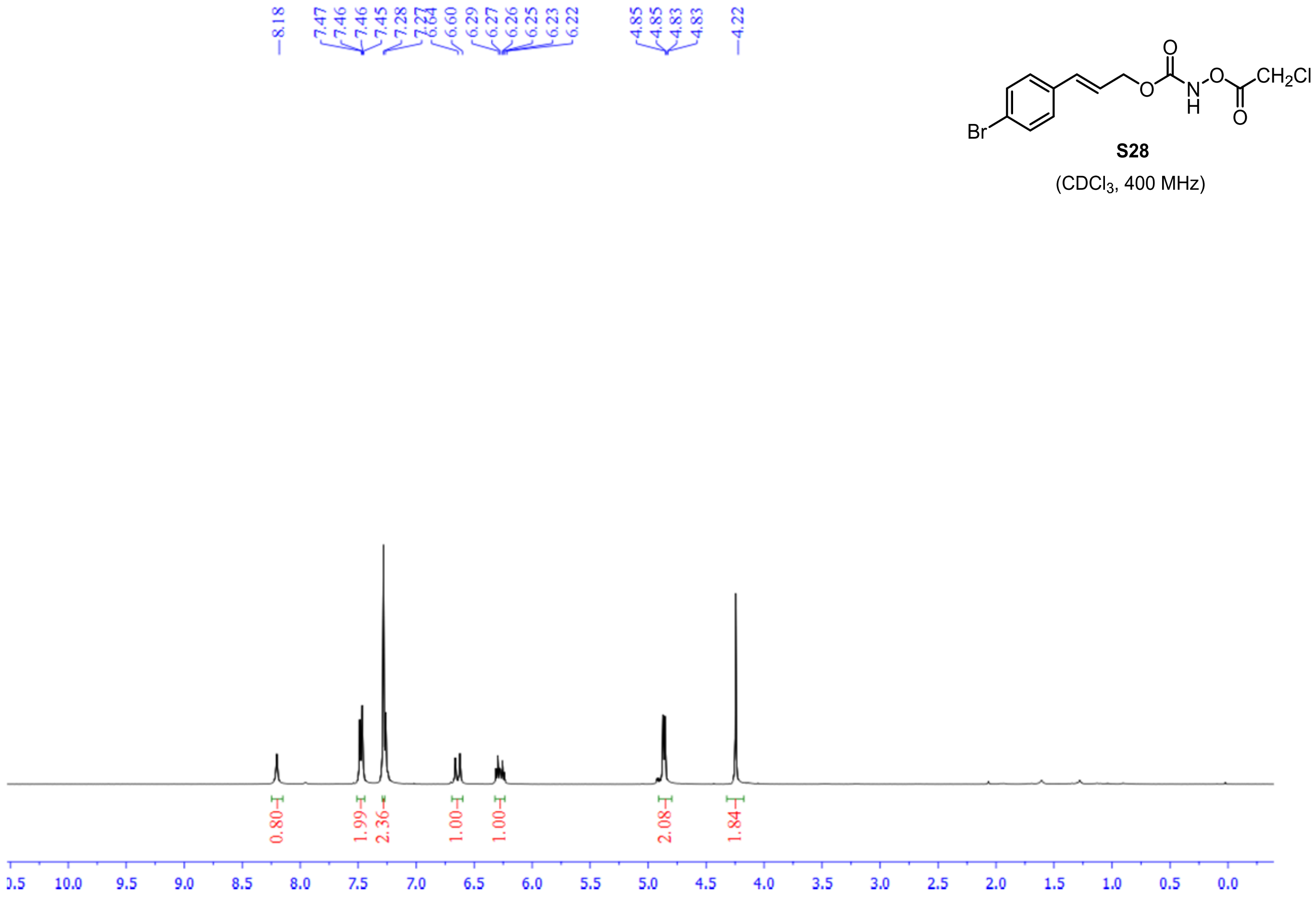

S28

($CDCl_3$, 400 MHz)

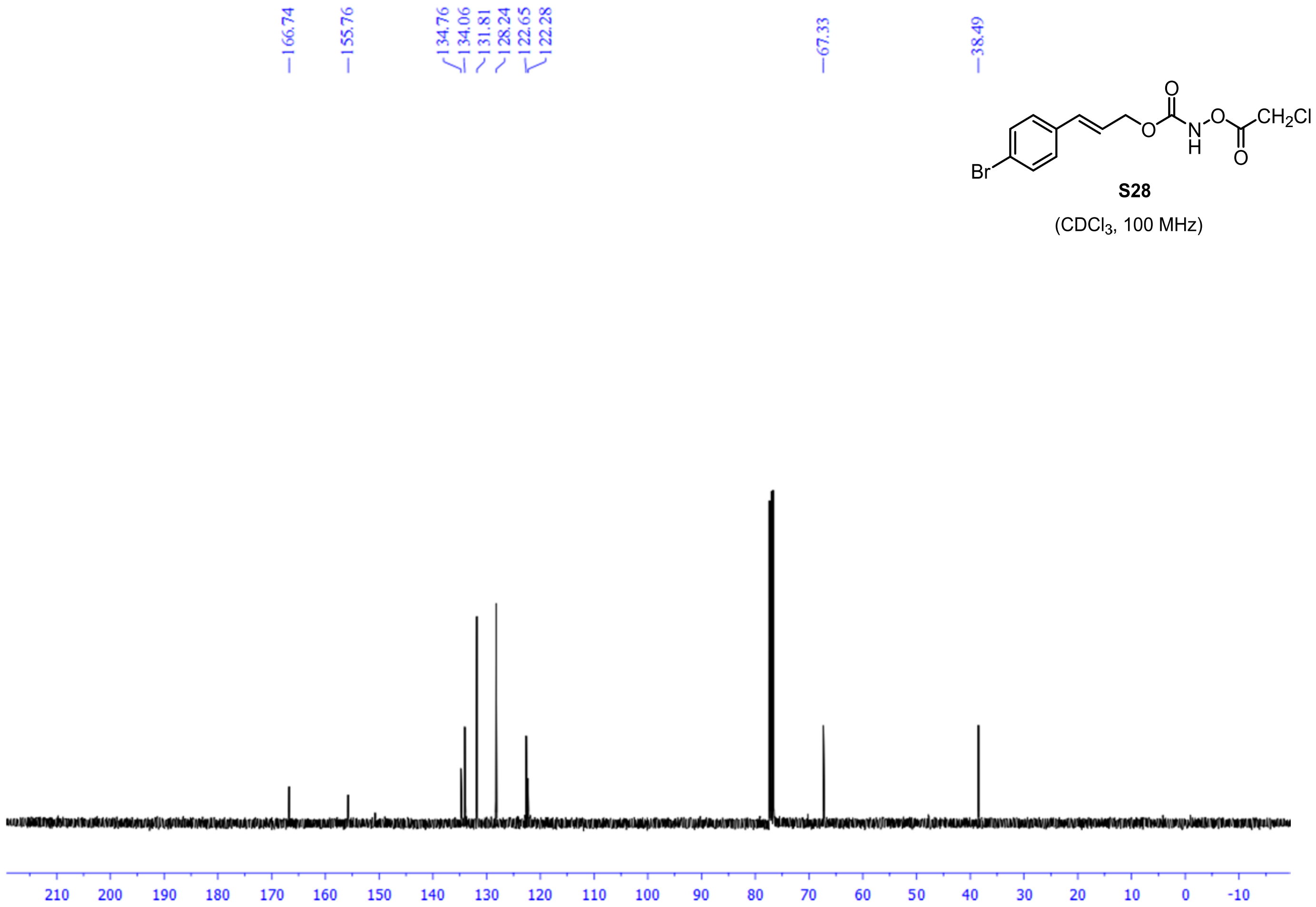

S28

($CDCl_3$, 100 MHz)

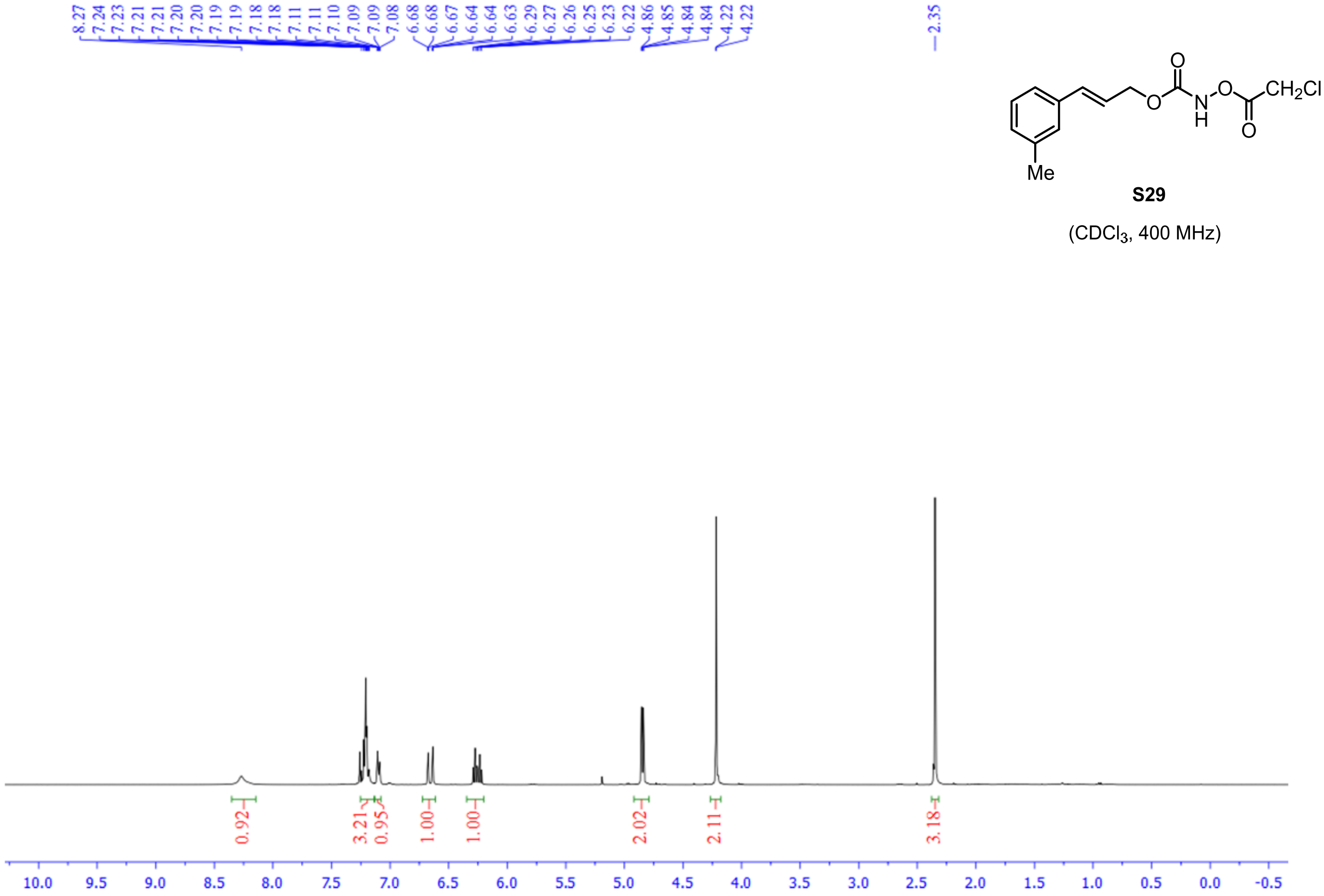

S29

($CDCl_3$, 400 MHz)

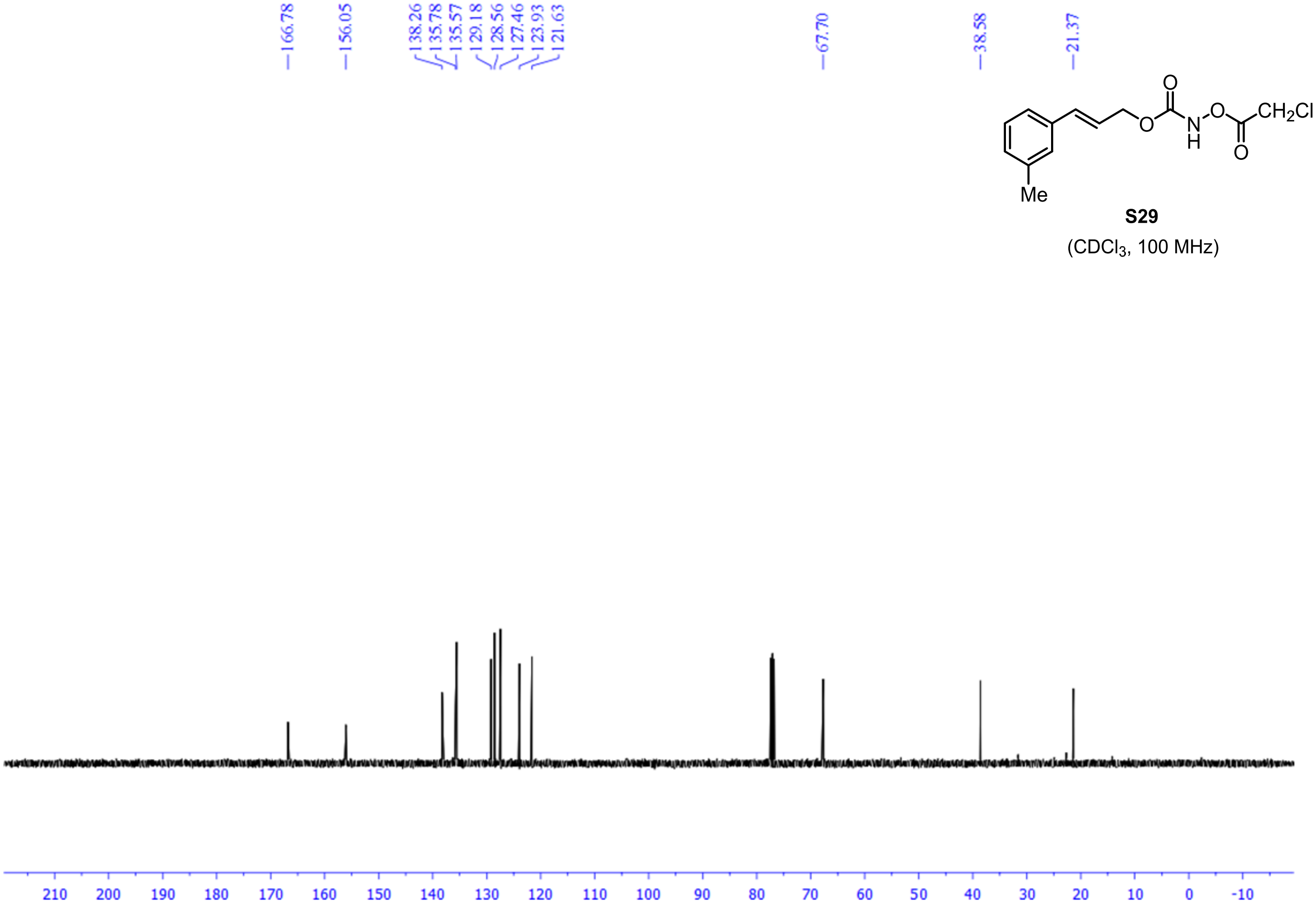

S29

($CDCl_3$, 100 MHz)

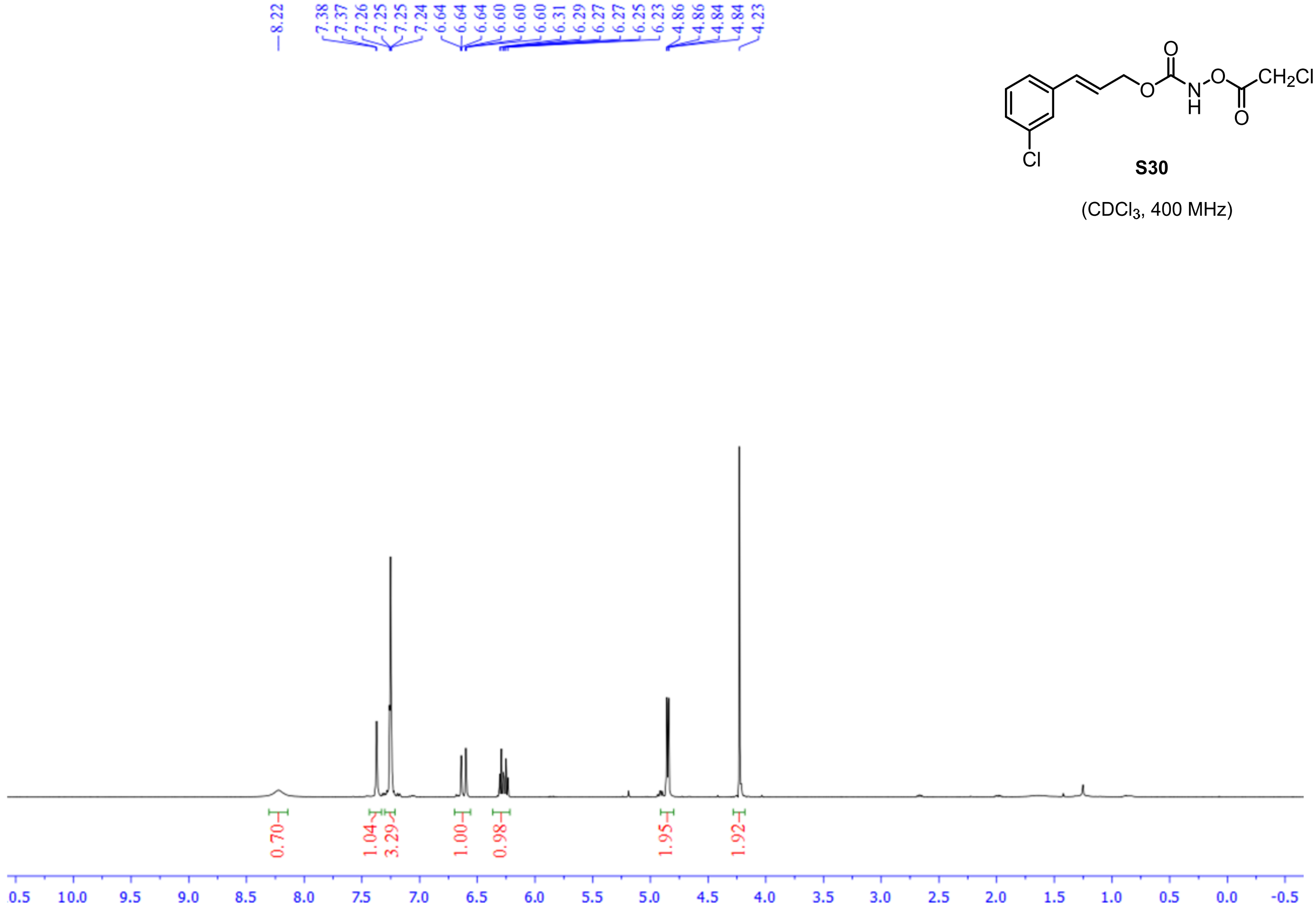
8.22
7.38 7.37 7.26 7.25 7.25 7.24 6.64 6.64 6.64 6.60 6.60 6.60 6.31 6.29 6.27 6.27 6.25 6.23 4.86 4.86 4.84 4.84 4.23
O
O
N
H
O
CH_2Cl
O
Cl
S30
($CDCl_3$, 400 MHz)
0.70
1.04
3.29
1.00
0.98
1.95
1.92
0.5 10.0 9.5 9.0 8.5 8.0 7.5 7.0 6.5 6.0 5.5 5.0 4.5 4.0 3.5 3.0 2.5 2.0 1.5 1.0 0.5 0.0 -0.5

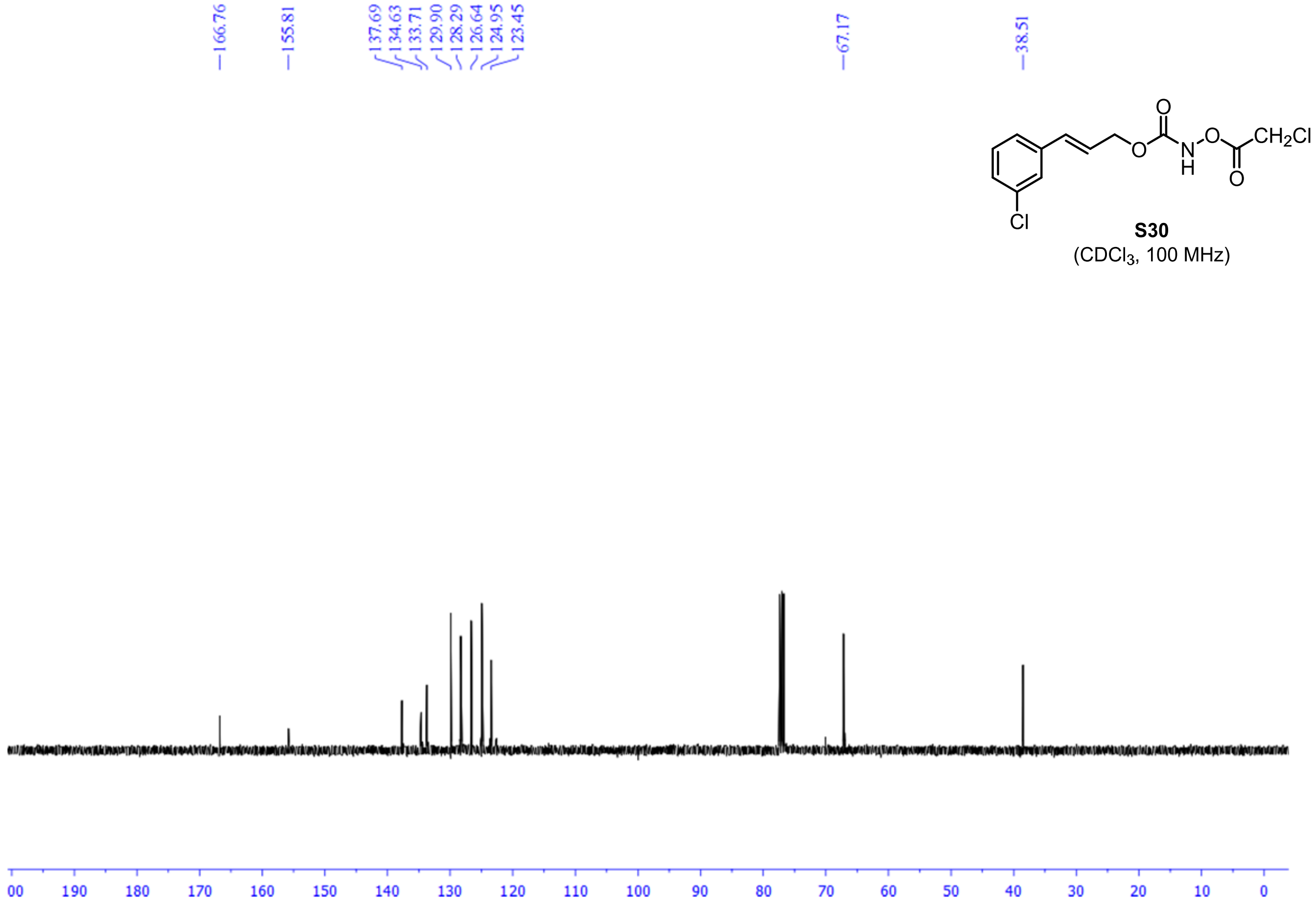
166.76
155.81
137.69 134.63 133.71 129.90 128.29 126.64 124.95 123.45
67.17
38.51
O
O
N
H
O
CH_2Cl
O
Cl
S30
($CDCl_3$, 100 MHz)
00 190 180 170 160 150 140 130 120 110 100 90 80 70 60 50 40 30 20 10 0

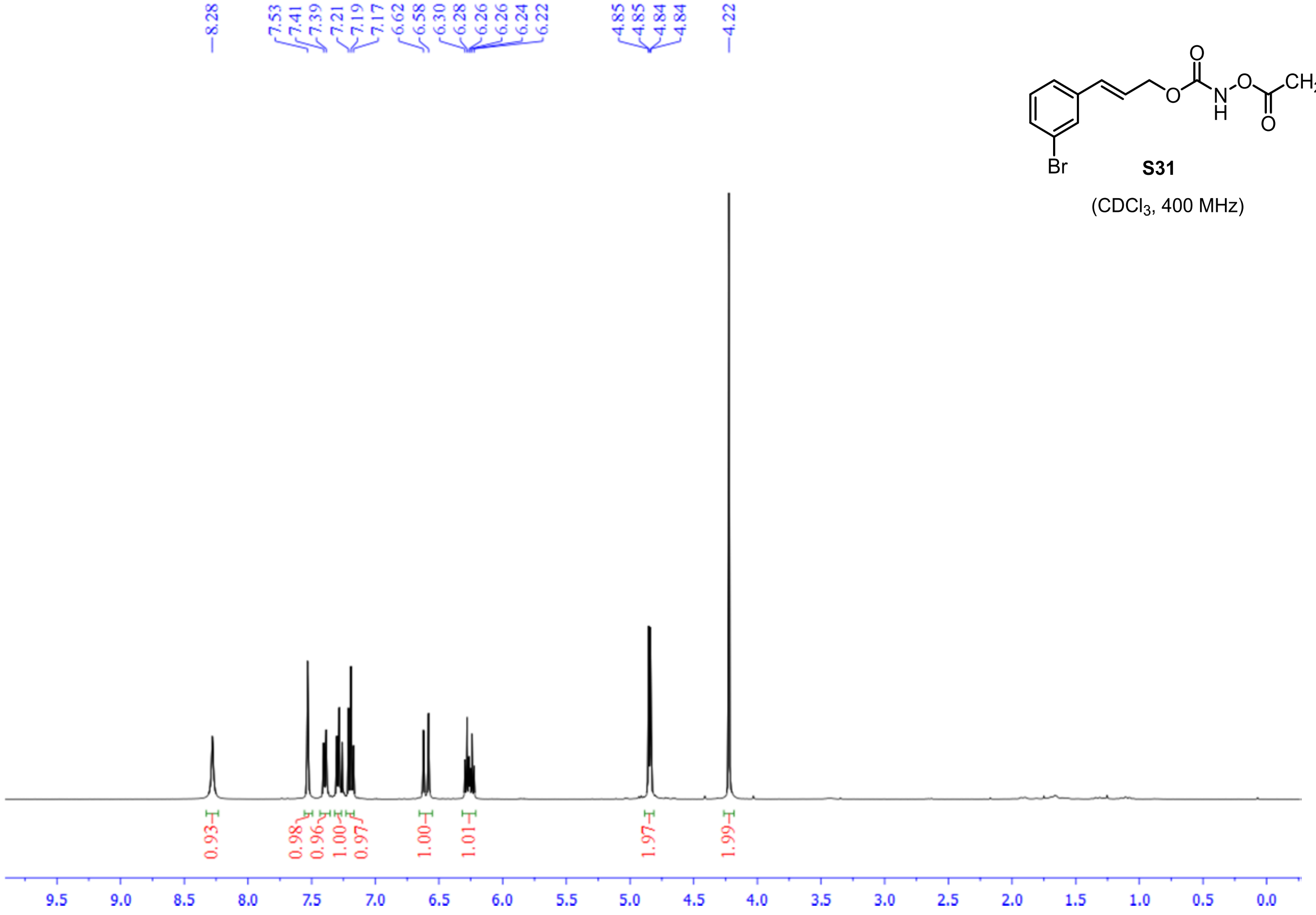
8.28
7.53
7.41
7.39
7.21
7.19
7.17
6.62
6.58
6.30
6.28
6.26
6.26
6.24
6.22
4.85
4.85
4.84
4.84
4.22
S31
(CDCl3, 400 MHz)
0.93
0.98
0.96
1.00
0.97
1.00
1.01
1.97
1.99

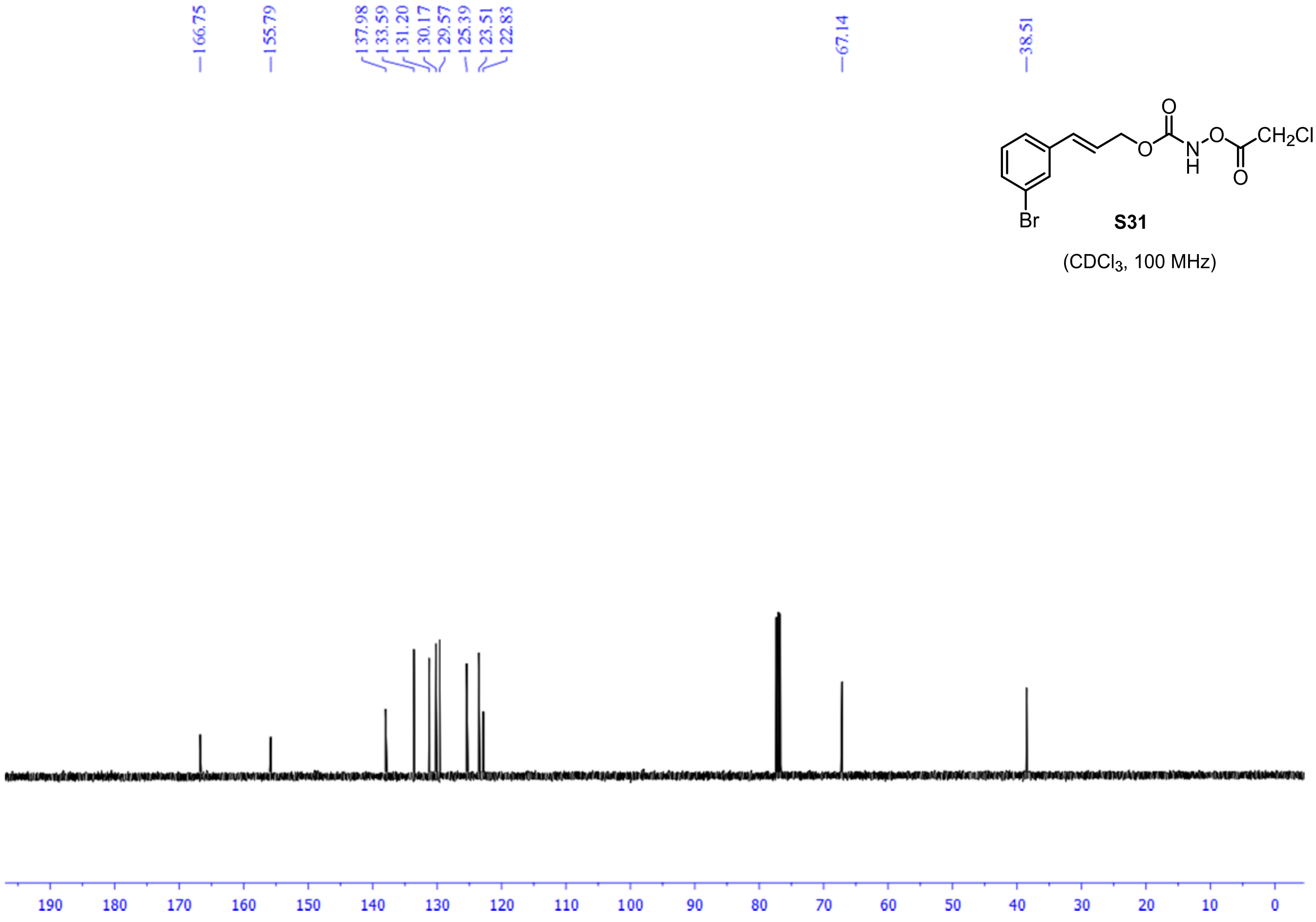
166.75
155.79
137.98
133.59
131.20
130.17
129.57
125.39
123.51
122.83
67.14
38.51
S31
(CDCl3, 100 MHz)

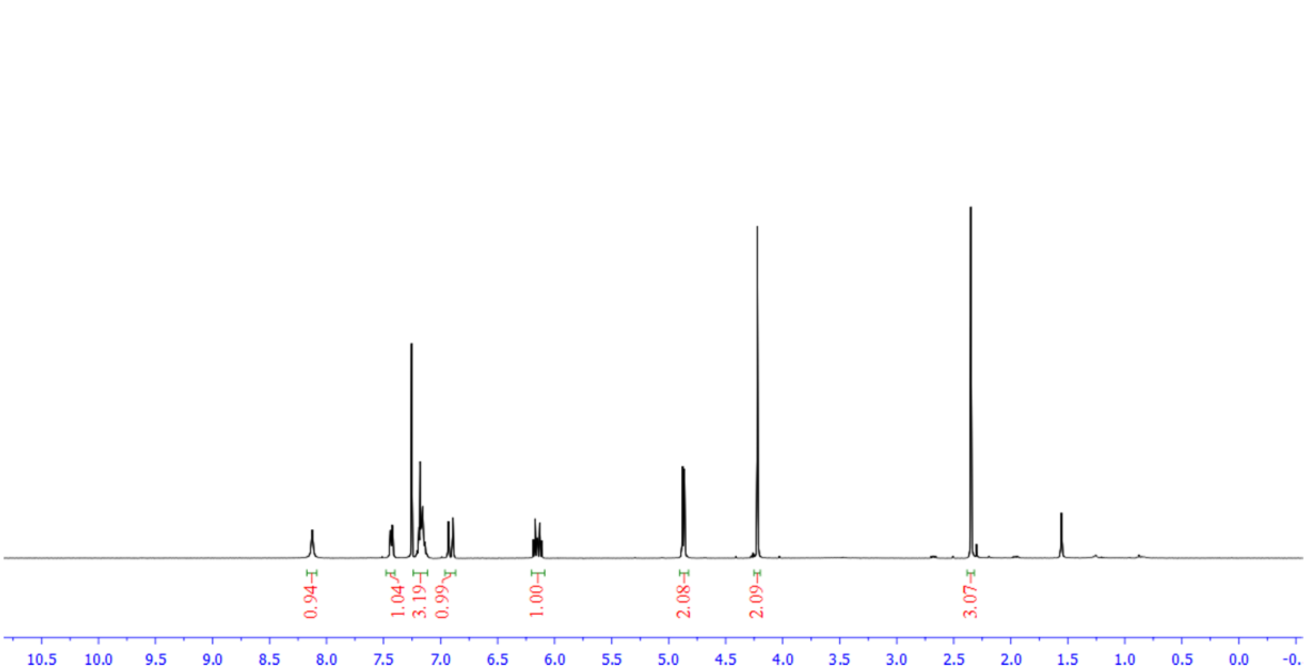

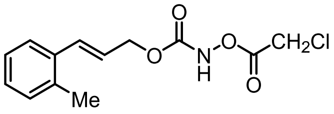

S32
($CDCl_3$, 400 MHz)

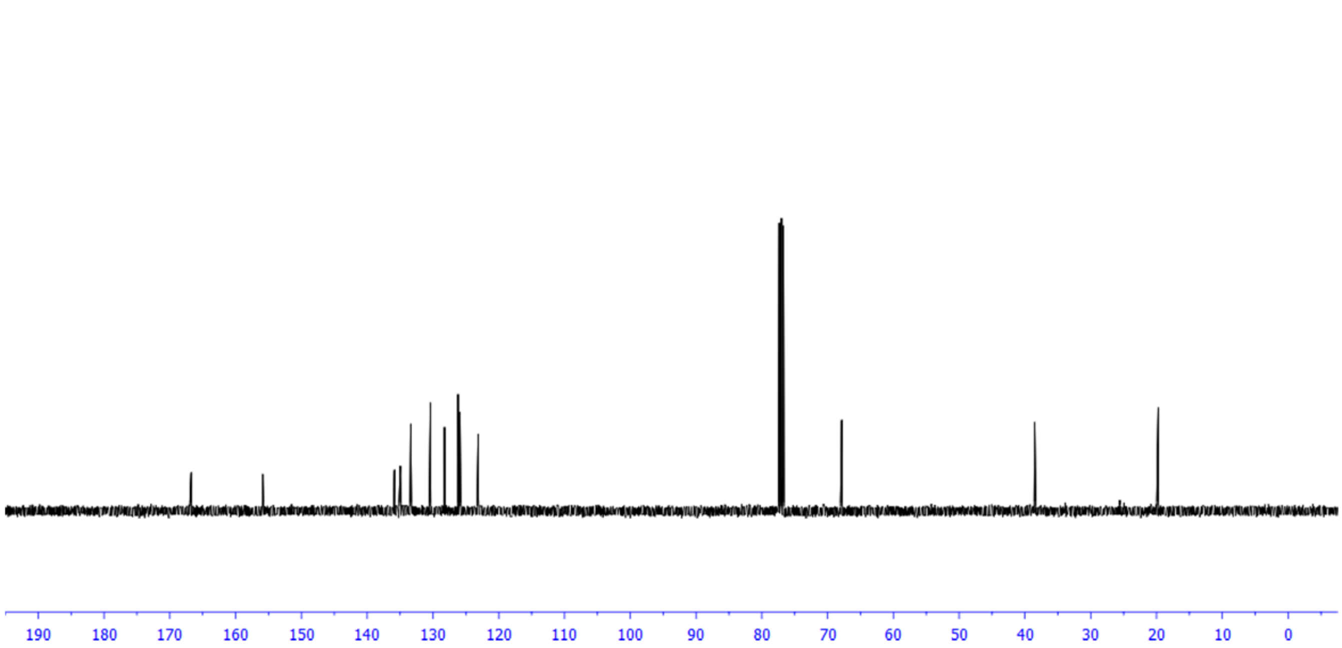

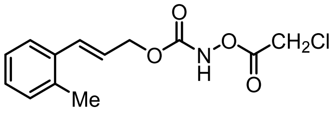

S32
($CDCl_3$, 100 MHz)

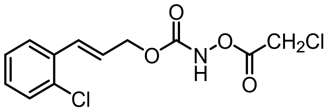

S33

($CDCl_3$, 400 MHz)

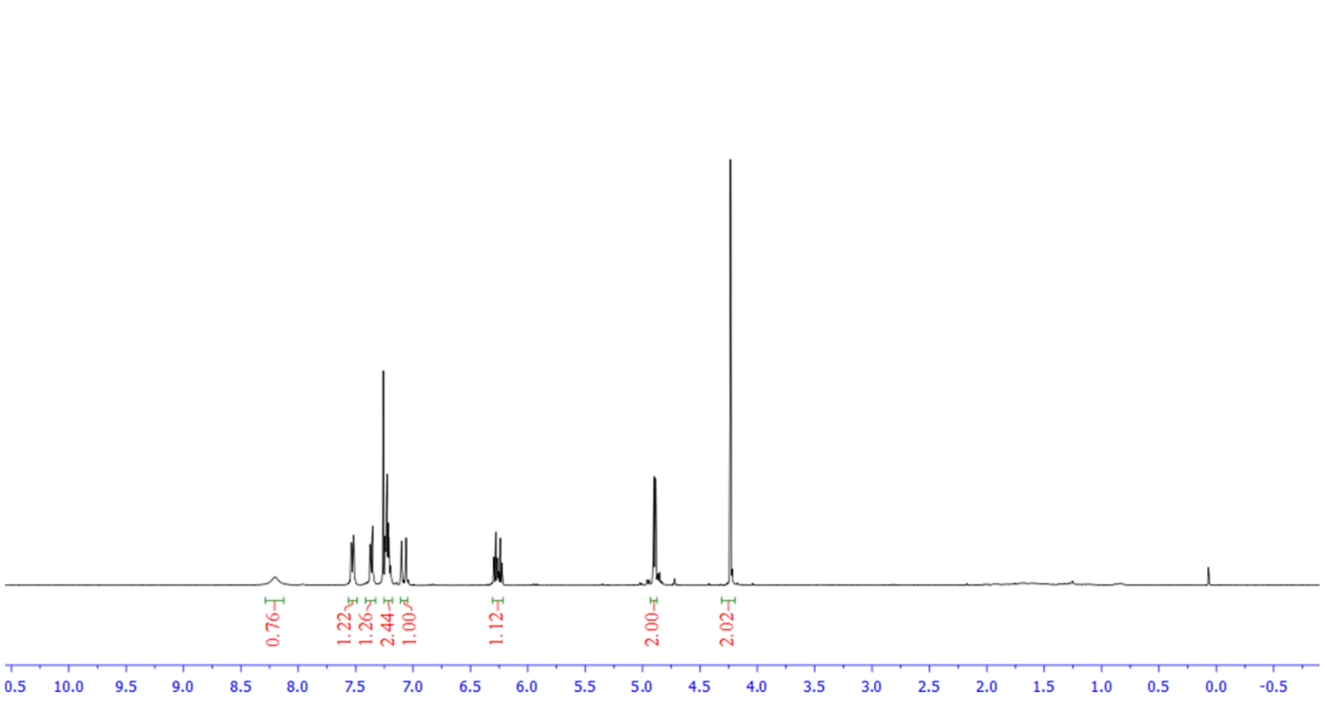

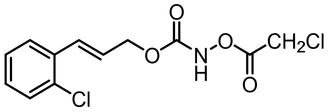

S33

($CDCl_3$, 100 MHz)

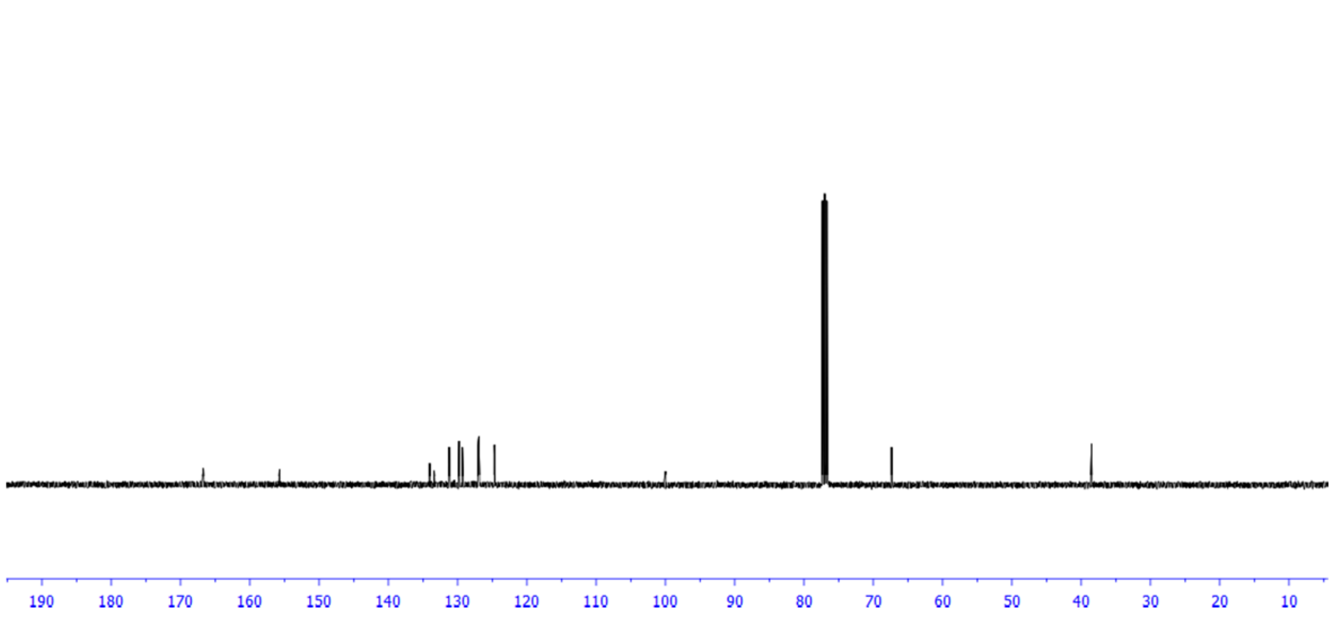

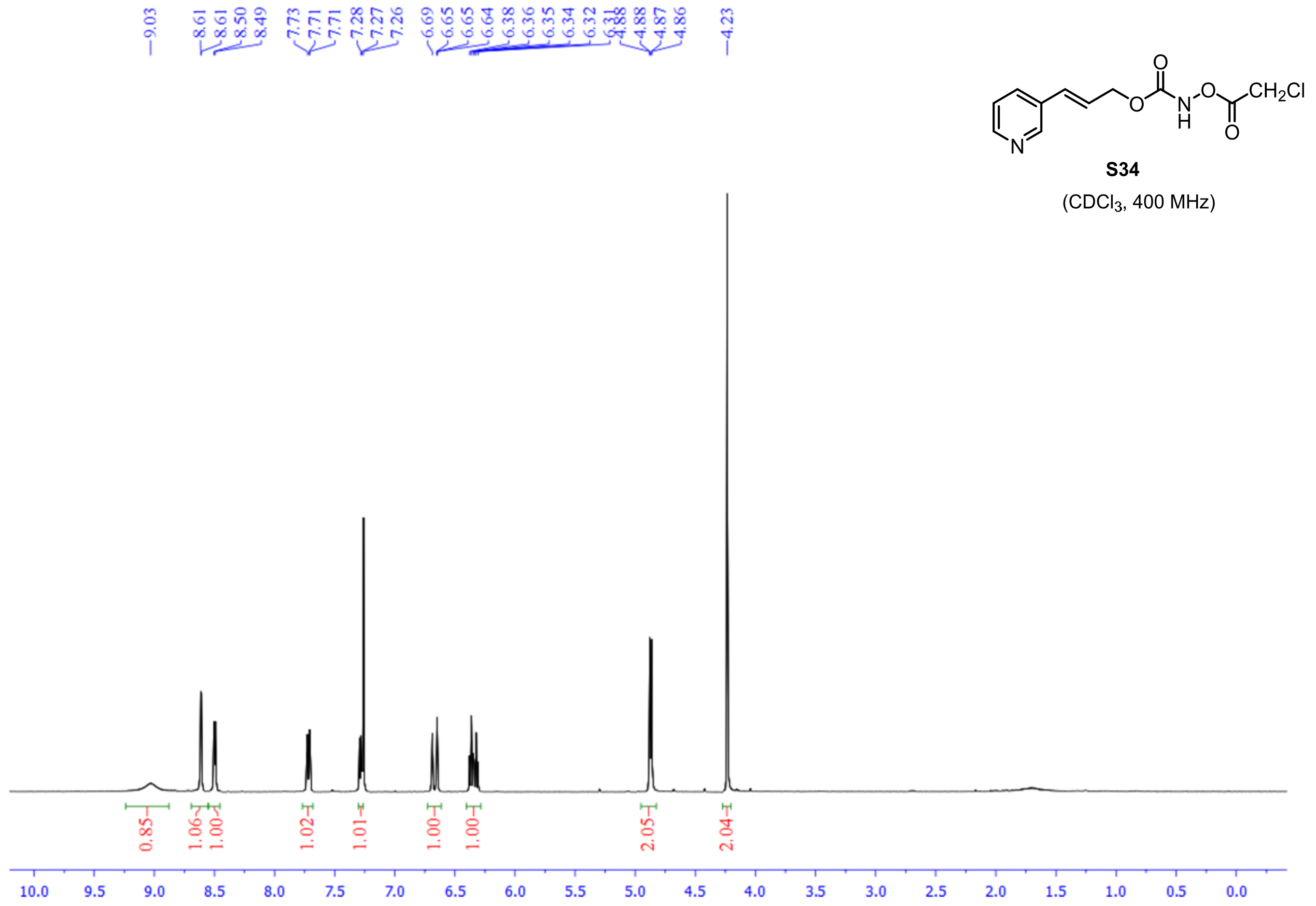

S34

($CDCl_3$, 400 MHz)

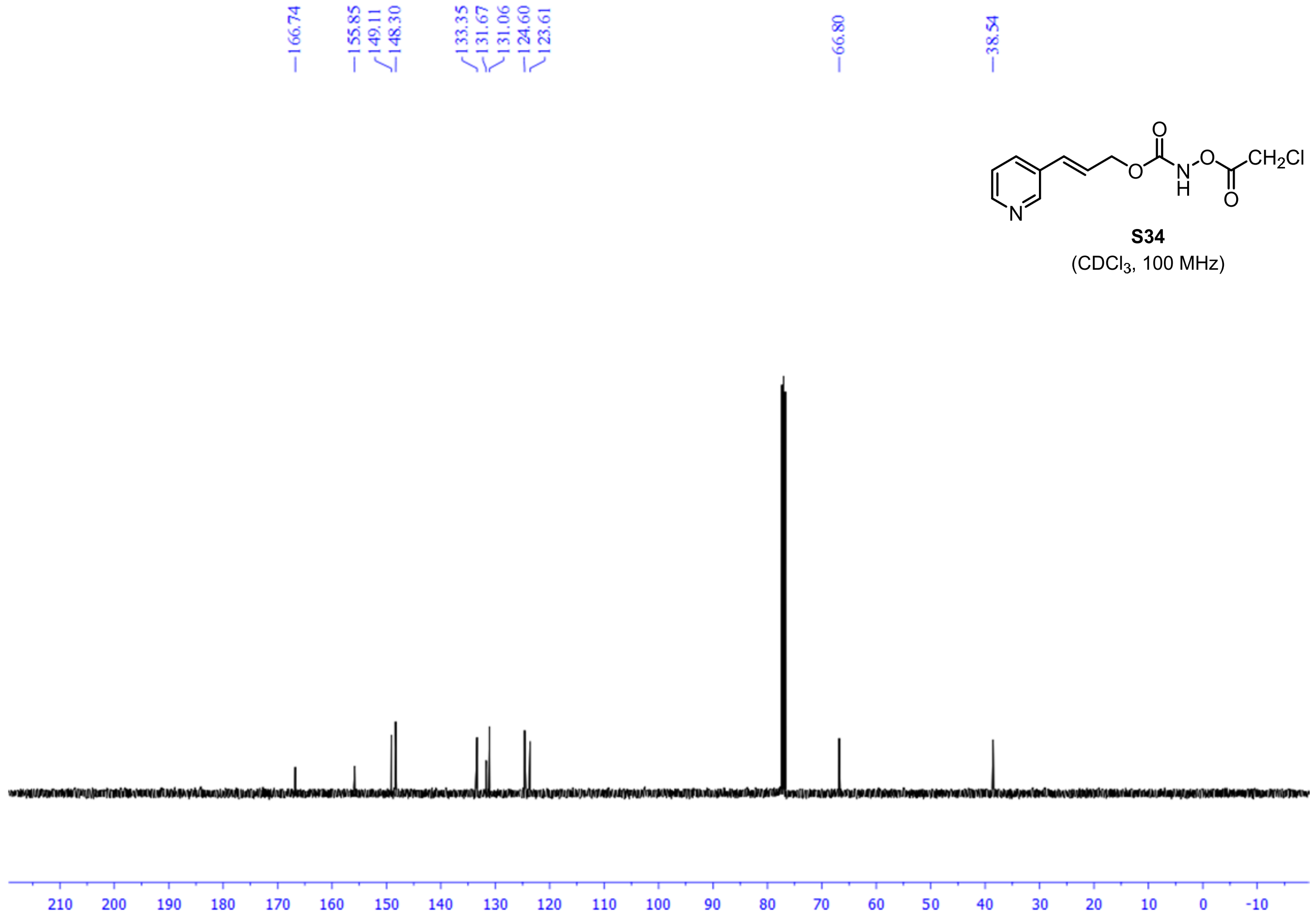

S34

($CDCl_3$, 100 MHz)

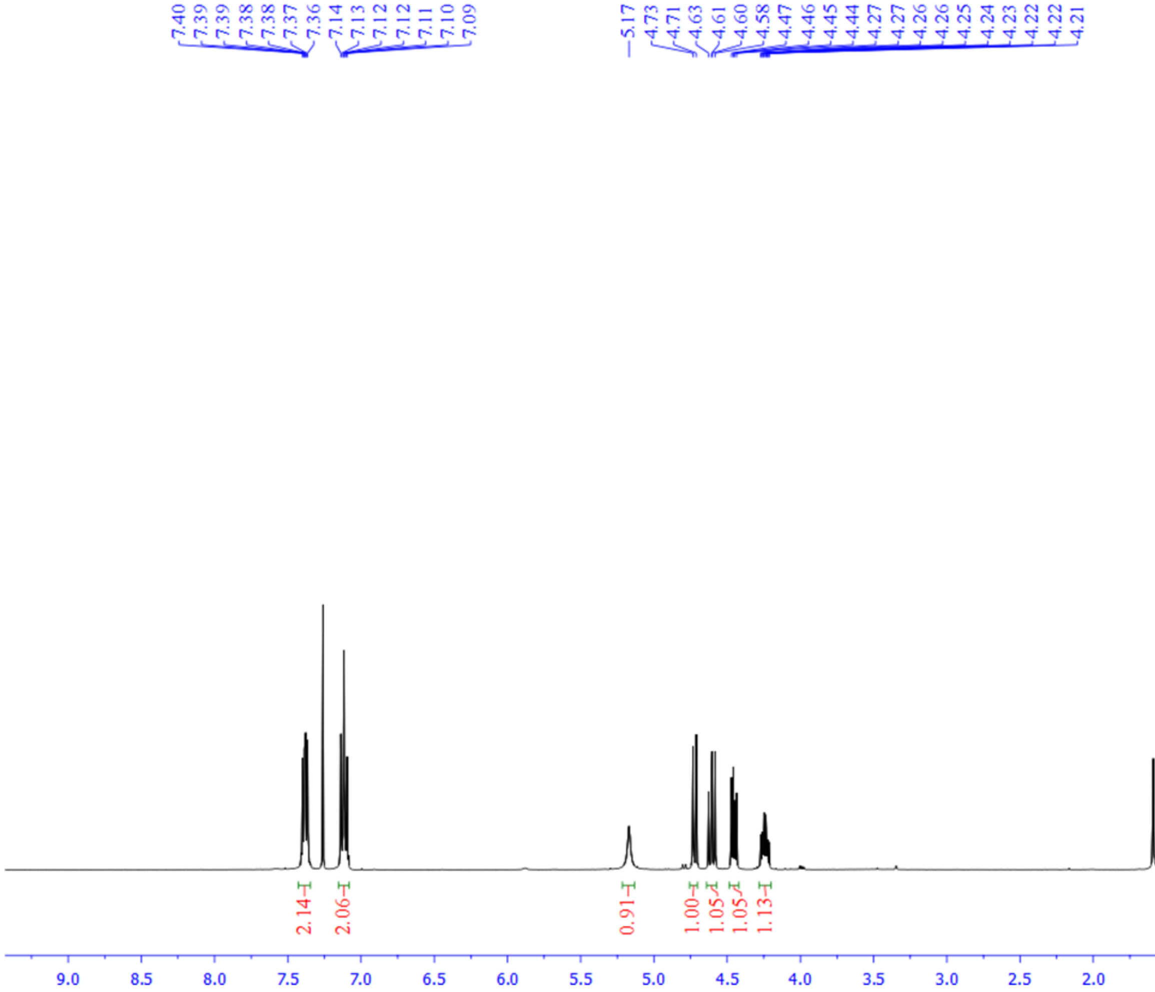

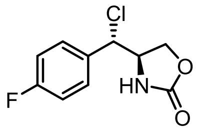

S35

($CDCl_3$, 400 MHz)

dr :17:1

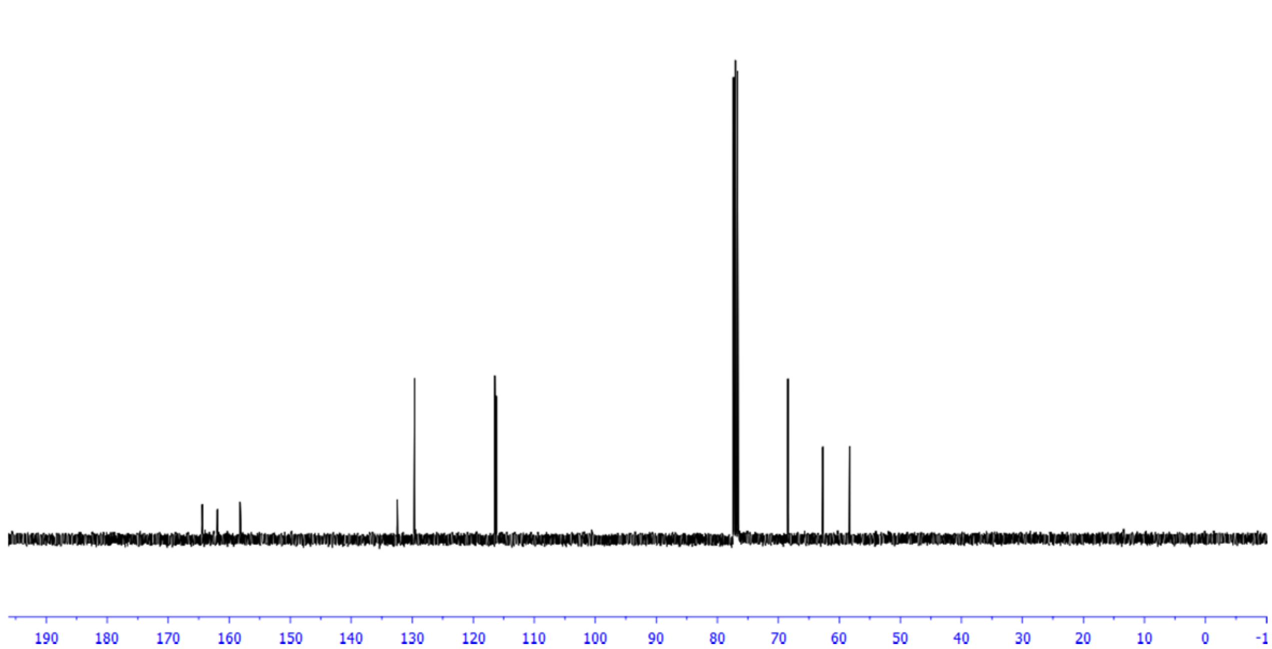

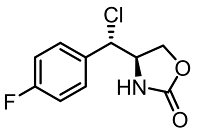

S35

($CDCl_3$, 100 MHz)

dr :17:1

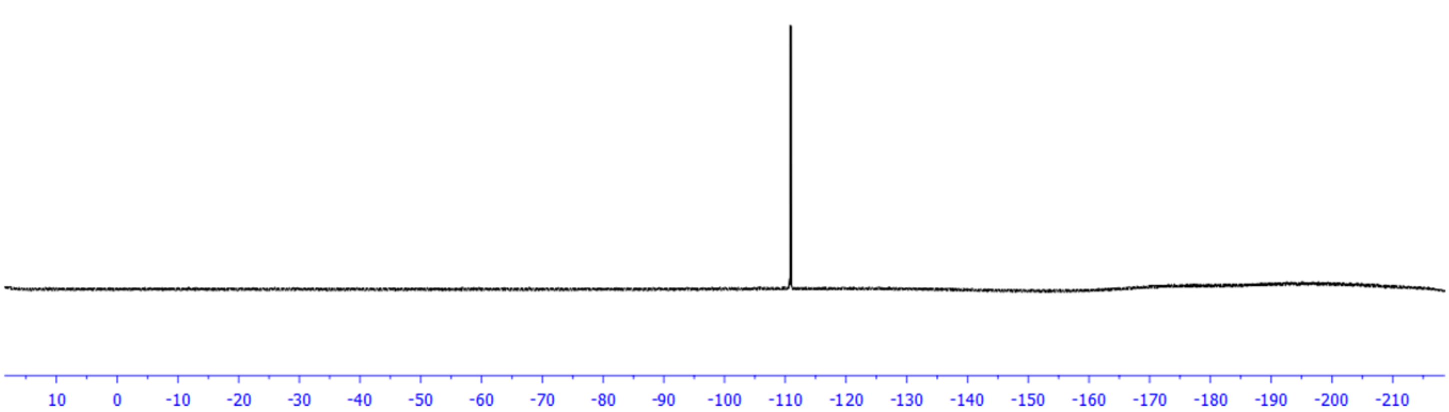

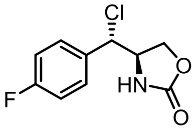

S35

($CDCl_3$, 376MHz)
dr :17:1

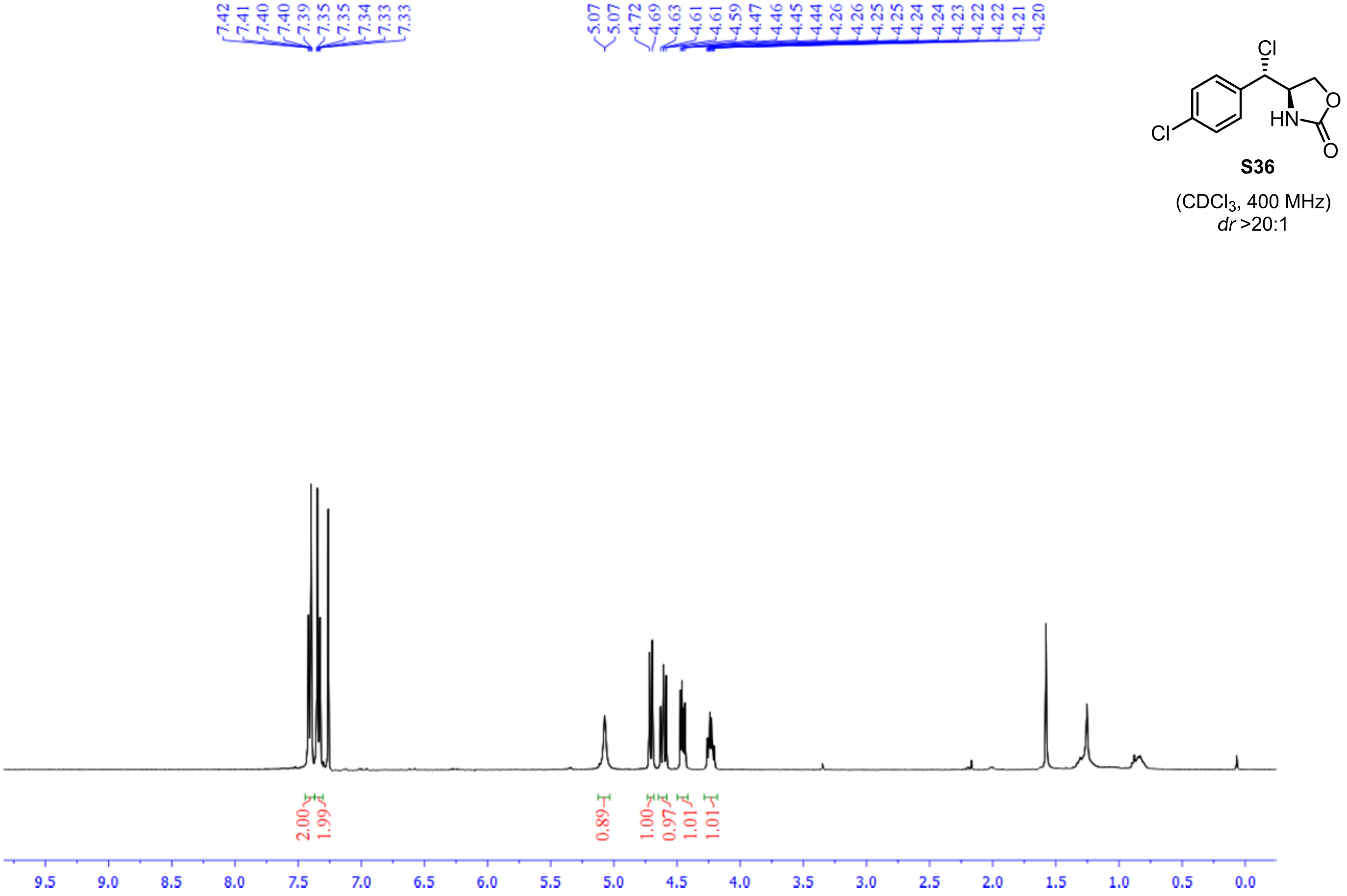

S36

($CDCl_3$, 400 MHz)
dr >20:1

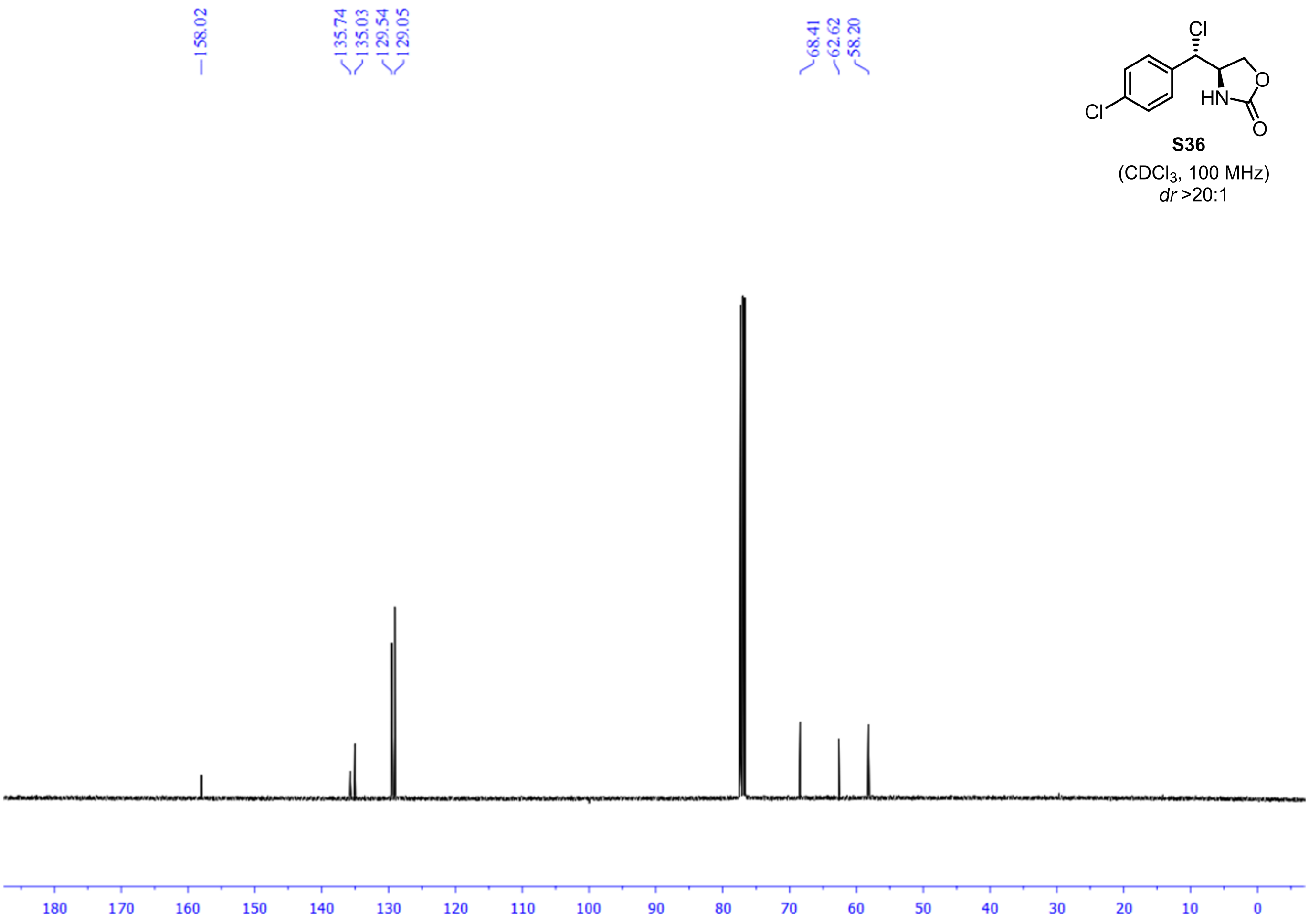

S36

($CDCl_3$, 100 MHz)
dr >20:1

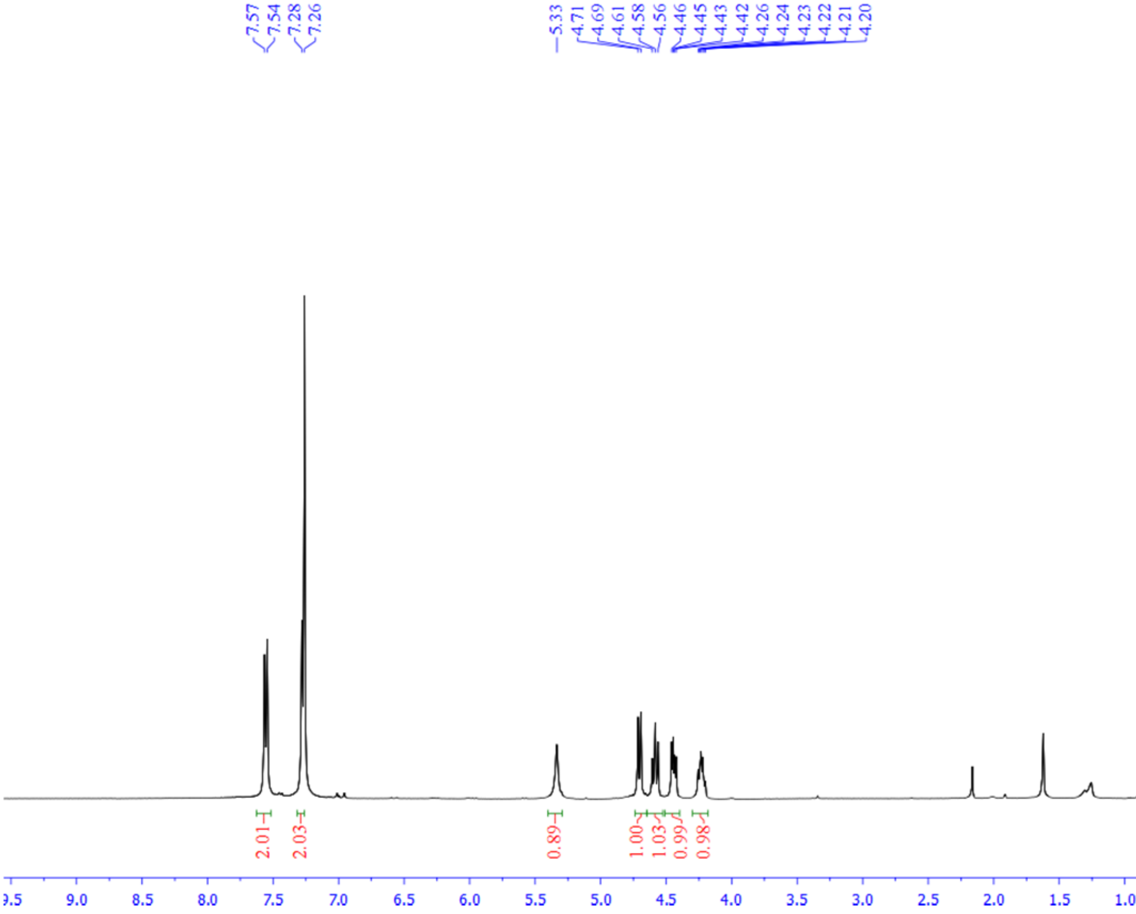

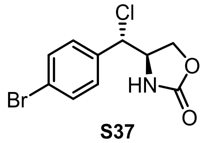

S37

($CDCl_3$, 400 MHz)
dr >20:1

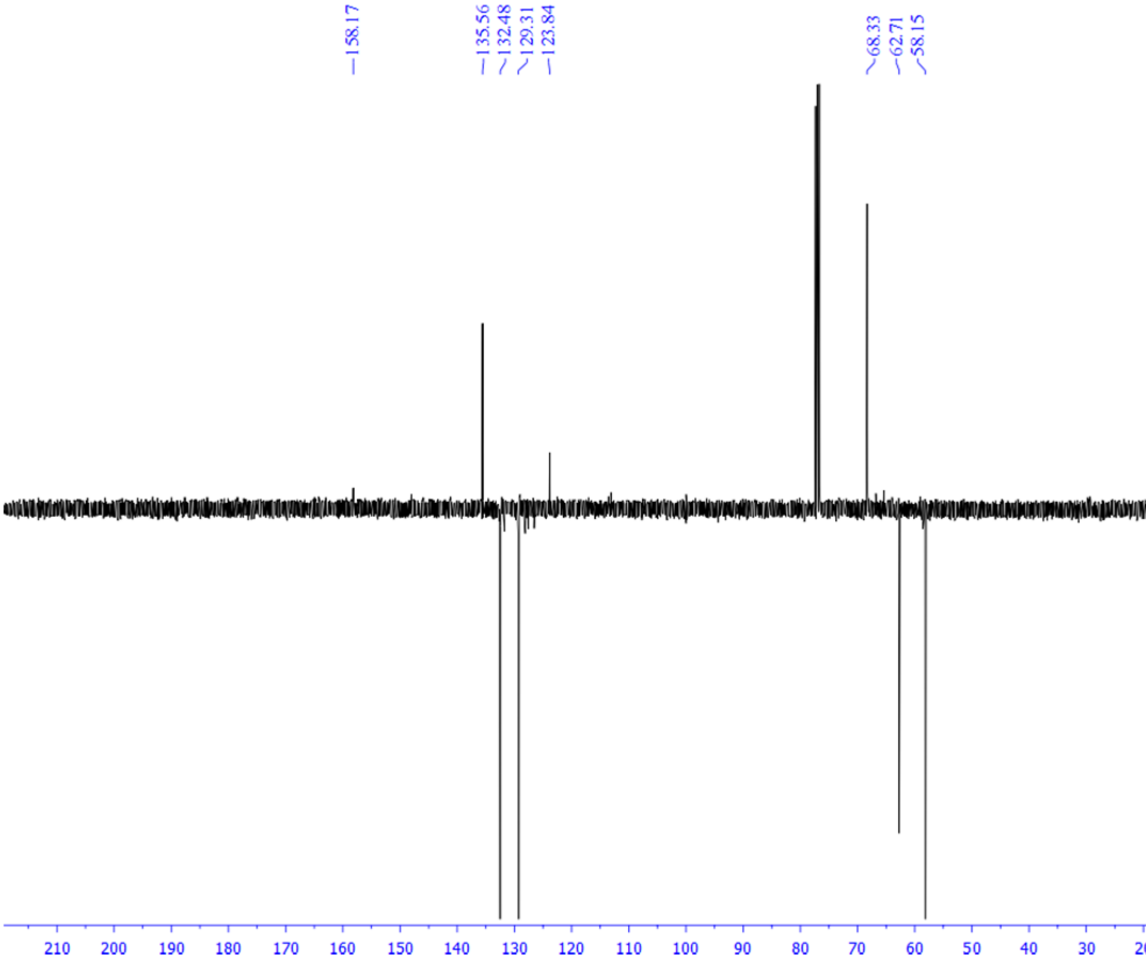

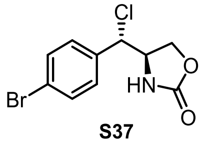

S37

($CDCl_3$, 100 MHz)
dr >20:1

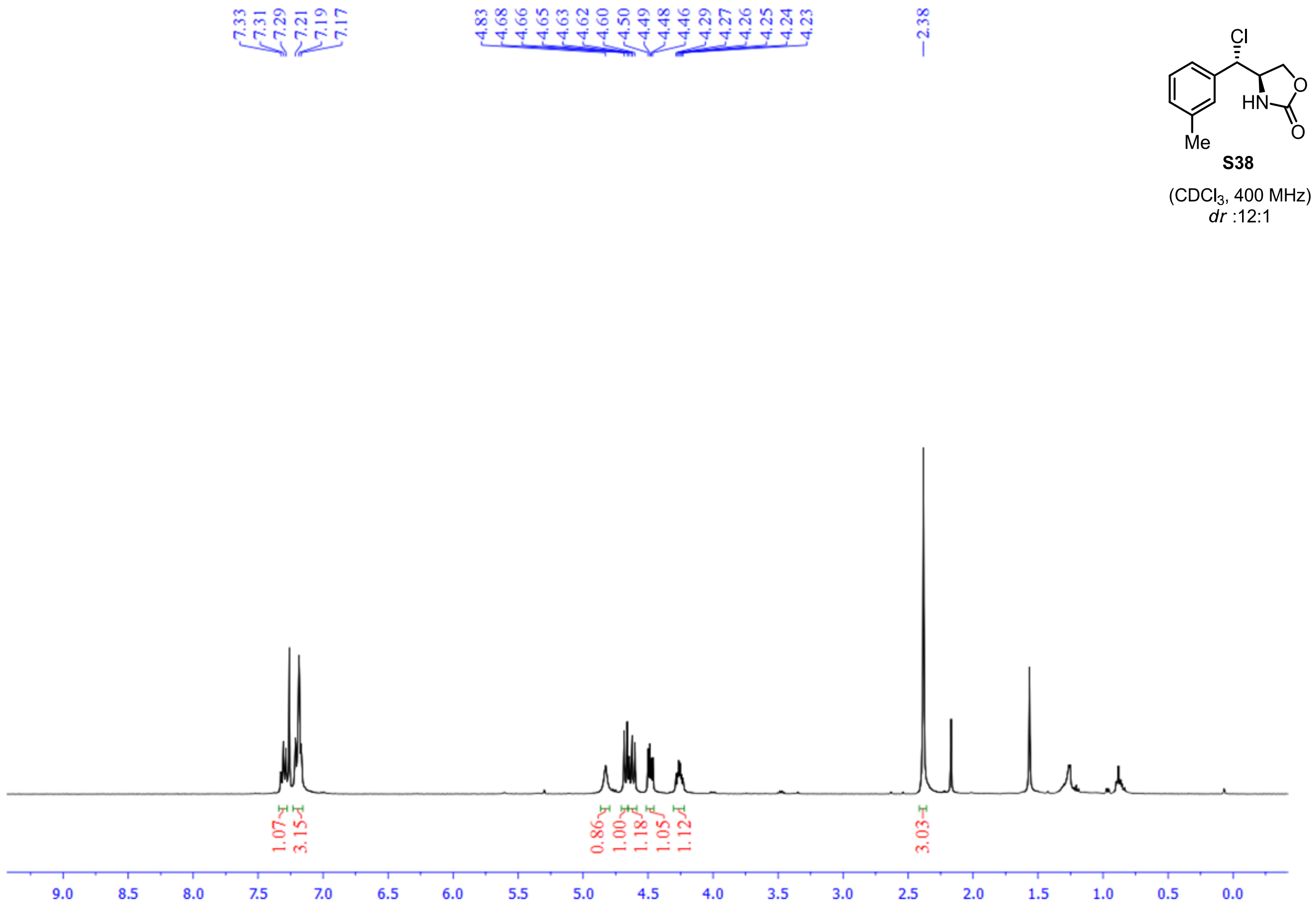
7.33
7.31
7.29
7.21
7.19
7.17
4.83
4.68
4.66
4.65
4.63
4.62
4.60
4.50
4.49
4.48
4.46
4.29
4.27
4.26
4.25
4.24
4.23
2.38
Cl
O
HN
O
Me
S38
(CDCl3, 400 MHz)
dr :12:1
1.07
3.15
0.86
1.00
1.18
1.05
1.12
3.03
9.0 8.5 8.0 7.5 7.0 6.5 6.0 5.5 5.0 4.5 4.0 3.5 3.0 2.5 2.0 1.5 1.0 0.5 0.0

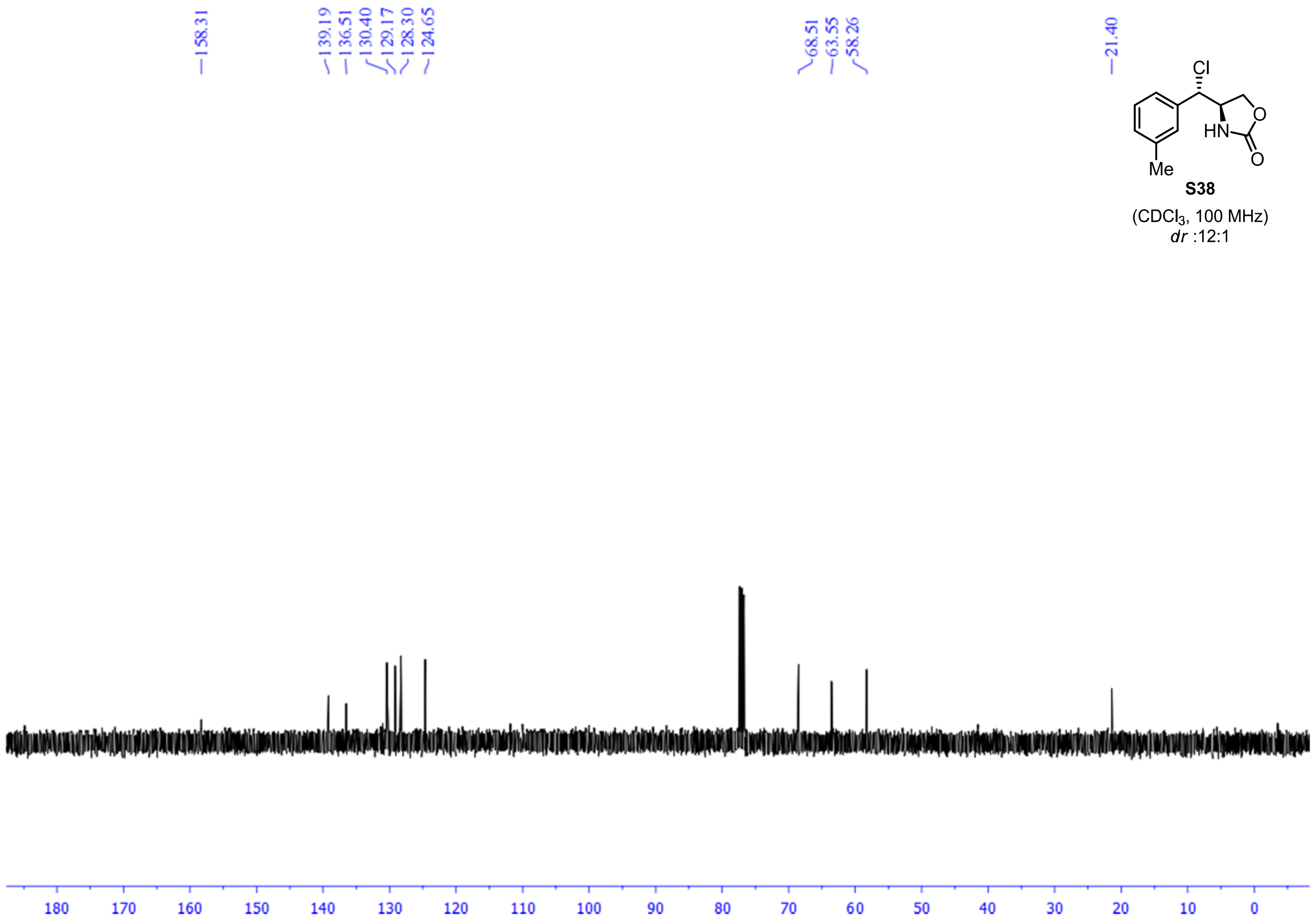
158.31
139.19
136.51
130.40
129.17
128.30
124.65
68.51
63.55
58.26
21.40
Cl
O
HN
O
Me
S38
(CDCl3, 100 MHz)
dr :12:1
180 170 160 150 140 130 120 110 100 90 80 70 60 50 40 30 20 10 0

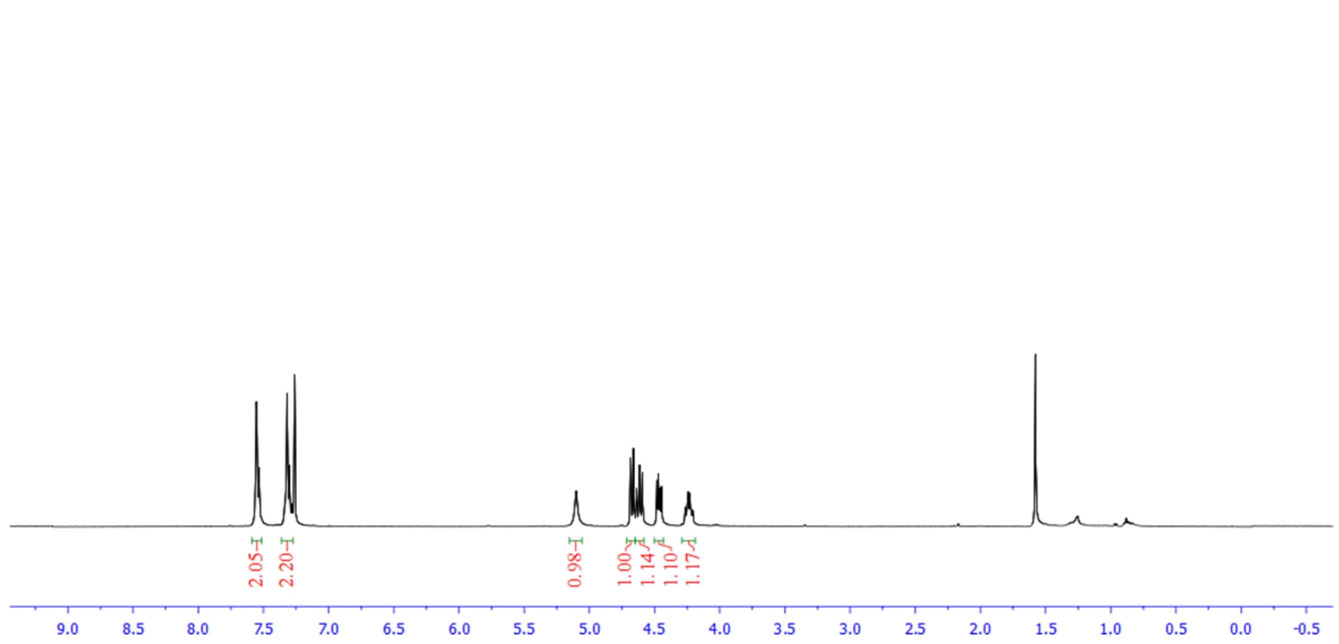

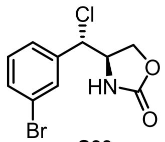

S39

($CDCl_3$, 400 MHz)
dr >20:1

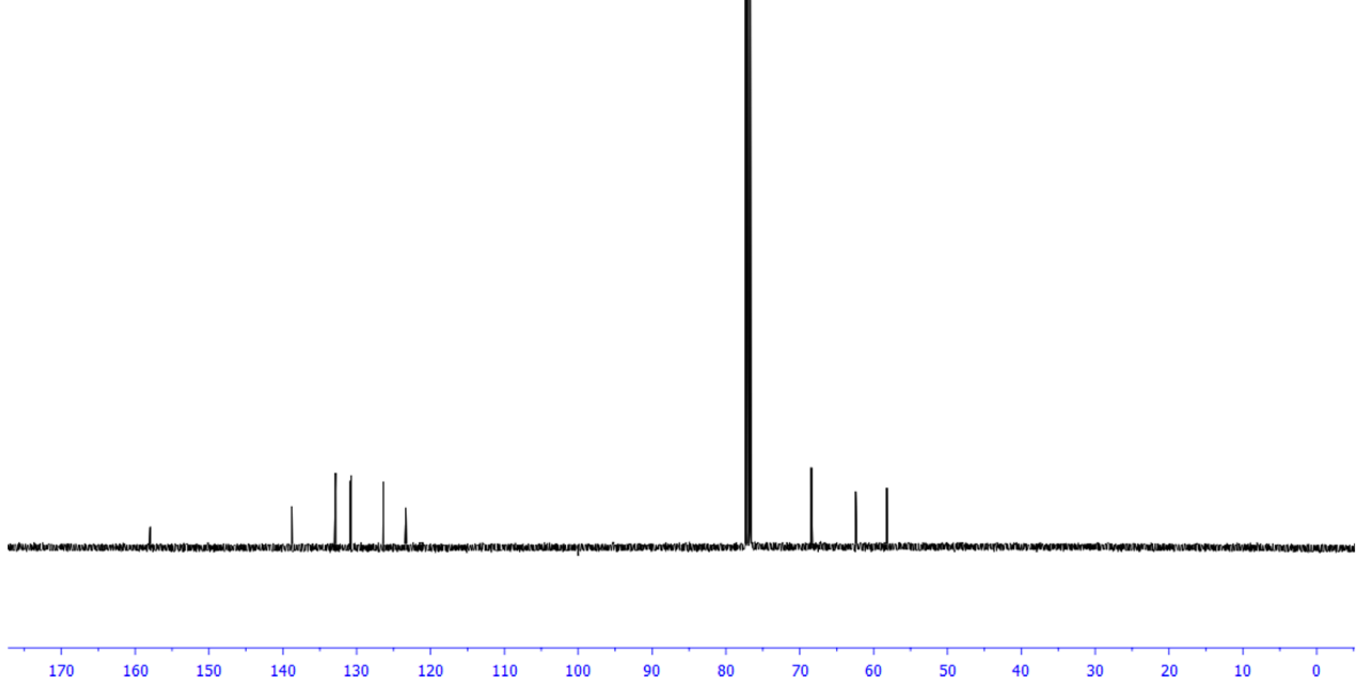

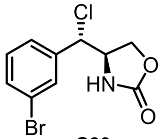

S39

($CDCl_3$, 100 MHz)
dr >20:1

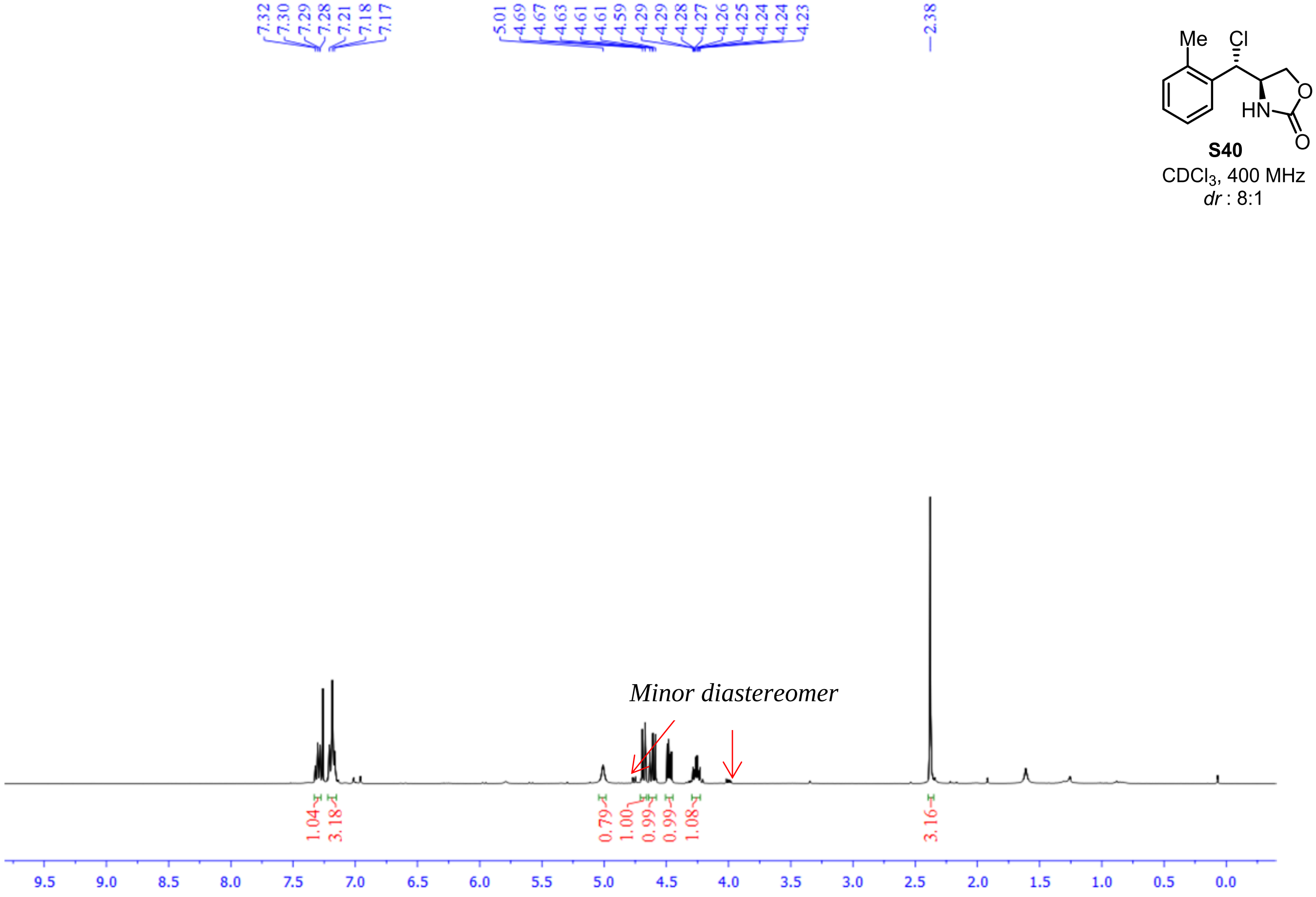

7.32
7.30
7.29
7.28
7.21
7.18
7.17
5.01
4.69
4.67
4.63
4.61
4.61
4.59
4.29
4.29
4.28
4.27
4.26
4.25
4.24
4.24
4.23
2.38
Me
Cl
O
HN
O
S40
CDCl3, 400 MHz
dr : 8:1
Minor diastereomer
1.04
3.18
0.79
1.00
0.99
0.99
1.08
3.16
9.5
9.0
8.5
8.0
7.5
7.0
6.5
6.0
5.5
5.0
4.5
4.0
3.5
3.0
2.5
2.0
1.5
1.0
0.5
0.0

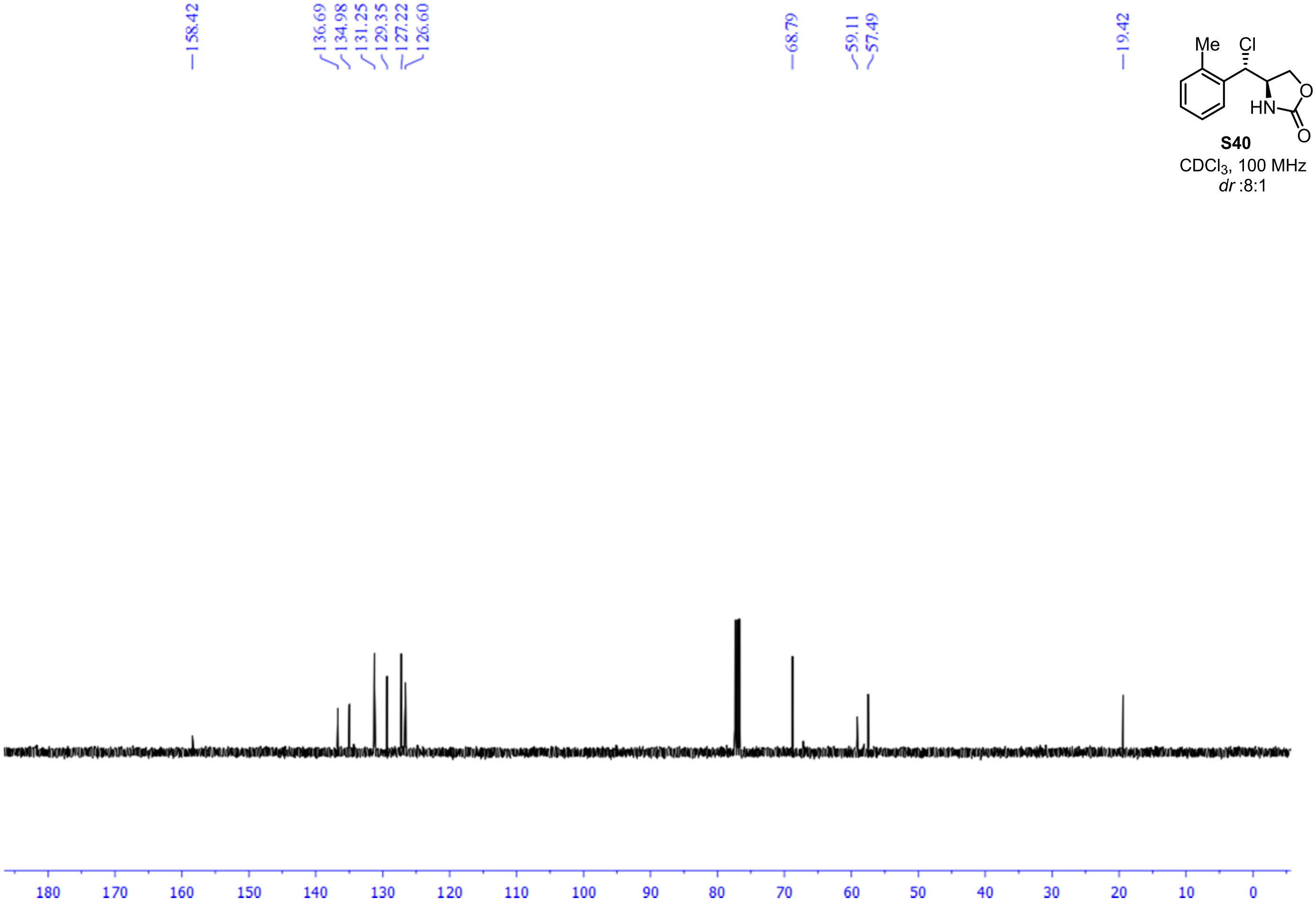

158.42
136.69
134.98
131.25
129.35
127.22
126.60
68.79
59.11
57.49
19.42
Me
Cl
O
HN
O
S40
CDCl3, 100 MHz
dr :8:1
180
170
160
150
140
130
120
110
100
90
80
70
60
50
40
30
20
10
0

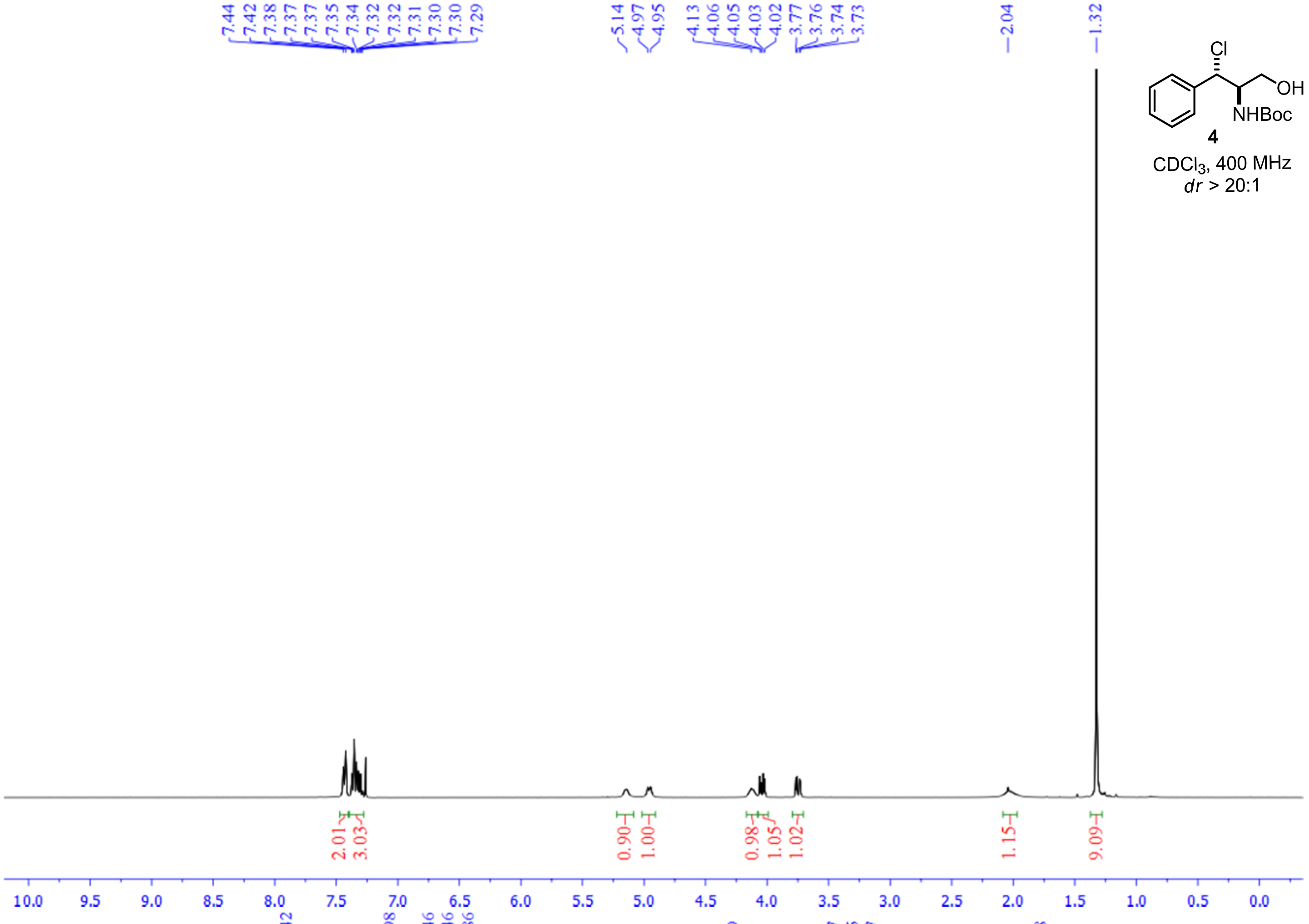
7.44
7.42
7.38
7.37
7.37
7.35
7.34
7.32
7.32
7.31
7.30
7.30
7.29
5.14
4.97
4.95
4.13
4.06
4.05
4.03
4.02
3.77
3.76
3.74
3.73
2.04
1.32
Cl
OH
NHBoc
4
CDCl3, 400 MHz
dr > 20:1
2.01
3.03
0.90
1.00
0.98
1.05
1.02
1.15
9.09

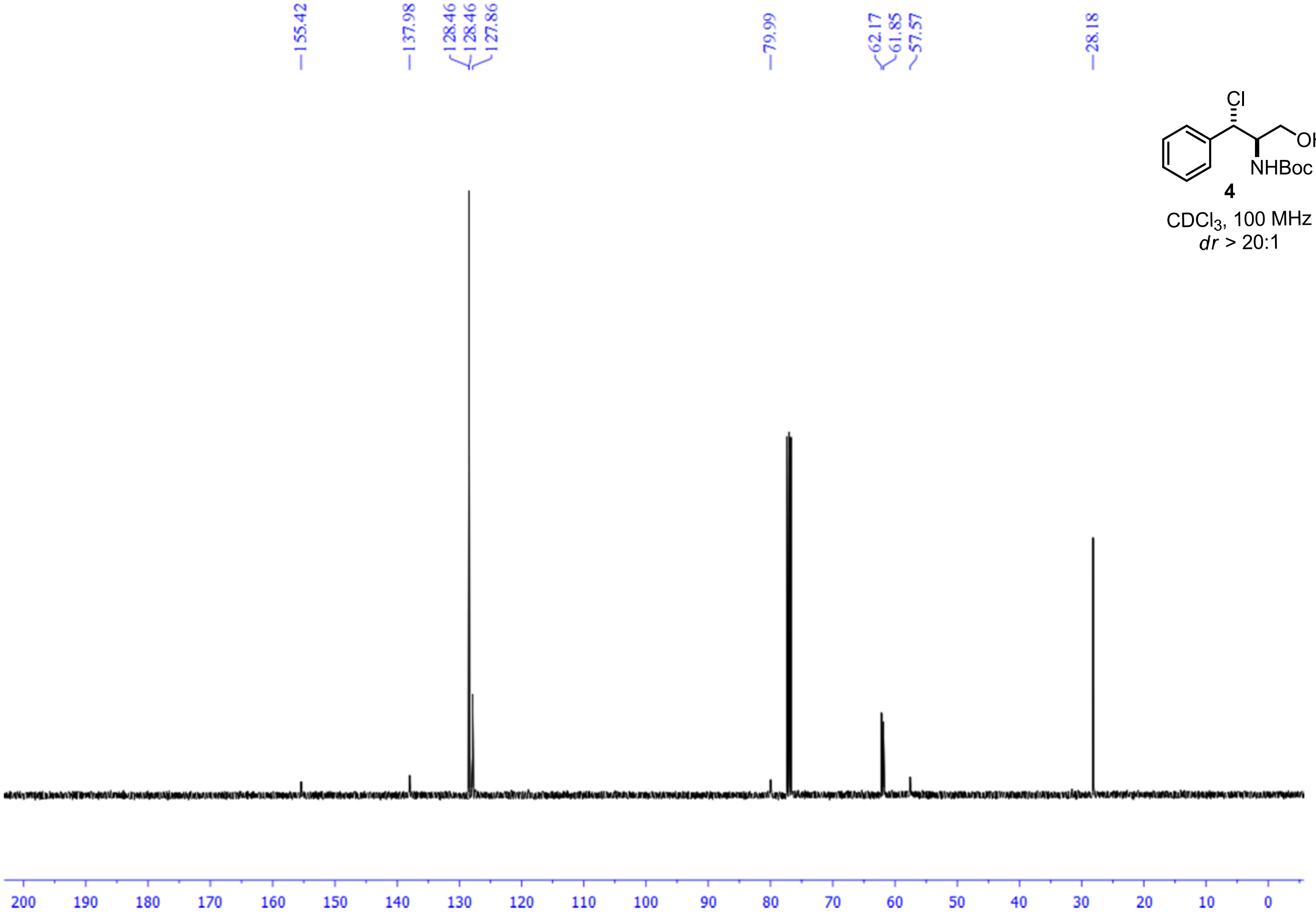
155.42
137.98
128.46
128.46
127.86
79.99
62.17
61.85
57.57
28.18
Cl
OH
NHBoc
4
CDCl3, 100 MHz
dr > 20:1
